# Novel Inhibitors of *Plasmodium* Phosphatidylinositol 4-kinase IIIβ with Low Propensity for Resistance: Life Cycle Stage Activity and *In Vivo* Efficacy in a Humanized Mouse Malaria Infection Model

**DOI:** 10.1021/acs.jmedchem.5c01417

**Published:** 2025-08-14

**Authors:** Godwin A. Dziwornu, Mmakwena M. Mmonwa, Dina Coertzen, Liezl Krugmann, Nicolaas Salomane, Meta Leshabane, Jean Thomas, Shante da Rocha, Janette Reader, Keabetswe Masike, Mathew Njoroge, Nicole Sevilleno, Rachael Coyle, Nonlawat Boonyalai, Emily Mayville, Marcus C. S. Lee, David A. Fidock, Lauren B. Coulson, John G. Woodland, Kathryn J. Wicht, Sandeep R. Ghorpade, Lyn-Marié Birkholtz, Kelly Chibale

**Affiliations:** † Holistic Drug Discovery and Development Centre (H3D), Department of Chemistry, 37716 University of Cape Town, Rondebosch 7701, South Africa; ‡ Department of Chemistry, University of Cape Town, Rondebosch 7701, South Africa; § Department of Biochemistry, Genetics and Microbiology, Institute for Sustainable Malaria Control, 56410 University of Pretoria, Hatfield, Pretoria 0028, South Africa; ∥ Holistic Drug Discovery and Development Centre (H3D), Institute of Infectious Disease and Molecular Medicine, University of Cape Town, Observatory, Cape Town 7925, South Africa; ⊥ 47665 Wellcome Sanger Institute, Wellcome Genome Campus, Hinxton CB10 1SA, U.K.; # Biological Chemistry and Drug Discovery, Wellcome Centre for Anti-Infectives Research, 3042 University of Dundee, Dundee DD1 5EH, U.K.; ∇ Department of Microbiology and Immunology, Columbia University Medical Center, Hammer Health Sciences Center, 701 W. 168th Street, New York, New York 10032, United States; ○ Center for Malaria Therapeutics and Antimicrobial Resistance, Division of Infectious Diseases, Department of Medicine, Columbia University Irving Medical Center, New York, New York 10032, United States; ◆ South African Medical Research Council Drug Discovery and Development Research Unit, Department of Chemistry and Institute of Infectious Disease and Molecular Medicine, University of Cape Town, Rondebosch 7701, South Africa; ¶ Department of Biochemistry, Stellenbosch University, Matieland, Stellenbosch 7601, South Africa

## Abstract

Anticancer ATP-competitive inhibitors are a promising source of new starting points for antimalarial drug discovery. Herein, we present a novel antimalarial chemotype based on the anticancer human ataxia-telangiectasia-mutated (ATM) kinase inhibitor AZD0156. This class inhibits phosphatidylinositol 4-kinase IIIβ (PI4K) in the human malaria parasite *Plasmodium*, demonstrating remarkable activities against all stages of the *Plasmodium falciparum* life cycle. The current series exhibited a lower propensity for resistance and toxicity compared to previous *Plasmodium* PI4K inhibitors. The lead compound **18** was efficacious in a humanized NOD-*scid IL-2Rγnull* mouse model of *P. falciparum* malaria, with an ED_90_ value of 4.6 mg kg^-1^.

## Graphical abstract

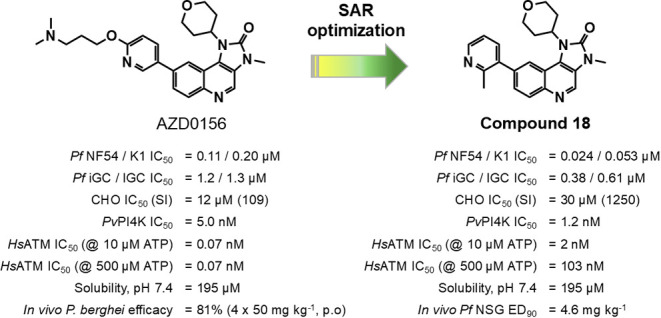

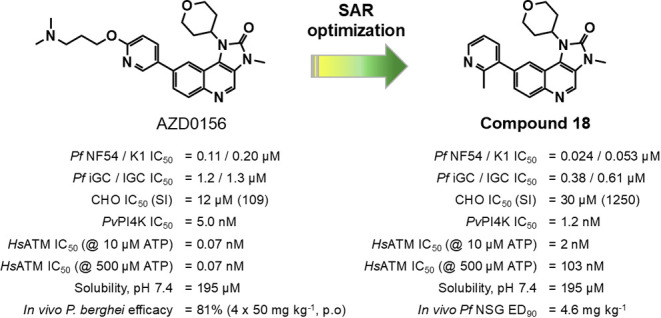

## Introduction

The emergence of resistance to current antimalarial drugs hampers progress toward the control and elimination of malaria.^1,2^ The artemisinin-based combination therapy (ACT) regimen is recommended as a first-line treatment for malaria.^1–3^ However, resistance toward the artemisinin component compromises the future efficacy of this regimen.^4–6^ The RTS,S/AS01 and R21/Matrix-M malaria vaccines, showing moderate efficacy, are expected to contribute to the control and eradication of malaria.^7,8^ Seasonal malaria chemoprevention (SMC) community-based intervention is highly effective in preventing malaria in areas with seasonal transmission. In clinical trials, the SMC regimen of sulfadoxine-pyrimethamine and amodiaquine (SPAQ) was highly effective, providing up to 88% protection against infection in the first 28 days and a 61% reduction in clinical malaria 29–42 days after administration.^9^ The current malaria prevention strategy focuses on long-acting oral and injectable drugs.^10^

Drug repurposing and repositioning approaches have shown potential in identifying antimalarial agents from known antiparasitic agents.^11^ Similarly, several anticancer agents have shown potential to be repurposed and/or repositioned for malaria.^11,12^ In spite of the safety concerns around anticancer chemotherapy, where high doses are typically administered for a prolonged period, anticancer agents have the potential to be repurposed and/or repositioned against acute malaria, whose treatment requires lower doses and a much shorter duration of treatment.

We have recently reported the antimalarial potential of the human ataxia-telangiectasia mutated (ATM) kinase inhibitor AZD0156 (Figure 1) through inhibition of the clinically validated target *P. falciparum* phosphatidylinositol 4-kinase IIIβ (*Pf*PI4K).^13^ Like other *Pf*PI4K inhibitors (Figure 1),^14–16^ AZD0156 exhibits activity against multiple stages of the *Plasmodium* life cycle (Table 1) and showed efficacy in the *Plasmodium berghei* mouse malaria infection model,^13^ making it a promising starting point for antimalarial drug discovery. Toward repositioning AZD0156 for malaria, we present here our first report of structure–activity relationship (SAR) studies based on the imidazo­[4,5-*c*]­quinolin-2-one core scaffold of AZD0156, leading to several compounds with improved antimalarial activity relative to the parent compound.

**Figure 1:**
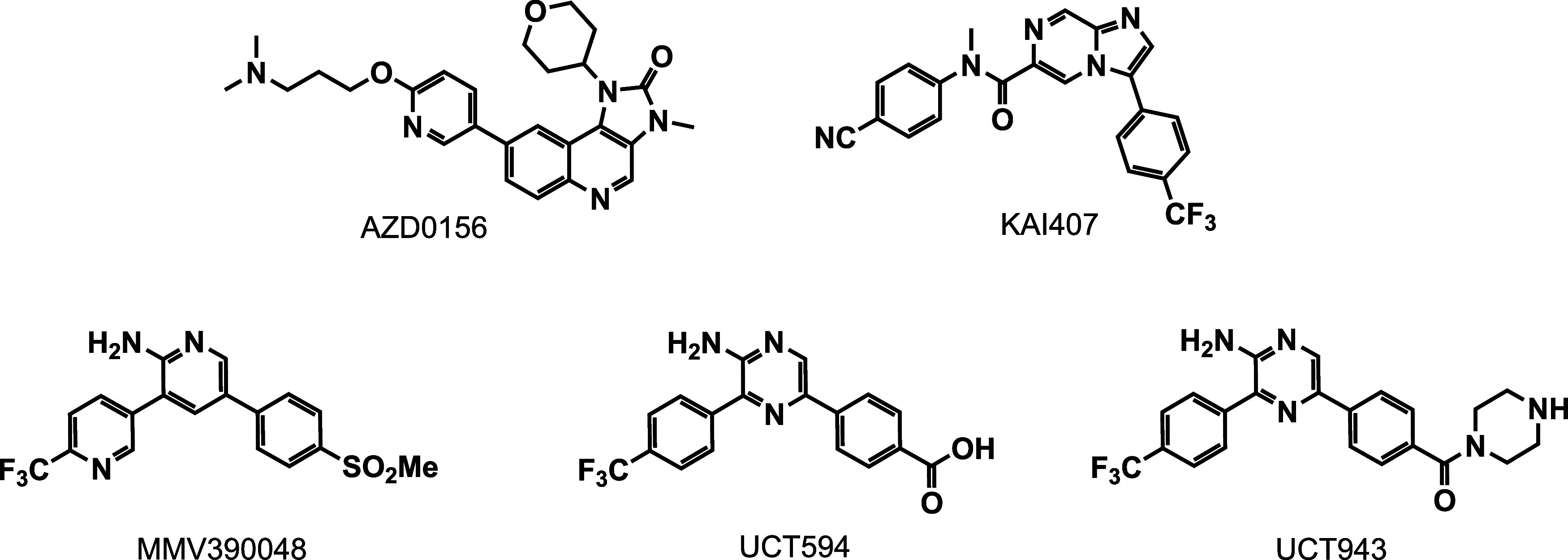
Structure of AZD0156 and examples of other *Pf*PI4K inhibitors.

**Table 1:**
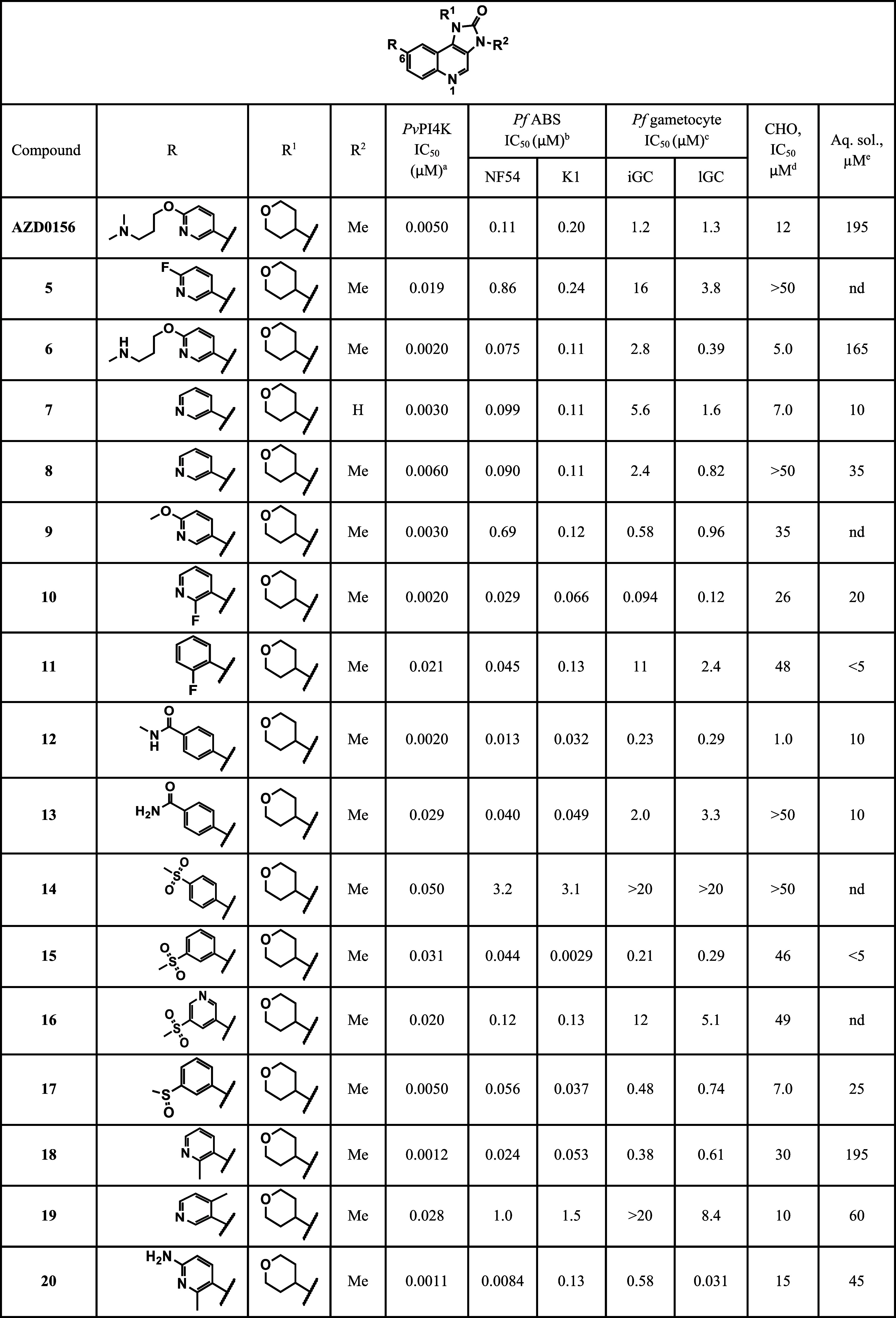

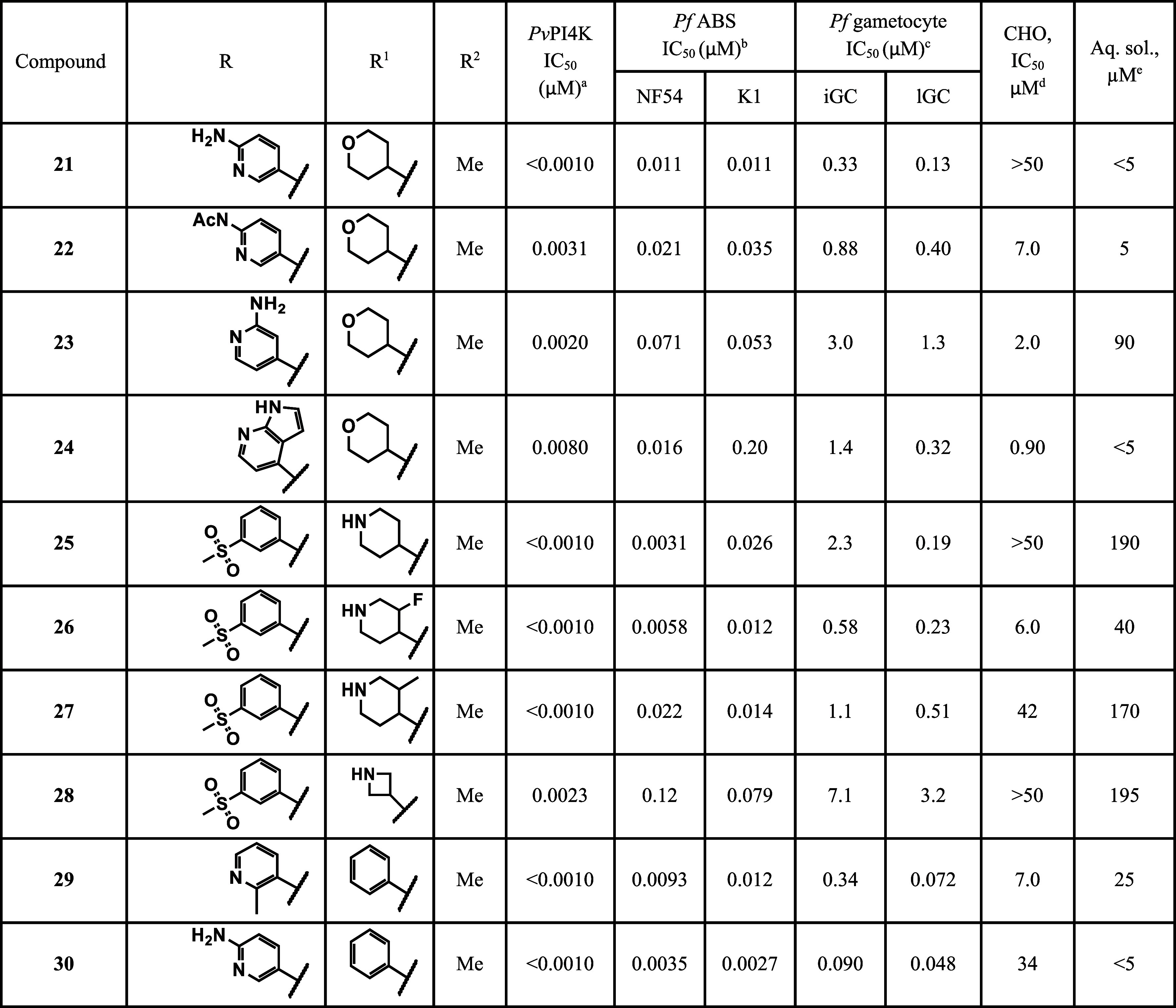
*In Vitro Pv*PI4K, Asexual Blood-Stage (ABS), and Gametocytocidal Activities against *P. falciparum*, and Toxicity Evaluation

nd: Not determined; ^a^Inhibition of purified recombinant *Pv*PI4K was determined in the presence of 10 μM ATP and ADP formation was quantified using the ADP-Glo Kinase assay kit; Sapanisertib at a concentration of 10 μM was used as the positive control (*Pv*PI4K IC_50_ = 4 nM). Mean IC_50_ values were calculated based on *n* ≥ 2 independent experiments, each with technical duplicates. IC_50_ < 1 nM means below the limit of detection of the assay. ^b^IC_50_ values were determined using a SYBR Green I parasite proliferation assay for 96 h, with means calculated from three independent experiments, each with technical triplicates; Chloroquine (*Pf*NF54/*Pf*K1 IC_50_ = 11/143 nM) was used as positive control. ^c^Gametocytocidal activity, iGC (immature gametocytes >90% stages II-III), lGC (late-stage gametocytes, >90% stages IV-V) measured by luciferase expression; Methylene blue (iGC/lGC IC_50_ = 190/900 nM) and MMV390048 (iGC/lGC IC_50_ = 215/134 nM) were used as positive controls. Data are from three biological repeats, performed in technical triplicates. ^d^Chinese hamster ovary cells tested as one biological replicate with technical triplicates; Emetine (IC_50_ <3 nM) was used as positive control. ^e^Thermodynamic Solubility at pH6. *In vitro* data (Mean IC_50_ values ± standard deviation (SD) or standard error (SE)) are provided as Supplementary information file.

## Results and Discussion

### Chemistry

As previously reported,^17^ amination of the 6-bromo-4-chloroquinoline-3-carboxylic acid ethyl ester through nucleophilic displacement of the 4-chloro group with selected amines afforded intermediate **1**, which upon ester hydrolysis under basic conditions gave the quinoline-carboxylic acid derivative **2** (Scheme 1). Curtius rearrangement of **2** with diphenylphosphoryl azide (DPPA) yielded the 8-bromoimidazoquinolines **3**, which was subsequently methylated to generate *N*-methyl-bromoimidazoquinolines **4**. Palladium-catalyzed Suzuki cross-coupling reaction of **4** with either boronic acids or pinacol esters led to the target compounds **8** – **30** (Table 1). Compound **5**, obtained by Suzuki coupling of 6-fluoro-3-pyridinylboronic acid with **4** (R^1^ = 4-tetrahydropyran, 4-THP), was reacted with *tert*-butyl 3-hydroxypropylmethylcarbamate and followed by the removal of the *tert*-butyloxycarbonyl group with trifluoroacetic acid to obtain compound **6**. Compound **7** was obtained by Suzuki coupling of **3** (R^1^ = 4-THP) with pyridine-3-boronic acid (Scheme 1). The synthesis of key intermediates is provided as Supporting Information.

**Scheme 1:**
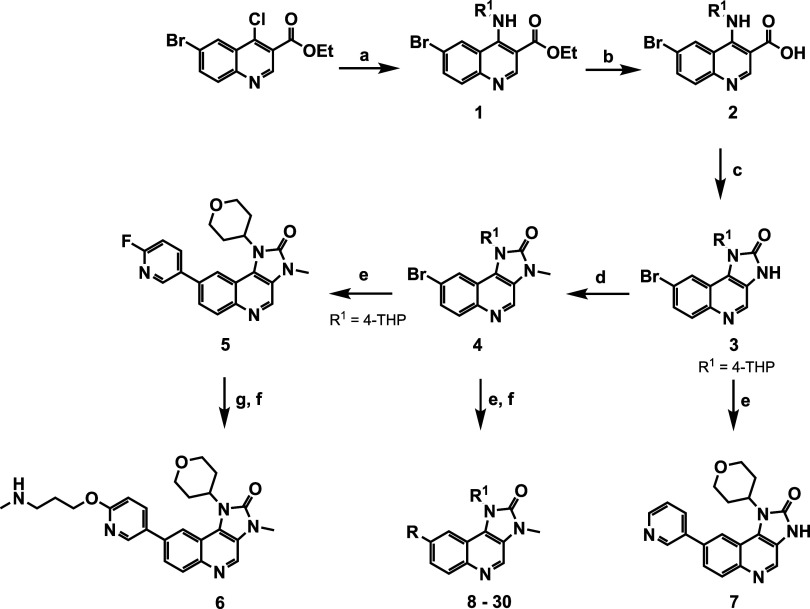
Synthesis of 1,8-Disubstituted Imidazo­[4,5-*c*]­quinolin-2-one Compounds^a^

a Reagents and reaction conditions: (a) relevant amine, DIPEA or NEt_3_, DMF or DMA, 25–90 °C, 3–16 h, 79–87%; (b) NaOH or LiOH, H_2_O, MeOH or THF, 25–70 °C, 1–6 h, 75–90%; (c) NEt_3_, DPPA, DMF, 60 °C, 0.5–3 h, 74–85%; (d) DMF-DMA, 90 °C, 2 h, 75–92%; (e) boronic acids or pinacol esters, PdCl_2_(PPh_3_)_2_, PCy_3_, K_2_CO_3_, DMF/dioxane:H_2_O (4:1, *v/v*), 100 °C, 1 to 2 h, 68–88%; (f) TFA, DCM, 25 °C, 1 h, 36 – 98% (for **6**, **25**-**28**); (g) *tert*-butyl (3-hydroxypropyl)­(methyl)­carbamate, NaH (60% dispersion in mineral oil), DMF, 30 °C, 3 h.

### SAR Exploration Strategies

To identify more potent antimalarial derivatives of AZD0156, we used our published *Pf*PI4K homology model^18^ to design compounds able to generate stronger molecular interactions in the *Pf*PI4K active site pocket (Figure 2). As depicted in Figure 2, a hydrogen bond (H-bond) between the quinoline nitrogen of AZD0156 and the hinge amide of V1357 is crucial for ligand binding. Further hydrophobic interactions between the quinoline ring and Y1356 maximize binding. The THP group of AZD0156 extends into the ribose pocket without any apparent polar interactions with S1362. The imidazolinone motif is exposed to the solvent front. The C6 alkoxypyridine extends into the catalytic site of ATP and makes critical H-bond interactions with K1308. The dimethylaminopropyloxy substituent on the C6 pyridine ring is the key structural feature of AZD0156, affording its high potency against the human ortholog *Hs*ATM through H-bond interactions between the terminal amine and a back pocket Y2969 in ATM.^17^ However, as this pocket is not accessible in *Pf*PI4K, the amino group of the dimethylaminopropyloxy substituent interacts with D1430. We hypothesized that new derivatives of AZD0156 lacking the dimethylaminopropyloxy substituent would exhibit lower inhibitory activity against *Hs*ATM and potentially be more selective toward the *Plasmodium* enzyme. Similarly, *Pf*PI4K bears a F827 on the phosphate-binding loop, contrary to L374 in the human ortholog, which could be exploited to achieve *Pf*PI4K selectivity through π-stacking interactions.

**Figure 2:**
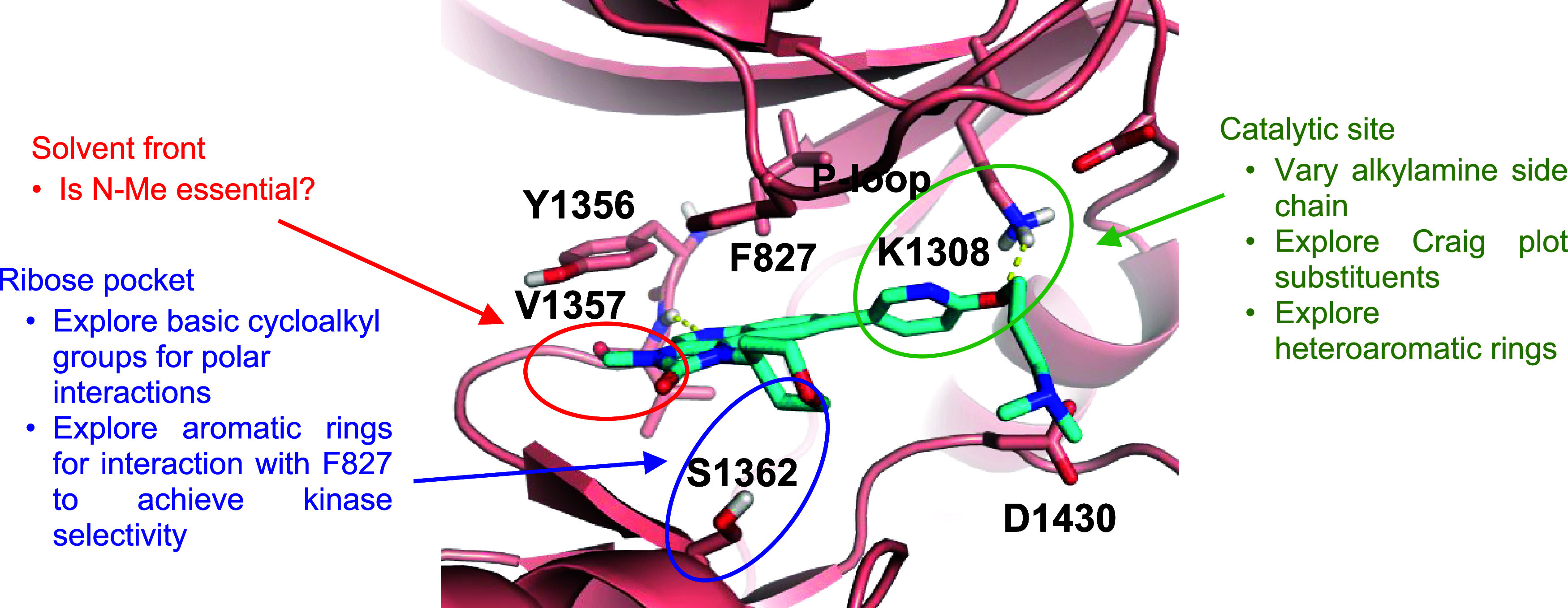
Binding mode of AZD0156 (cyan) in the *Pf*PI4K homology model and SAR exploration strategies. The *Pf*PI4K homology model was built off the human PI4K structure (PDB ID: 4D0L), as previously described.^18^

As summarized in Figure 2, the new analogs maintained the imidazoquinoline core scaffold. Extensive exploration focused on exploiting the catalytic site. The THP group was maintained in most of the new analogues (**5**–**24**, Table 1). The key learnings from the antiplasmodial SAR studies are described below.

### Multistage Antiplasmodial Activity

The *in vitro* antiplasmodial activity of the synthesized compounds was assessed against multiple stages of the *P. falciparum* life cycle, including the asexual blood stage (ABS) drug-sensitive (NF54) and multidrug-resistant (K1) strains, immature (>90% stages II–III) and late-stage (>90% stages IV–V) gametocytes (Table 1), as well as liver schizonts, gametes, and oocysts. The compounds were also tested in a *Plasmodium vivax* PI4Kβ (*Pv*PI4K) recombinant enzyme assay to confirm that they maintained PI4K as the primary target, with activity in the low nanomolar range (IC_50_ < 50 nM, Table 1). *Pv*PI4K shares a 97% sequence homology with *Pf*PI4K, sharing similar ATP-binding sites and catalytic regions, and the former can be expressed recombinantly.^18,19^ In general, *Pv*PI4K inhibition translated to whole-cell activity against ABS parasites. As expected, there is a general shift in potency between the *Pv*PI4K enzyme and whole-cell IC_50_ values. This can be explained, at least in part, by the high ATP concentrations in the cellular environment relative to the low ATP concentrations used in the enzyme assay. Binding to albumin in the cell culture medium will also reduce the free drug concentration in whole-cell assays in a compound-specific manner.

#### Asexual Blood-Stage Activity

The removal of the dimethylaminopropyloxy substituent yielded compound **8**, which retained both *Pv*PI4K inhibition (IC_50_ = 0.0060 μM) and ABS activity (IC_50_ *Pf*NF54/*Pf*K1 = 0.090/0.11 μM) comparable to AZD0156. Shortening the dimethylaminopropyloxy substituent to the methoxy in **9** retained *Pv*PI4K activity (IC_50_ = 0.0030 μM), but not *Pf*NF54 (IC_50_ = 0.69 μM). Compound **6** with a methylaminopropyloxy substituent maintained *Pv*PI4K inhibition and *Pf*NF54 activity, slightly more potent than AZD0156. Compound **7**, the *N*-demethylated imidazolinone derivative of **8**, showed comparable *Pv*PI4K inhibition (IC_50_ = 0.0030 μM) and ABS activity (*Pf*IC_50_ = 0.099–0.11 μM) to **8**.

The analogues (**8**–**24**) modified at the C-6 position of the imidazoquinoline core scaffold revealed the significant contributions of these modifications to ABS activity, in some cases regardless of the *Pv*PI4K activity. The SAR suggests that optimal catalytic site interactions (revealed as *Pv*PI4K inhibition) and consequent ABS activity of this series of analogues are influenced by first, efficient H-bonding with K1308 (Figure 2), which is maximized by the dihedral angle with the quinoline core (as in **5** vs **10**, and **18** vs **19**), and second, an aromatic C-6 group (as in **10** and **11**). That is, **5** (IC_50_ *Pv*PI4K/*Pf*NF54/*Pf*K1 = 0.019/0.86/0.24 μM) showed a marked loss in activity against *Pv*PI4K and ABS relative to its regioisomer **10** (IC_50_ *Pv*PI4K/*Pf*NF54/*Pf*K1 = 0.0020/0.029/0.066 μM). The difference in the activities of **5** and **10** may suggest that a potential twist in the dihedral angle due to the *ortho*-fluoro group in **10** orients the pyridyl N for optimal interactions with K1308, resulting in an improved activity. This hypothesis is corroborated by a similar observation between **18** (IC_50_ *Pv*PI4K/*Pf*NF54/*Pf*K1 = 0.0012/0.024/0.053 μM) and **19** (IC_50_ *Pv*PI4K/*Pf*NF54/*Pf*K1 = 0.028/1.0/1.5 μM). In this case, assuming that the twisting of the dihedral angle by an *ortho* substituent is unidirectional, the pyridyl N in **19** moves further away (opposite phase compared to **18**) from K1308, hence lacked H-bond interaction with loss of activity. In support of this hypothesis, the fluoropyridyl analogue **10** (IC_50_ *Pv*PI4K/*Pf*NF54 = 0.0020/0.029 μM) showed about 10-fold better *Pv*PI4K potency than the fluorophenyl **11** (IC_50_ *Pv*PI4K/*Pf*NF54 = 0.021/0.045 μM), although their ABS activity was within 2-fold. The potent ABS activity of **11** despite the lack of interaction with K1308 highlights the significant contribution of the C-6 aromatic group to whole-cell activity.

Based on these observations, we explored aromatic groups widely reported in *Pf*PI4K inhibitors (*e.g*., MMV390048 and KDU691) to leverage all possible interactions in the catalytic site for potency. These groups included phenyl amides (**12**, **13**), sulfones (**14**–**16**) and sulfoxides (**17**), aminopyridines (**20**–**23**), and an indole (**24**). The methylamide analogue **12** showed significant improvements in activity (IC_50_ *Pv*PI4K/*Pf*NF54/*Pf*K1 = 0.0020/0.013/0.032 μM) compared to AZD0156. Compound **13** showed marginal (<3-fold) loss in ABS activity and >10-fold loss in *Pv*PI4K potency compared to **12**. The phenyl amides showed superior activity over the 4-sulfonylphenyl congener **14** (IC_50_ *Pv*PI4K/*Pf*NF54/*Pf*K1 = 0.050/3.2/3.1 μM). However, the 3-sulfonylphenyl regioisomer **15** regained significant ABS potency (IC_50_ *Pv*PI4K/*Pf*NF54/*Pf*K1 = 0.031/0.044/0.0029 μM) comparable to **13**. The combination of the 3-pyridyl and *meta*-sulfonyl group as in **16** (IC_50_ *Pv*PI4K/*Pf*NF54/K1 = 0.020/0.12/0.13 μM) did not potentiate activity. Interestingly, the racemic 3-sulfinylphenyl analogue **17** showed an improvement in *Pv*PI4K inhibition (IC_50_ = 0.0050 μM) compared to the sulfone **15** (IC_50_ = 0.031 μM), indicating stronger interactions of the chiral sulfoxide with K1308 through optimal orientation. All the C-6 aminopyridine analogues (**20**–**23**) retained potent *Pv*PI4K inhibition (IC_50_ ≤ 0.0031 μM) and ABS activity (IC_50_ = 0.0084–0.13 μM). The indole derivative **24** showed comparative activity to the aminopyridine congeners.

Replacing the THP group with cycloalkylamines in **25**–**28** while keeping 3-methylsulfonylphenyl at the C-6 position significantly improved *Pv*PI4K inhibition (IC_50_ < 0.0023 μM) and ABS activity (IC_50_ ≤ 0.026 μM), as observed for the piperidine analogues (**25**–**27**). These modifications were supported by docking in *Pf*PI4K. Moreover, we envisaged that basic groups would improve the aqueous solubility of these analogs. The most active compounds in our studies were **29** and **30** with *Pv*PI4K activity below the limit of detection of the enzyme assay (IC_50_ < 0.0010 μM), translating to ABS IC_50_ values ≤ 0.012 μM in both *Pf*NF54 and K1 strains. These compounds demonstrate the contribution of the π-stacking interactions between the phenyl group and F827 (Figure 2) to *Pf*PI4K potency and ABS activity.

#### Gametocytocidal Activity

Analyses of the gametocytocidal activities in Table 1 reveal that the compounds were active against either one or both stages of gametocytes. SAR trends above were recapitulated for the gametocytocidal activities of the series, with the activity against late-stage gametocytes directly correlating with *Pv*PI4K inhibition and activity against ABS. Three active compounds (**20**, **29**, and **30**) against *Pv*PI4K (IC_50_ ≤ 0.001 μM) and ABS (IC_50_ < 10 μM) were also the most potent on late-stage gametocytes with IC_50_’*s* < 100 μM (lGC = 0.031/0.072/0.048 μM). These dual-stage activities against both ABS and gametocytes have been described for other *Pf*PI4K frontrunner chemotypes.^19^ However, as with the majority of other antimalarials, there is still a 4- to 8-fold drop in activity between ABS parasites and late-stage gametocytes as in **20** and **29**, although **30** nears equipotency. Overall, most compounds show <10-fold activity loss toward late-stage gametocytes, a property in line with those from other frontrunner antimalarial candidates, including the known *Pf*PI4K inhibitor MMV390048.^20^ With lipoidal uptake seen as the main barrier for dual-stage activity, compounds showing a > 19-fold loss in gametocyte activity could be due to poor compound uptake (i.e., **13**, **16, 23** and **28** > 75Å tPSA, with a 19- to 83-fold loss in activity, **11** and **19** LogD > 3, with an 8- to 53-fold loss in activity),^20^ rather than a difference in target availability between ABS parasites and gametocytes. Interestingly, some compounds (**6** and **8**), aminopyridines (**20**-**23**), indole (**24**), and the THP-modified compounds (**25**-**30**) displayed significant loss in activity to immature gametocytes but not late-stage gametocytes (iGC *p* = 0.02 compared to ABS, respectively, and lGC *p* = 0.7 compared to ABS, respectively, two-tailed *t* test). This is an unusual phenotype that has only been seen for one other *Pf*PI4K targeting compound, UCT943 (IC_50_ *Pf*NF54 = 6.9, iGC = 199, and lGC = 26 nM).^20^

Comparatively, **18** was selected as the best representative compound of the current series for further studies, as detailed below. This compound was potent against *Pv*PI4K, with comparable *Pf*NF54 and *Pf*K1, and gametocytocidal activities. Additionally, **18** possessed a better combination of aqueous solubility and cytotoxicity (Table 1).

#### Activity against Liver Schizonts, Gametes, and Oocysts

Compound **18** recorded a potent IC_50_ value of 0.065 μM (MMV390048, IC_50_ = 0.046 μM) against *P. falciparum* liver schizonts, which affords it the potential to block the development of schizonts into hypnozoites.^21^ These results inspired further studies to investigate the potential of the current compounds to halt the development of sexual parasite forms in the vector stage of the *Plasmodium* life cycle. First, we evaluated the ability of **18** to block transmission using the *P. falciparum* dual gamete formation assay (*Pf*DGFA) in the carry-over format. This assay measures the functional viability of male and female gametocytes and involves the treatment of stage V gametocytes to measure their ability to form gametes.^22^ In this assay, the late-stage gametocyte activity of **18** (IC_50_ = 0.61 μM) translated to blocking female gamete formation (IC_50_ = 0.87 μM), albeit to a lesser degree than that against male gamete formation (IC_50_ = 1.5 μM). In related studies, compound **10**, a close analogue of **18**, was 97% effective (at 2 μM) at inhibiting the formation of female gametes from mature gametocytes in the *P. falciparum* female gametocyte activation assay (*Pf*FGAA). Compound **10** recorded comparative activity in the male gamete exflagellation inhibition assay (EIA) by 99% (at 2 μM). Compounds **12** and **15** were equally effective at 100% in the *Pf*FGAA, and 92 and 99% at 2 μM in EIA, respectively.

Lastly, using the standard membrane feeding assay (SMFA) to assess the inhibition of oocyst formation in mosquitoes fed with gametocyte-infected blood, **15** reduced oocyst prevalence by 40% (transmission-blocking activity) and intensity by 69% (transmission-reducing activity). In summary, the current series demonstrates activity against all stages of the *Plasmodium* life cycle.

### *Pf*PI4K Is the Primary Target of **18**

We assessed the activity of the current series against field strains of *P. falciparum* and resistant mutant lines raised to compounds in development. In this regard, **17** and **18** showed a <2-fold difference in activity against field isolates relative to the wild-type NF54 strain (Table 2). They were also equally active (<2-fold IC_50_ shift) against laboratory mutants selected by DDD107498,^23^ SJ557733,^24^ KAF156,^25^ and ELQ300 ^26^ relative to the parental *Pf*Dd2 line. Compound **17** showed a 4-fold decrease in activity against the MMV390048-selected *Pf*PI4K S743T^14^ mutant, while **18** maintained a marginal 1.9-fold decrease.

**Table 2:** Activity against Field Isolates and Lab-Raised Resistant Lines of Known Antimalarials^a^

		**IC**_**50**_ **(μM)** (fold shift vs NF54)	
field isolates/strain	resistance profile	**17**	**18**
NF54	Wild type	0.059	0.024
K1	CQ, PYR, CYC, SDOX	0.062 (1.0)	0.053 (2.2)
3D7	Wild type	0.032 (0.5)	0.044 (1.8)
7G8	CQ, CYC, PYR	0.024 (0.4)	0.043 (1.8)
TM90C2B	ATO, CYC, PYR	0.034 (0.6)	0.043 (1.8)
RF12	PPQ	0.024 (0.4)	0.024 (1.0)
Dd2	CQ, CYC, PYR, SDOX	0.035 (0.6)	0.035 (1.4)

lab-raised mutants	mutated locus	**IC**_**50**_ **(μM)** (fold shift vs Dd2)	
Dd2 DDD107498	*Pf*eEF2_Y186N	0.051 (1.4)	0.051 (1.4)
Dd2MMV390048	*Pf*PI4K_S743T	0.15 (4.4)	0.068 (1.9)
Dd2 SJ557733	*Pf*ATP4_G358S	0.039 (1.1)	0.039 (1.1)
Dd2 KAF156	*Pf*CARL_I1139K	0.034 (1.0)	0.034 (1.0)
Dd2 ELQ300	*Pf*CYTB_I22L	0.059 (1.7)	0.059 (1.7)

a The fold change in IC_50_ relative to the reference strain is shown in brackets (chloroquine, CQ; Pyrimethamine, PYR; Cycloguanil, CYC; Sulfadoxine, SDOX; Atovaquone, ATO; Piperaquine, PPQ). IC_50_’s are mean values from at least 2 independent biological replicates using the 72 h [^3^H]-hypoxanthine incorporation assay. The individual values varied less than 2-fold. Means were calculated from 2 independent experiments each with four technical repeats. RF12 has the additional H97Y mutation in *PfCRT*, which confers PPQ resistance as well as CQ resistance.

To confirm that *Pf*PI4K is the primary target of the current series, we explored the antimalarial resistome barcode sequencing (AReBar) platform^27^ for cross-resistance profiling against a pool of over 50 genetically barcoded drug-resistant parasite lines in both the Dd2 and 3D7 background and the two parent lines. The pool, generated using CRISPR/Cas9 genome editing to insert a short barcode cassette into the nonessential *PARE* locus of each parasite line, affords a range of targets and resistance mechanisms to be evaluated (Table S1). Under drug pressure of 3× the Dd2 IC_50_, the pool exhibited a rapid growth recovery on days 7 and 5 for **17** and **18**, respectively (Figure S2a). Both compounds showed a log2 fold change >2.5 for only the *Pf*PI4K S1320L+L1418F mutant parasite line originally selected by KAI407, an imidazopyrazine *Pf*PI4K inhibitor (Figure S2b,c).^16^ Clones raised to AZD0156 showed a S1320L mutation in *Pf*PI4K.^13^ The *Pf*PI4K S1320L+L1418F mutant showed a significant loss in sensitivity to **17** and **18** relative to the Dd2 line (Figure 3). The *Pf*PI4K S743T+H1484Y mutant line was comparatively more sensitive to the two compounds than the former mutant line. These data support PI4K as the target of **17** and **18** in *Plasmodium*, as is AZD0156.

**Figure 3:**
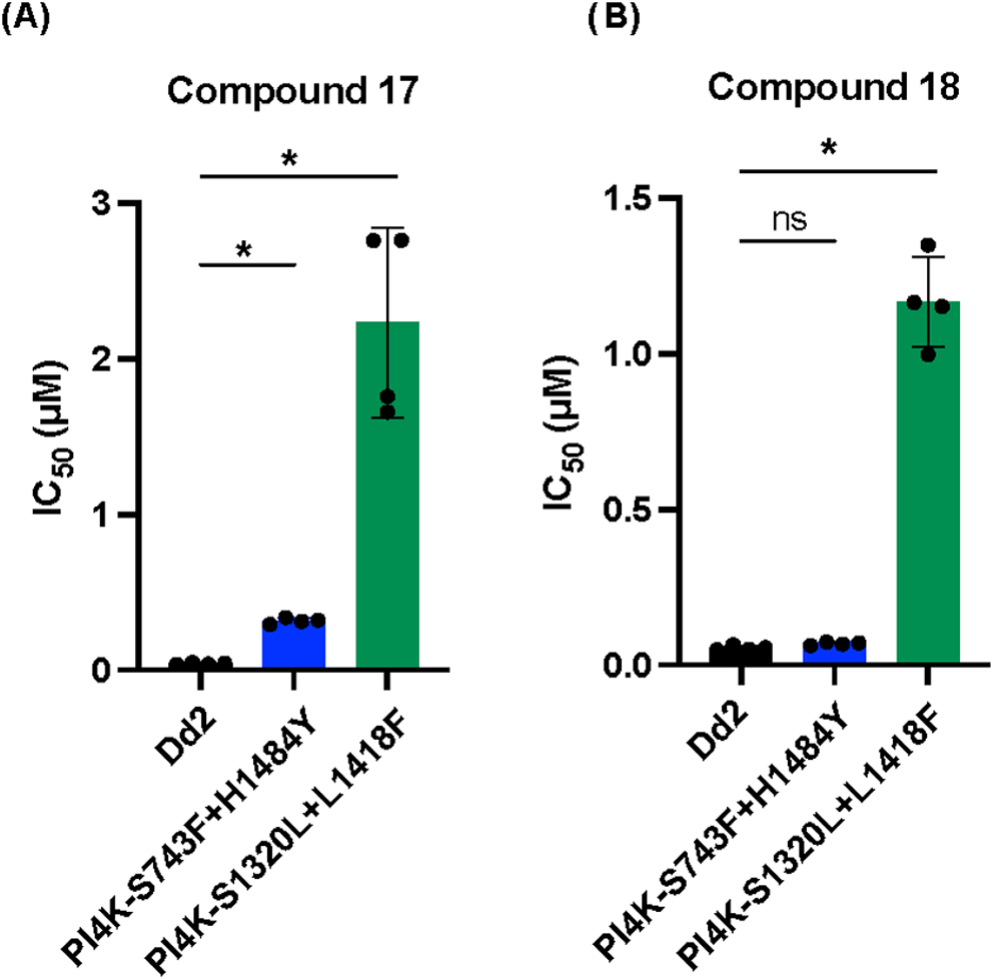
IC_50_ values for (A) **17** and (B) **18** against the parental Dd2 line and the two PI4K mutant lines in the AReBar pool, PI4K–S743F+H1484Y, and PI4K–S1320L+L1418F. EC_50_ data represent means from 4 biological replicates (technical triplicates), with error bars showing standard deviation.

A major drawback in the development of *Pf*PI4K inhibitors is their propensity to generate resistance arising from mutations in the target, raising concerns about how they will fare in the field. For example, at least nine different *Pf*PI4K single-point mutations within the kinase domain as well as PI4K copy number variations and a single-point mutation in Rab11a have been reported for laboratory-derived resistant lines generated against a range of *Pf*PI4K inhibitors.^14–16,28,29^ A recent analysis by researchers in the Malaria Drug Accelerator (MalDA), a consortium aimed to improve and accelerate the early antimalarial drug discovery process by identifying new, essential, druggable targets,^30^ classified *Pf*PI4K among deprioritised targets for the reasons above. The propensity for resistance *in vitro* for most *Pf*PI4K inhibitors falls within the moderate range with a minimum inoculum for resistance (MIR) value between 10^6^ and 10^7^.^14,15^

The MIR of **18** was studied by using the *P. falciparum* Dd2-B2 clone. One MIR single-step selection was set up using 3.3 × 10^6^ Dd2-B2 ABS parasites in 24 wells for a total of 7.9 × 10^7^ parasites, at a starting concentration of 3 × IC_90_ (192 nM). Each well had 2 mL of culture with an initial parasitemia of 1 at 3% hematocrit. Parasite clearance was observed on day 7. The selection had a consistent drug pressure of 3× IC_90_ (192 nM) for 35 days, and cultures were screened three times weekly by flow cytometry and smearing. Wells were considered positive for recrudescence when the overall parasitemia reached 0.3% and parasites were seen on a blood smear. No recrudescence was observed over the course of this selection (MIR > 7.9 × 10^7^). The low propensity for resistance observed for **18**, relative to other *Pf*PI4K inhibitors such as MMV390048 (MIR 10^6^–10^7^), makes it a promising antimalarial chemotype and provides further evidence that the resistance risk associated with a particular target can vary based on chemotype.^31^

Lastly, like MMV390048 and UCT943 (Figure 1),^14^ **17** and **18** displayed a slow parasite reduction ratio (PRR) profile slower than pyrimethamine (log PRR 3.4). Compounds **17** and **18** recorded an *in vitro* log PPR value of 2.4 and 2.6, respectively, comparable to that of MMV390048 (log PRR = 2.7). The parasite clearance time is in the range of 66–69 h (Figure S1). No lag phase was detected for **17**, but a 24 h lag phase was detected for **18**. PRR studies predict how quickly a drug would relieve clinical symptoms as a measure of parasite load. Although slow *in vitro*, MMV390048 showed a moderate rate of kill *in vivo* but a fast-killing profile in the clinic.^29^

### Cytotoxicity and hERG

Compound **18** had good safety indices against mammalian cancer cell lines and showed a low human ether-a-go-go-related gene (hERG) cardiotoxicity risk (Table 3). Inhibition of hERG channels may lead to life-threatening cardiac arrhythmias through QT prolongation, which has been associated with many drugs and resulted in their withdrawal from clinical development. Like previous *Pf*PI4K inhibitors, **18** showed potential off-target kinase liabilities based on *in vitro* human kinase inhibition data for a small panel of key kinase off-targets as described below (Table 3).

**Table 3:** Key Toxicity Profile of Compound **18**

	AZD0156	**18**	MMV390048 ^14,15^
*Pf*NF54/K1, IC_50_, μM	0.11/0.19	0.024/0.053	0.028/0.025
*Pv*PI4K, IC_50_, μM @ 10 μM ATP	0.0052	0.0012	0.0010
*Pv*PI4K, IC_50_, μM @ 500 μM ATP	0.008	0.003	nd
CHO, IC_50_ μM (SI)^a^	12 (109)	30 (1250)	>254 (9071)
HepG2, IC_50_ μM (SI)^a^	4.1% @ 2 μM	18.2 (758)	>100 (3571)
L6, IC_50_ μM (SI)^a^	nd	47.8 (1991)	nd
Vero, IC_50_ μM (SI)^a^	nd	>50 (>2083)	nd
hERG IC_50_, μM (SI)^a^	>30 (>96)	17.2 (716)	>11 (>393)
*Hs*ATM IC_50_, μM (SI)^b^ @ 10 μM ATP	0.00007 (0.035)	0.002 (2)	nd
*Hs*ATM IC_50_, μM (SI)^b^ @ 500 μM ATP	0.00007 (0.008)	0.103 (34)	nd
*Hs*PI3Kα IC_50_, μM (SI)^b^ @ 10 μM ATP	0.32 (160)	0.53 (530)	7.80 (7800)
*Hs*PI4Kβ IC_50_, μM (SI)^b^ @ 10 μM ATP	1.22 (600)	0.30 (300)	1.00 (1000)
*Hs*MINK1 IC_50_, μM (SI)^b^@ 10 μM ATP	>10 (>5000)	3.14 (3140)	0.80 (810)
*Hs*MAP4K4 IC_50_, μM (SI)^b^ @ 10 μM ATP	>10 (>5000)	3.17 (3170)	0.70 (740)

a Selectivity index (SI = IC_50_/*Pf*NF54 IC_50_;

b SI = IC_50_/*Pv*PI4K IC_50_). Mean *Pv*PI4K IC_50_ values were calculated based on two independent experiments where the 10 μM and 500 μM ATP conditions were included in the same assay using the same inhibitor dilution to enable direct comparison. CHO: Chinese Hamster Ovary cells; HepG2: Human hepatoblastoma cell line; L6: Rat myoblast cell line; Vero: Monkey kidney epithelial cells; hERG: Human ether-a-go-go-related gene; *Hs*MINK1: human misshapen-like kinase 1; *Hs*MAP4K4: human mitogen-activated protein kinase 4; nd: Not determined.

### Human Kinase Profiles

#### Compound **18** and the PIKKs

AZD0156 shows significant selectivity toward *Hs*ATM compared to other members (such as ATR, DNA-PK, mTOR)^17^ of the phosphatidylinositol 3-kinase-related kinase (PIKK) family. Although the PIKKs are a family of serine/threonine kinases, as the name suggests, they are structurally similar to the phosphatidylinositol 3-kinases (PI3Ks).^32^ Hence, we tested AZD0156 and **18** against both *Hs*ATM and *Hs*PI3Kα to gain insight into the kinase selectivity. Like AZD0156, **18** showed moderate inhibition of *Hs*PI3Kα, with good selectivity for *Pv*PI4K. Although compound **18** retained potency against *Hs*ATM (IC_50_ = 2 nM), a 30-fold reduction in potency was observed relative to that of AZD0156 (IC_50_ = 0.07 nM).

Given the marked difference in the ATP Michaelis constant (*K*_m_^ATP^) values for *Pv*PI4K (*K*_m_^ATP^ = 300 μM) and *Hs*ATM (*K*_m_^ATP^ = 10 μM), we investigated the activity of **18** at a higher ATP concentration in both the *Pv*PI4K and *Hs*ATM biochemical assays. Under these conditions, the *Pv*PI4K IC_50_ values for **18** and AZD0156 at 10 and 500 μM were within 2- to 4-fold, in line with what is expected based on the Cheng–Prusoff equation (*K*_i_ = IC_50_/(1 + [S]/*K*_m_), where *K*_i_ is the inhibition constant, and [S] is the ATP concentration). AZD0156, which has been reported to retain subnanomolar potency in a cell-based ATM inhibition assay,^17^ displayed an *Hs*ATM IC_50_ value of 0.07 nM in the presence of both 10 μM and 500 μM ATP. This is most likely because the IC_50_ value for AZD0156 is at the lower limit of detection of the *in vitro* enzyme assay, so the expected increase in IC_50_ at higher ATP concentration is not observed. In contrast, **18** showed ∼50-fold higher IC_50_ (*Hs*ATM IC_50_ = 103 nM) at 500 μM ATP than at 10 μM ATP (*Hs*ATM IC_50_ = 2 nM), as expected based on the Cheng–Prusoff equation. Compared to AZD0156, **18** is only ∼28-fold less potent against *Hs*ATM at 10 μM ATP (AZD0156/**18** IC_50_ = 0.07/2 nM), but ∼ 1470-fold less potent against *Hs*ATM at 500 μM ATP (AZD0156/**18** IC_50_ = 0.07/103 nM). We hypothesize that **18** would show much lower *Hs*ATM inhibitory activity under cellular conditions where the ATP concentration is much higher than 500 μM.^33^ In contrast, the shift in IC_50_ for *Pv*PI4K is expected to be lower due to the higher *K*_m_^ATP^.

In Figure 4, **18** shows similar interactions with the amino acid residues in the ATP-binding site of *Pf*PI4K and *Hs*ATM. However, subtle differences could be explored to achieve selectivity. For example, *Pf*PI4K has a small S1362 compared to a larger Q2874 of *Hs*ATM. New analogues designed to incorporate bulky groups extending into the ribose pocket could disrupt binding in *Hs*ATM but could be accommodated in *Pf*PI4K, thereby achieving selectivity at the enzyme level.

**Figure 4:**
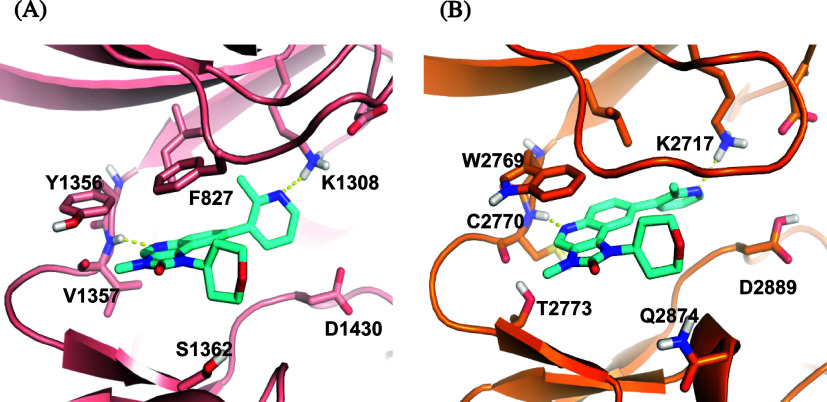
Binding mode of **18** in (A) *Pf*PI4K homology model and (B) *Hs*ATM. The *Pf*PI4K homology model was built off the human PI4K structure (PDB ID: 4D0L), as previously described.^18^ The *Hs*ATM crystal structure (PDB ID 7NI4) was used.^34^

#### Compound **18** and HsPI4K, MINK1, and MAP4K4

A recent study on MMV390048 hypothesized a potential link between the inhibition of *Hs*PI4K and *Hs*MINK1 and/or *Hs*MAP4K4 and the *in vivo* malformations observed in embryofetal developmental studies in rats (but not rabbits).^35^ Compound **18** showed ∼4-fold reduction in potency for *Hs*MINK1 and *Hs*MAP4K4, relative to MMV390048, while AZD0156 was inactive (IC_50_ > 10 μM, Table 3). AZD0156 and MMV390048 showed comparable inhibition of *Hs*PI4K (IC_50_ ∼ 1 μM), while **18** was ∼3-fold more potent, highlighting the need to improve the selectivity of future congeners in relation to *Hs*PI4K. *Plasmodium* and human PI4K have similar ATP-binding sites and *K*_m_ values. As described for *Hs*ATM (Figure 4), it has been speculated that the selectivity of MMV390048 over *Hs*PI4K is due to a clash between the pyridyl-CF_3_ group and the bulky ribose pocket Q606 residue.^18^ These hypotheses (that is, exploring bulky groups predicted to extend into the ribose pocket of the *Hs*PI4K and *Hs*ATM ATP-binding sites and testing the analogues in a cell-based *Hs*ATM assay) are the subject of ongoing work in our laboratories.

### *In Vitro* and *In Vivo* DMPK

Compound **18** showed an excellent *in vitro* ADME profile to support *in vivo* pharmacokinetic studies in rodents (mouse and rat) (Table 4). Consistent with the *in vitro* data, the unbound clearance of **18** was low across the species. Further to this, **18** showed significantly lower unbound clearance in mice compared to AZD0156. The low unbound clearance of **18** resulted in higher free drug concentration relative to AZD0156 (free AUC = 380.9 and 42.7 min.μmol/L for **18** and AZD0156, respectively, in mouse at a 10 mg/kg oral dose). Absorption was complete (bioavailability = 100%) in rats but lower in mice (55%) due to the lower unbound clearance in rats compared to mice.

**Table 4:** *In Vitro* and *In Vivo* DMPK Parameters^a^

compound	AZD0156	**18**
NF54/K1, IC_50_, μM	0.11/0.19	0.024/0.053
solubility PBS pH 6.5 (μM)/LogD/tPSA	195/1.26/71	195/2.45/58
PAMPA Papp (10^–6^ cm/s)	20	340
Caco-2 Papp A > B (10^–6^ cm/s)	nd	23
efflux ratio	nd	1
PPB fu h/m	0.22/0.05	0.12/0.42
microsomal CL_int_, app h/r/m (mL/min/kg)	<10/<21/<46	<10/<21/<46
hepatocyte CL_int_, app h/r (mL/min/kg)	<15/nd	<15/<30
*in vivo* mouse CL_b_/CL_u_ (mL/min/kg)	15.3/306	16.3/38.8
*in vivo* rat CL_b_/CL_u_ (mL/min/kg)	nd	4.6/10.9
*t*_1/2_ terminal r/m (h)	nd/4.1	4.1/9.0
V_ss_/V_ss,u_ mouse (L/kg)	3.5/70	3.9/9
V_ss_/V_ss,u_ rat (L/kg)	nd	1.8/4
AUC_0–t_ r/m (min.μM)	nd/853	7172/907
F r/m (%)	nd/50	100/55
F_abs_ r/m	nd/0.56	1.0/0.63

a *In vivo* mouse PK parameters calculated from noncompartmental analysis of intravenous dosing at 3 mg kg^-1^; tPSA: total polar surface area; h/r/m: Human/Rat/Mouse; PPB: Plasma protein binding; fu: Fraction unbound; Vss: Apparent volume of distribution at steady state; Vss,u: Unbound apparent volume of distribution at steady state; CL_b_: Total body clearance determined from whole blood; CL_u_: Unbound blood clearance determined from CL_b_ and plasma protein binding (assuming blood to plasma ratio of 1). AUC: Area under the curve; not determined (nd).

### *In Vivo* Efficacy of **18**

Compound **18** was tested in a humanized NOD-*scid IL-2Rγnull* (NSG) mouse model of malaria by administering quadruple ascending oral doses of 1, 3, 10, and 30 mg kg^-1^ 3 days postinfection. These quadruple doses were administered 24 h apart. The blood samples from treated mice were screened for drug concentration and parasitemia every 24 h from day 3 until day 7 post-treatment to generate the pharmacokinetic and pharmacodynamic parameters (Figure 5). The compound showed an ED_90_, the effective dose in mg kg^-1^ that reduces parasitemia by 90% on day 7 postinfection compared to the untreated control group, of 4.6 mg kg^-1^ with the corresponding oral exposure levels (AUC_ED90_) of 22.7 μM.hr.

**Figure 5:**
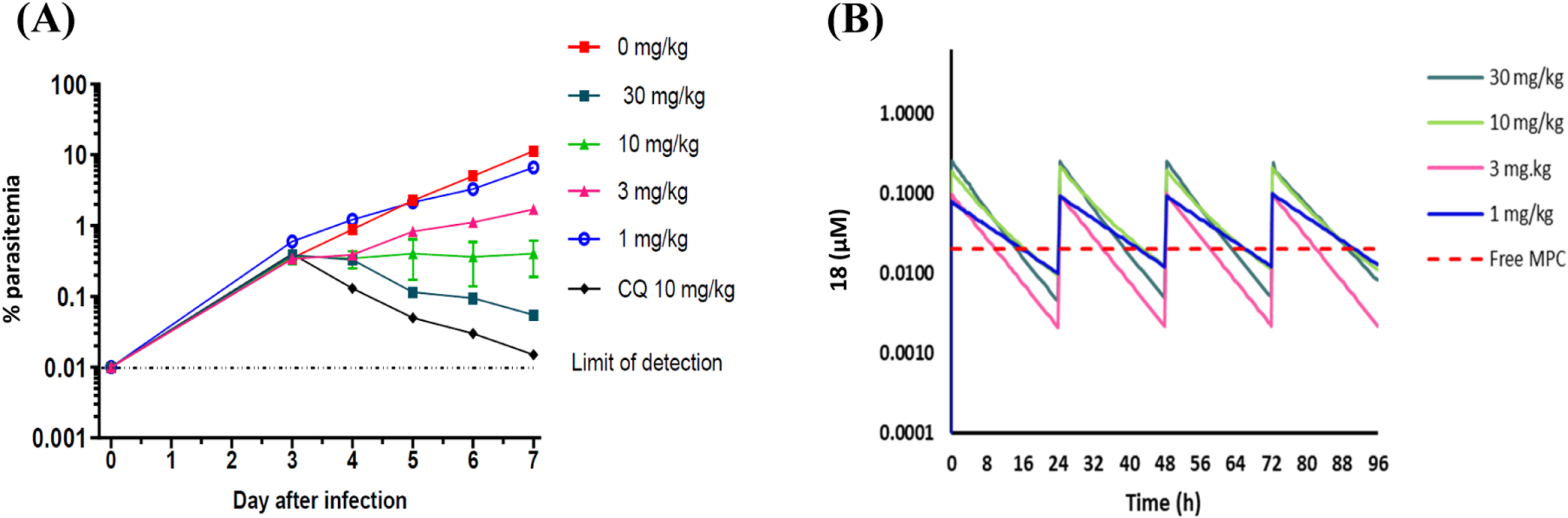
(A) *In vivo* therapeutic efficacy of **18** in the humanized NSG mice infected with *P. falciparum* *Pf*3D7^0087/N9^ cells and (B) the free plasma concentrations vs time in NSG mice following a quadruple dose regimen. The red dashed line represents the free *in vitro* minimum parasiticidal concentration (MPC) of compound **18**.

## Conclusions

New affordable antimalarial drugs with a low propensity for resistance are needed to circumvent the rapid rise in resistance toward ACTs. In addition, blocking transmission of *Plasmodium* parasites from humans to mosquitoes is essential for malaria elimination as set out in the World Health Organization Global Technical Strategy for Malaria 2016–2030.^36^ Therefore, developing drugs that can target the *Plasmodium* parasites at the asexual blood stage to ease symptoms and block human-to-mosquito transmission is critical for malaria control and elimination. The AZD0156-based PI4K inhibitors described in this study show the potential for further optimization to identify clinical candidates that would be effective against both asexual and sexual life stages of malaria in achieving this goal. In this regard, further work is ongoing to improve the antimalarial potential of **18** with a special focus on improving the selectivity relative to the human ATM, PI3, and PI4 kinases.

## Experimental Section

### Modeling

All docking simulations were run on the *Pf*PI4K homology model^18^ or structures downloaded from the PDB. Protein structures were all processed using the Maestro GUI of the Schrodinger 2023–4 (Schrödinger Release 2023–4: Schrödinger, LLC, New York, NY, 2023) software suite. Protein structures were prepared using the Schrodinger Protein Preparation wizard using default preprocessing, H-bond optimization, and minimization settings. Crystallization artifacts were manually removed, and residues with alternate positions were manually assigned. The prepared ligand binding site was then visually inspected for the correct tautomer and H-bond assignment.

Docking grids were then prepared using the GLIDE Receptor Grid Generation tool with a grid centered upon previously docked ligands in the active site or the crystallized ligand from *Hs*PI4K.^37^ A hydrogen bonding constraint on the hinge H-bond donating amide (*Pf*PI4K V1357, *Hs*PI4K V598, *Hs*ATM C2770) was also set. All ligands were prepared from SMILES using LigPrep with ionization states determined to a pH of 7 ± 1.0, calculated using the Epik and the OPLS4 atomic force field.^38^ The prepared ligands were then docked into the prepared docking grids by using SP docking. All docking images were generated using open source PyMOL 2.5.0 (Schrodinger, LLC. 2010. The PyMOL Molecular Graphics System, Version 2.5.0).

### Chemistry

All commercially available chemicals were purchased from Sigma-Aldrich, Merck, or Combi-Blocks Limited and were of >95% purity, thus generally used without further purification. Reactions were monitored with LCMS. Compounds were purified on silica gel chromatography using flash column chromatography on a Biotage Isolera One system (Biotage AB, Uppsala, Sweden). ^1^H NMR spectra were recorded on a Varian Mercury (300 MHz) or Bruker (600 MHz). ^13^C NMR spectra were recorded on the Bruker Ultrashield-Plus (101 MHz) or a Bruker (151 MHz). NMR samples were dissolved in deuterated dimethyl sulfoxide (DMSO-*d*_6_), chloroform (CDCl_3_), or methanol (CD_3_OD). Chemical shifts (δ) are reported in parts per million (ppm) and rounded to two decimal places. Coupling constants (*J*) are reported in hertz (Hz) and rounded to two decimal places. Abbreviations used in assigning ^1^H NMR signals are d (doublet), dd (doublet of doublets), ddd (doublet of doublets of doublets), m (multiplet), q (quartet), s (singlet), t (triplet), and td (triplet of doublets).

Purity was determined using an Agilent 1260 Infinity binary pump, Agilent 1260 Infinity diode array detector (DAD), Agilent 1290 Infinity column compartment, Agilent 1260 Infinity standard autosampler, and Agilent 6120 quadrupole (single) mass spectrometer equipped with an ESI ionization source. LC purity analyses were performed using a Kinetex 1.7 μM C18 reverse phase column, 1 μL injection volume, flow 1.2 mL/min; gradient: 5–100% B in 1.5 min (hold 0.4 min), 100–5% in 0.3 min (hold 0.5 min) (Mobile phase A: 0.1% formic acid in H_2_O and Mobile phase B: 0.1% formic acid in acetonitrile). The diode array detector was programmed to scan the eluents at an absorption wavelength range of 210–640 nm. All synthesized intermediates were characterized by LCMS, while final compounds were confirmed by LCMS and, at least, ^1^H NMR data. All compounds tested for biological activity were confirmed to have >95% purity. Intermediates **2–4** were synthesized as previously described.^17^

### General Synthesis Methods

Method A: 8-Bromo-3-methyl-1-(tetrahydro-2*H*-pyran-4-yl)-1,3-dihydro-2*H*-imidazo­[4,5-*c*]­quinolin-2-one **4** (0.100 g, 0.28 mmol), the appropriate boronic acid/boronic acid pinacol ester (1.50 equiv), PdCl_2_(PPh_3_)_2_ (0.007 g, 0.01 mmol), PCy_3_ (0.008 mg, 0.03 mmol), and K_2_CO_3_ (0.057 g, 0.41 mmol) were placed in a round-bottom flask equipped with a stirrer bar and a condenser. DMF/dioxane/water (4:1 *v/v*, 75 mL/mmol of **4**) was added, and the mixture was purged with nitrogen gas for 10 min. A balloon filled with nitrogen gas was connected to the top of the condenser, and the mixture was stirred at 100 °C for 2 h or until the reaction was complete (LCMS monitored). After the completion of the reaction, the mixture was cooled to ambient temperature and then quenched with ice-cold water. The precipitate formed was obtained by filtration, dissolved in DCM (50 mL), and washed with brine (2 × 20 mL). The organic layer was dried over anhydrous MgSO_4_ and was filtered through Celite. The solvent was evaporated under reduced pressure on a rotary evaporator. The crude product was adsorbed onto silica gel and purified by flash column chromatography, eluting with a gradient of ethyl acetate in petroleum ether, unless otherwise stated. Fractions containing the desired compound were evaporated to dryness to afford the target compounds.

Method B: Boc-protected compound was dissolved in a DCM:TFA mixture (15 mL, 2:1 *v*/*v*). The mixture was stirred at 25 °C for 1 h, and then the solvent was removed under reduced pressure (LCMS monitored). The crude product was purified by reverse phase chromatography (C18 OBD column, 5 μm silica, 19 mm diameter, 150 mm length), using decreasingly polar mixtures of Milli-Q water and MeOH as eluents. Fractions containing the desired compound were evaporated to dryness to afford pure product **6**.

#### 8-(6-Fluoropyridin-3-yl)-3-methyl-1-(tetrahydro-2H-pyran-4-yl)-1,3-dihydro-2H-imidazo­[4,5-*c*]­quinolin-2-one (**5**)

Synthesized from **4** (0.100 g, 0.28 mmol) and 6-fluoro-3-pyridinylboronic acid (0.058 g, 0.41 mmol), according to Method A to give **5** as a yellow solid (0.085 g, 82% yield); ^1^H NMR (300 MHz, CDCl_3_) δ 8.78 (s, 1H), 8.61 (s, 1H), 8.51 (s, 1H), 8.32 (d, *J* = 8.8 Hz, 1H), 8.30–8.14 (m, 1H), 7.85 (d, *J* = 8.8 Hz, 1H), 7.14 (dd, *J* = 8.5, 3.1 Hz, 1H), 5.17–5.09 (m, 1H), 4.30–4.24 (m, 2H), 3.67–3.58 (m, 5H), 3.04–2.80 (m, 2H), 2.01–1.94 (m, 2H). LC-MS: *t*_R_ = 0.763 min (Purity = 98%); *m*/*z* = 379.1 [M + H]^+^ (anal. calcd. for C_21_H_19_FN_4_O_2_: *m*/*z* = 378.1).

#### 3-Methyl-8-(6-(3-(methylamino)­propoxy)­pyridin-3-yl)-1-(tetrahydro-2H-pyran-4-yl)-1,3-dihydro-2H-imidazo­[4,5-*c*]­quinolin-2-one (**6**)

Following Method B, *tert*-butyl methyl­(3-((5-(3-methyl-2-oxo-1-(tetrahydro-2*H*-pyran-4-yl)-2,3-dihydro-1*H*-imidazo­[4,5-*c*]­quinolin-8-yl)­pyridin-2-yl)­oxy)­propyl)­carbamate (0.10 g; 0.18 mmol) in DCM:TFA (15 mL) afforded **6** as a white solid (0.05 g, 62% yield); ^1^H NMR (300 MHz, DMSO-*d*_*6*_) δ 9.14 (s, 1H), 8.72 (s, 1H), 8.66–8.39 (m, 2H), 8.52 (s, 1H), 8.27 (d, *J* = 8.8 Hz, 1H), 8.16 (d, *J* = 8.8 Hz, 1H), 7.05 (d, *J* = 8.9 Hz, 1H), 5.24–5.13 (m, 1H), 4.43 (t, *J* = 6.1 Hz, 2H), 4.09–4.06 (m, 2H), 3.64–3.55 (m, 5H), 2.74–2.71 (m, 2H), 2.62 (s, 3H), 2.54–2.48 (m, 2H), 2.10 (t, *J* = 7.2 Hz, 2H), 1.99–1.95 (m, 2H) ^13^C NMR (101 MHz, CDCl_3_) δ 163.57, 162.83, 162.48, 153.43, 145.16, 137.76, 136.85, 131.85, 129.16, 129.03, 128.66, 127.64, 123.75, 115.20, 111.82, 67.53 (2C), 62.91, 46.76, 33.14, 29.97 (2C), 29.72, 27.86, 25.93. LC-MS: *t*_R_ = 0.596 min (Purity = 98%); *m*/*z* = 448.2 [M + H]^+^ (anal. calcd. for C_25_H_29_N_5_O_3_: *m*/*z* = 447.2).

#### 8-(Pyridin-3-yl)-1-(tetrahydro-2H-pyran-4-yl)-1,3-dihydro-2H-imidazo­[4,5-*c*]­quinolin-2-one (**7**)

Synthesized from 8-bromo-1-(tetrahydro-2*H*-pyran-4-yl)-1,3-dihydro-2*H*-imidazo­[4,5-*c*]­quinolin-2-one **3** (0.120 g, 0.34 mmol) and 3-(4,4,5,5-tetramethyl-1,3,2-dioxaborolan-2-yl)­pyridine (0.106 g, 0.52 mmol) according to Method A to give **7** as a beige solid (0.023 g, 19% yield); ^1^H NMR (300 MHz, DMSO-*d*_6_) δ 11.59 (s, 1H), 9.09 (s, 1H), 8.72–8.61 (m, 2H), 8.51 (s, 1H), 8.27 (d, *J* = 8.0 Hz, 1H), 8.15 (d, *J* = 8.8 Hz, 1H), 8.00 (d, *J* = 8.8 Hz, 1H), 7.87–7.16 (m, 1H), 5.11 (s, 1H), 4.12–3.99 (m, 2H), 3.58 (t, *J* = 11.9 Hz, 2H), 2.83–2.65 (m, 2H), 2.02–1.88 (m, 2H). LC-MS: *t*_R_ = 0.536 min (Purity = 100%); *m*/*z* = 347.1 [M + H]^+^ (anal. calcd. for C_20_H_18_N_4_O_2_: *m*/*z* = 346.1).

#### 3-Methyl-8-(pyridin-3-yl)-1-(tetrahydro-2H-pyran-4-yl)-1,3-dihydro-2H-imidazo­[4,5-*c*]­quinolin-2-one (**8**)

Synthesized from **4** (0.125 g, 0.34 mmol) and 3-(4,4,5,5-tetramethyl-1,3,2-dioxaborolan-2-yl)­pyridine (0.106 g, 0.52 mmol) according to Method A to afford **8** as a beige solid (0.056g, 45% yield); ^1^H NMR (300 MHz, DMSO-*d*_6_) δ 9.09 (s, 1H), 8.92 (s, 1H), 8.69–8.61 (m, 1H), 8.51 (s, 1H), 8.27 (d, *J* = 8.0 Hz, 1H), 8.17 (d, *J* = 8.8 Hz, 1H), 8.00 (d, *J* = 8.9 Hz, 1H), 7.59 (t, *J* = 6.3 Hz, 1H), 5.17 (d, *J* = 12.6 Hz, 1H), 4.12–4.01 (m, 2H), 3.66–3.53 (m, 2H), 3.52 (s, 3H), 2.86–2.62 (m, 2H), 1.93 (d, *J* = 12.8 Hz, 2H). LC-MS: *t*_R_ = 0.590 min (Purity = 100%); *m*/*z* = 361.2 [M + H]^+^ (anal. calcd. for C_21_H_20_N_4_O_2_: *m*/*z* = 360.2).

#### 8-(6-Methoxypyridin-3-yl)-3-methyl-1-(tetrahydro-2H-pyran-4-yl)-1,3-dihydro-2H-imidazo­[4,5-*c*]­quinolin-2-one (**9**)

Synthesized from **4** (0.100 g, 0.28 mmol) and 2-methoxypyridine-5-boronic acid (0.063 g, 0.41 mmol) according to Method A to give **9** as a white solid (0.057 g, 53% yield); ^1^H NMR (600 MHz, DMSO-*d*_*6*_) δ 8.88 (s, 1H), 8.67 (d, *J* = 2.6 Hz, 1H), 8.43 (m, 1H), 8.18 (dd, *J* = 8.6, 2.6 Hz, 1H), 8.14 (d, *J* = 8.8 Hz, 1H), 7.96–7.92 (m, 1H), 7.00 (d, *J* = 8.7 Hz, 1H), 5.18–5.09 (m, 1H), 4.08 (dd, *J* = 11.6, 4.6 Hz, 2H), 3.96 (s, 3H), 3.63–3.56 (m, 2H), 3.52 (s, 3H), 2.74 (m, 2H), 1.96–1.90 (m, 2H). ^13^C NMR (151 MHz, DMSO-*d*_*6*_) δ 163.26, 152.76, 145.04, 143.78, 137.67, 134.52, 133.06, 131.25, 128.78, 127.87, 125.04, 123.13, 117.99, 115.14, 110.64, 66.53 (2C), 53.19, 52.67, 29.62 (2C), 26.98. LC-MS: *t*_R_ = 0.787 min (Purity = 97%); *m*/*z* = 391.2 [M + H]^+^ (anal. calcd. for C_22_H_22_N_4_O_3_: *m*/*z* = 390.2).

#### 8-(2-Fluoropyridin-3-yl)-3-methyl-1-(tetrahydro-2H-pyran-4-yl)-1,3-dihydro-2H-imidazo­[4,5-*c*]­quinolin-2-one (**10**)

Synthesized from **4** (0.100 g, 0.28 mmol) and 2-fluoropyridine-3-boronic acid (0.059 g, 0.41 mmol) according to Method A to give **10** as a white solid (0.077 g, 74% yield); ^1^H NMR (600 MHz, DMSO-*d*_*6*_) δ 8.93 (s, 1H), 8.57 (m, 1H), 8.35 (m, 1H), 8.31 (m, 1H), 8.18 (d, *J* = 8.8 Hz, 1H), 7.89 (m, 1H), 7.57 (m, 1H), 5.08 (m, 1H), 4.05 (m, 2H), 3.59–3.48 (m, 5H), 2.72 (m, 2H), 1.94–1.88 (m, 2H). ^13^C NMR (151 MHz, DMSO-*d*_*6*_) δ 159.67 (d, *J* = 237.2 Hz), 152.74, 146.56 (d, *J* = 15.2 Hz), 143.91, 141.34 (d, *J* = 3.8 Hz), 133.72, 130.90, 130.67 (d, *J* = 5.3 Hz), 128.05, 126.47, 123.18, 122.69 (d, *J* = 4.1 Hz), 122.45, 122.27, 114.71, 66.45 (2C), 52.97, 29.58 (2C), 26.99. LC-MS: *t*_R_ = 0.750 min (Purity = 96%); *m*/*z* = 379.2 [M + H]^+^ (anal. calcd. for C_21_H_19_FN_4_O_2_: *m*/*z* = 378.1).

#### 8-(2-Fluorophenyl)-3-methyl-1-(tetrahydro-2H-pyran-4-yl)-1,3-dihydro-2H-imidazo­[4,5-*c*]­quinolin-2-one (**11**)

Synthesized from **4** (0.100 g, 0.28 mmol) and 2-fluorophenylboronic acid (0.058g, 0.41 mmol) according to Method A to give **11** as a white solid (0.085 g, 82% yield); ^1^H NMR (600 MHz, DMSO-*d*_*6*_) δ 8.92 (s, 1H), 8.51 (m, 1H), 8.15 (d, *J* = 8.8 Hz, 1H), 7.87–7.82 (m, 1H), 7.81–7.75 (m, 1H), 7.67–7.59 (m, 1H), 7.59–7.47 (m, 2H), 7.42–7.37 (m, 2H), 5.06 (m, 1H), 4.05 (m, 2H), 3.55–3.48 (m, 5H), 2.76–2.66 (m, 2H), 1.90 (m, 2H). ^13^C NMR (151 MHz, DMSO-*d*_*6*_) δ 159.41 (d, *J* = 246.5 Hz), 152.82, 143.82, 133.63, 132.44, 131.97, 131.43 (d, *J* = 9.5 Hz), 130.00 (d, *J* = 8.6 Hz), 128.69 (d, *J* = 11.6 Hz), 128.07, 127.55 (d, *J* = 12.3 Hz), 127.01, 126.99, 125.22, 123.17, 116.40 (d, *J* = 22.6 Hz), 114.86, 66.64 (2C), 53.67, 29.74 (2C), 27.13. LC-MS: *t*_R_ = 0.853 min (Purity = 100%); *m*/*z* = 378.2 [M + H]^+^ (anal. calcd. for C_22_H_20_FN_3_O_2_: *m*/*z* = 377.2).

#### *N*-Methyl-4-(3-methyl-2-oxo-1-(tetrahydro-2H-pyran-4-yl)-2,3-dihydro-1H-imidazo­[4,5-*c*]­quinolin-8-yl)­benzamide (**12**)

Synthesized from **4** (0.100 g, 0.28 mmol) and (4-(methylcarbamoyl)­phenyl)­boronic acid (0.074 g, 0.41 mmol) according to Method A to give **12** as a white solid (0.083 g, 73% yield); ^1^H NMR (300 MHz, DMSO-*d*_*6*_) δ 8.92 (s, 1H), 8.58–8.49 (m, 2H), 8.17 (d, *J* = 8.9 Hz, 1H), 8.07–7.89 (m, 5H), 5.28–5.04 (m, 1H), 4.12–4.05 (m, 2H), 3.67–3.47 (m, 5H), 2.83 (s, 3H), 2.82–2.64 (m, 2H), 1.97–1.92 (m, 2H). ^13^C NMR (151 MHz, DMSO-*d*_*6*_) δ: 166.73, 153.47, 142.51, 137.34, 134.40, 133.90, 131.84, 128.41 (2C), 128.19, 127.33 (2C), 127.08, 125.91, 123.88, 119.74, 115.73, 67.26 (2C), 40.76, 30.34 (2C), 27.66, 26.67. LC-MS: *t*_R_ = 0.710 min (Purity = 97%); *m*/*z* = 417.2 [M + H]^+^ (anal. calcd. for C_24_H_24_N_4_O_3_: *m*/*z* = 416.2).

#### 4-(3-Methyl-2-oxo-1-(tetrahydro-2H-pyran-4-yl)-2,3-dihydro-1H-imidazo­[4,5-*c*]­quinolin-8-yl)­benzamide (**13**)

Synthesized from **4** (0.100 g, 0.28 mmol) and (4-carbamoylphenyl)­boronic acid (0.068 g, 0.41 mmol) according to Method A to give **13** as a white solid (0.077 g, 70% yield); ^1^H NMR (300 MHz, DMSO-*d*_*6*_) δ 8.93 (s, 1H), 8.54 (s, 1H), 8.17 (d, *J* = 8.9 Hz, 1H), 8.11–8.02 (m, 3H), 7.96 (d, *J* = 8.4 Hz, 2H), 7.48 (br.s 2H), 5.30–5.00 (m, 1H), 4.11–4.06 (m, 2H), 3.65–3.49 (m, 5H), 2.80–2.66 (m, 2H), 2.02–1.89 (m, 2H). ^13^C NMR (151 MHz, DMSO-*d*_*6*_) δ: 167.98, 153.47, 142.29, 137.37, 134.21, 133.95, 131.89, 128.84 (2C), 128.62 (2C), 127.29 (2C), 127.06 (2C), 125.92, 67.27 (2C), 30.35 (2C), 27.65 (2C). LC-MS: *t*_R_ = 0.675 min (Purity = 98%); *m*/*z* = 403.2 [M + H]^+^ (anal. calcd. for C_23_H_22_N_4_O_3_; *m*/*z* = 402.2).

#### 3-Methyl-8-(4-(methylsulfonyl)­phenyl)-1-(tetrahydro-2H-pyran-4-yl)-1,3-dihydro-2H-imidazo­[4,5-*c*]­quinolin-2-one (**14**)

Synthesized from **4** (0.100 g, 0.28 mmol) and (4-(methylsulfonyl)­phenyl)­boronic acid (0.082 g, 0.41 mmol) according to Method A to give compound **14** as a yellow solid (0.082 g, 73% yield); ^1^H NMR (300 MHz, DMSO-*d*_*6*_) δ 8.95 (s, 1H), 8.56 (s, 1H), 8.24–7.99 (m, 6H), 5.32–4.99 (m, 1H), 4.10–4.05 (m, 2H), 3.55–3.61 (m, 2H), 3.53 (s, 3H), 3.32 (s, 3H), 2.83–2.63 (m, 2H), 1.96–1.92 (m, 2H). ^13^C NMR (101 MHz, DMSO-*d*_*6*_) δ: 154.30, 147.39, 142.69, 142.12, 137.99, 136.01, 130.96, 128.68, 129.00 (2C), 127.02, 126.83, 124.98 (2C), 121.19, 116.01, 67.19 (2C), 44.52, 30.11 (2C), 28.14 (2C). LC-MS: *t*_R_ = 0.744 min (Purity = 97%); *m*/*z* = 438.1 [M + H]^+^ (anal. calcd. for C_23_H_23_N_3_O_4_S: *m*/*z* = 437.1).

#### 3-Methyl-8-(3-(methylsulfonyl)­phenyl)-1-(tetrahydro-2H-pyran-4-yl)-1,3-dihydro-2H-imidazo­[4,5-*c*]­quinolin-2-one (**15**)

Synthesized from **4** (0.100 g, 0.28 mmol) and (3-(methylsulfonyl)­phenyl)­boronic acid (0.082 g, 0.41 mmol) according to Method A to give **15** as a yellow solid (0.080 g, 72% yield); ^1^H NMR (300 MHz, DMSO-*d*_*6*_) δ 8.92 (s, 1H), 8.56 (s, 1H), 8.38 (s, 1H), 8.28–8.13 (m, 2H), 8.10–7.95 (m, 2H), 7.84 (m, 1H), 5.21–5.15 (m, 1H), 4.10–4.04 (m, 2H), 3.67–3.54 (m, 2H), 3.51 (s, 3H), 3.35 (s, 3H), 2.79–2.65 (m, 2H), 2.02–1.88 (m, 2H). ^13^C NMR (101 MHz, DMSO-*d*_*6*_) δ: 153.36, 144.75, 142.43, 141.24, 136.33, 134.29, 132.50, 132.04, 130.85, 128.71, 126.60, 126.04, 125.80, 123.84, 120.35, 115.65, 67.14 (2C), 43.96, 30.25 (2C), 27.71 (2C). LC-MS: *t*_R_ = 0.751 min (Purity = 97%); *m*/*z* = 438.1 [M + H]^+^ (anal. calcd. for C_23_H_23_N_3_O_4_S: *m*/*z* = 437.1).

#### 3-Methyl-8-(5-(methylsulfonyl)­pyridin-3-yl)-1-(tetrahydro-2H-pyran-4-yl)-1,3-dihydro-2H-imidazo­[4,5-*c*]­quinolin-2-one (**16**)

Synthesized from **4** (0.100 g, 0.28 mmol) and (5-(methylsulfonyl)­pyridin-3-yl)­boronic acid (0.041 g; 0.21 mmol) according to Method A to give **16** as a white solid (0.04 g, 66%); ^1^H NMR (300 MHz, DMSO-*d*_*6*_) δ 9.45 (s, 1H), 9.14 (d, *J* = 10.1 Hz, 1H), 8.97 (s, 1H), 8.82 (s, 1H), 8.75 (s, 1H), 8.63 (s, 1H), 8.19 (d, *J* = 10.5 Hz, 1H), 5.21–5.15 (m, 1H), 4.07–4.03 (m, 2H), 3.62–3.53 (m, 5H), 3.46 (s, 3H), 2.79–2.67 (m, 2H), 1.97–1.93 (m, 2H). ^13^C NMR (151 MHz, DMSO-*d*_*6*_) δ 153.32, 152.81, 147.99, 146.92, 144.84, 137.87, 136.05, 134.55, 134.52, 133.94, 133.30, 132.12, 128.72, 125.73, 123.88, 67.03, 66.80, 44.08 (2C), 30.14 (2C), 27.69. LC-MS: *t*_R_ = 0.657 min (Purity = 97%); *m*/*z* = 439.1 [M + H]^+^ (anal. calcd. for C_22_H_22_N_4_O_4_S: *m*/*z* = 438.1).

#### 3-Methyl-8-(3-(methylsulfinyl)­phenyl)-1-(tetrahydro-2H-pyran-4-yl)-1,3-dihydro-2H-imidazo­[4,5-*c*]­quinolin-2-one (**17**)

Synthesized from **4** (0.100 g, 0.28 mmol) and (3-(methylsulfinyl)­phenyl)­boronic acid (0.076 g, 0.41 mmol) according to Method A to give **17** as a yellow solid (0.081 g, 70% yield); ^1^H NMR (300 MHz, DMSO-*d*_*6*_) δ 8.90 (s, 1H), 8.54 (s, 1H), 8.17–8.14 (m, 2H), 8.07–7.97 (m, 2H), 7.72–7.77 (m, 2H), 5.19–5.11 (m, 1H), 4.11–4.04 (m, 2H), 3.71–3.45 (m, 5H), 2.86 (s, 3H), 2.86–2.67 (m, 2H), 1.95–1.91 (m, 2H). ^13^C NMR (101 MHz, DMSO-*d*_*6*_) δ: 153.35, 148.14, 144.67, 141.07, 136.99, 134.10, 131.93, 130.59, 129.68, 128.66, 125.85, 123.77, 123.45, 122.47, 119.97, 115.65, 67.18 (2C), 43.78, 30.26 (2C), 27.69 (2C). LC-MS: *t*_R_ = 0.706 min (Purity = 96%); *m*/*z* = 422.1 [M + H]^+^ (anal. calcd. for C_23_H_23_N_3_O_3_S: *m*/*z* = 421.1).

#### 3-Methyl-8-(2-methylpyridin-3-yl)-1-(tetrahydro-2H-pyran-4-yl)-1,3-dihydro-2H-imidazo­[4,5-*c*]­quinolin-2-one (**18**)

Synthesized from **4** (0.100 g, 0.28 mmol) and 2-methylpyridine-3-boronic acid (0.057 g, 0.41 mmol) according to Method A to give **18** as a white solid (0.056 g, 54% yield); ^1^H NMR (600 MHz, DMSO-*d*_*6*_) δ 8.93 (s, 1H), 8.54 (dd, *J* = 4.8, 1.8 Hz, 1H), 8.28 (d, *J* = 1.9 Hz, 1H), 8.15 (d, *J* = 8.7 Hz, 1H), 7.82 (dd, *J* = 7.7, 1.8 Hz, 1H), 7.70 (dd, *J* = 8.7, 1.8 Hz, 1H), 7.39 (dd, *J* = 7.6, 4.8 Hz, 1H), 5.04 (tt, *J* = 11.9, 4.4 Hz, 1H), 4.01 (dd, *J* = 11.6, 4.7 Hz, 2H), 3.53 (s, 3H), 3.49 (td, *J* = 12.1, 2.0 Hz, 2H), 2.76–2.66 (m, 2H), 2.57 (s, 3H), 1.92–1.85 (m, 2H). ^13^C NMR (151 MHz, DMSO-*d*_*6*_) δ 154.92, 152.78, 147.89, 143.65, 137.17, 137.01, 135.63, 133.36, 130.61, 127.91, 127.55, 123.18, 121.36, 121.07, 114.75, 66.43 (2C), 52.80, 29.44 (2C), 26.99, 23.17. LC-MS: *t*_R_ = 0.450 min (Purity = 96%); *m*/*z* = 375.2 [M + H]^+^ (anal. calcd. for C_22_H_22_N_4_O_2_: *m*/*z* = 374.2).

#### 3-Methyl-8-(4-methylpyridin-3-yl)-1-(tetrahydro-2H-pyran-4-yl)-1,3-dihydro-2H-imidazo­[4,5-*c*]­quinolin-2-one (**19**)

Synthesized from **4** (0.100 g, 0.28 mmol) and 4-methylpyridine-3-boronic acid (0.057 g, 0.41 mmol) according to Method A to give **19** as a white solid (0.027 g, 27% yield); ^1^H NMR (600 MHz, DMSO-*d*_*6*_) δ 8.95 (s, 1H), 8.60 (s, 1H), 8.50 (d, *J* = 5.0 Hz, 1H), 8.29 (d, *J* = 1.9 Hz, 1H), 8.16 (d, *J* = 8.7 Hz, 1H), 7.71 (dd, *J* = 8.7, 1.8 Hz, 1H), 7.42 (d, *J* = 5.0 Hz, 1H), 5.05 (m, 1H), 4.00 (m, 2H), 3.53 (s, 3H), 3.49 (m, 2H), 2.70 (m, 2H), 2.41 (s, 3H), 1.90–1.84 (m, 2H). ^13^C NMR (151 MHz, DMSO-*d*_*6*_) δ: 152.86, 149.72, 148.39, 144.17, 143.79, 136.57, 135.10, 133.61, 130.74, 127.99, 127.89, 125.53, 123.24, 121.47, 114.90, 66.53 (2C), 53.67, 29.53 (2C), 27.15, 19.54. LC-MS: *t*_R_ = 0.533 min (Purity = 100%); *m*/*z* = 375.2 [M + H]^+^ (anal. calcd. for C_22_H_22_N_4_O_2_: *m*/*z* = 374.2).

#### 8-(6-Amino-2-methylpyridin-3-yl)-3-methyl-1-(tetrahydro-2H-pyran-4-yl)-1,3-dihydro-2H-imidazo­[4,5-*c*]­quinolin-2-one (**20**)

8-(6-Fluoro-2-methylpyridin-3-yl)-3-methyl-1-(oxan-4-yl)­imidazo­[4,5-*c*]­quinolin-2-one (0.127 g, 32 mmol) obtained from **4** (0.148 g, 0.40 mmol) and (6-fluoro-2-methylpyridin-3-yl)­boronic acid (0.076 g, 0.49 mmol) according to Method A was heated (120 °C) with sodium carbonate (0.173 g, 1.63 mmol) and tert-Butylamine (2 mL, 19 mmol) in DMSO (1.5 mL) for 7 days. After cooling, the reaction was diluted with water (5 mL) to precipitate the crude product to give a 0.100 g beige solid (50% conversion to the desired product 8-[6-(tert-butylamino)-2-methylpyridin-3-yl]-3-methyl-1-(oxan-4-yl)­imidazo­[4,5-*c*]­quinolin-2-one). The crude product was refluxed in TFA (3 mL, 39 mmol) at 80 °C for 16 h. Purification was performed using silica gel flash chromatography eluting a gradient of ethyl acetate in petroleum ether to give **20** as a white solid (0.021 mg, 45% yield); ^1^H NMR (300 MHz, DMSO-*d*_6_) δ 8.88 (s, 1H), 8.16 (s, 1H), 8.07 (d, *J* = 8.7 Hz, 1H), 7.63 (d, *J* = 8.8 Hz, 1H), 7.47 (d, *J* = 8.4 Hz, 1H), 6.44 (d, *J* = 8.4 Hz, 1H), 6.08 (s, 2H), 5.13–4.93 (m, 1H), 4.10–3.96 (m, 2H), 3.56–3.42 (m, 5H), 2.79–2.58 (m, 2H), 2.39 (s, 3H), 1.91–1.78 (m, 2H). ^13^C NMR (151 MHz, DMSO) δ: 158.47, 152.88, 143.30, 139.01, 138.24, 132.93, 130.43, 128.25, 127.78, 123.60, 123.11, 120.44, 114.93, 105.87, 66.67 (2C), 52.89, 29.59 (2C), 27.14, 22.98. LC-MS: *t*_R_ = 0.279 min (Purity = 100%); *m*/*z* = 390.2 [M + H]^+^ (anal. calcd. for C_22_H_23_N_5_O_2_: *m*/*z* = 389.2).

#### 8-(6-Aminopyridin-3-yl)-3-methyl-1-(tetrahydro-2H-pyran-4-yl)-1,3-dihydro-2H-imidazo­[4,5-*c*]­quinolin-2-one (**21**)

Synthesized from **4** (0.250 g, 0.69 mmol) and 2-aminopyridine-5-boronic acid, pinacol ester (0.182 g, 0.83 mmol) according to Method A to give **21** as a beige solid (0.025 g, 9% yield); ^1^H NMR (300 MHz, DMSO-*d*_6_) δ 8.84 (s, 1H), 8.45 (s, 1H), 8.33 (s, 1H), 8.07 (d, *J* = 8.9 Hz, 1H), 7.89 (d, *J* = 8.8 Hz, 2H), 6.60 (d, *J* = 8.6 Hz, 1H), 6.24 (s, 2H), 5.11 (s, 1H), 4.14–4.02 (m, 2H), 3.64–3.54 (m, 2H), 3.50 (s, 3H), 2.80–2.62 (m, 2H), 1.91 (d, *J* = 12.3 Hz, 2H). ^13^C NMR (101 MHz, DMSO-*d*_6_) δ 159.52, 152.90, 146.40, 143.56, 135.88, 135.66, 132.76, 131.24, 127.81, 124.81, 123.33, 123.16, 116.53, 115.42, 108.18, 66.76 (2C), 52.76, 29.83 (2C), 27.18. LC-MS: *t*_R_ = 0.524 min (Purity = 100%); *m*/*z* = 376.2 [M + H]^+^ (anal. calcd. for C_21_H_21_N_5_O_2_: *m*/*z* = 375.2).

#### N-(5-(3-Methyl-2-oxo-1-(tetrahydro-2H-pyran-4-yl)-2,3-dihydro-1H-imidazo­[4,5-*c*]­quinolin-8-yl)­pyridin-2-yl)­acetamide (**22**)

Synthesized from **4** (0.150 g, 0.41 mmol) and (6-acetamidopyridin-3-yl)­boronic acid (0.089 g, 0.49 mmol) according to Method A to give **22** as a beige solid (0.052 g, 28% yield); ^1^H NMR (300 MHz, DMSO-*d*_6_) δ 10.70 (s, 1H), 8.90 (s, 1H), 8.83 (d, *J* = 2.0 Hz, 1H), 8.49 (s, 1H), 8.32–8.22 (m, 2H), 8.15 (d, *J* = 8.8 Hz, 1H), 8.01 (d, *J* = 8.8 Hz, 1H), 5.23–5.08 (m, 1H), 4.13–4.02 (m, 2H), 3.60 (t, *J* = 11.7 Hz, 2H), 3.52 (s, 3H), 2.72 (td, *J* = 12.7, 8.0 Hz, 2H), 2.14 (s, 3H), 1.98–1.88 (m, 2H). ^13^C NMR (101 MHz, DMSO-*d*_6_) δ: 169.42, 152.90, 151.73, 146.22, 143.99, 136.56, 134.45, 133.47, 131.47, 130.49, 128.07, 125.11, 123.30, 118.60, 115.28, 113.31, 66.68 (2C), 29.76 (2C), 27.24, 23.97. LC-MS: *t*_R_ = 0.743 min (Purity = 96%); *m*/*z* = 418.2 [M + H]^+^ (anal. calcd. for C_23_H_23_N_5_O_3_: *m*/*z* = 417.2).

#### 8-(2-Aminopyridin-4-yl)-3-methyl-1-(tetrahydro-2H-pyran-4-yl)-1,3-dihydro-2H-imidazo­[4,5-*c*]­quinolin-2-one (**23**)

tert-Butyl (4-(3-methyl-2-oxo-1-(tetrahydro-2H-pyran-4-yl)-2,3-dihydro-1H-imidazo­[4,5-*c*]­quinolin-8-yl)­pyridin-2-yl)­carbamate (0.209 g, 0.44 mmol) crude product was obtained from **4** (0.200 g, 0.55 mmol) and Boc-2-aminopyridine-4-boronic acid pinacol ester (0.212 g, 0.66 mmol) according to Method A. Following Method B, in DCM:TFA (5 mL) gave **23** as a beige solid (0.123 g, 59% yield); ^1^H NMR (300 MHz, DMSO-*d*_6_) δ 8.91 (s, 1H), 8.46 (s, 1H), 8.21–8.01 (m, 2H), 7.88 (d, *J* = 8.9 Hz, 1H), 6.95 (d, *J* = 5.3 Hz, 1H), 6.84 (s, 1H), 6.14 (s, 2H), 5.25–4.94 (m, 1H), 4.13–4.03 (m, 2H), 3.67–3.40 (m, 5H), 2.80–2.62 (m, 2H), 2.01–1.83 (m, 2H). 13C NMR (101 MHz, DMSO-*d*_6_) δ: 160.62, 152.88, 148.73, 147.82, 144.51, 135.98, 133.75, 131.45, 128.28, 124.93, 123.27, 119.05, 115.11, 110.23, 105.52, 66.77 (2C), 53.16, 29.82 (2C), 27.22. LC-MS: *t*_R_ = 0.562 min (Purity = 100%); *m*/*z* = 376.2 [M + H]^+^ (anal. calcd. for C_21_H_21_N_5_O_2_: *m*/*z* = 375.2).

#### 3-Methyl-8-(1H-pyrrolo­[2,3-*b*]­pyridin-4-yl)-1-(tetrahydro-2H-pyran-4-yl)-1,3-dihydro-2H-imidazo­[4,5-*c*]­quinolin-2-one (**24**)

Synthesized from **4** (0.150 g, 0.41 mmol) and 1H-pyrrolo­[2,3-*b*]­pyridine, 4-(4,4,5,5-tetramethyl-1,3,2-dioxaborolan-2-yl) (0.114 g, 0.47 mmol) according to Method A to give **24** as a yellow solid (0.133 g, 80% yield); ^1^H NMR (300 MHz, DMSO-*d*_6_) δ 11.96 (s, 1H), 8.93 (s, 1H), 8.68 (s, 1H), 8.37 (d, *J* = 4.9 Hz, 1H), 8.22 (d, *J* = 8.7 Hz, 1H), 8.02 (d, *J* = 8.8 Hz, 1H), 7.72–7.64 (m, 1H), 7.37 (d, *J* = 4.9 Hz, 1H), 6.86–6.79 (m, 1H), 5.18–4.97 (m, 1H), 4.01 (dd, *J* = 11.8, 4.3 Hz, 2H), 3.54–3.40 (m, 5H), 2.82–2.64 (m, 2H), 2.00–1.88 (m, 2H). ^13^C NMR (151 MHz, DMSO- *d*_6_) δ: 152.78, 149.29, 144.27, 143.05, 139.61, 135.89, 133.64, 131.41, 128.19, 127.10, 126.41, 123.19, 120.70, 117.28, 115.15, 114.56, 98.87, 66.78 (2C), 53.62, 29.67 (2C), 27.12. LC-MS: *t*_R_ = 0.647 min (Purity = 100%); *m*/*z* = 400.2 [M + H]^+^ (anal. calcd. for C_23_H_21_N_5_O_2_: *m*/*z* = 399.2).

#### 3-Methyl-8-(3-(methylsulfonyl)­phenyl)-1-(piperidin-4-yl)-1,3-dihydro-2H-imidazo­[4,5-*c*]­quinolin-2-one (**25**)

Following Method B, *tert*-butyl 4-(3-methyl-8-(3-(methylsulfonyl)­phenyl)-2-oxo-2,3-dihydro-1*H*-imidazo­[4,5-*c*]­quinolin-1-yl)­piperidine-1-carboxylate (0.05 g, 0.09 mmol) in DCM:TFA (15 mL) gave **25** as a white solid (0.038 g; 93% yield); ^1^H NMR (300 MHz, DMSO-*d*_*6*_) δ 9.08 (s, 1H), 9.02 (s, 1H), 8.56 (s, 1H), 8.43 (s, 1H), 8.35–8.23 (m, 2H), 8.18 (d, *J* = 7.7 Hz, 1H), 8.02 (d, *J* = 7.7 Hz, 1H), 7.85 (t, *J* = 7.8 Hz, 1H), 5.31–5.30 (m, 1H), 3.54 (s, 3H), 3.48–3.44 (m, 2H), 3.35 (s, 3H), 3.34–3.12 (m, 2H), 3.00–2.87 (m, 2H), 2.29–2.25 (m, 2H). ^13^C NMR (101 MHz, DMSO-*d*_*6*_) δ: 153.27, 142.25, 141.01, 136.90, 132.90 (3C), 130.72 (3C), 126.60 (3C), 123.87, 120.12, 115.48, 52.69, 43.97 (2C), 43.46 (2C), 127.86, 26.55. LC-MS: *t*_R_ = 0.506 min (Purity = 98%); *m*/*z* = 437.1 [M + H]^+^ (anal. calcd. for C_23_H_24_N_4_O_3_S: *m*/*z* = 436.2).

#### 1-(3-Fluoropiperidin-4-yl)-3-methyl-8-(3-(methylsulfonyl)­phenyl)-1,3-dihydro-2H-imidazo­[4,5-*c*]­quinolin-2-one (**26**)

Following Method B, *tert-*butyl 3-fluoro-4-(3-methyl-8-(3-(methylsulfonyl)­phenyl)-2-oxo-2,3-dihydro-1*H*-imidazo­[4,5-*c*]­quinolin-1-yl)­piperidine-1-carboxylate (0.05 g, 0.09 mmol) in DCM:TFA (15 mL) gave a mixture of diasteroisomers **26** as a white solid (0.034 g; 83%); ^1^H NMR (300 MHz, DMSO-*d*_*6*_) δ 9.02 (s, 1H), 8.96–8.95 (m, 2H), 8.84 (s, 1H), 8.52 (m, 2H), 8.45–7.95 (m, 10H), 7.85 (m, 2H), 5.92–4.96 (m, 4H), 3.59–3.53 (m, 6H), 3.34 (s, 6H), 3.21–3.13 (m, 2H), 3.04–2.89 (m, 2H), 2.97–2.66 (m, 2H), 2.63–2.58 (m, 2H), 2.14–2.10 (m, 2H), 1.91–1.87 (m, 2H). ^13^C NMR (151 MHz, DMSO-*d*_*6*_) δ 153.75, 153.12, 144.77, 144.69, 142.35, 142.30, 141.33, 141.23, 136.28, 134.29, 134.26, 132.62, 132.56, 132.47, 131.94, 131.90, 130.79, 130.74, 129.30, 128.87, 126.54, 126.48, 126.09, 126.04, 125.94, 125.83, 125.78, 123.97, 123.76, 119.62, 115.80, 115.67, 89.70, 88.97, 88.52, 87.79, 60.32, 60.21, 50.18, 50.03, 45.02, 44.89, 43.97, 30.23, 27.78, 27.63. LC-MS: *t*_R_ = 0.508 and 0.556 min (Purity = 98%); *m*/*z* = 455.1 [M + H]^+^ (anal. calcd. for C_23_H_23_FN_4_O_3_S: *m*/*z* = 454.1).

#### 3-Methyl-1-(3-methylpiperidin-4-yl)-8-(3-(methylsulfonyl)­phenyl)-1,3-dihydro-2H-imidazo­[4,5-*c*]­quinolin-2-one (**27**)

Following Method B, *tert-*butyl 3-methyl-4-(3-methyl-8-(3-(methylsulfonyl)­phenyl)-2-oxo-2,3-dihydro-1*H*-imidazo­[4,5-*c*]­quinolin-1-yl)­piperidine-1-carboxylate (0.05 g, 0.09 mmol) in DCM:TFA (15 mL) gave a mixture of diasteroisomers **27** as a white solid (0.037 g; 90% yield); ^1^H NMR (300 MHz, DMSO-*d*_*6*_) δ 9.00–8.99 (m, 2H), 8.96 (s, 1H), 8.66 (s, 1H), 8.58 (s, 1H), 8.43–8.18 (m, 7H), 8.14–7.97 (m, 4H), 7.88–7.81­(m, 2H), 5.45–5.41 (m, 1H), 4.96–4.88 (m, 1H), 3.55 (s, 3H), 3.52 (s, 3H), 3.34–3.30 (m, 15H), 3.03–2.99 (m, 1H), 2.90–2.87 (m, 1H), 2.77–2.73 (m, 1H), 2.39–2.28 (m, 1H), 2.09–2.04 (m, 1H), 1.16 (d, *J* = 7.0 Hz, 3H), 0.84 (d, *J* = 6.4 Hz, 3H). ^13^C NMR (151 MHz, DMSO-*d*_*6*_) δ: 153.99, 153.07, 144.62, 144.56, 142.36, 142.31, 141.41, 141.24, 136.94, 136.69, 134.17, 134.04, 132.77, 132.74, 131.88, 131.82, 130.67 (2C), 129.37, 128.99, 126.52, 126.46 (2C), 126.33, 126.23, 126.11, 123.96, 123.91, 119.75, 119.58, 115.72, 115.66, 58.70, 55.27, 48.90, 48.12, 44.11, 44.07, 43.80, 43.51, 32.24, 30.61, 27.75, 27.71, 26.75, 22.24, 15.15, 12.10. LC-MS: *t*_R_ = 0.537 and 0.554 min (Purity = 98%);. *m*/*z* = 451.2 [M + H]^+^ (anal. for C_24_H_26_N_4_O_3_S: *m*/*z* = 450.2)

#### 1-(Azetidin-3-yl)-3-methyl-8-(3-(methylsulfonyl)­phenyl)-1,3-dihydro-2H-imidazo­[4,5-*c*]­quinolin-2-one (**28**)

Following Method B, *tert*-butyl 3-(3-methyl-8-(3-(methylsulfonyl)­phenyl)-2-oxo-2,3-dihydro-1*H*-imidazo­[4,5-*c*]­quinolin-1-yl)­azetidine-1-carboxylate (0.05 g; 0.10 mmol) in DCM:TFA (15 mL) gave **28** as a white solid (0.035 g; 87% yield); ^1^H NMR (300 MHz, DMSO-*d*_*6*_) δ 9.03 (m, 2H), 8.50 (s, 1H), 8.38–8.28 (m, 2H), 8.22 (d, *J* = 8.8 Hz, 1H), 8.13–7.98 (m, 2H), 7.86 (t, *J* = 7.7 Hz, 1H), 6.23–6.12 (m, 1H), 4.88–4.85 (m, 2H), 4.48–4.42 (m, 2H), 3.60 (s, 3H), 3.37 (s, 3H). ^13^C NMR (151 MHz, DMSO-*d*_*6*_) δ 153.81, 144.38, 142.37, 141.47, 136.98, 134.17, 133.05, 131.52, 130.59, 128.80, 126.75, 126.52, 126.18, 123.82, 119.62, 115.67, 50.85 (2C), 47.48, 44.01, 27.79. LC-MS: *t*_R_ = 0.452 min (Purity = 98%); *m*/*z* = 409.1 [M + H]^+^ (anal. calcd. for C_21_H_20_N_4_O_3_S: *m*/*z* = 408.1).

#### 3-Methyl-8-(2-methylpyridin-3-yl)-1-phenyl-1,3-dihydro-2H-imidazo­[4,5-*c*]­quinolin-2-one (**29**)

Synthesized from **4b** (0.150 g, 0.42 mmol) and 2-methylpyridine-3-boronic acid pinacol ester (0.111 g, 0.51 mmol) according to Method A to give **29** as a white solid (0.116 g, 74% yield); ^1^H NMR (300 MHz, DMSO-*d*_6_) δ 9.05 (s, 1H), 8.42 (dd, *J* = 4.6, 1.8 Hz, 1H), 8.12 (d, *J* = 8.8 Hz, 1H), 7.67–7.56 (m, 6H), 7.48 (dd, *J* = 7.9, 1.8 Hz, 1H), 7.26 (dd, *J* = 7.8, 4.8 Hz, 1H), 6.99 (d, *J* = 2.0 Hz, 1H), 3.62 (s, 3H), 2.22 (s, 3H). ^13^C NMR (101 MHz, DMSO-*d*_6_) δ: 154.67, 153.10, 148.05, 143.55, 137.09, 136.49, 135.46, 135.21, 133.85, 130.44, 130.01 (2C), 129.68, 128.86 (2C), 128.64, 127.97, 123.26, 121.49, 120.28, 114.33, 27.71, 23.15. LC-MS: *t*_R_ = 0.610 min (Purity = 100%); *m*/*z* = 367.2 [M + H]^+^ (anal. calcd. for C_23_H_18_N_4_O: *m*/*z* = 366.1).

#### 8-(6-Aminopyridin-3-yl)-3-methyl-1-phenyl-1,3-dihydro-2H-imidazo­[4,5-*c*]­quinolin-2-one (**30**)

Synthesized from **4b** (0.150 g, 0.42 mmol) and 2-aminopyridine-5-boronic acid pinacol ester (0.111 g, 0.51 mmol) according to Method A to give **30** as a beige solid (0.007 g, 4% yield); ^1^H NMR (300 MHz, DMSO-*d*_6_) δ 8.95 (s, 1H), 8.03 (d, *J* = 8.9 Hz, 1H), 7.86 (d, *J* = 2.4 Hz, 1H), 7.80 (d, *J* = 8.9 Hz, 1H), 7.76–7.62 (m, 5H), 7.39 (dd, *J* = 8.7, 2.6 Hz, 1H), 7.04 (s, 1H), 6.44 (d, *J* = 8.6 Hz, 1H), 6.19 (s, 2H), 3.60 (s, 3H). ^13^C NMR (101 MHz, DMSO-*d*_6_) δ 159.39, 153.04, 145.71, 143.34, 135.57, 135.12, 134.91, 132.90, 130.71, 129.93 (2C), 129.67, 129.08 (2C), 128.33, 124.81, 123.09, 122.65, 114.96, 107.93, 27.67.LC-MS: *t*_R_ = 0.587 min (Purity = 100%); *m*/*z* = 368.1 [M + H]^+^ (anal. calcd. for C_22_H_17_N_5_O: *m*/*z* = 367.1).

### DMPK

All protocols for *in vitro* DMPK studies and mouse PK studies are available in the supplementary document. Animal studies were conducted following guidelines and policies as stipulated in the University of Cape Town Research Ethics Code for Use of Animals in Research and Teaching, after review and approval of the experimental protocol by the UCT Senate Animal Ethics Committee (protocol FHS-AEC 013/032).

## Supplementary Material













## Notes

Synthesis of intermediates; analytical spectra of compounds profiled *in vivo*; parasitology methods; *Pv*PI4K and human kinase assays; *in vitro* ADME assays, and mouse pharmacokinetic and *in vivo* efficacy studies (PDF)
*In vitro* data of all final compounds (mean IC_50_ values ± standard deviation (SD) or standard error (SE)) (CSV)
Molecular formula strings (CSV)
PfPI4K-AZD0156 (PDB)
PfPI4K-Compound 18 (PDB)
HsATM-Compound 18 (PDB)

## ⧓

Department of Chemistry, KwaDlangezwa Campus, University of Zululand, Empangeni 3886, South Africa

## Abbreviations Used

ABS: asexual blood stage
ADME: absorption, distribution, metabolism, and excretion
ATM: ataxia-telangiectasia-mutated
ATP: adenosine triphosphate
ATR: ataxia telangiectasia and Rad3-related
AUC: area under the curve
DCM: dichloromethane
DIPEA: diisopropylethylamine
DMA: dimethylacetamide
DMF: *N*,*N*-dimethylformamide
DMF-DMA: *N*,*N*-dimethylformamide dimethyl acetal
DMPK: drug metabolism and pharmacokinetics
DPPA: diphenylphosphoryl azide
ED_90_: the effective dose for 90% reduction in parasitaemia
HATU: 1-[Bis­(dimethylamino)­methylene]-1*H*-1,2,3-triazolo­[4,5-*b*]­pyridinium 3-oxid hexafluorophosphate
iGC: immature gametocytes
K_2_CO_3_: potassium carbonate
LCMS: liquid chromatography–mass spectrometry
lGC: late-stage gametocytes
LiOH: lithium hydroxide
MAP4K4: mitogen-activated protein kinase kinase kinase kinase 4
MeOH: methanol
MINK1: misshapen-like kinase 1
mTOR: mammalian target of rapamycin
NaH: sodium hydride
NaOH: sodium hydroxide
NEt_3_: triethylamine
NMR: nuclear magnetic resonance
PCy_3_: tricyclohexylphosphine
PdCl_2_(PPh_3_)_2_: bis­(triphenylphosphine)­palladium­(II) chloride
PI4K: phosphatidylinositol-4-kinase IIIβ
SAR: structure–activity relationship
SMILES: simplified molecular input line entry system
TFA: trifluoroacetic acid
THF: tetrahydrofuran
THP: tetrahydropyran
tPSA: topological polar surface area
