# Supplementary material for: Novel Inhibitors of Plasmodium Phosphatidylinositol 4-kinase IIIβ with Low Propensity for Resistance: Life Cycle Stage Activity and In Vivo Efficacy in a Humanized Mouse Malaria Infection Model
Source: J Med Chem. 2025 Aug 14;68(16):17736–51. doi: 10.1021/acs.jmedchem.5c01417 (PMC12406205; doi:10.1021/acs.jmedchem.5c01417)
Supplement: Supplementary file 1 [file jm5c01417_si_001.pdf]

Supporting Information

Novel inhibitors of Plasmodium phosphatidylinositol-4-kinase IIIβ with low propensity for resistance: life cycle stage activity and *in vivo* efficacy in a humanised mouse malaria infection model.

Godwin A. Dziwornu,[a†] *Mmakwena M. Mmonwa,*[b†‡] *Dina Coertzen,*[c] *Liezl Krugmann,*[d] *Nicolaas Salomane,*[d] *Meta Leshabane,*[e] *Jean Thomas,*[c] *Shante da Rocha,*[c] *Janette Reader,*[c] *Keabetswe Masike,*[d] *Mathew Njoroge,*[d] *Nicole Sevilleno,*[e] *Rachael Coyle,*[e] *Nonlawat Boonyalai,*[f] *Emily Mayville,*[g, h] *Marcus C. S. Lee,*[e, f] *David A. Fidock,*[g, h] *Lauren B. Coulson,*[d] *John G. Woodland,*[a, i] *Kathryn J. Wicht,*[a, i] *Sandeep R. Ghorpade,*[a] *Lyn-Marié Birkholtz,*[c, j] *and Kelly Chibale*[a, i*]

[a]Holistic Drug Discovery and Development Centre (H3D), Department of Chemistry, University of Cape Town, Rondebosch 7701, South Africa.

[b]Department of Chemistry, University of Cape Town, Rondebosch 7701, South Africa.

[c]Department of Biochemistry, Genetics and Microbiology, Institute for Sustainable Malaria Control, University of Pretoria, Hatfield, Pretoria, 0028, South Africa.

[d]Holistic Drug Discovery and Development Centre (H3D), Institute of Infectious Disease and Molecular Medicine, University of Cape Town, Observatory, Cape Town 7925, South Africa.

[e]Wellcome Sanger Institute, Wellcome Genome Campus, Hinxton, CB10 1SA, UK.

[f]Biological Chemistry and Drug Discovery, Wellcome Centre for Anti-Infectives Research, University of Dundee, Dundee DD1 5EH, U.K.

[g]Department of Microbiology and Immunology, Columbia University Medical Center, Hammer Health Sciences Center, 701 W. 168th Street, New York, NY 10032, USA.

[h]Center for Malaria Therapeutics and Antimicrobial Resistance, Division of Infectious Diseases, Department of Medicine, Columbia University Irving Medical Center, New York, NY, 10032, USA.

[i]South African Medical Research Council Drug Discovery and Development Research Unit, Department of Chemistry and Institute of Infectious Disease and Molecular Medicine, University of Cape Town, Rondebosch 7701, South Africa.

[j]Department of Biochemistry, Stellenbosch University, Matieland, Stellenbosch 7601, South Africa.

[†]These authors contributed equally to this work.

‡Current Address: Department of Chemistry, KwaDlangezwa Campus, University of Zululand, Empangeni 3886, South Africa.

*Corresponding author: Kelly Chibale (kelly.chibale@uct.ac.za)

Contents

1. Synthesis of Intermediates S3

2. Analytical data of **18** S6

3. *In vitro* asexual blood stage (ABS) antiplasmodium activity S9

4. *In vitro* gametocytocidal assays S9

5. Male gamete exflagellation inhibition assay (EIA) S9

6. Dual Gamete Formation Assay (DGFA) S10

7. Female gametocyte activation assay (FGAA) S10

8. Standard membrane feeding assay (SMFA) S10

9. Activity against liver stage *P. falciparum* NF54 S10

10. *In vitro* parasite reduction ratio (PRR) assays S11

11. Cytotoxicity S12

12. hERG cardiotoxicity assay S12

13. *Pv*PI4K enzyme assay S12

14. Antimalarial resistome barcode sequencing (AReBar) assay S13

15. Profiling against off-target kinases *Hs*ATM, *Hs*PI4Kβ, *Hs*MAP4K4 and *Hs*MINK1 S17

16. Minimum Inoculation of Resistance (MIR) S17

17. *In vitro* ADME assays S18

18. Mouse pharmacokinetic studies S19

19. *In vivo* efficacy and pharmacokinetics in malaria-infected humanized mice S20

20. HPLC traces and NMR spectra of final compounds S22

21. References S91

1. Synthesis of Intermediates

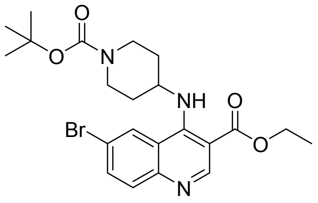

ethyl 6-bromo-4-[[1-[(2-methylpropan-2-yl)oxycarbonyl]piperidin-4-yl]amino]quinoline-3-carboxylate (1a)

To a suspension of ethyl 6-bromo-4-chloroquinoline-3-carboxylate (2000 mg, 6.36 mmol) in *N,N*-dimethylacetamide (25 mL) was added Triethylamine (2.66 mL, 19.07 mmol) . The reaction mixture was stirred at 25 ˚C for 16 h, precipitating a solid. On completion, the crude reaction mixture was transferred to an ice-cold water (100 mL). The precipitate was filtered, washed with cold water, and dried to give **1a** as a beige solid (2600 mg, 84% yield). LC-MS: t_R = 0.896 min (Purity = 98%); *m/z* = 478.1 $[M+H]^+$ (anal. calcd. for $C_{22}H_{28}BrN_3O_4$: *m/z* = 477.1).

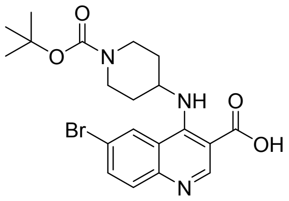

6-bromo-4-((1-(tert-butoxycarbonyl)piperidin-4-yl)amino)quinoline-3-carboxylic acid (2a)

To a solution of **1a** (650 mg, 1.36 mmol) in tetrahydrofuran (8 mL) was added water (2 mL) and stirred vigorously. To the mixture was added Lithium hydroxide monohydrate (228 mg, 5.44 mmol). The reaction was stirred at 25 ˚C for 6 h. When completion, the organics was removed *in vacuo* leaving an aqueous residue, which was diluted with ethyl acetate. The mixture was acidified (pH 2-5) with 2M aqueous HCl solution, precipitating out a white solid. The precipitate was filtered and washed with water severally. The residue was dried to give **2a** as a white solid (550 mg, 88% yield). LC-MS: t_R = 0.883 min (Purity = 98%); *m/z* = 452.1 $[M+H]^+$ (anal. calcd. for $C_{20}H_{24}BrN_3O_4$: *m/z* = 451.1).

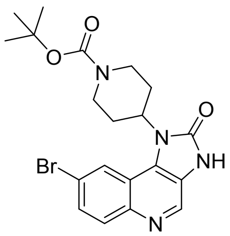

tert-butyl 4-(8-bromo-2-oxo-2,3-dihydro-1H-imidazo[4,5-c]quinolin-1-yl)piperidine-1-carboxylate (3a)

To a suspension of **2a** (2640 mg, 5.86 mmol) in DMF (10 mL) was added Triethylamine (4.9 mL, 35.17 mmol). The mixture was stirred for 15 minutes after which Diphenylphosphoryl azide (3.79 mL, 17.59 mmol) was added. The reaction was heated at 60 ˚C, forming the product in 30 minutes. The solvent was removed in vacuo. The residue was taken in water and filtered, washing with more water, followed by diethyl ether. The solid was dried to give **3a** as a yellow solid (2078 mg, 79% yield). LC-MS: t_R = 0.939 min (Purity = 98%); *m/z* = 447.1 $[M+H]^+$ (anal. calcd. for $C_{20}H_{23}BrN_4O_3$: *m/z* = 446.1).

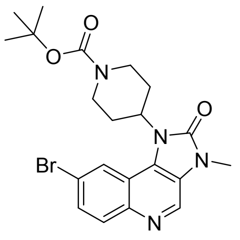

tert-butyl 4-(8-bromo-3-methyl-2-oxo-2,3-dihydro-1H-imidazo[4,5-c]quinolin-1-yl)piperidine-1-carboxylate (4a)

To a suspension of **3a** (1000.mg, 2.24 mmol) in DMF (15 mL) was added *N,N*-Dimethylformamide dimethyl acetal (0.89 mL, 6.71 mmol). The reaction was heated at 80 ˚C for 16 h. The solvent was removed *in vacuo*. The residue was taken in ice-cold water, triturated, and filtered. The residue was washed with cold water and dried to give 4a as a white solid (898 mg, 87% yield). LC-MS: t_R = 1.012 min (Purity = 98%); *m/z* = 461.1 $[M+H]^+$ (anal. calcd. for $C_{21}H_{25}BrN_4O_3$: *m/z* = 460.1).

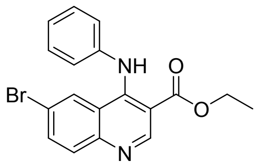

ethyl 6-bromo-4-(phenylamino)quinoline-3-carboxylate (1b)

To a suspension of ethyl 6-bromo-4-chloroquinoline-3-carboxylate (800 mg, 2.54 mmol) in acetic acid (7 mL) was added Potassium acetate (349 mg, 3.56 mmol) and Aniline (0.28 mL, 3.05 mmol). The reaction mixture was stirred at 25 ˚C until completion (1 h). The crude reaction mixture was transferred to an ice-cold water (100 mL). The precipitate was filtered, washed with cold water, and dried to give **1b** as a white solid (835 mg, 85% yield). LC-MS: t_R = 0.961 min (Purity = 96%); *m/z* = 371.1 $[M+H]^+$ (anal. calcd. for $C_{18}H_{15}BrN_2O_2$: *m/z* = 370.1).

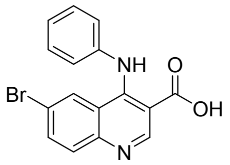

6-bromo-4-(phenylamino)quinoline-3-carboxylic acid (2b)

Synthesized from **1b** (835 mg, 2.16 mmol) as described for **2a** above. White solid (650 mg, 88% yield); LC-MS: t_R = 0.867 min (Purity = 98%); *m/z* = 343.1 $[M+H]^+$ (anal. calcd. for $C_{16}H_{11}BrN_2O_2$: *m/z* = 342.1).

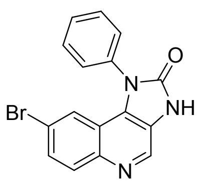

8-bromo-1-phenyl-1,3-dihydro-2H-imidazo[4,5-c]quinolin-2-one (3b)

Synthesized from **2b** (650 mg, 1.89 mmol) as described for **3a** above. White solid (593 mg, 90% yield); LC-MS: t_R = 0.870 min (Purity = 98%); *m/z* = 340.0 $[M+H]^+$ (anal. calcd. for $C_{16}H_{10}BrN_3O$: *m/z* = 339.0).

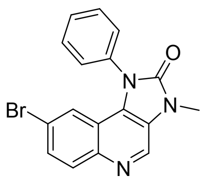

8-bromo-3-methyl-1-phenyl-1,3-dihydro-2H-imidazo[4,5-c]quinolin-2-one (4b)

Synthesized from **3b** (300 mg, 0.86 mmol) as described for **4a** above. Yellow solid (250 mg, 82% yield); LC-MS: t_R = 0.939 min (Purity = 98%); *m/z* = 354.0 $[M+H]^+$ (anal. calcd. for $C_{17}H_{12}BrN_3O$: *m/z* = 353.0).

2. Analytical data of 18

HPLC_UV and MS spectra of **18**

Additional Info : Peak(s) manually integrated

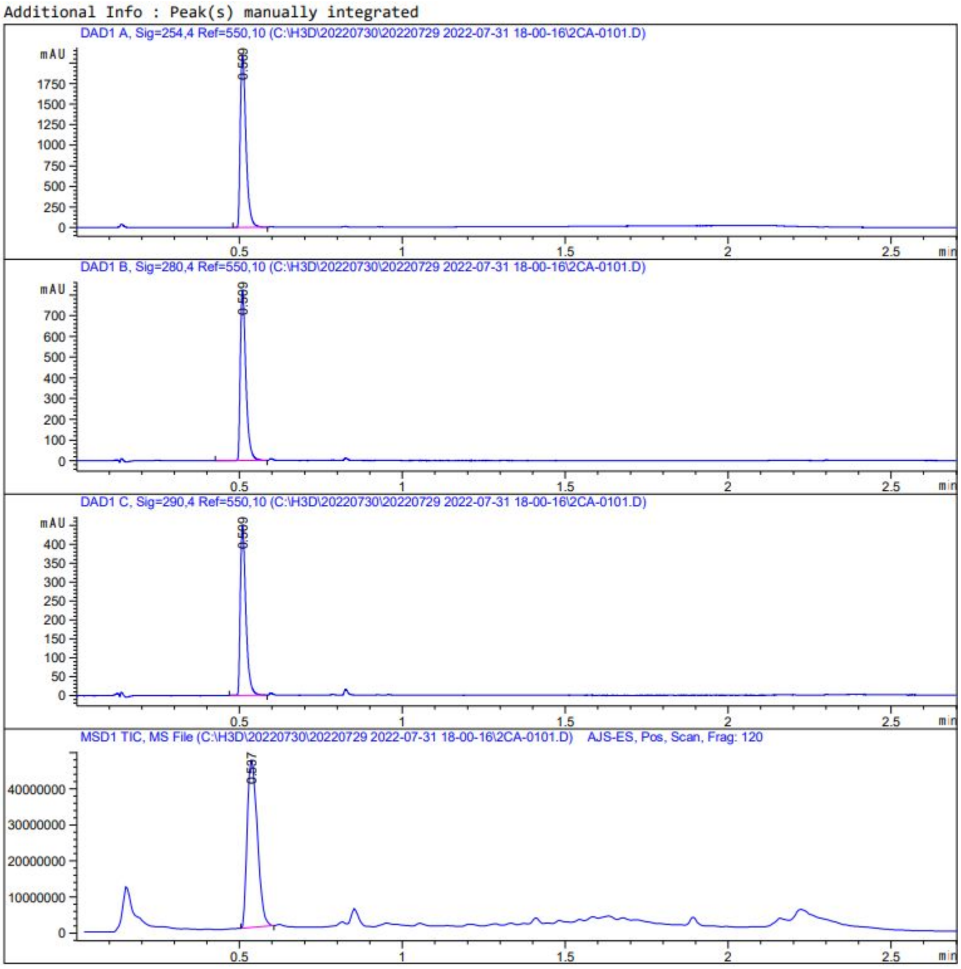

Signal 1: DAD1 A, Sig=254,4 Ref=550,10

Peak #	RetTime [min]	Type	Width [min]	Area [mAU*s]	Height [mAU]	Area %
1	0.509	BB	0.0187	2531.15161	2084.32422	100.0000
Totals :				2531.15161	2084.32422	

Signal 2: DAD1 B, Sig=280,4 Ref=550,10

Peak #	RetTime [min]	Type	Width [min]	Area [mAU*s]	Height [mAU]	Area %
1	0.509	BB	0.0183	969.16718	818.99579	100.0000
Totals :				969.16718	818.99579	

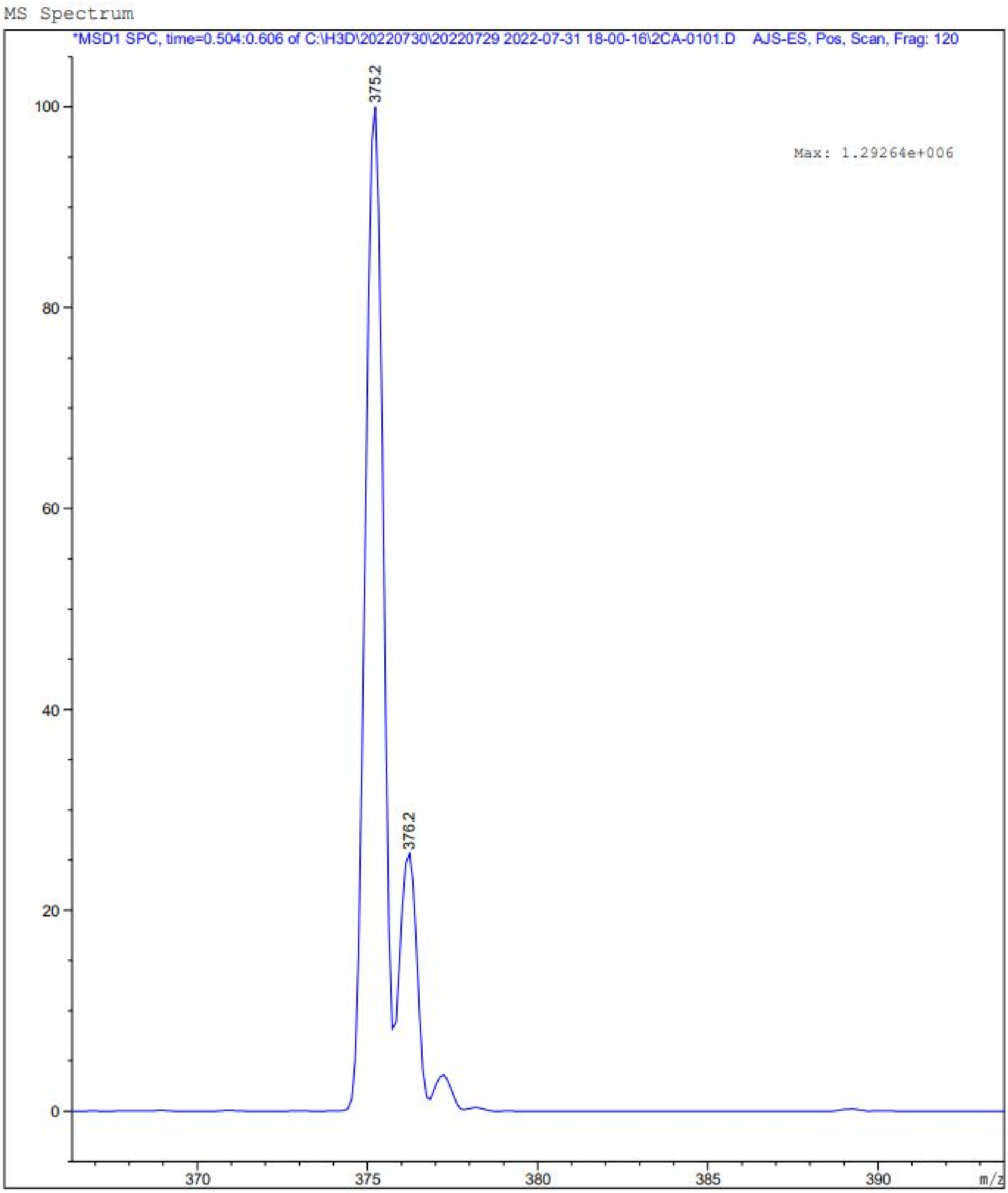
MS Spectrum
*MSD1 SPC, time=0.504:0.606 of C:\H3D\20220730\20220729 2022-07-31 18-00-16\2CA-0101.D AJS-ES, Pos, Scan, Frag: 120
375.2
376.2
Max: 1.29264e+006
100
80
60
40
20
0
370
375
380
385
390
m/z

^{1}H NMR spectrum of **18**

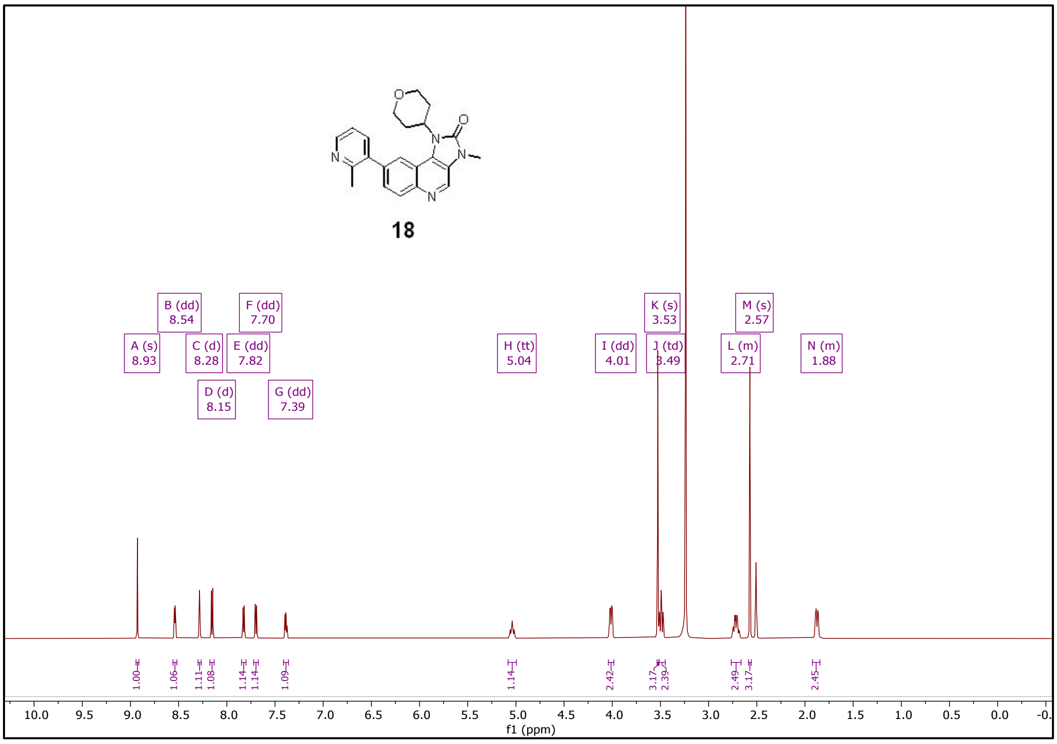

^{13}C NMR spectrum of **18**

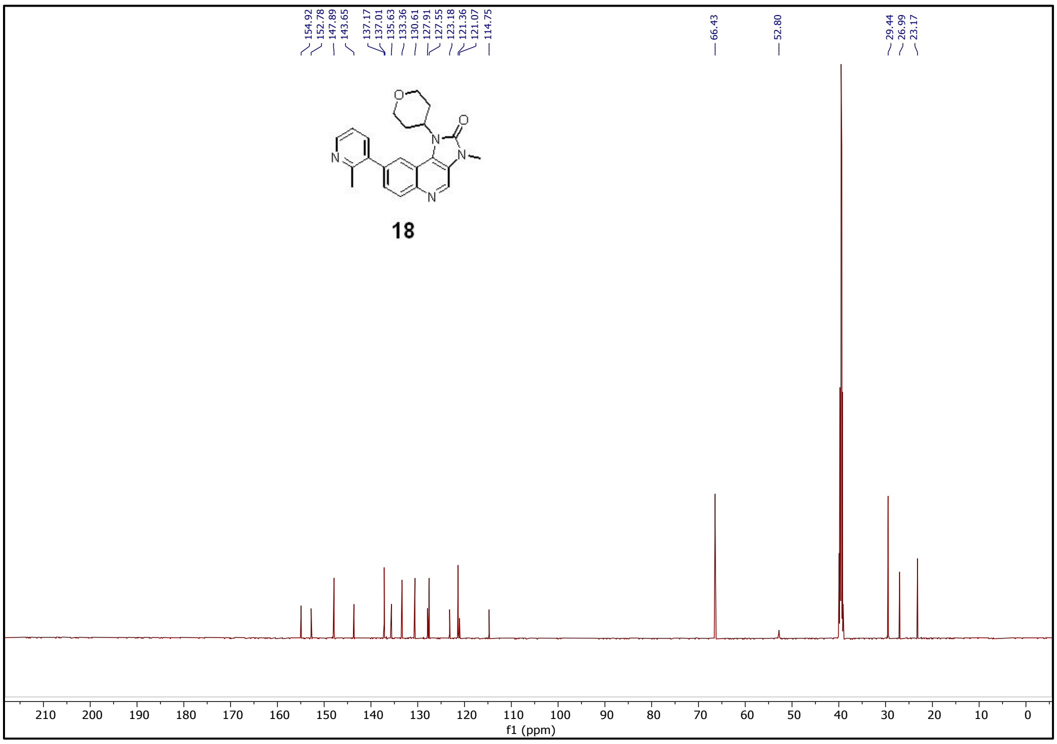

3. *In vitro* asexual blood stage (ABS) antiplasmodial activity

For the *in vitro* 96 h SYBR Green I-based antiplasmodial assays, the asexual *Pf*NF54 parasite strain was obtained from the Malaria Research and Reference Reagent Resource Centre (MR4 BEI resources, Manassas, USA) and cultivated under adapted conditions as previously described.[1] Parasites were maintained daily at 5% haematocrit (A^+/O^+ human erythrocytes) in complete culture media [RPMI 1640 supplemented with 25 mM HEPES, 20 mM D-glucose, 200 µM hypoxanthine, 0.2% (w/v) sodium bicarbonate (Sigma-Aldrich), 24 µg/mL gentamicin, and 0.5% (w/v) AlbuMAX II, Gibco)] under hypoxic conditions (90% N_2, 5% O_2 and 5% CO_2, AFROX, SA) at 37˚C with agitation. Cultures were synchronised to 95% ring stage using 5% (w/v) D-sorbitol (Sigma-Aldrich). Parasite growth and morphology were monitored microscopically using Giemsa-stained thin smears at 100× magnification. The SYBR Green I parasite proliferation assay was performed with *Pf*NF54 parasites (1% parasitaemia, 1% haematocrit) for 96 h under hypoxic conditions, using chloroquine as an internal reference control. Following incubation, parasite proliferation was determined after the addition of 1× SYBR Green lysis buffer (1× SYBR Green I, 20 mM Tris-HCl, pH 7.5, 5 mM EDTA, 0.008% saponin (w/v) and 0.08% Triton X-100) and incubated for 1 h at room temperature in the dark. Fluorescence was quantified with the GloMaxR-Multi+ Detection System (485/538 nm). *In vitro* IC_{50} values were determined using GraphPad Prism V6 (log(inhibitor) vs. normalised response – variable slope). Data represent three independent biological repeats (n=3), each performed in technical triplicates.

Cross-resistance profiling against lab-raised resistant lines and field isolates was performed at Swiss TPH using the [^{3}H]-hypoxanthine incorporation assay, as previously reported.[2–4]

4. *In vitro* gametocytocidal assays

Stage-specific gametocytocidal action of the compounds were determined using against immature (>90% stage II–III), late (>90% stage IV–V) and mature (>95% stage V) gametocytes using the *P. falciparum* luciferase reporter line, 3D7elo1-pfs16-CBG99 (kind gift from Pietro Alano, ISS, Italy), as previously described.[5] Drug assays were set up at 1.5% gametocytaemia and 2% haematocrit for a 48 h incubation under drug pressure in a gas chamber (90% N_2, 5% O_2, and 5% CO_2) at 37˚C, with methylene blue and MMV390048 serving as internal reference controls. The luciferase reporter assay was performed using 0.5 mM non-lysing d-luciferin (Regis Technologies) in citrate buffer (50 mM citric acid, 50 mM trisodium citrate hydrate) with bioluminescence detection using the GloMax®-Multi Detection System with Instinct® software. *In vitro* IC_{50} values were determined using GraphPad Prism V8 software for three independent biological repeats (n=3) each performed in technical duplicates.

5. Male gamete exflagellation inhibition assay (EIA)

The male gamete exflagellation inhibition assay (EIA)[6] was performed on *Pf*NF54 mature (>95% stage V) gametocytes treated with 2 µM drug in 50% A^+ male serum for 48 hours under hypoxic conditions at 37˚C (2 µM methylene blue as positive control). Following treatment male gametogenesis was

induced with 100 µM xanthurenic acid (Sigma-Aldrich) at room temperature for 16 minutes in a Neubauer chamber followed by capturing of temporal movement of exflagellating centres by video microscopy (Carl Zeiss NT 6V/10 W Stab microscope, MicroCapture camera, 10× magnification) for 8 minutes at 30-second intervals for 8–10 seconds. Total exflagellating centres were quantified using ICY (open-source imaging software GPLv3) normalised to an untreated control.

6. Dual Gamete Formation Assay (DGFA)

The compounds were evaluated for their ability to inhibit the "functional viability" of mature stage V gametocytes as reported by their ability to undergo onward development and form gametes, as previously described.[7,8]

7. Female gametocyte activation assay (FGAA)

The compounds were evaluated for their ability to inhibit female gamete activation, as previously described.[9] Gametocytes (>95% stage V) were treated with compounds (2 µM) for 48 h before triggering gamete formation. Female gamete activation was induced by both a temperature drop and the addition of 100 µM xanthurenic acid. Monoclonal anti-Pfs25 antibody (BEI Resources catalogue number MRA-28; 1:1000 dilution) conjugated to FITC was used to detect female gametes. Image acquisition was performed using a Zeiss Axio Lab.A1 epifluorescence microscope with a 100/1.4 numerical aperture (NA) oil immersion objective and a Zeiss Axiocam 202 mono digital camera. Using a 100x objective, 30 images were taken per sample and analysed manually. The size, roundness and intensity of fluorescence of activated female gametocytes were evaluated.

8. Standard membrane feeding assay (SMFA)

Performed as previously described.[10] Briefly, the SMFA was carried out using *Anopheles coluzzii s.s.* females (colonised in 2009 from the Democratic Republic of the Congo, G3 colony).[11,12] The mosquitoes were maintained under BSL2 insectary conditions (80% humidity, 25°C, 12-hour day/12-hour night cycle with 50-minute dusk/dawn transitions) with ad libitum access to 10% sucrose supplemented with 0.05% (*v*/*v*) 4-aminobenzoic acid. Mature *Pf*NF54 gametocytes (>98% stage V, 1.5 to 2.5% gametocytaemia, and 50% haematocrit) were treated with 2 µM of compound with DMSO as a vehicle control for 48 hours before mosquito feeding. SMFA was carried out as previously described. Two independent biological experiments were carried out (total of 25 mosquitoes in treated control groups). The TRA (transmission-reducing activity, reduction in oocyst intensity: %TRA $\frac{Ci-Ti}{Ci} * 100$, where *i*: oocyst number (intensity), *C*: control and *T*: treated) and the TBA, which measures the reduction in prevalence of mosquitoes infected with oocysts(percentage of block of transmission or reduction in prevalence, % TBA: $\frac{Cp-Tp}{Cp} * 100$, where *p*: oocyst prevalence (intensity), *C*: control and

T: treated),[13] were determined. Data were analysed using GraphPad Prism, and the Mann-Whitney U test was used to compare the statistical significance between the treatment and control groups.

9. Activity against liver stage *P. falciparum* NF54

Primary human hepatocytes are cultured for 2 days and then overlaid with *P. falciparum* NF54 sporozoites and compounds.[14] Supernatant is refreshed daily with fresh compounds. 4 days post-infection, hepatocytes are stained for the presence of liver stage parasites. Cryopreserved human primary hepatocytes (H1500.H15B+ Lot No. HC0-6, TebuBio or F00995-P Lot No. IRZ, BioIVT) were thawed and seeded at 60,000 (HC0-6) or 18,000 (IRZ) cells per well in collagen-coated 96w or 384w microtiter plates, respectively. Cells were cultured at 37˚C in 5% CO_2. Five and 24 hours post plating, medium was replaced for donor HC0-6, whereas for donor IRZ medium was refreshed 24 hours post plating only. 48 hours post plating, salivary glands from *Plasmodium*-NF54-infected *Anopheles stephensi* mosquitoes were dissected, and 50,000 or 25,000 sporozoites per well were added to 96w or 384w plates respectively and allowed to infect for 3 hours. Sporozoites were then aspirated and compounds diluted in medium were added to the hepatocytes. Compounds were tested in duplicate. Medium containing compounds was refreshed daily for four days. Hepatocytes were fixed with ice-cold methanol and monolayers were blocked with 10% hiFBS in PBS. Schizonts were stained with rabbit anti-*Pf*HSP70 (heat shock protein 70) in 10% hiFBS for 1-2 hours followed by incubation with a mixture of secondary goat anti-rabbit AlexaFluor 594 antibody and 4′,6-Diamidine-2′-phenylindole dihydrochloride (DAPI) in 10% hiFBS for 30 min. Samples were washed with PBS containing 0.05% Tween 20 between different steps. Cells were imaged on a PicoExpress high content imager (Molecular Devices) and images were analyzed automatically using CellReporterXpress software. Data were analyzed by logistic regression using a four-parameter (Hill equation) model and a least-squares method to find the best fit.

10. *In vitro* parasite reduction ratio (PRR) assays

The assay uses limiting dilution technique to quantify number of parasites that remain viable after drug treatment. *P. falciparum* strain 3D7 was treated with the selected drug at concentration corresponding to 10X IC_{50}. Parasites were treated for 120 hours. Drug was renewed daily over the entire treatment period. Samples of parasites were taken from the treated culture every 24 hours (0 - for the control of initial number of parasites, followed by 24, 48-, 72-, 96- and 120-hours' time points), drug was washed out and drug-free parasites were cultured in 96 well plates by adding fresh erythrocytes and new culture media. To quantify number of viable parasites after treatment, 3-fold serial dilution was used with the above-mentioned samples after removing the drug. Parasites were cultured in microtiter plates to allow all wells with viable parasites, to render detectable parasitaemia. Four independent serial dilutions were done with each sample to correct experimental variations. After 22 days of culturing, samples were taken to examine growth. Additional sampling was done after 28 days to confirm growth/no growth. The number of viable parasites was determined by counting the number of wells with growth.

The number of viable parasites was back calculated by using the formula X^{n-1} where n is the number of wells able to render growth and X the dilution factor (when n=0 number of viable parasites was estimated as zero). The experimental number of viable parasites after each treatment was back calculated by using formula X^{n-1} where n was the number of wells able to render growth and X the dilution factor (when n=0 number of viable parasites is estimated as zero). The number of initial parasites (no treatment) was calculated in a similar manner and used to calculate a normalization factor to correct the deviation of the experimental determination from the theoretical number of initial parasites (10^5, 100μl from a 10^6 parasites/ml inoculum). Normalization factor was used to correct all the experimental data for a starting number of parasites equal to 10^5 and enables comparison between different experiments. Additional parameters were calculated such as lag phase (time needed to observe the maximal killing effect of the drug being tested), PRR (parasite reduction ratio; such as the reduction in number of parasites by the drug in a parasite life cycle) and $PCT_{99.9\%}$ (parasite clearance time; the time required to kill 99.9% of the initial population). Chloroquine, pyrimethamine, atovaquone and artesunate are used in each assay to validate the assay and allow for comparative classification on the killing rate of the tested compound.

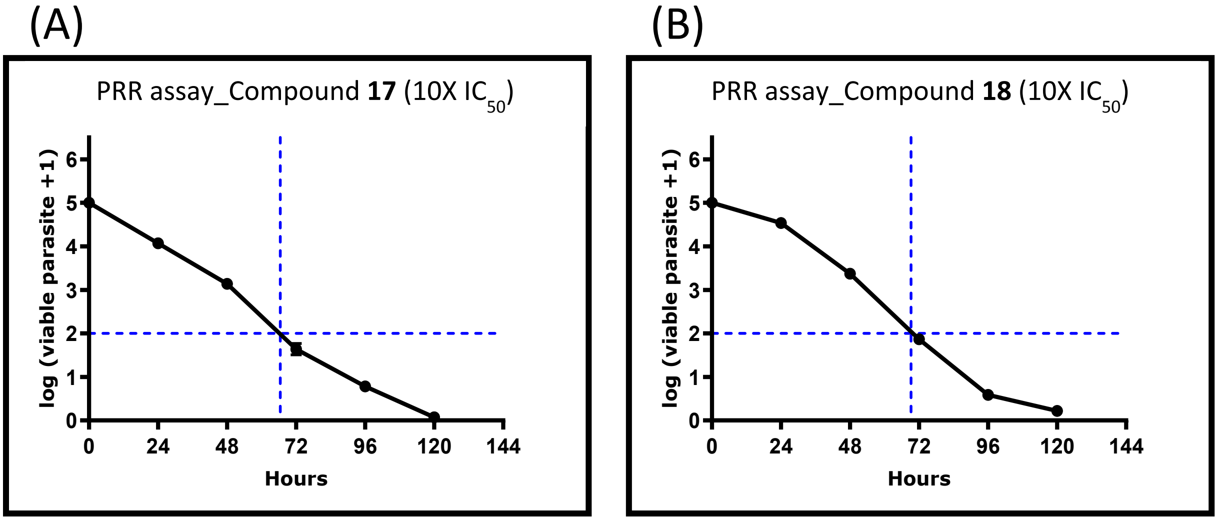

Figure S1: Killing profile of *P. falciparum* 3D7 after treatment with 10X IC_{50} concentration of (A) compound **17** and (B) compound **18**.

11. Cytotoxicity

The IC_{50} against mammalian cells was determined using the MTT assay over 48h as described.[15] Samples were prepared to a 10 mmol/L stock solution in 100% DMSO and stored at room temperature until testing. Dilutions to the desired starting concentration were freshly prepared in growth media on each occasion of the experiment. Cells were plated 24h prior to exposure and allowed to adhere to the well surfaces. After 24h, media was aspirated, compounds and fresh media were introduced, and plates were returned to the incubator for a further 44h of growth; thereafter 25mL of sterile MTT dye was

added to each well and plates were incubated for the final 4h of the experiment. Plates were centrifuged for 10 minutes to concentrate the reduced dye crystals; the supernatant was aspirated and 50mL DMSO added to dissolve the dye. Absorbance was measured at 540nM, and the IC_{50} was determined by regression analysis of these values using the Dotmatics software platform.

12. hERG cardiotoxicity assay

The human-ether-a-go-go related gene (hERG) inhibition assay was carried out at B'SYS GmBH, Switzerland. The whole-cell patch-clamp technique was used to investigate the effects of the compounds and E-4031 (positive control), on hERG potassium channels stably expressed in CHO cells.

13. *Pv*PI4K enzyme assay

Full-length *Pv*PI4Kβ (PVX_098050) recombinant protein was expressed in a baculovirus-insect cell expression system and purified as previously described.[16,17] Briefly, *N*-terminal His-tagged recombinant *Pv*PI4Kβ protein was purified using a HisTrap HP column (GE Healthcare), followed by size-exclusion chromatography (HiLoad 16/600 Superdex 200 pg column, GE Healthcare). Final buffer composition of purified protein was 20 mM HEPES pH 7.5, 500 mM NaCl, 5% (*v*/*v*) glycerol, 10 mM β-mercaptoethanol.

*Pv*PI4Kβ kinase inhibition assays were performed using the ADP-Glo Kinase Assay kit (Promega) to measure ADP formation. l-alpha-phosphatidylinositol (PI; Avanti Polar Lipid, cat. 840042P) dissolved in 3% *n*-octylglucoside to a stock concentration of 20 mg/mL was used as the lipid substrate. Briefly, a three-fold serial dilution of each inhibitor was carried out in DMSO and inhibitors were subsequently diluted into assay buffer (25 mM HEPES pH 7.4, 100 mM NaCl, 3 mM $MgCl_2$, 1 mM DTT, 0.025 mg/ml BSA, 0.2% (*v*/*v*) Triton X-100) to 1.5 × the final required concentration. 2 µL of each inhibitor dilution was transferred into a white 384-shallow well plate (Nunc #264706). A MANTIS® Liquid Handler (Formulatrix) was used to dispense the remaining assay components. 0.5 µL *Pv*PI4Kβ protein was added and following a five-minute pre-incubation with inhibitor, 0.5 µL substrate buffer (ATP and PI) was added to each well. The final 3 µL kinase reaction contains ~6 nM *Pv*PI4Kβ protein, 10 µM ATP, 0.1 mg/ml PI, 1% (*v*/*v*) DMSO and inhibitor in assay buffer. Reactions were incubated for 40 minutes at 22˚C (resulting in < 10% ATP conversion). ADP formation was measured using the ADP-Glo Kinase Kit (Promega). Briefly, 2 µL ADP-Glo reagent containing 10 mM $MgCl_2$ was added to each well and incubated for 40 minutes at 22˚C to deplete the remaining ATP. 2 µL of Kinase Detection Reagent was then added and the reaction was incubated for a further 30 minutes at 22˚C. The plate was sealed with an adhesive foil seal for all incubation steps. Luminescent signal was measured using the EnSpire Multimode Plate Reader (PerkinElmer). The data was normalised based on the 100% activity controls (1% DMSO only) and the 100% inhibition controls (10 µM sapanisertib[9]). The mean IC_{50} value was calculated from $n \geq 3$ independent experiments, each with technical duplicates (log(inhibitor) vs.

normalised response – variable slope). IC_{50} values within three-fold from independent experiments are considered reproducible.

14. Antimalarial resistome barcode sequencing (AReBar) assay

The AReBar assay was conducted as previously described.[18] The list of parasite lines in the AReBar pool is listed below in Table S1.

Table S1: Parasite lines present in the AReBar screening pool. Table shows the line name (including strain background and mutation), the gene description and gene ID of the mutated gene.

Line name	Gene Description	Gene ID
3D7	Wild type	NA
3D7 ABCI3 R2180P	ABC transporter I family member 1	PF3D7_0319700
3D7 ACS10 M300I	Acyl CoA synthase	PF3D7_0525100
3D7 ACS11 D648Y	Acyl CoA synthase	PF3D7_1238800
3D7 ACS11 E668K	Acyl CoA synthase	PF3D7_1238800
3D7 ATP2 CNV2	Phospholipid-transporting ATP2	PF3D7_1219600
3D7 DHFR-TS G378E	Dihydrofolate reductase-thymidylate synthase	PF3D7_0417200
3D7 DHFR-TS I403L	Dihydrofolate reductase-thymidylate synthase	PF3D7_0417200
3D7 FTb A515T	Farnesyltransferase subunit beta	PF3D7_1147500
3D7 MDR2 K840N	Multidrug resistance protein 2	PF3D7_1447900
3D7 NCR1 A1108T	Niemann-Pick type C1-related protein	PF3D7_0107500
Dd2	Wild type	NA
Dd2 AcAS A597V	Acetyl-CoA synthetase	PF3D7_0627800
Dd2 AsnRS R487S	Asn-tRNA synthetase	PF3D7_0509600
Dd2 ATP4 A353E + CARL I1139K	ATP4+CARL double mutant	PF3D7_1211900
Dd2 ATP4 G358S	Non-SERCA-type Ca2+ -transporting P-ATPase (ATP4)	PF3D7_1211900
Dd2 ATP4 L927V	Non-SERCA-type Ca2+ -transporting P-ATPase (ATP4)	PF3D7_1211900
Dd2 CARL I1139K	Cyclic amine resistance locus (CARL)	PF3D7_0321900
Dd2 CARL L1073Q	Cyclic amine resistance locus (CARL)	PF3D7_0321900
Dd2 CARL V1103L	Cyclic amine resistance locus (CARL)	PF3D7_0321900
Dd2 CPSF Y408S E	Cleavage and polyadenylation specific factor	PF3D7_1438500
Dd2 CPSF Y408S E	Cleavage and polyadenylation specific factor	PF3D7_1438500
Dd2 CRT M343L	Chloroquine resistance transporter	PF3D7_0709000

Dd2 CSC1 L800P	CSC1-like protein putative	PF3D7_1250200
Dd2 cytBC1 G33V	Cytochrome b	PF3D7_MIT02300
Dd2 cytBC1 V284L	Cytochrome b	PF3D7_MIT02300
Dd2 DHFR-TS S216R	Dihydrofolate reductase-thymidylate synthase	PF3D7_0417200
Dd2 DHODH C276Y	Dihydroorotate dehydrogenase	PF3D7_0603300
Dd2 DHODH F227I	Dihydroorotate dehydrogenase	PF3D7_0603300
Dd2 DHODH I263F	Dihydroorotate dehydrogenase	PF3D7_0603300
Dd2 DHODH L531F	Dihydroorotate dehydrogenase	PF3D7_0603300
Dd2 eEF2 L755F	Elongation factor 2	PF3D7_1451100
Dd2 eEF2 Y186N	Elongation factor 2	PF3D7_1451100
Dd2 GGPPS S228T	Geranylgeranyl diphosphate synthase	PF3D7_1128400
Dd2 IleRS E180D	Ile-tRNA synthetase	PF3D7_1332900
Dd2 IleRS V500A	Ile-tRNA synthetase	PF3D7_1332900
Dd2 IleRS L810F	Ile-tRNA synthetase	PF3D7_1332900
Dd2 kelch13 C580C	Kelch protein K13	PF3D7_1343700
Dd2 kelch13 C580Y	Kelch protein K13	PF3D7_1343700
Dd2 kelch13 R539T	Kelch protein K13	PF3D7_1343700
Dd2 MCP D195N	Mitochondrial carrier protein	PF3D7_0908800
Dd2 MDR1 F1072L	Multidrug resistance protein 1	PF3D7_0523000
Dd2 PI4K CNV	Phosphatidylinositol 4-kinase	PF3D7_0509800
Dd2 PI4K S1320L+L1418F	Phosphatidylinositol 4-kinase	PF3D7_0509800
Dd2 PI4K S743F+H1484Y	Phosphatidylinositol 4-kinase	PF3D7_0509800
Dd2 ProRS L482H	Pro-tRNA synthetase	PF3D7_1213800
Dd2 PROTB5 A20V	Proteasome beta 5 26S (A80V immature)	PF3D7_1011400

(A) Growth of the AReBar pool.

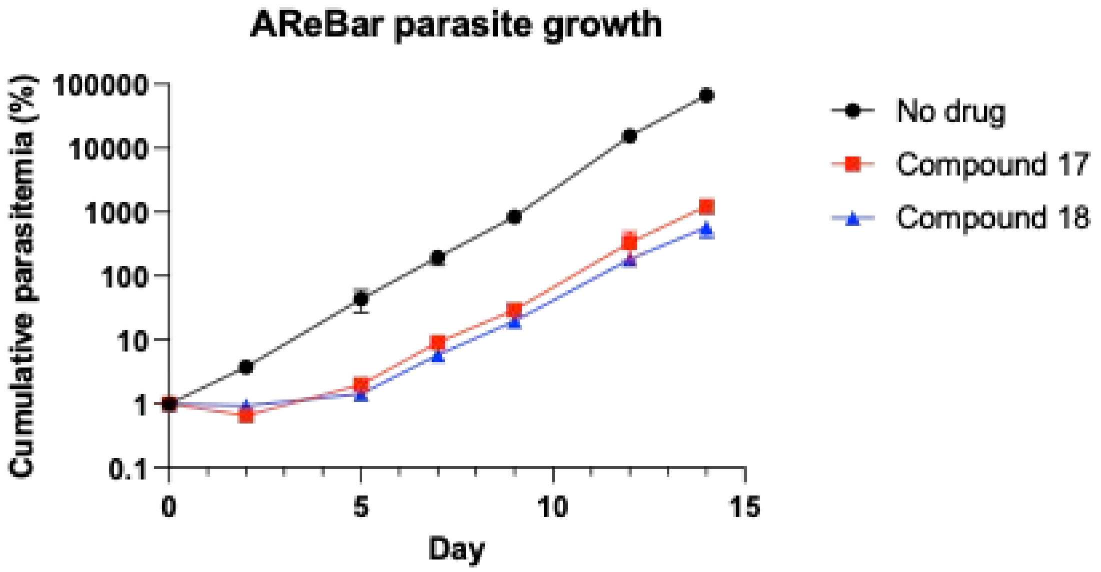

(B) Compound **17** log fold change relative to untreated control

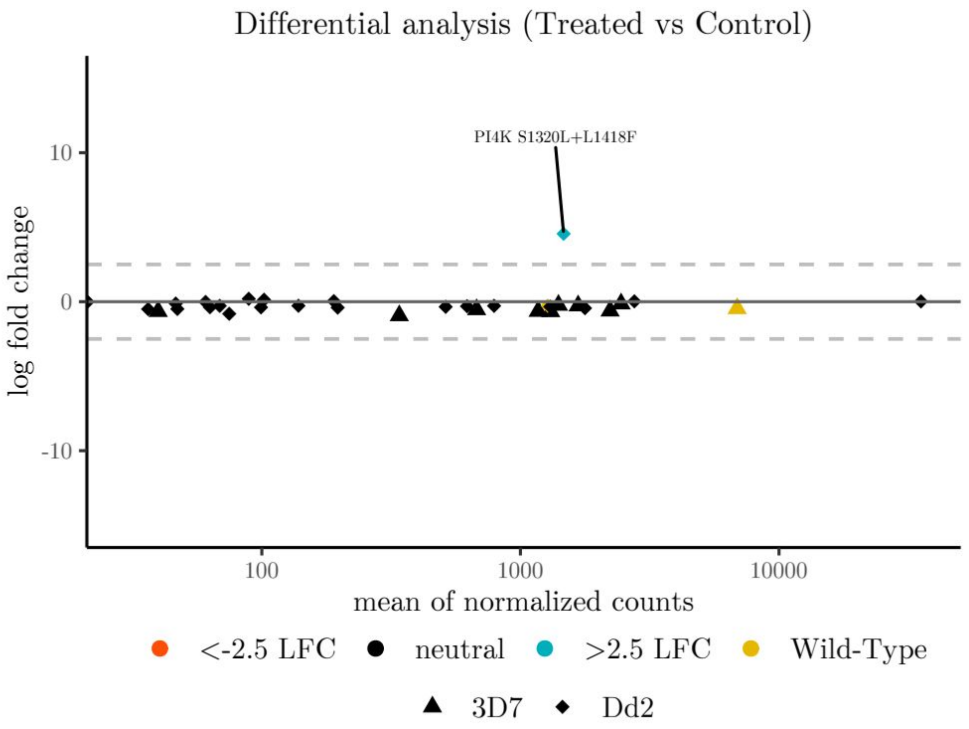

(C) Compound **18** log fold change relative to untreated control

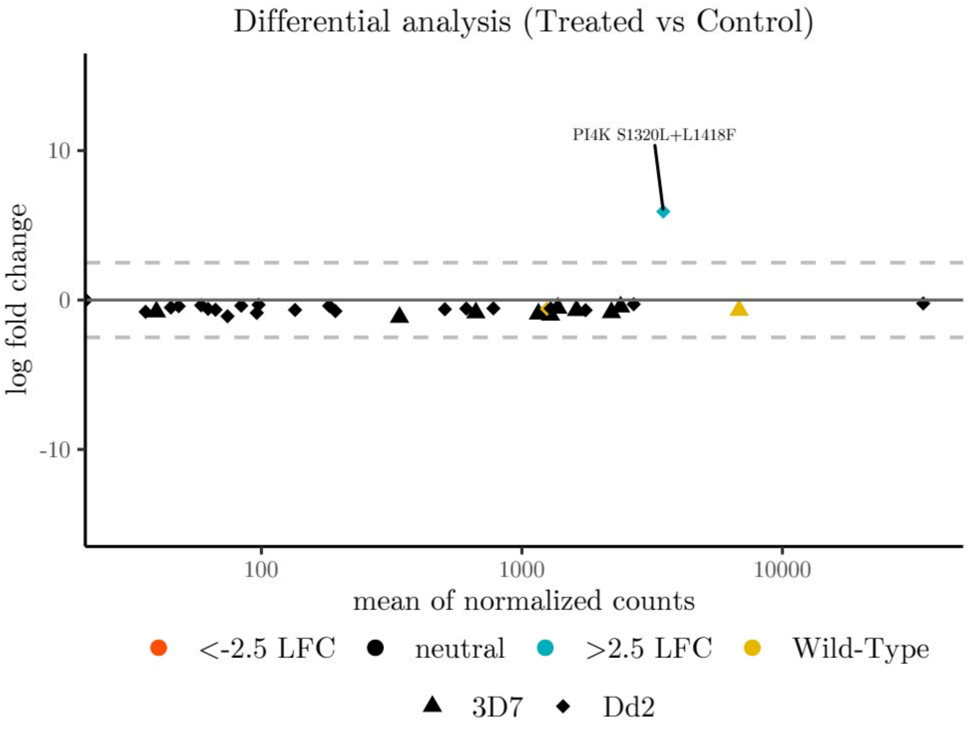

Figure S2: AReBar screening. (A) Growth (cumulative parasitaemia) of the AReBar pool over the 14-day assay period. Shown are cumulative parasitaemia of the pool treated with 3×IC_{50} of either compound 17 or compound 18, or the untreated control. (B,C) Relative change in abundance of parasite lines under compound pressure (3×IC_{50}). The log2 fold change of each line is shown for (B) Compound 17 and (C) Compound 18, with 3D7 (triangle) and Dd2 (diamond) strain backgrounds indicated. For both compounds the PI4K S1320L+L1418F mutant showed a >2.5 log fold change signifying cross resistance.

15. Biochemical enzyme evaluation against off-target kinases *Hs*ATM, *Hs*PI3Kα, *Hs*PI4Kβ, *Hs*MINK1, and *Hs*MAP4K4.

The biochemical inhibitory activities (IC_{50} values) of the compounds against the human kinases *Hs*ATM, *Hs*PI4Kβ, *Hs*MAP4K4 and *Hs*MINK1 were measured at Reaction Biology Corporation (Devault, Pennsylvania, USA) with an ADP-Glo Kinase Assay platform (Promega). The compounds were tested in 10-dose IC_{50} mode with a three-fold serial dilution starting at 10 μM. Control compounds PI-103 (for PI3Kα) and PIK-93 (for PI4Kβ) were tested in 10-dose IC_{50} mode with three-fold serial dilution starting at 1 μM while control compound staurosporine (for MINK1 and MAP4K4) was tested in 10-dose IC_{50} mode with four-fold serial dilution starting at 20 μM. AZD0156 was used as control for the ATM assay was tested in 10-dose IC_{50} with 3-fold serial dilution starting at 0.1 μM. Reactions were carried out at 10 μM ATP (for ATM also tested at 500 μM). A detailed description of the ADP-Glo assay is available online (https://www.promega.co.uk/products/cell-signaling/kinaseassays-and-kinase-biology/adp-glo-kinase-assay/?catNum=V6930).

16. Minimum Inoculation of Resistance (MIR)

16.1 Drug preparation

Drug stocks were made at 10 mM and 1 mM in dimethyl sulfoxide (DMSO). Aliquots in use were stored at -20˚C and long-term storage was at -80˚C. All *in vitro* studies were done such that the final DMSO concentration was <0.5%.

16.2 Parasite culture

P. falciparum asexual blood stage (ABS) parasites were cultured at 2% hematocrit (HCT) in human RBCs in RPMI-1640 media, supplemented with 25 mM HEPES, 50 mg/L hypoxanthine, 2mM L-glutamine, 0.21% sodium bicarbonate, 0.5% (wt/vol) AlbuMAXII (Invitrogen) and 10 μg/mL gentamycin, in modular incubator chambers (Billups-Rothenberg) at 5% O_2, 5% CO_2 and 90% N_2 at 37˚C. Dd2 parasites were obtained from T. Wellems (NIAID, NIH). Dd2-B2 is a genetically homogenous line that was cloned from Dd2 by limiting dilution in the Fidock lab.

16.3 Drug susceptibility assays

To define the IC_{50} of ABS parasites, Dd2-B2 ring-stage cultures at 0.3% parasitemia and 1% hematocrit were exposed for 72 hours to a range of ten drug concentrations that were 2-fold serially diluted in duplicates along with drug-free controls. Parasite survival was assessed by flow cytometry on an iQue flow cytometer (Sartorius) using SYBR Green and MitoTracker Deep Red FM (Life Technologies) as nuclear stain and vital dyes respectively.

17. *In vitro* ADME assays

17.1 Solubility

Solubility was performed using a miniaturized shake flask method. 10 mM stock solutions of each compound were used to prepare calibration standards (10-220 μM) in DMSO. The same 10mM stock solutions were accurately dispensed in duplicate into 96-well plates and the DMSO dried down (MiVac GeneVac, 90 min, 37 ˚C). Thereafter, the samples were reconstituted (200 μM) in aqueous solution and shaken (20 hours, 25 ˚C). The solutions were analysed by means of HPLC-DAD (Agilent 1200 Rapid Resolution HPLC with a diode array detector). Solubility was then determined using the peak areas of the aqueous samples and the best fit calibration curves constructed using the calibration standards.[19]

17.2 LogD

The LogD assay was performed in triplicate using a shake-flask procedure. 10 mM stock solutions of each test compound were used to spike (100 μM) a 1:1 mixture of phosphate buffer (pH 7.4) and *n*-octanol. The solutions were shaken vigorously (1500 rpm) on an orbital shaker for 3 hours at room temperature. Thereafter the samples were centrifuged in order to fully separate the two immiscible fluids. The samples were analyzed by HPLC-DAD (Agilent 1200 Rapid Resolution HPLC with a diode array detector) and the amount of compound in the buffer and *n*-octanol were used to determine the partition coefficient, $LogD_{7.4}$.[20]

17.3 In vitro Microsomal stability

The *in vitro* microsomal stability assay was performed in duplicate in a 96-well micro titre plate. The test compounds (1 μM) were incubated individually in mouse, rat and pooled human liver microsomes (final protein concentration of 0.4 mg/mL; XenoTech, Lenexa, KS), suspended in 0.1M phosphate buffer (pH 7.4) for predetermined time points, in the presence and absence of the cofactor NADPH (1 mM). Reactions were quenched by adding 300 μL of ice-cold acetonitrile containing internal standard (carbamazepine, 0.0236 μg/mL). The samples were centrifuged and test in the supernatant were analyzed by means of LC-MS/MS (Agilent Rapid Resolution HPLC, AB SCIEX 4500 MS). The relative loss of parent compound over time was monitored and plots (concentration vs. time) were prepared per compound to determine the first order rate constant for compound depletion. This was in turn used to calculate half-life, *in vitro* intrinsic clearance and *in vivo* hepatic extraction ratio.[21]

17.4 Plasma protein binding (PPB)

Plasma protein binding was determined by ultracentrifugation. In brief, pooled human plasma was spiked with test compound (5 μM) from a 10mM DMSO stock. An aliquot was immediately removed and quenched using ice cold acetonitrile containing internal standard (carbamazepine, 0.0236 μg/mL), and placed in the freezer. This served as the total concentration sample. After pre-incubation (37 °C for 1 hour) duplicate aliquots of the spiked plasma were transferred to ultra-centrifugation tubes, and ultracentrifuged for 4 hours (42000 rpm, 37 °C, Beckman Optima L-80XP). The samples were then analysed by LC-MS/MS (Agilent Rapid Resolution HPLC, AB SCIEX 4500 QTRAP MS). Protein binding was then calculated by comparing analyte:peak area ratios of the ultracentrifuged sample to those of the total concentration sample.

17.5 Parallel artificial membrane permeability assay (PAMPA)

PAMPA method is used as an *in vitro* model of passive, trans- cellular permeability of drug-like compounds to screen for their oral absorption potential. Briefly, the PAMPA membrane filter is pre-coated with 5% hexadecane in hexane artificial lipid solution using a 96-well plate. Test compounds (1

mM) diluted with pre-warmed buffer (at desired pH) were added to the donor compartment of the PAMPA membrane filter, at pH's representative of the gastro- intestinal tract. The plates are incubated at room temperature for 4 hours with gentle shaking (40-50 rpm). Papp is determined by detecting the appearance of the test compound in the acceptor compartment (pH 7.4) of the artificial membrane plate following 4 hours of incubation. The samples are analyzed using LC-MS/MS and peak area ratios are used to calculate the apparent permeability (Cyprotex, in house method sheet).

18. Mouse pharmacokinetic studies

18.1 Ethics

Animal studies were conducted at the Holistic Drug Discovery and Development (H3D) Centre Animal Research Facility, University of Cape Town (UCT). Ethical approval was granted by the UCT Animal Ethics Committee prior to study commencement (ethics approval reference number 022/004), and all procedures were performed in accordance with UCT's animal ethics policies. Food and water were supplied ad libitum before and during the study.

18.2 Animal studies

Male BalbC mice were part of the Animal Unit located at the Division of Clinical Pharmacology, University of Cape Town, South Africa. The intravenous dose was administered as a bolus injection through the penile vein as solutions formulated in Dimethylacetamide/Polyethylene glycocl/Polypropylene Glycol (10:30:60). The oral dose was administered to 3 animals as an aqueous suspension containing 0.5% (w/v) hydroxypropyl methylcellulose and 0.2% (v/v) Tween 80. Mice were not fasted overnight and were permitted access *ad libitum* to water.

18.3 Sample analysis

Blood samples were collected from mice into heparinised microcentrifugation tubes at 0.17 (IV only), 0.5, 1, 3, 5, 8, 12, 24 hours after dosing and stored frozen (-80 ˚C) until analysis.

18.4 Bioanalytical method

The compound concentration was determined by LC-MS/MS. Samples were thawed and extracted by protein precipitation using acetonitrile containing an internal standard. The supernatant was then submitted for LC-MS/MS analysis. Calibration standards and quality controls prepared in drug-free whole mice blood were processed similarly. Elution of analytes was confirmed by multiple-reaction monitoring and compound concentrations were determined using the analyte response of the analytes

relative to the calibration curve. The accuracy, precision, and recovery for each compound were within acceptable limits.

18.5 Calculation of pharmacokinetic parameters

Pharmacokinetic parameters were calculated by non-compartmental analysis using PK Solutions 2.0 (Summit Research Services, Montrose, CO, USA) using a method based on curve stripping.

19. *In vivo* efficacy and pharmacokinetics in malaria-infected humanized mice

19.1 Ethics

Animal studies were conducted at the Holistic Drug Discovery and Development (H3D) Centre Animal Research Facility, University of Cape Town (UCT). Ethical approval was granted by the UCT Animal Ethics Committee prior to study commencement (ethics approval reference number 021/015), and all procedures were performed in accordance with UCT's animal ethics policies. Food and water were supplied ad libitum before and during the study.

19.2 P. falciparum infection of humanized mice

The antimalarial activity of **18** was determined in the *P. falciparum*-infected NSG mouse model, in 6- to 10-week-old, male NSG mice, weighing between 25 and 30 g, using methods previously described.[22,23] Briefly, NSG mice were intravenously engrafted daily with prepared human erythrocytes for 10 days, then the mice were intravenously injected in the tail vein with 2 x 10^7 asynchronous *Pf*3D7$^{0087/N9}$-infected human erythrocytes (day 0). *Pf*3D7$^{0087/N9}$ is a chloroquine-sensitive strain that was developed and selected for infection in NSG mice at GlaxoSmithKline, Tres Cantos, Spain. The infection was left to establish for 3 days before commencement of treatment on day 3. The percentage of human erythrocytes was maintained above 50% with daily engraftments until the experimental endpoint on day 7 after infection.

19.3 Administration and blood sampling

Compound **18** was administered as a single dose as either 100, 50, 30, 10 or 3 mg/kg, starting on the third day after infection with *P. falciparum.* Whole-blood PK and efficacy samples were collected via tail vein bleeding into lithium heparin-coated tubes. PK blood samples were collected for each dosage group at 0.5, 1, 2, 4, 6, 24, 48, 72 and 96 h after administration on day 3. PK samples were stored at -80˚C until bioanalysis. Efficacy blood samples were collected before treatment for all experimental groups on days 3, 4, 5, 6, and 7. These samples were processed immediately after collection, and the percentage of infected human erythrocytes, or the parasitemia, and the percentage engraftment

measurements were determined by fluorescence-activated cell sorting using an Accuri C6 Plus flow cytometer and FlowJo 10.8 software (Becton, Dickinson and Company), as previously described.[24] The relevant pharmacokinetic parameters for the oral groups are presented in Table S2. The efficacy result and the whole blood profiles obtained for the oral groups are graphically presented in Fig. 4 in the main manuscript.[23] To determine the ED_{90} and $AUCED_{90}$ values of **18** non-linear fitting to a sigmoid dose-response curve of log10 of % parasitemia on day 7 following infection versus the dose and $AUC_{(0-96)}$ was used, respectively. Compound **18** showed an ED_{90} value of 4.6 mg/kg. The exposure levels responsible for the ED_{90} was 22.7 µM.h.

19.4 Pharmacokinetic–pharmacodynamic modeling. Nonlinear mixed effects modeling, in Monolix

2021R1 software (Lixoft), was used to develop a sequential PK-PD model for the total whole-blood concentration-time data for **18** after the efficacy study in the *P. falciparum*-infected NSG mouse model. Concentration values that were below the lower limit of quantification (LLOQ) of 2 ng/mL were censored in the PK analysis. The model parameters were estimated using stochastic approximation expectation maximization algorithm.[25] A one compartment model with first-order absorption and elimination was used to describe the oral PK of **18**. The rate of change in parasitemia was described by the direct effect pharmacodynamic model, which was sequentially modelled with population pharmacokinetic data. The compound EC_{50} was 7.50 ng/mL with a wide variation in the kill rate (Kkill) of parasites and the steepness (H) of the pharmacodynamic curve.

20. HPLC traces and NMR spectra of final compounds

- ^{1}H NMR of Compound **5**

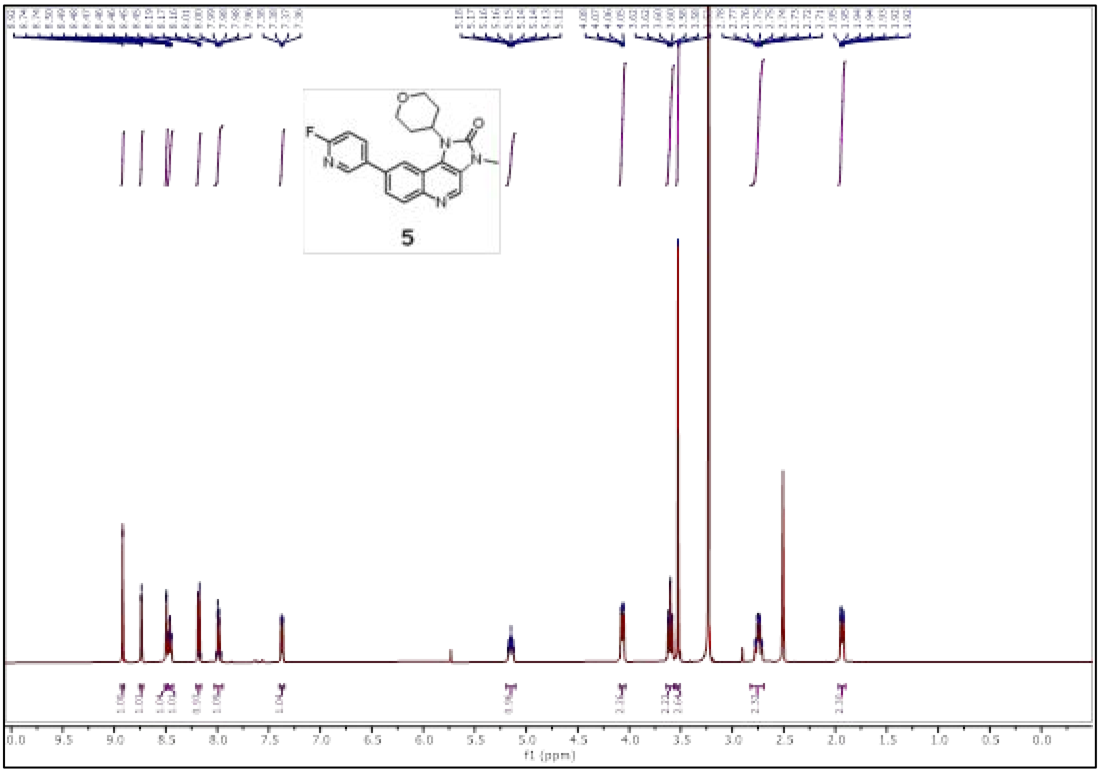

- HPLC trace of Compound **5**

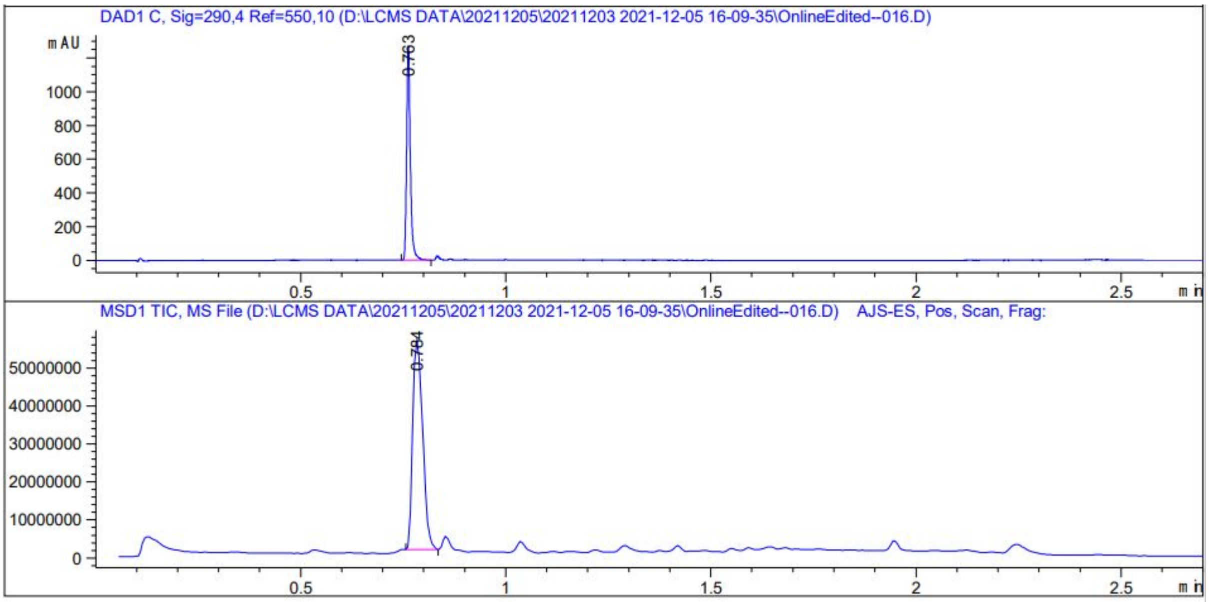

Peak #	RetTime [min]	Type	Width [min]	Area [mAU*s]	Height [mAU]	Area %
1	0.763	BB	9.85e-3	795.10529	1262.65796	100.0000
Totals :				795.10529	1262.65796	

MS Spectrum

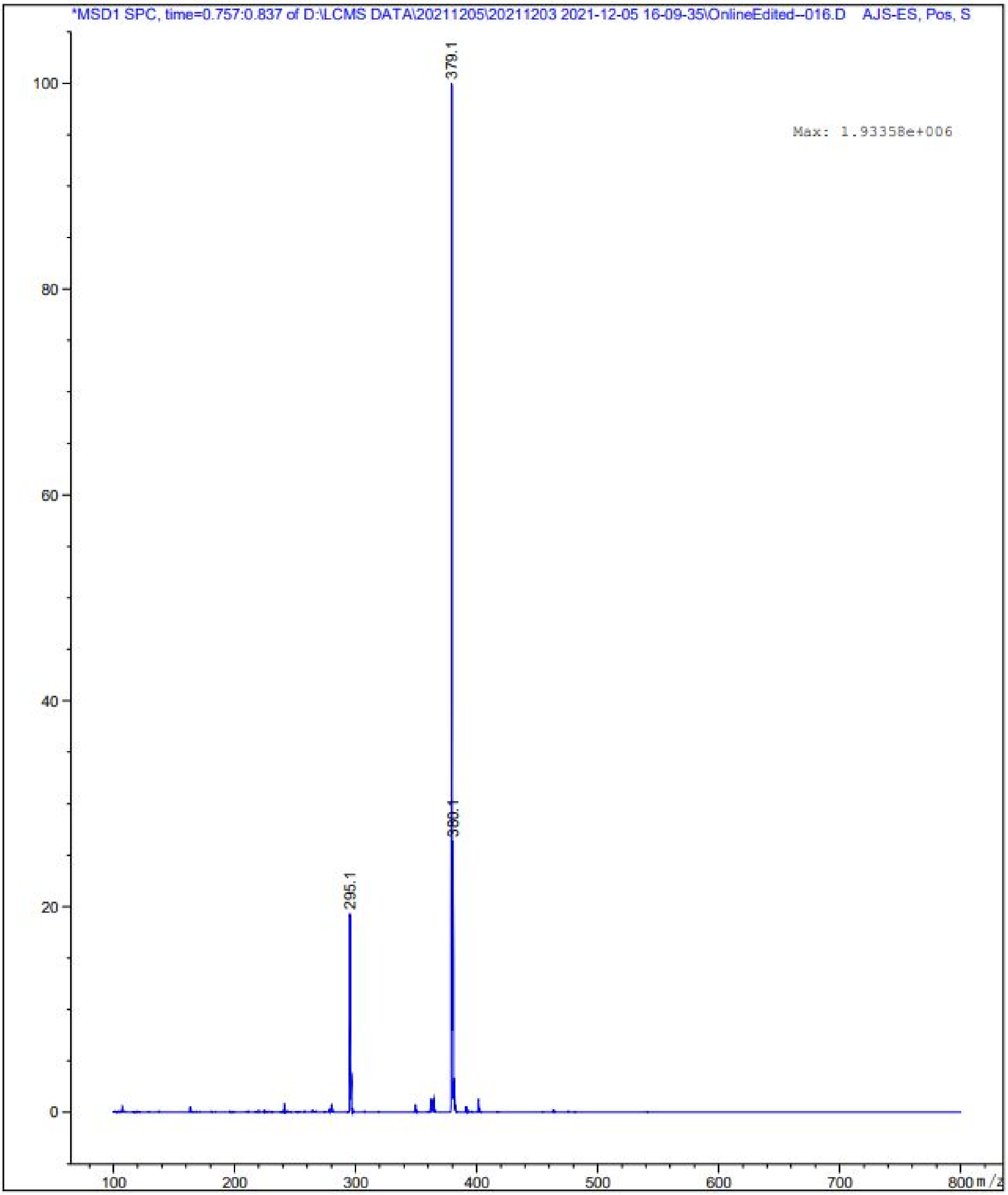
*MSD1 SPC, time=0.757:0.837 of D:\LCMS DATA\20211205\20211203 2021-12-05 16-09-35\OnlineEdited--016.D AJS-ES, Pos, S
Max: 1.93358e+006
379.1
380.1
295.1
100
80
60
40
20
0
100
200
300
400
500
600
700
800
m/z

- ^{1}H NMR spectrum of Compound **6**

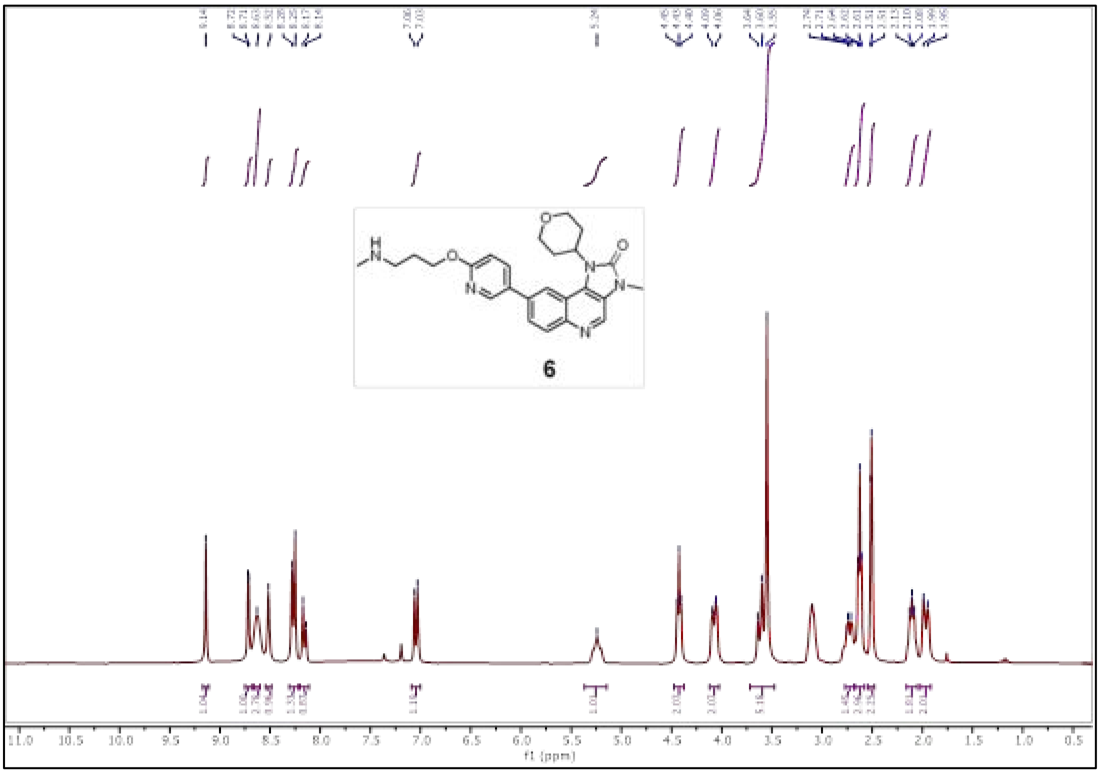

- ^{13}C NMR spectrum of Compound **6**

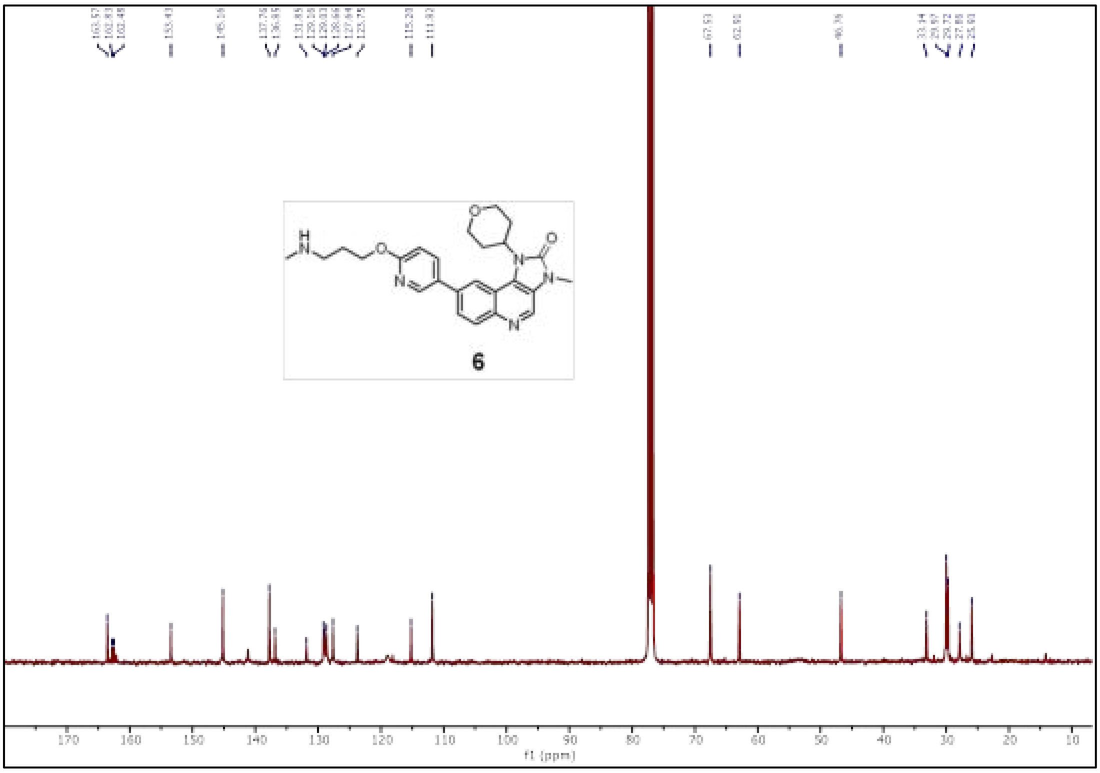

- HPLC trace of Compound **6**

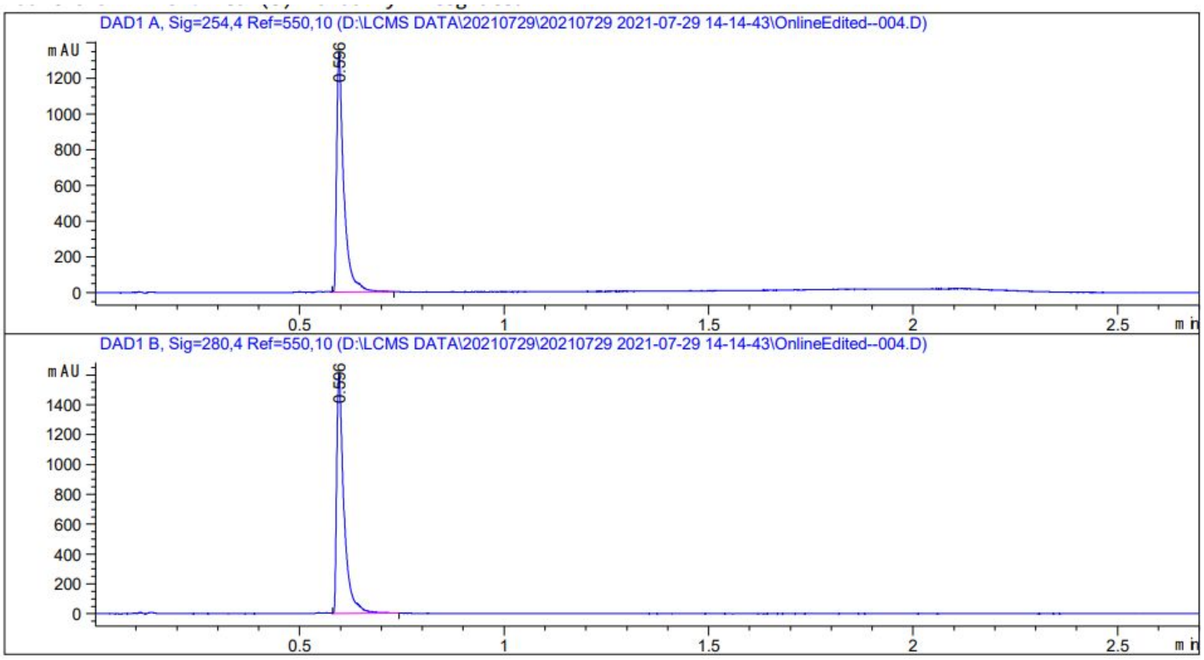

Peak #	RetTime [min]	Type	Width [min]	Area [mAU*s]	Height [mAU]	Area %
1	0.596	BB	0.0187	2009.92700	1599.10803	100.0000
Totals :				2009.92700	1599.10803	

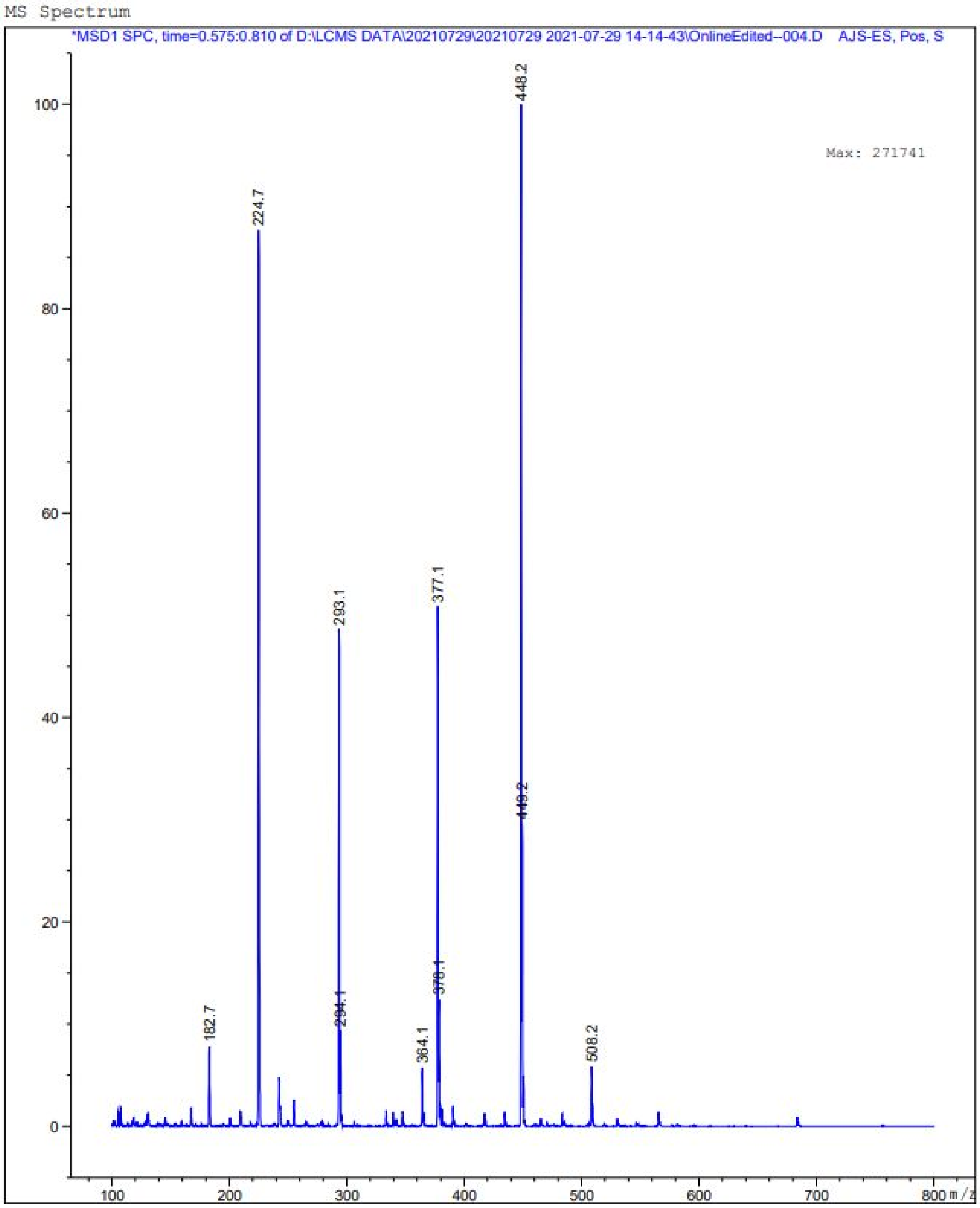
MS Spectrum
*MSD1 SPC, time=0.575:0.810 of D:\LCMS DATA\20210729\20210729 2021-07-29 14-14-43\OnlineEdited--004.D AJS-ES, Pos, S
Max: 271741
448.2
224.7
377.1
293.1
449.2
378.1
294.1
182.7
364.1
508.2
m/z

- ^{1}H NMR spectrum of Compound **7**

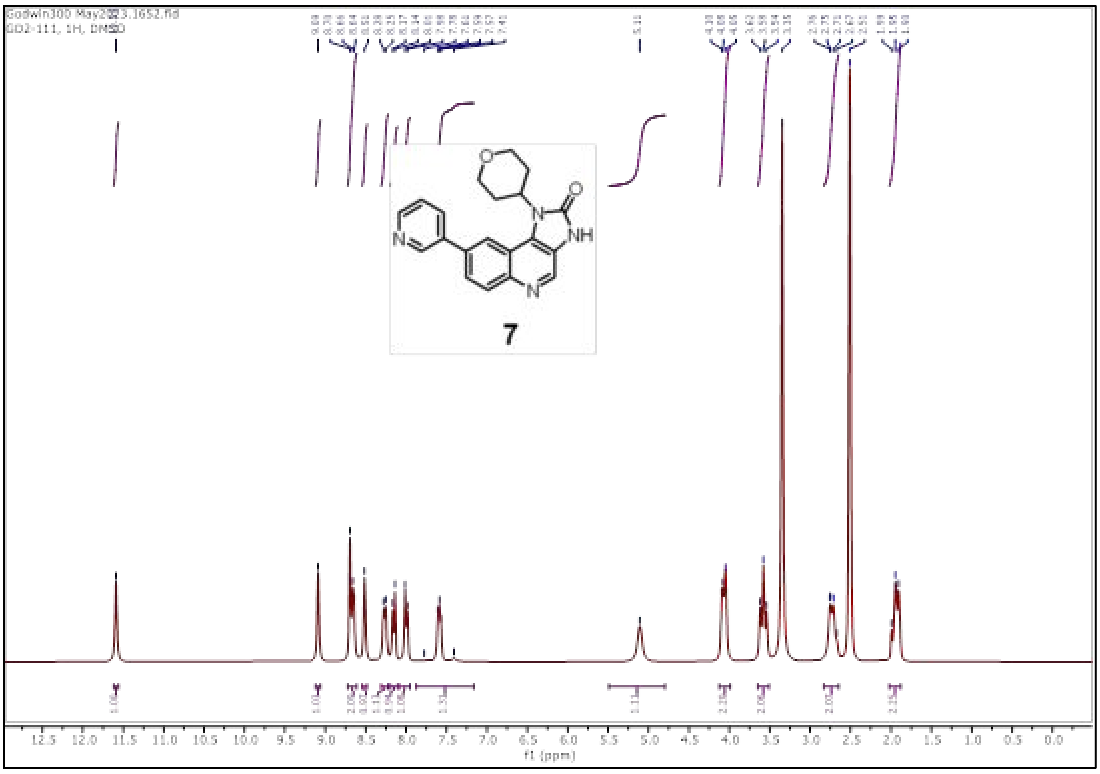

- HPLC trace of Compound **7**

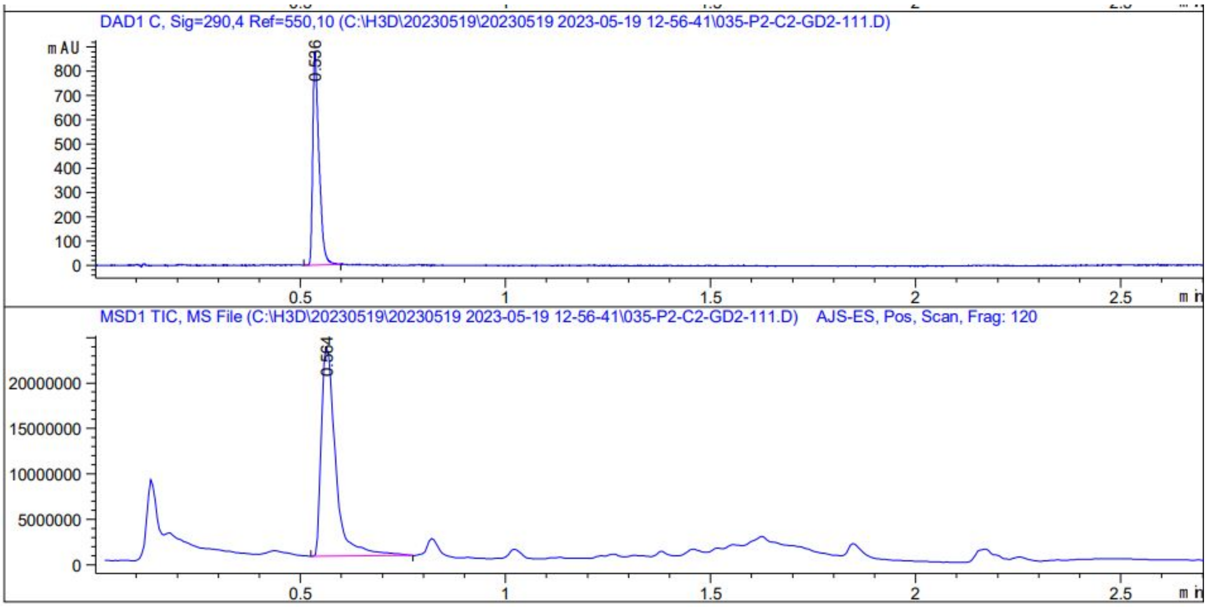

Peak #	RetTime [min]	Type	Width [min]	Area [mAU*s]	Height [mAU]	Area %
1	0.536	BB	0.0160	938.64331	877.14551	100.0000

Totals : 938.64331 877.14551

MS Spectrum

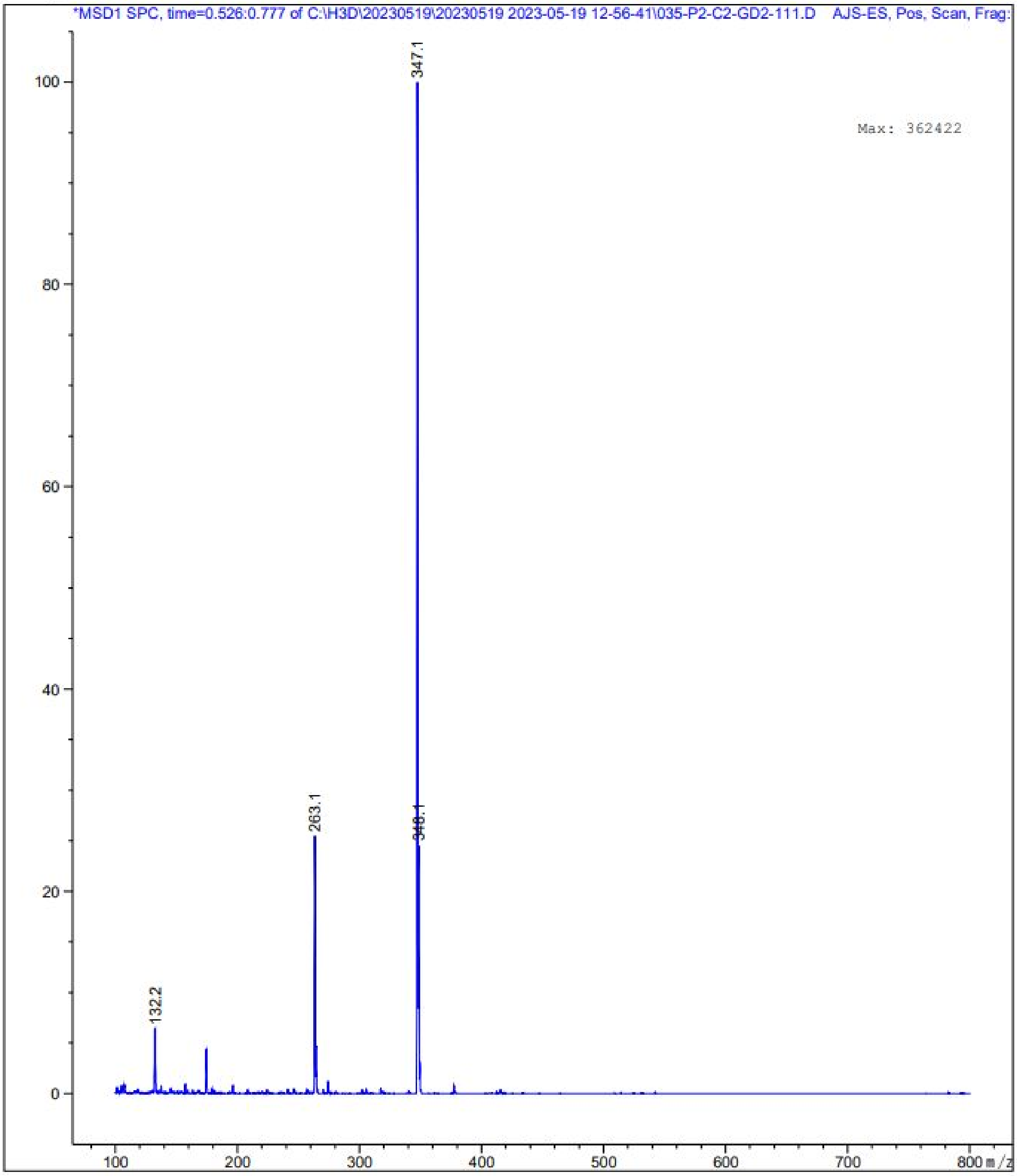

*MSD1 SPC, time=0.526:0.777 of C:\H3D\20230519\20230519 2023-05-19 12-56-41\035-P2-C2-GD2-111.D AJS-ES, Pos, Scan, Frag:
Max: 362422
347.1
263.1
348.1
132.2
100
80
60
40
20
0
100
200
300
400
500
600
700
800
m/z

- ^{1}H NMR spectrum of Compound **8**

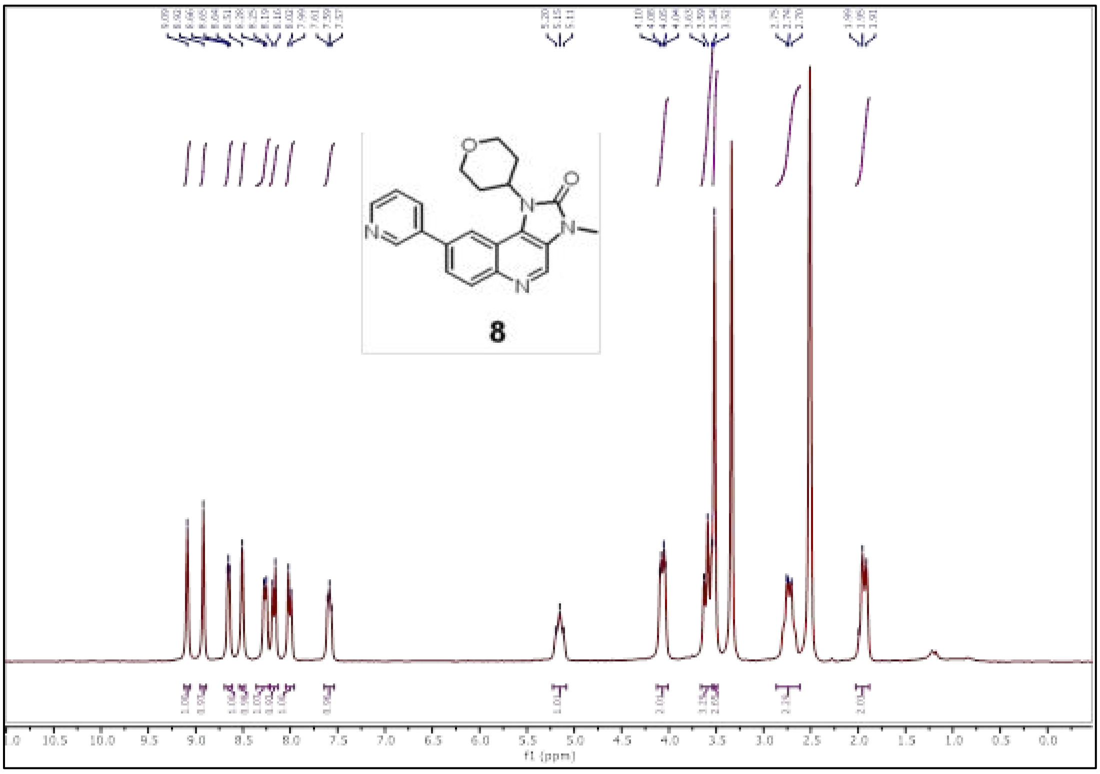

- HPLC trace of Compound **8**

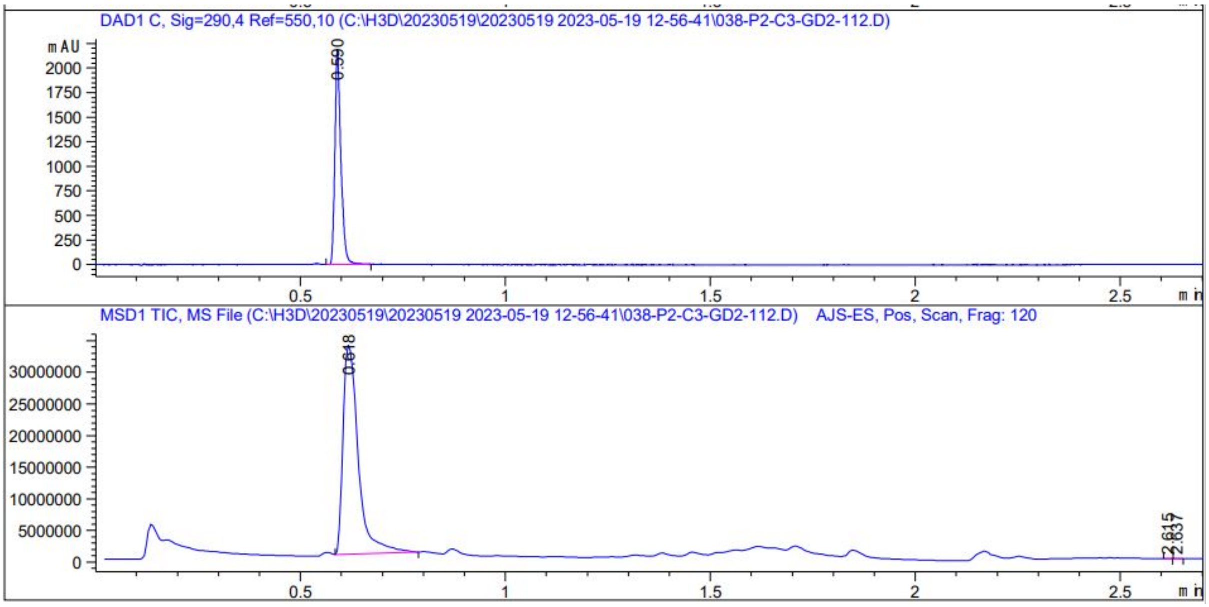

Peak #	RetTime [min]	Type	Width [min]	Area [mAU*s]	Height [mAU]	Area %
1	0.787	BB	0.0138	2377.77368	2682.21875	100.0000
Totals :				2377.77368	2682.21875	

MS Spectrum

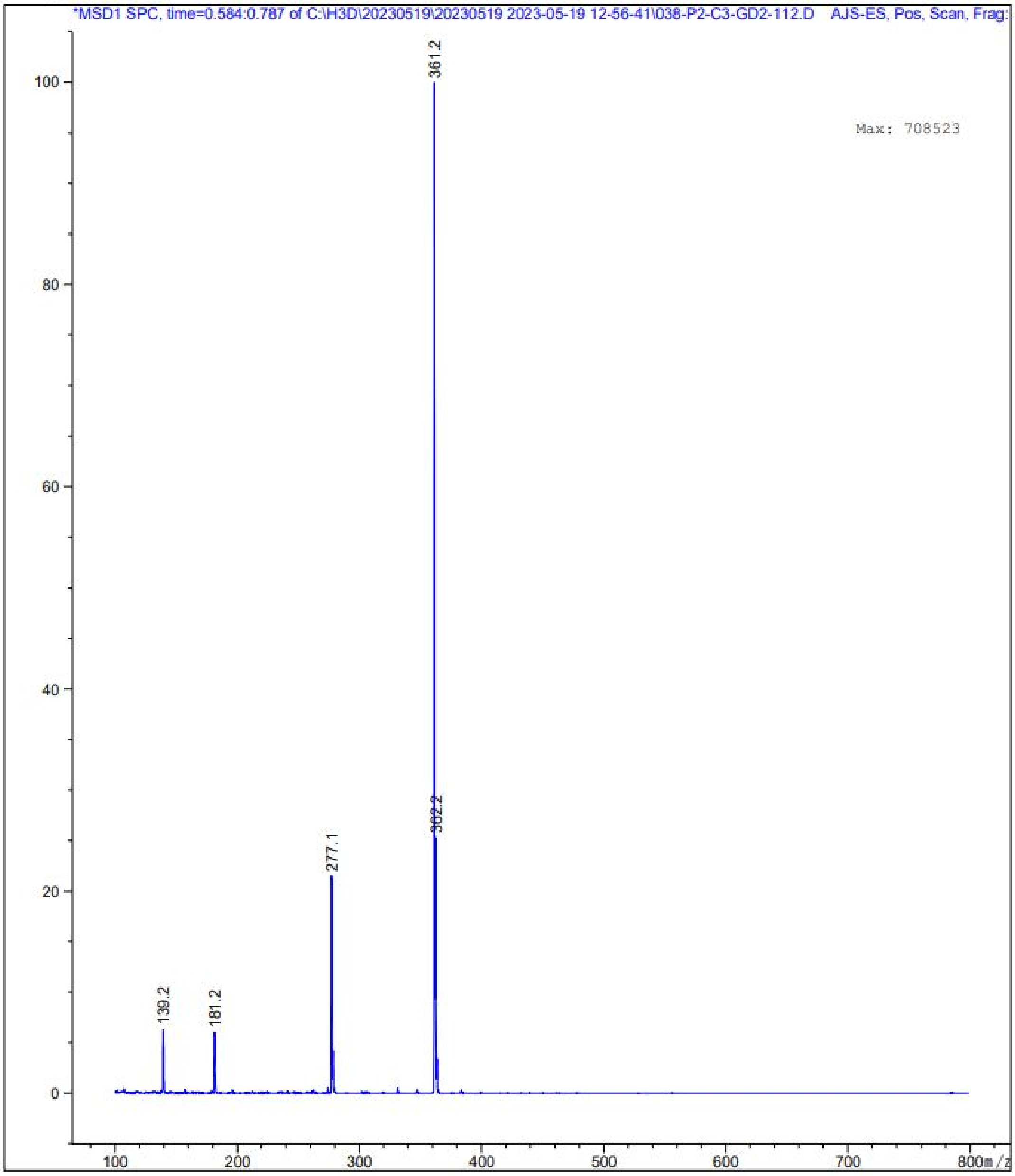

*MSD1 SPC, time=0.584:0.787 of C:\H3D\20230519\20230519 2023-05-19 12-56-41\038-P2-C3-GD2-112.D AJS-ES, Pos, Scan, Frag:
Max: 708523
361.2
362.2
277.1
139.2
181.2
100
80
60
40
20
0
100
200
300
400
500
600
700
800m/z

- ^{1}H NMR spectrum of Compound **9**

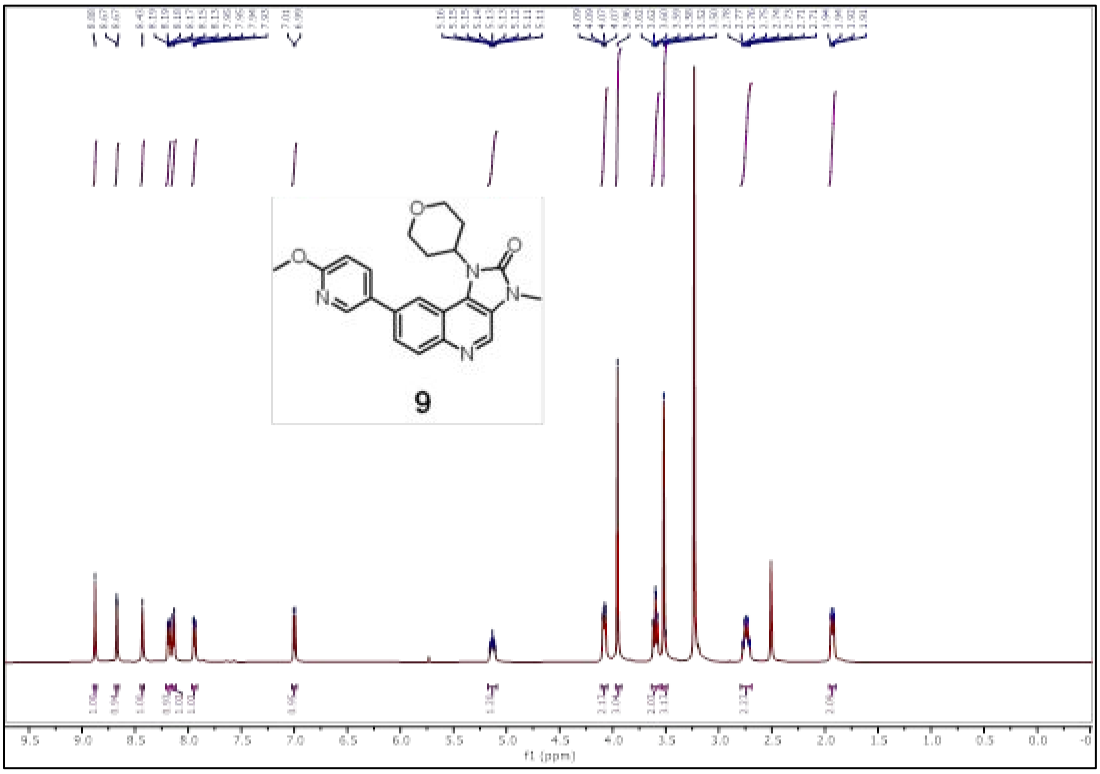

- ^{13}C NMR spectrum of Compound **9**

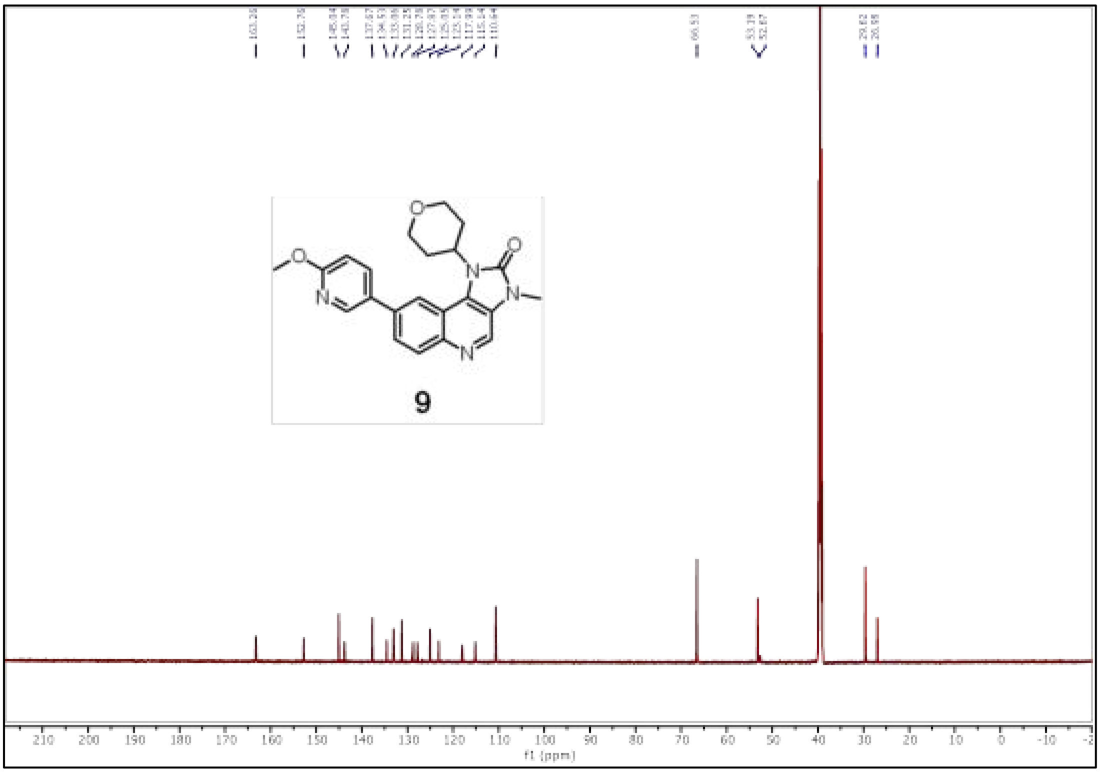

- HPLC trace of Compound **9**

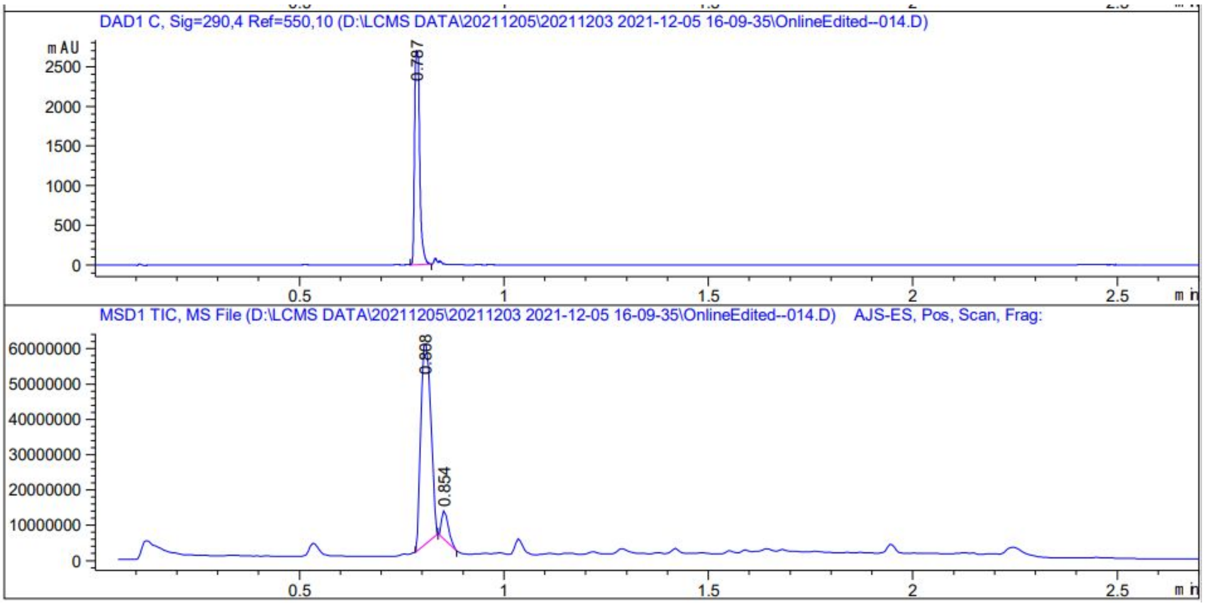

Peak #	RetTime [min]	Type	Width [min]	Area [mAU*s]	Height [mAU]	Area %
1	0.750	BB	0.0109	1486.61707	2191.57373	95.5239
2	0.834	BB	9.45e-3	69.66019	109.06633	4.4761
Totals :				1556.27725	2300.64006	

MS Spectrum

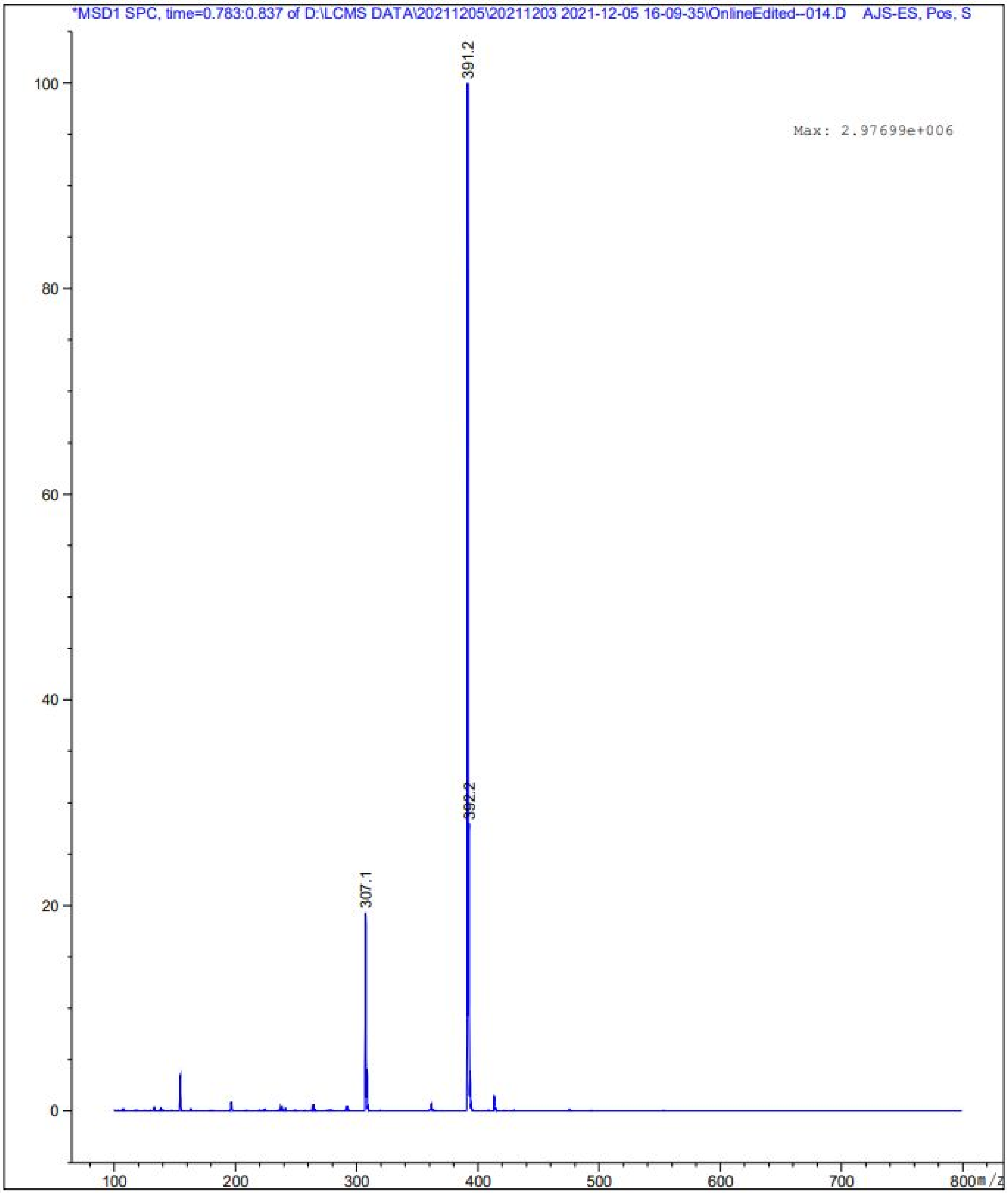
*MSD1 SPC, time=0.783:0.837 of D:\LCMS DATA\20211205\20211203 2021-12-05 16-09-35\OnlineEdited--014.D AJS-ES, Pos, S
Max: 2.97699e+006
391.2
392.2
307.1
100
80
60
40
20
0
100
200
300
400
500
600
700
800
m/z

- ^{1}H NMR spectrum of Compound **10**

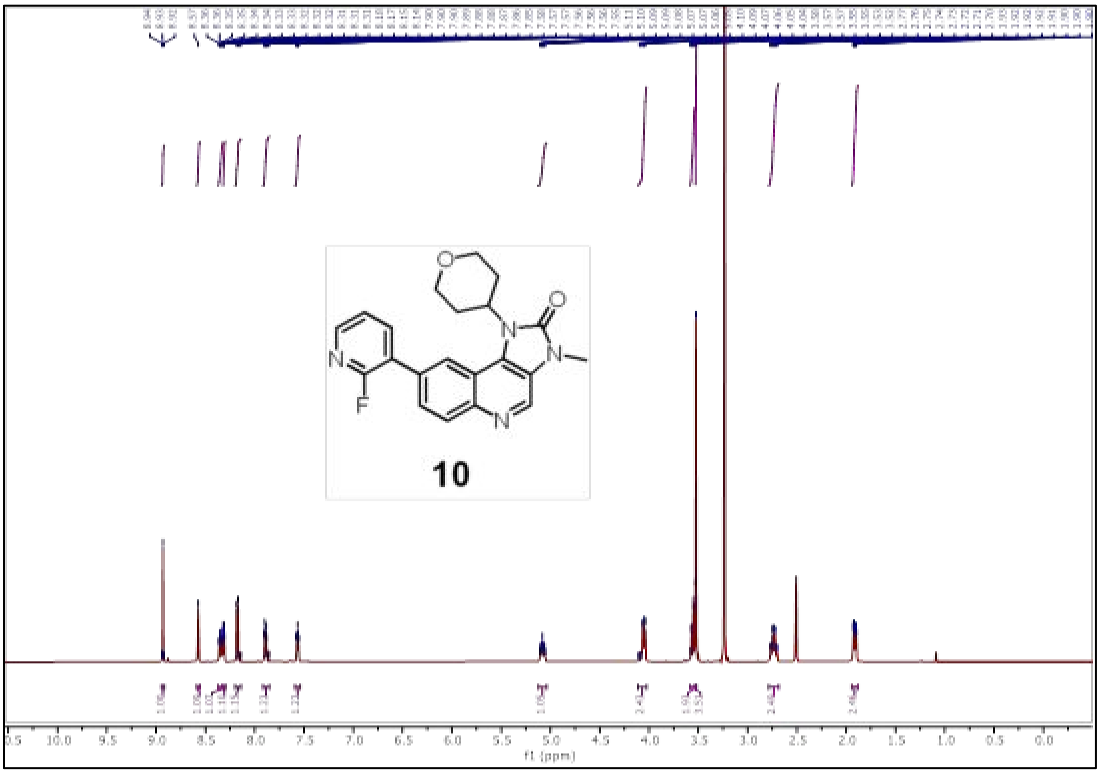

- ^{13}C NMR spectrum of Compound **10**

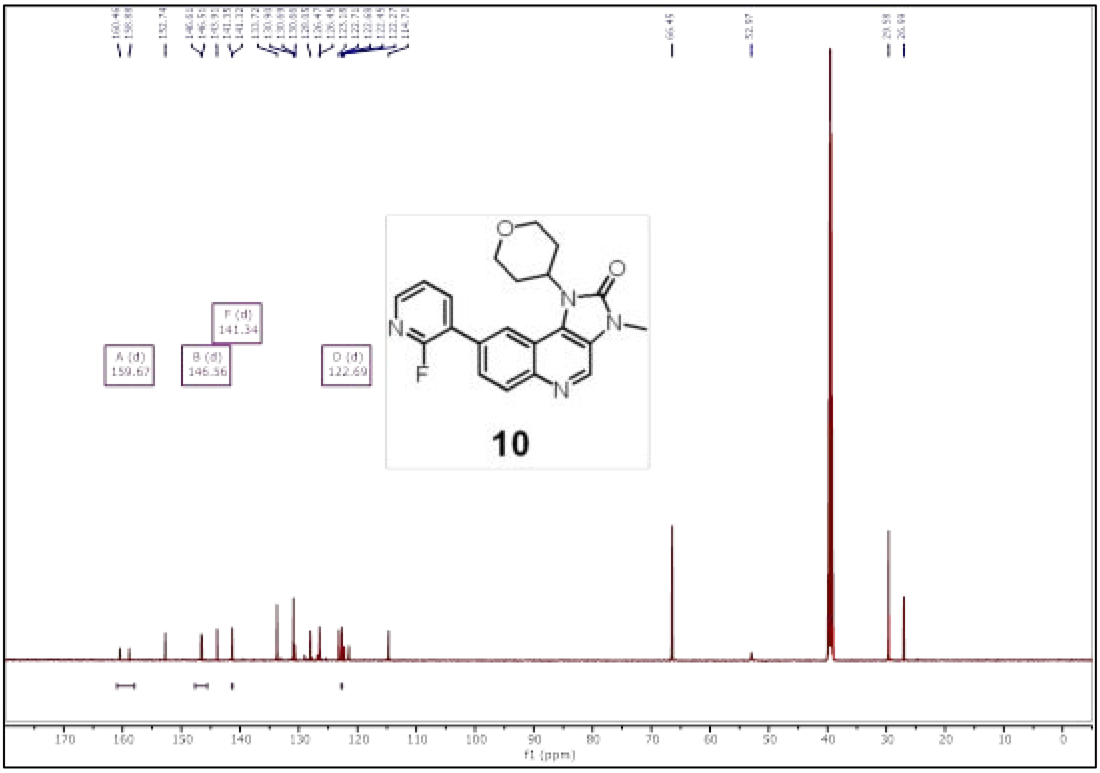

- HPLC trace of Compound **10**

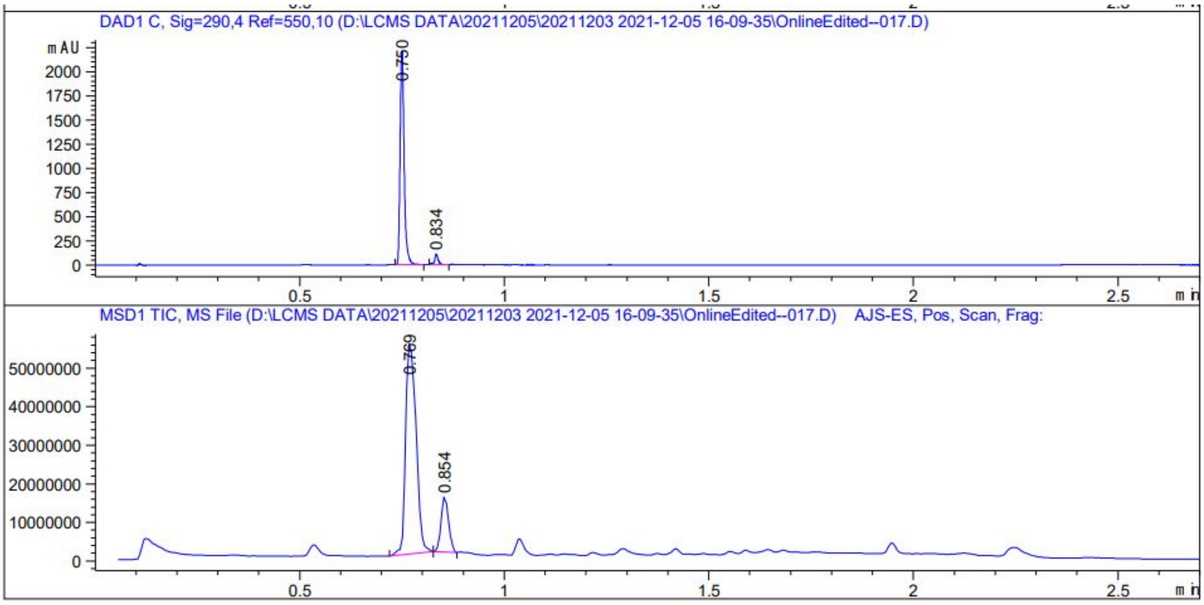

Peak #	RetTime [min]	Type	Width [min]	Area [mAU*s]	Height [mAU]	Area %
1	0.750	BB	0.0109	1486.61707	2191.57373	95.5239
2	0.834	BB	9.45e-3	69.66019	109.06633	4.4761
Totals :				1556.27725	2300.64006	

MS Spectrum

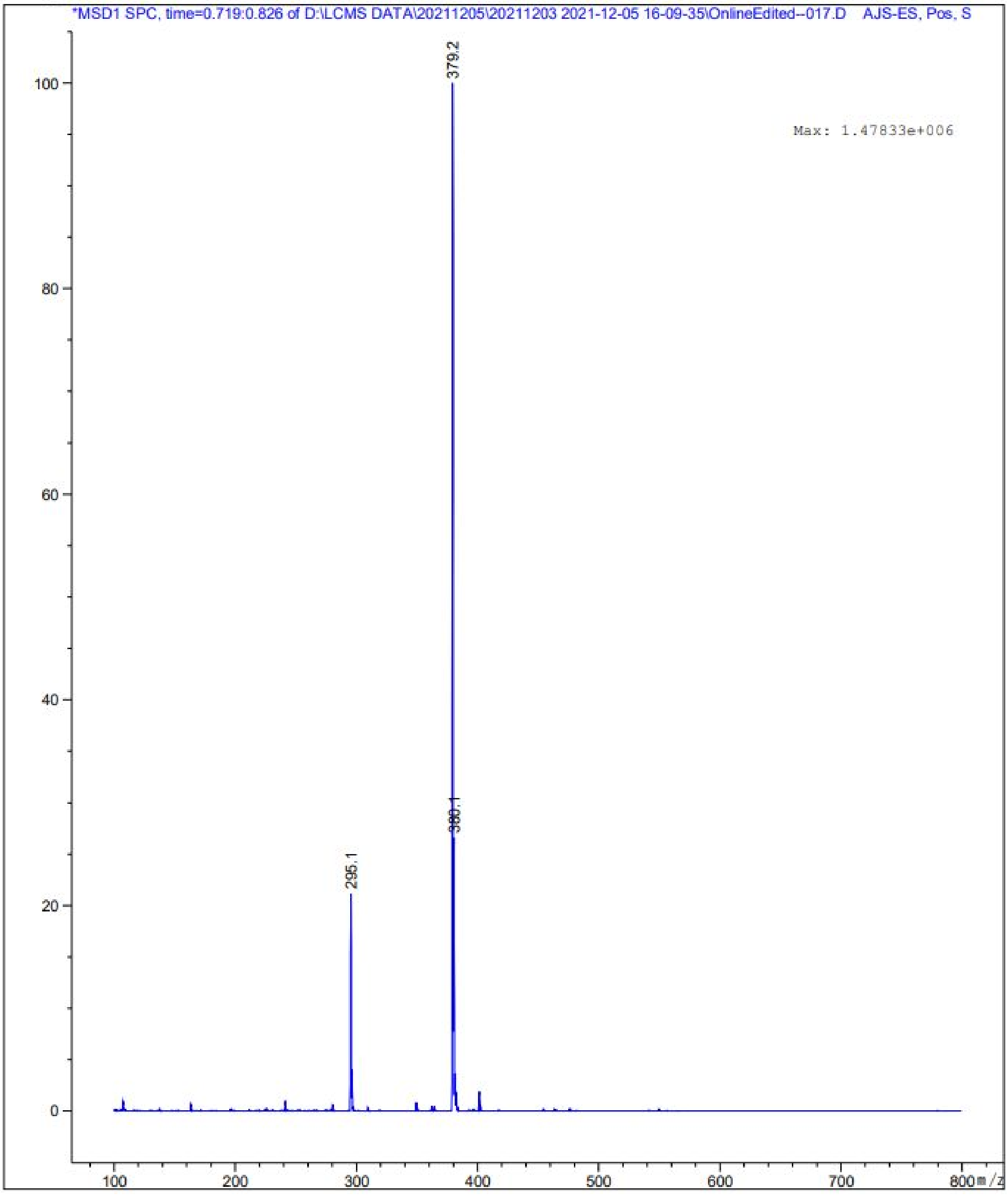

*MSD1 SPC, time=0.719:0.826 of D:\LCMS DATA\20211205\20211203 2021-12-05 16-09-35\OnlineEdited--017.D AJS-ES, Pos, S
Max: 1.47833e+006
379.2
380.1
295.1
100
80
60
40
20
0
100
200
300
400
500
600
700
800
m/z

- ^{1}H NMR spectrum of Compound **11**

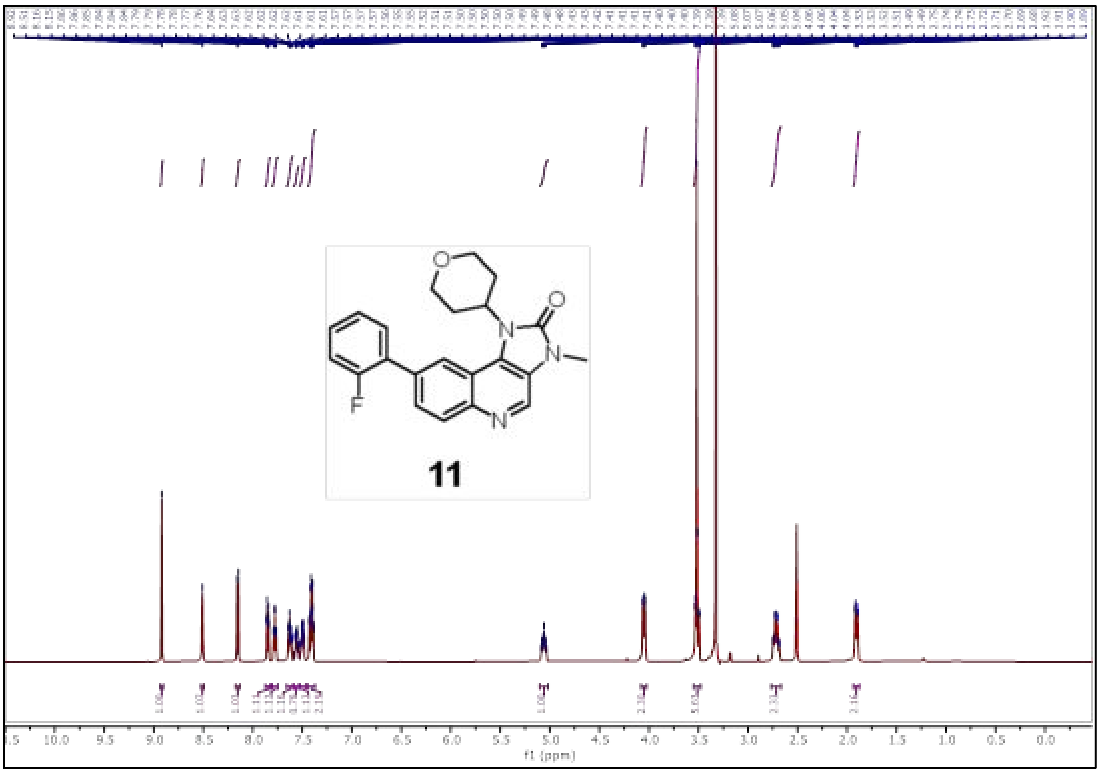

- ^{13}C NMR spectrum of Compound **11**

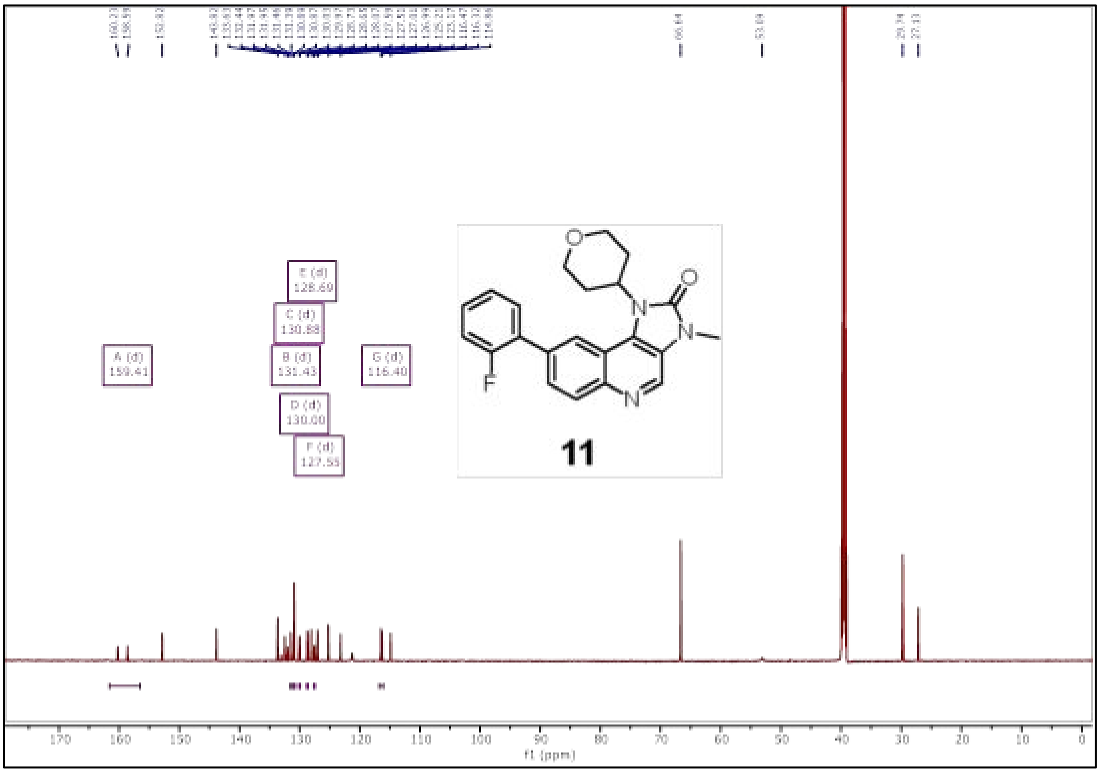

- HPLC trace of Compound **11**

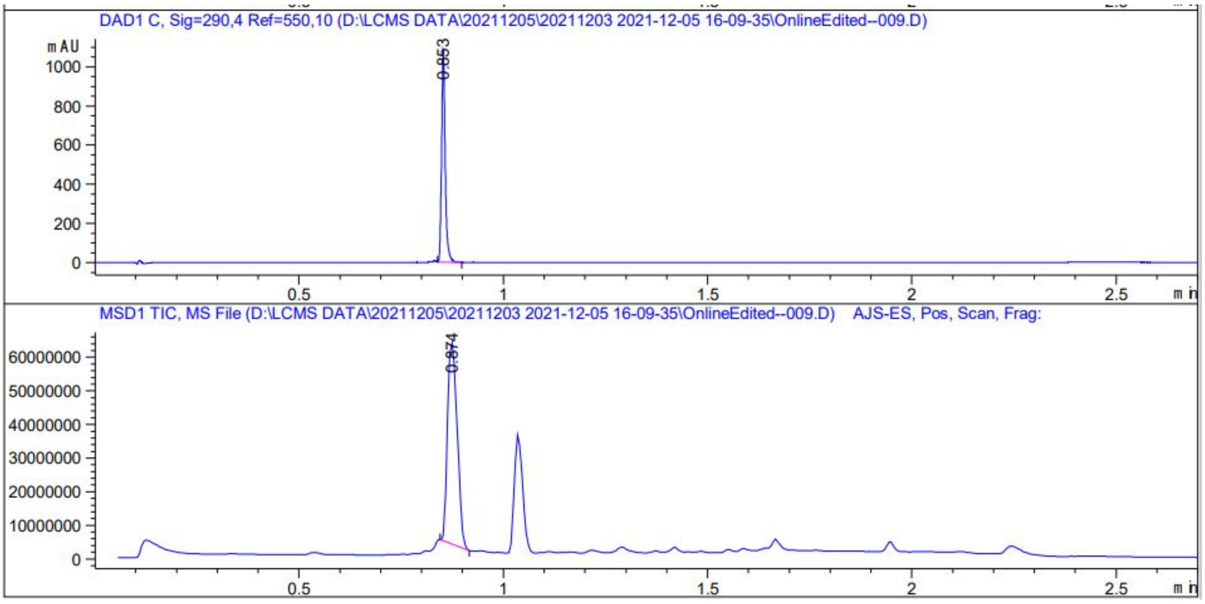

Peak #	RetTime [min]	Type	Width [min]	Area [mAU*s]	Height [mAU]	Area %
1	0.853	BB	9.69e-3	663.52264	1076.37683	100.0000
Totals :				663.52264	1076.37683	

MS Spectrum

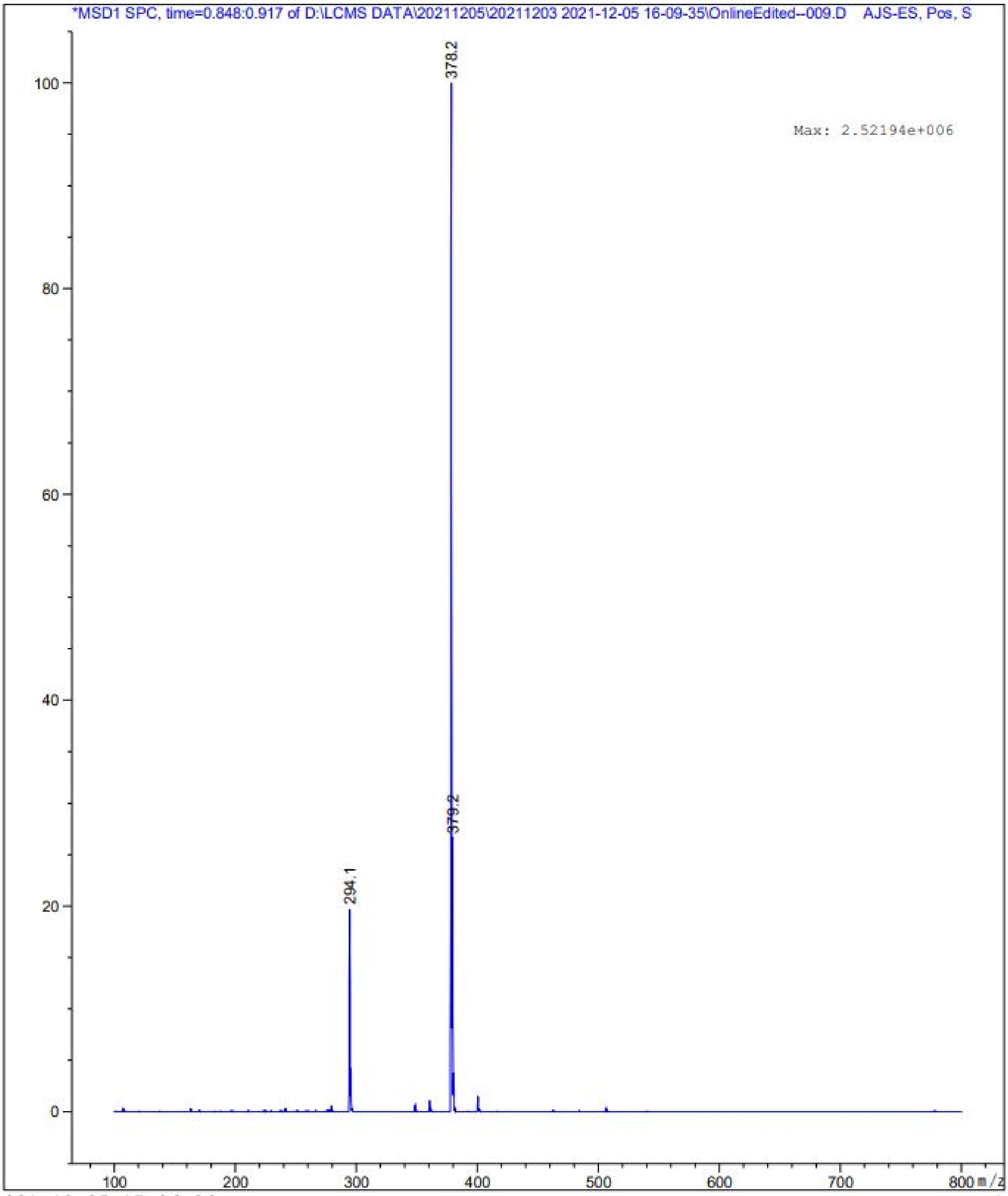

*MSD1 SPC, time=0.848:0.917 of D:\LCMS DATA\20211205\20211203 2021-12-05 16-09-35\OnlineEdited--009.D AJS-ES, Pos, S
Max: 2.52194e+006
378.2
379.2
294.1
100
80
60
40
20
0
100
200
300
400
500
600
700
800
m/z

- ^{1}H NMR spectrum of Compound **12**

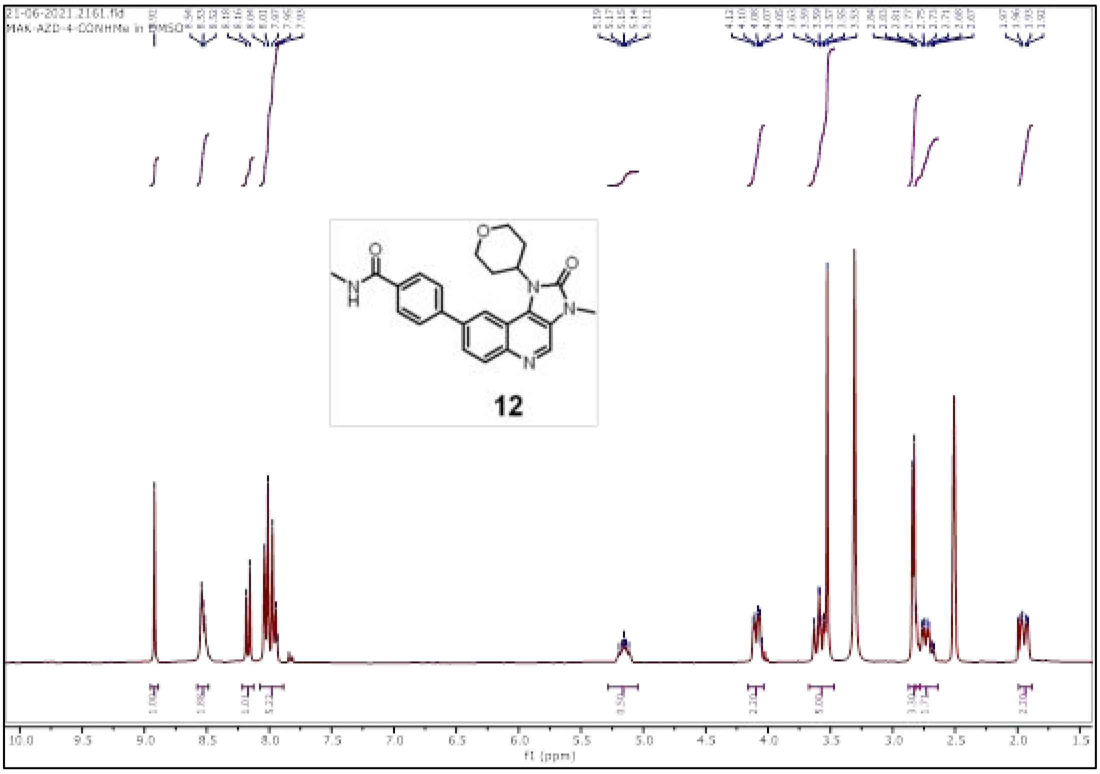

- HPLC trace of Compound **12**

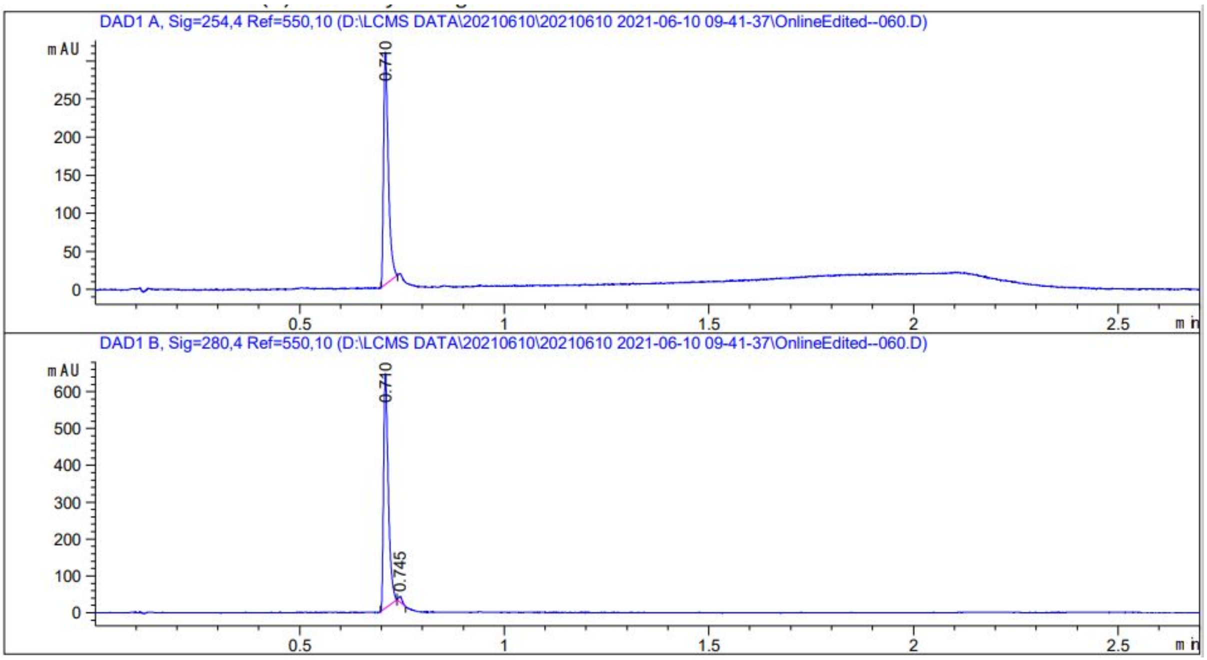

Peak #	RetTime [min]	Type	Width [min]	Area [mAU*s]	Height [mAU]	Area %
1	0.710	BB	0.0123	508.23392	634.58856	98.1965
2	0.745	BB	0.0106	9.33450	14.24004	1.8035
Totals :				517.56842	648.82861	

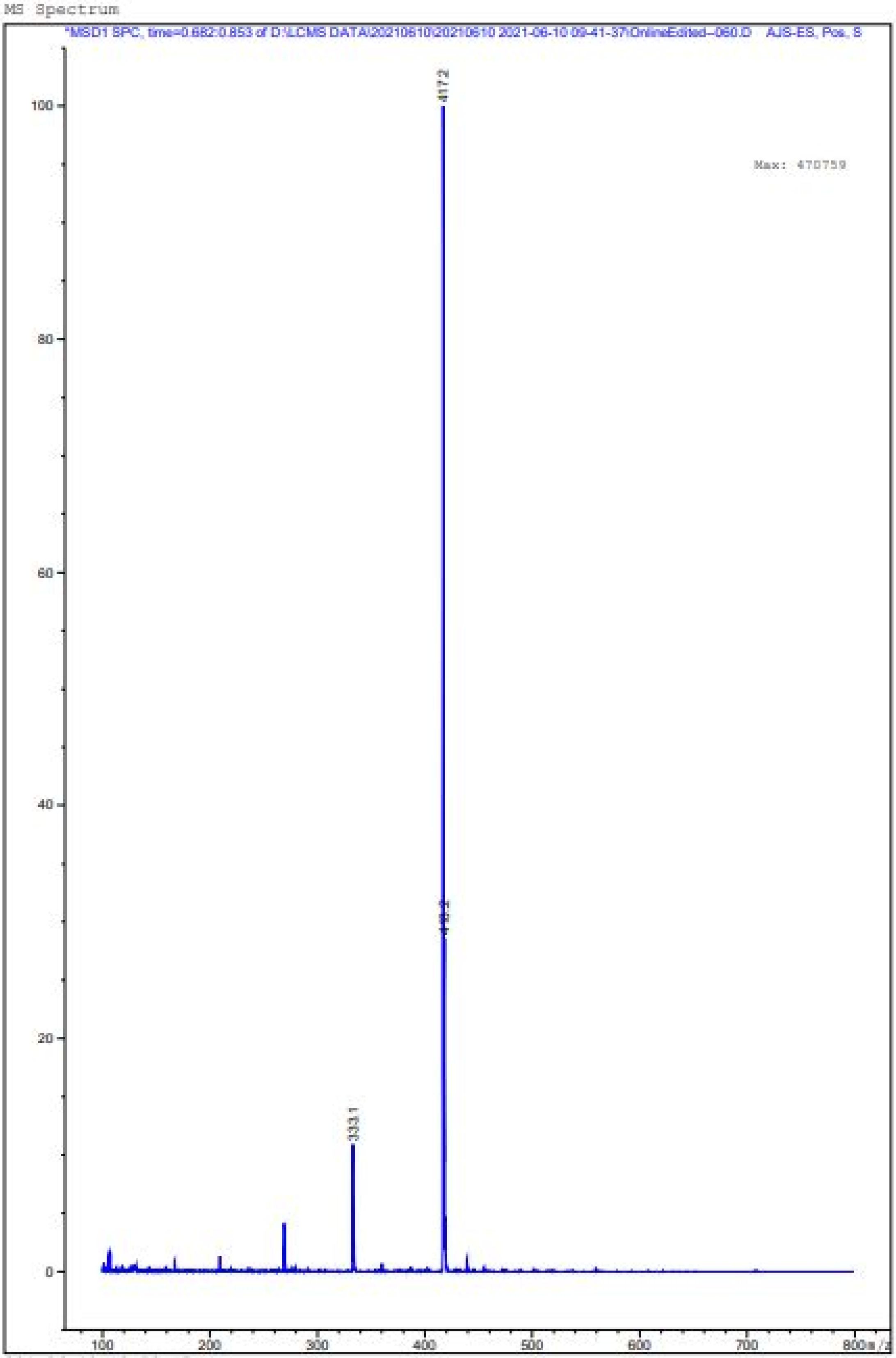
MS Spectrum
*MSD1 SPC, time=0.682:0.853 of D:\LCMS DATA\20210610\20210610 2021-06-10 09-41-37\OnlineEdited--060.D AJS-ES, Pos, S
Max: 470759
417.2
333.1
100
80
60
40
20
0
100
200
300
400
500
600
700
800m/z

- ^{1}H NMR spectrum of Compound **13**

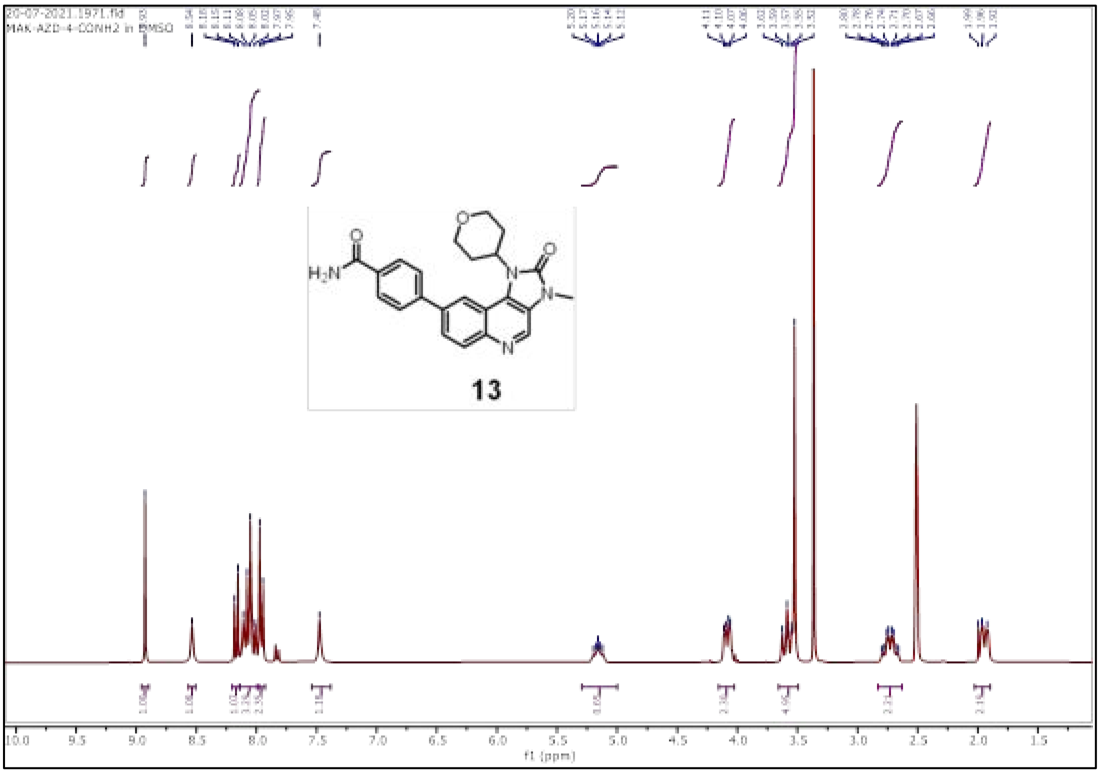

- HPLC trace of Compound **13**

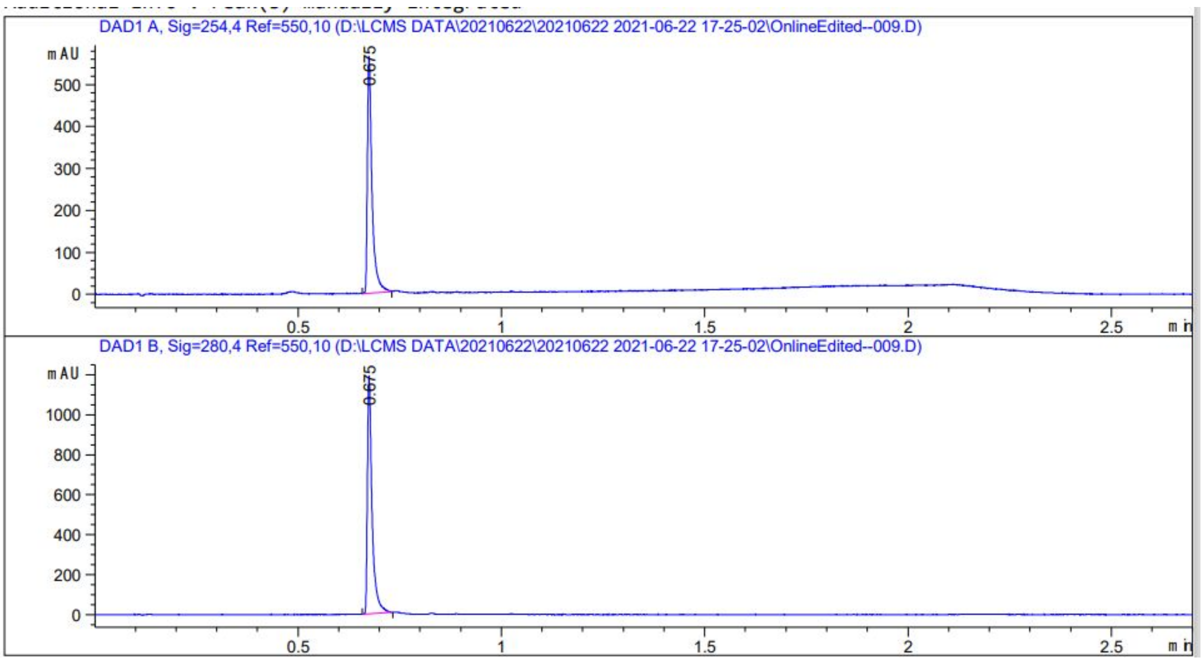

Peak #	RetTime [min]	Type	Width [min]	Area [mAU*s]	Height [mAU]	Area %
1	0.675	BB	0.0119	959.28656	1187.71875	100.0000
Totals :				959.28656	1187.71875	

MS Spectrum

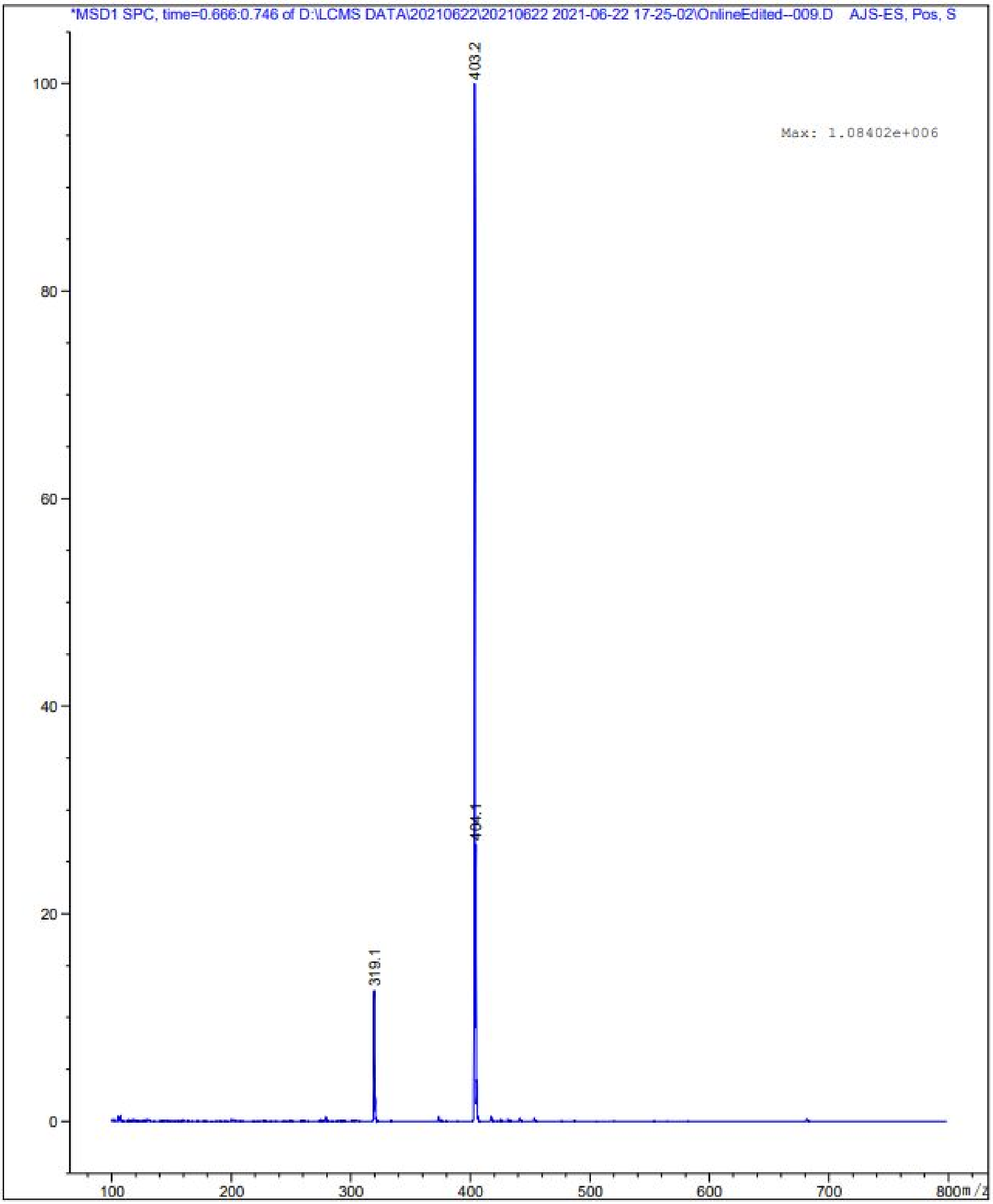

*MSD1 SPC, time=0.666:0.746 of D:\LCMS DATA\20210622\20210622 2021-06-22 17-25-02\OnlineEdited--009.D AJS-ES, Pos, S
Max: 1.08402e+006
403.2
404.1
319.1
100
80
60
40
20
0
100
200
300
400
500
600
700
800
m/z

- ^{1}H NMR spectrum of Compound **14**

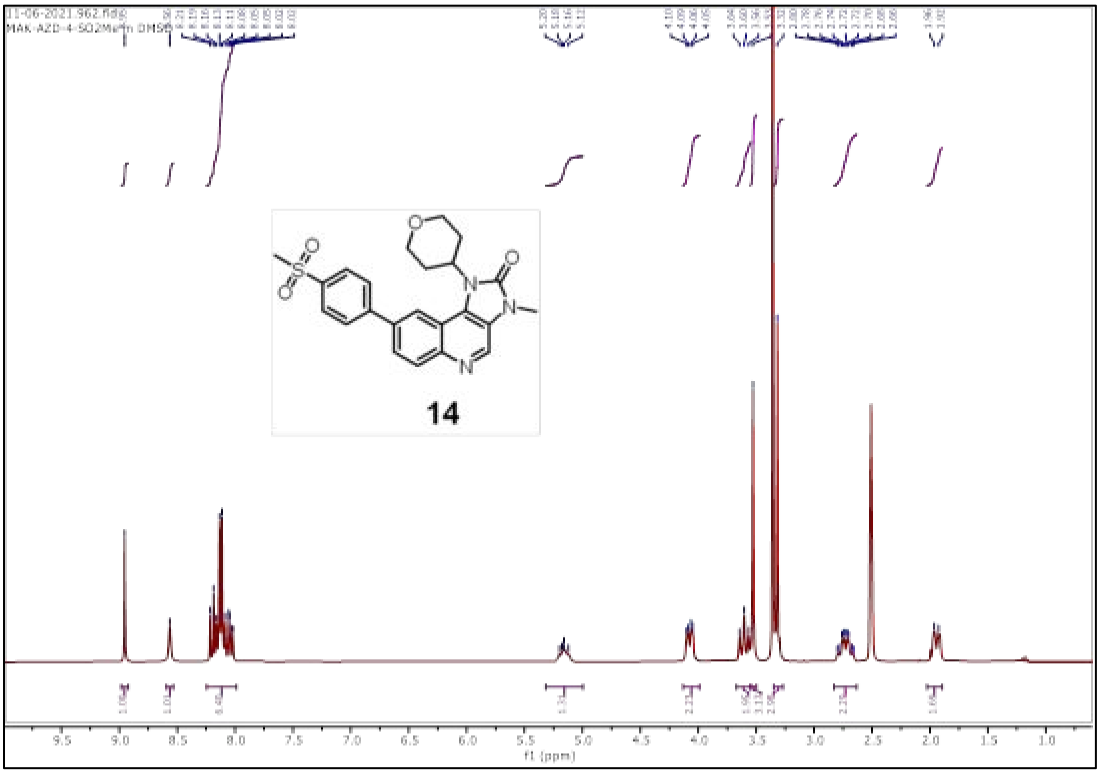

- HPLC trace of Compound **14**

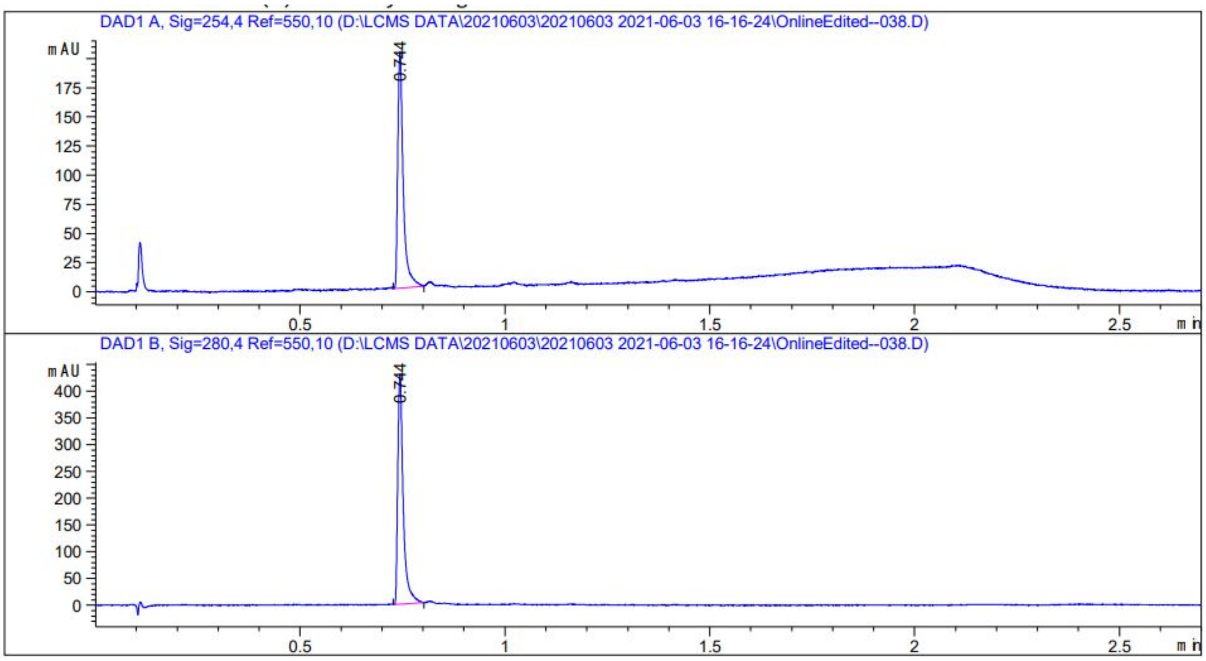

```
Peak RetTime Type  Width     Area      Height     Area
  #   [min]        [min]   [mAU*s]     [mAU]       %
----|-------|----|-------|----------|----------|--------|
   1   0.744 BB    0.0138  394.16068  427.07419 100.0000

Totals :                   394.16068  427.07419
```

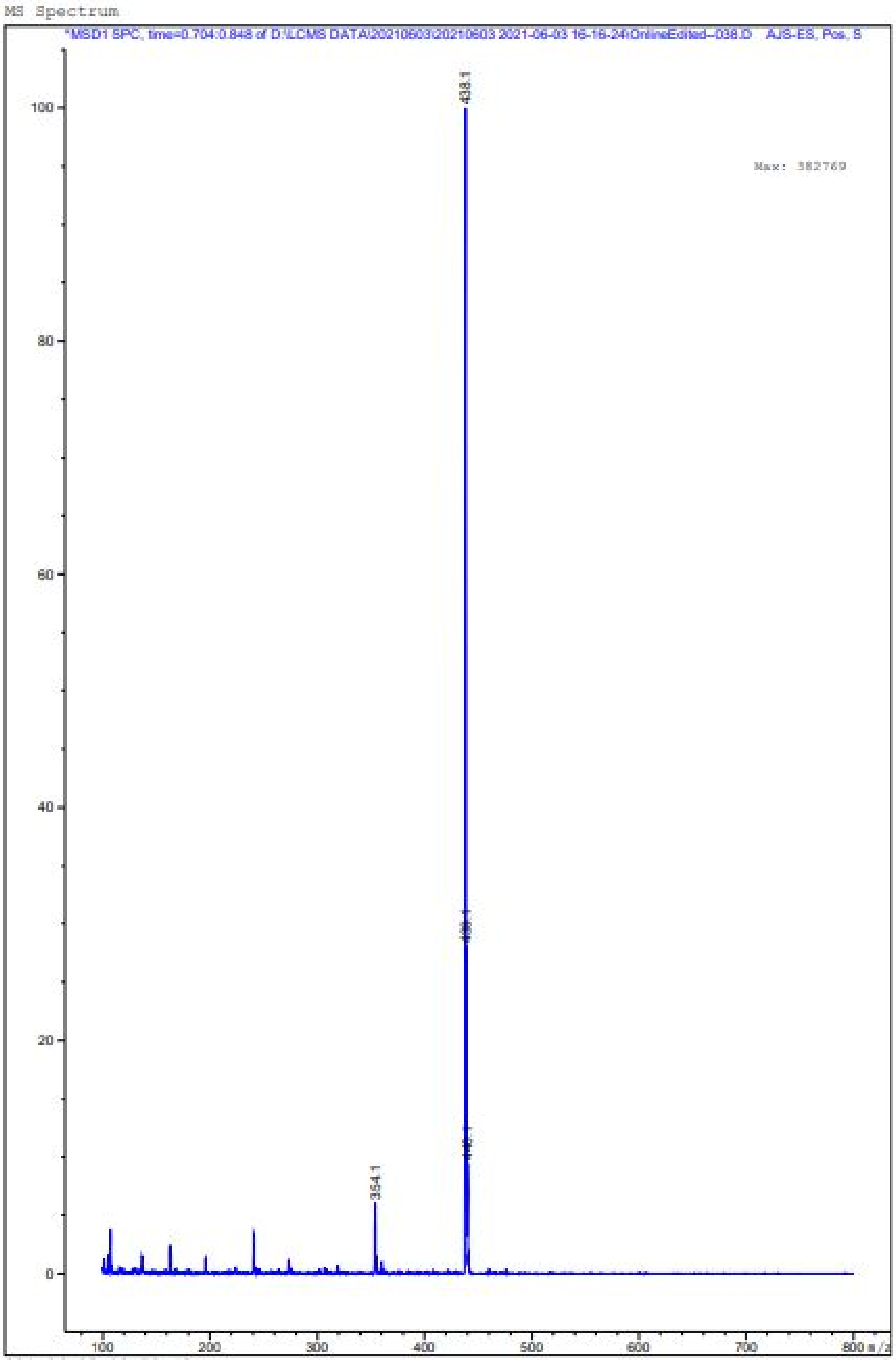
MS Spectrum
*MSD1 SPC, time=0.704:0.848 of D:\LCMS DATA\20210603\20210603 2021-06-03 16-16-24\OnlineEdited--038.D AJS-ES, Pos, S
438.1
Max: 382769
354.1
100
80
60
40
20
0
100
200
300
400
500
600
700
800 m/z

- ^{1}H NMR spectrum of Compound **15**

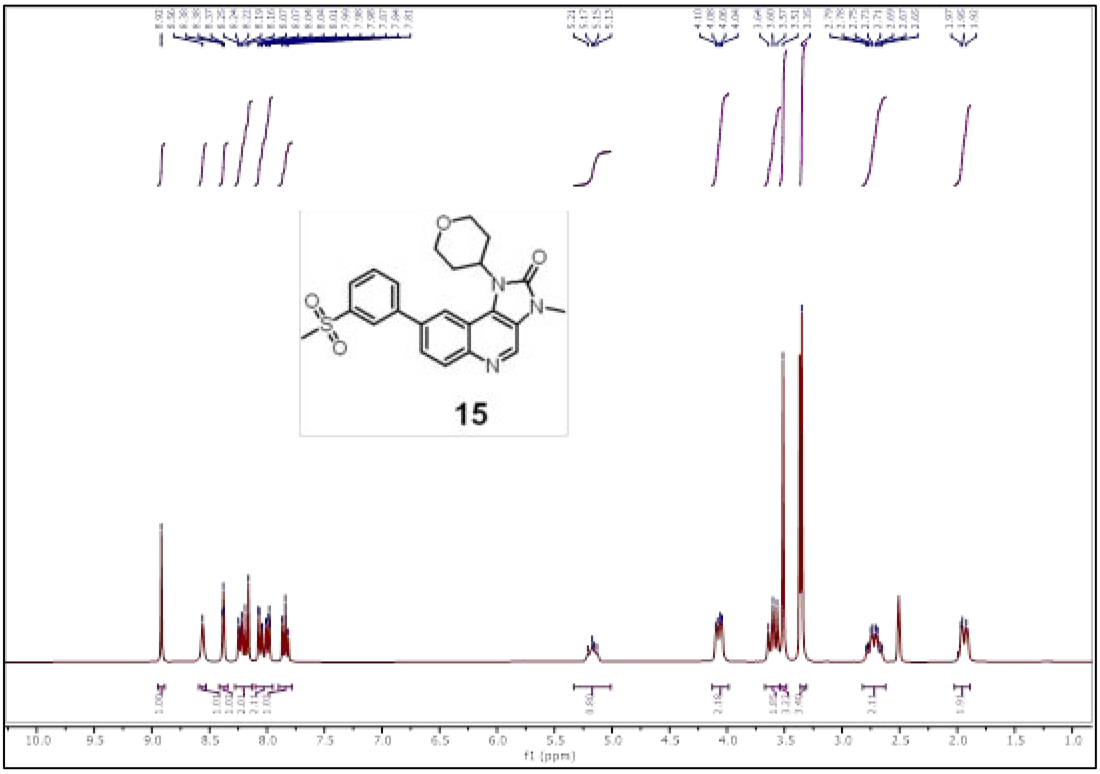

- ^{13}C NMR spectrum of Compound **15**

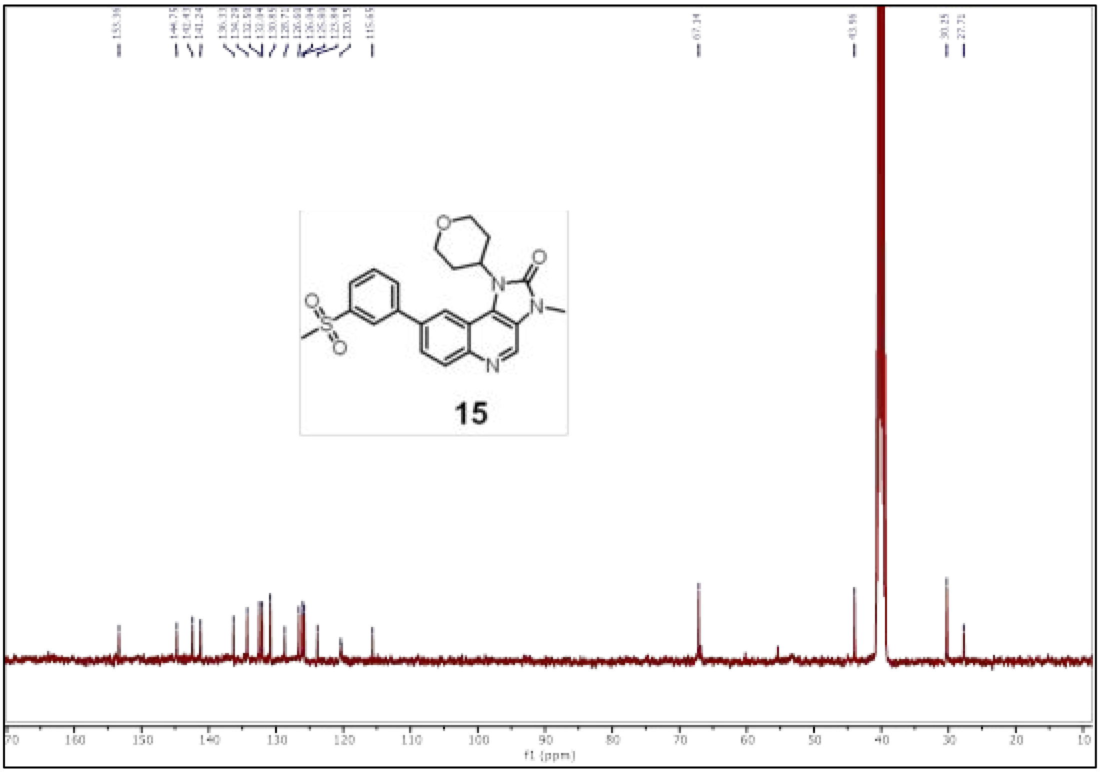

- HPLC trace of Compound **15**

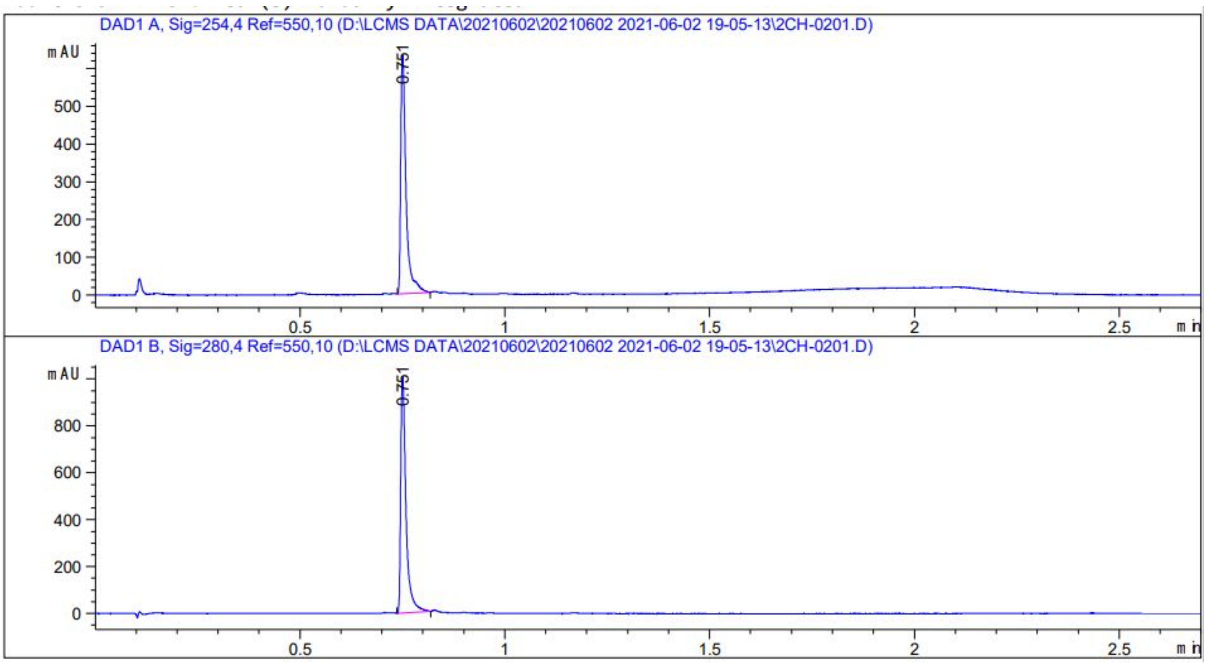

Peak #	RetTime [min]	Type	Width [min]	Area [mAU*s]	Height [mAU]	Area %
1	0.751	BB	0.0141	908.07971	998.62402	100.0000
Totals :				908.07971	998.62402	

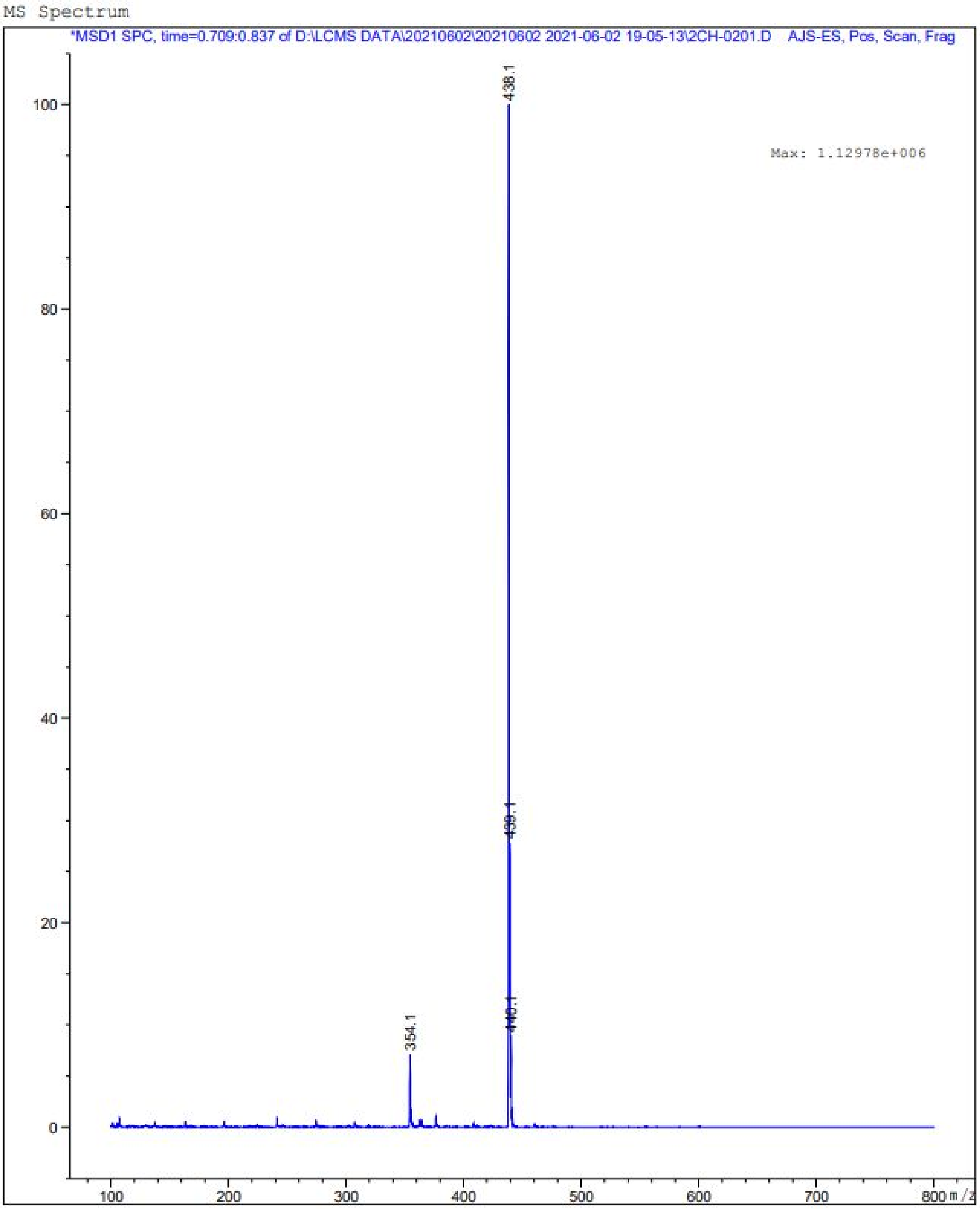
MS Spectrum
*MSD1 SPC, time=0.709:0.837 of D:\LCMS DATA\20210602\20210602 2021-06-02 19-05-13\2CH-0201.D AJS-ES, Pos, Scan, Frag
Max: 1.12978e+006
438.1
354.1
m/z

- ^{1}H NMR spectrum of Compound **16**

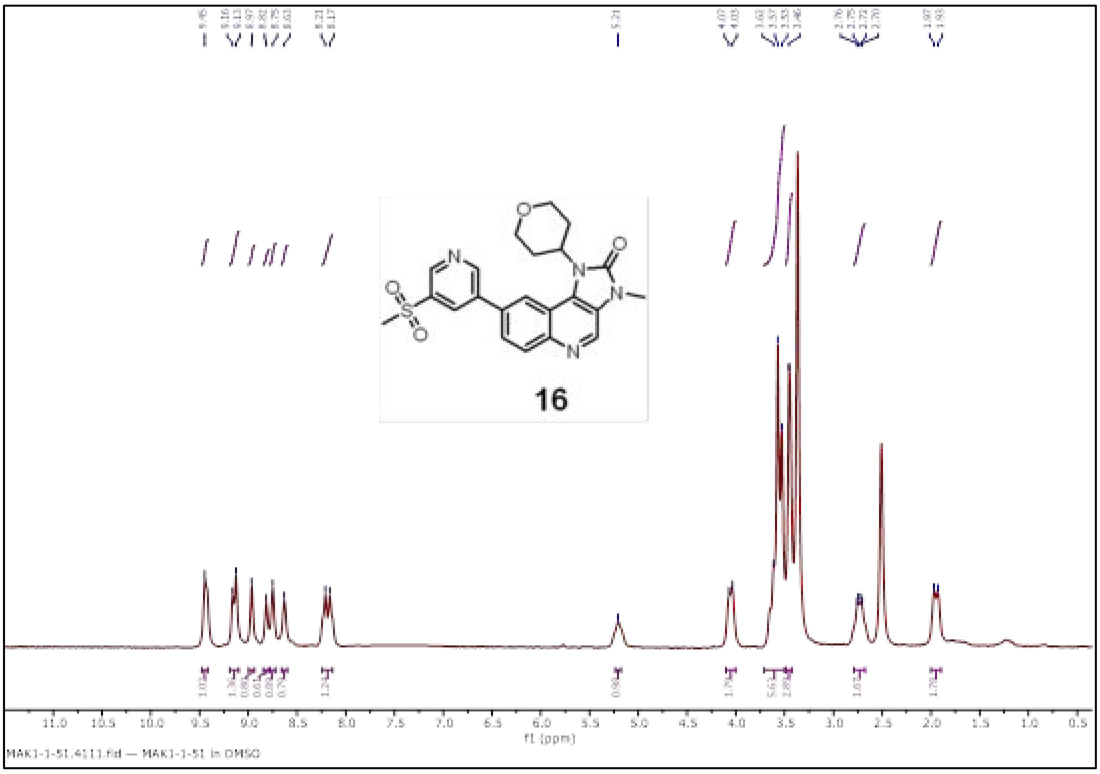

- ^{13}C NMR spectrum of Compound **16**

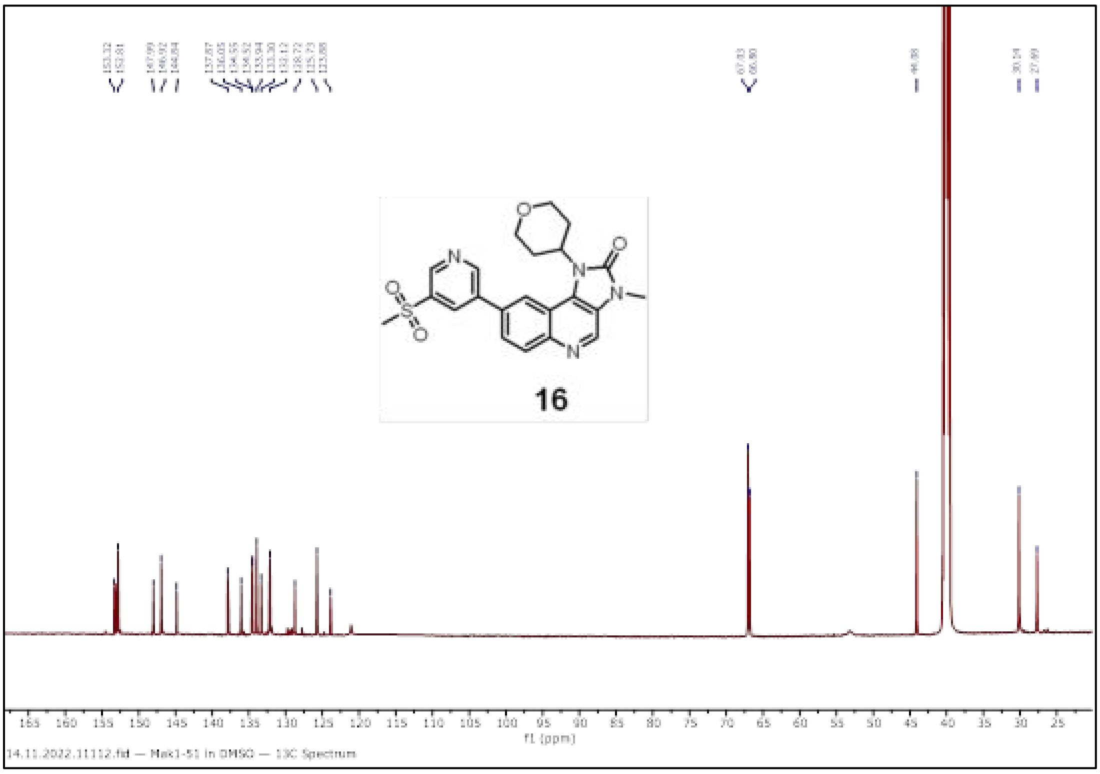

- HPLC trace of Compound **16**

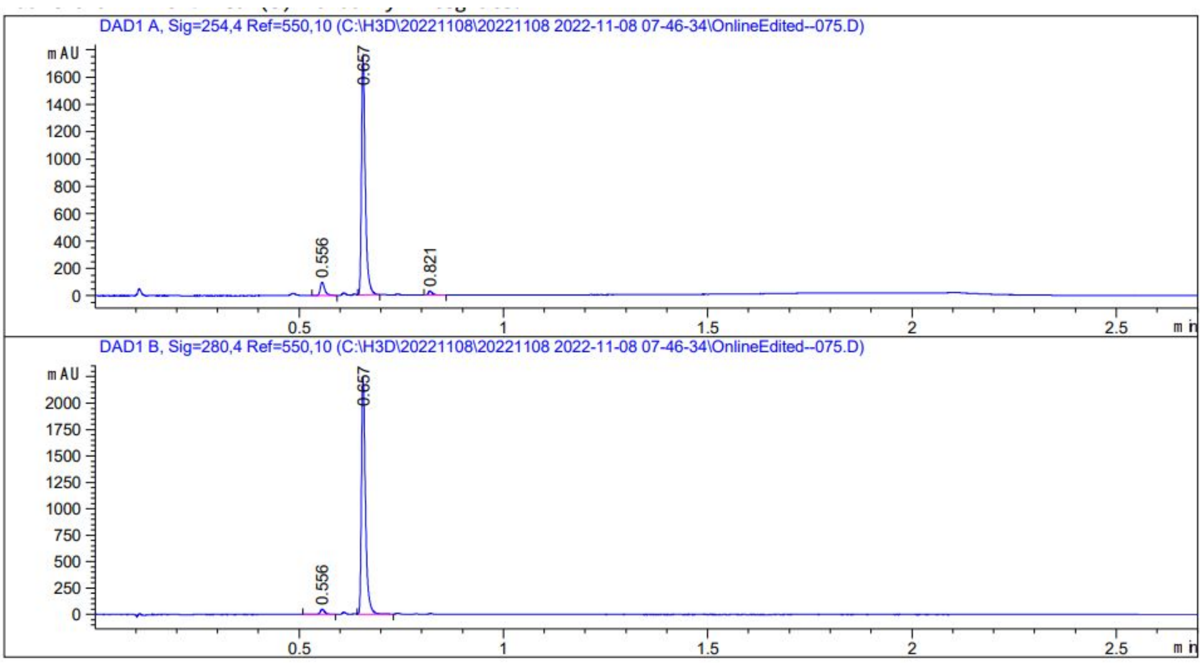

Peak #	RetTime [min]	Type	Width [min]	Area [mAU*s]	Height [mAU]	Area %
1	0.556	BB	0.0122	38.24431	48.75648	2.4572
2	0.657	BB	0.0109	1518.17310	2248.00952	97.5428
Totals :				1556.41741	2296.76600	

MS Spectrum

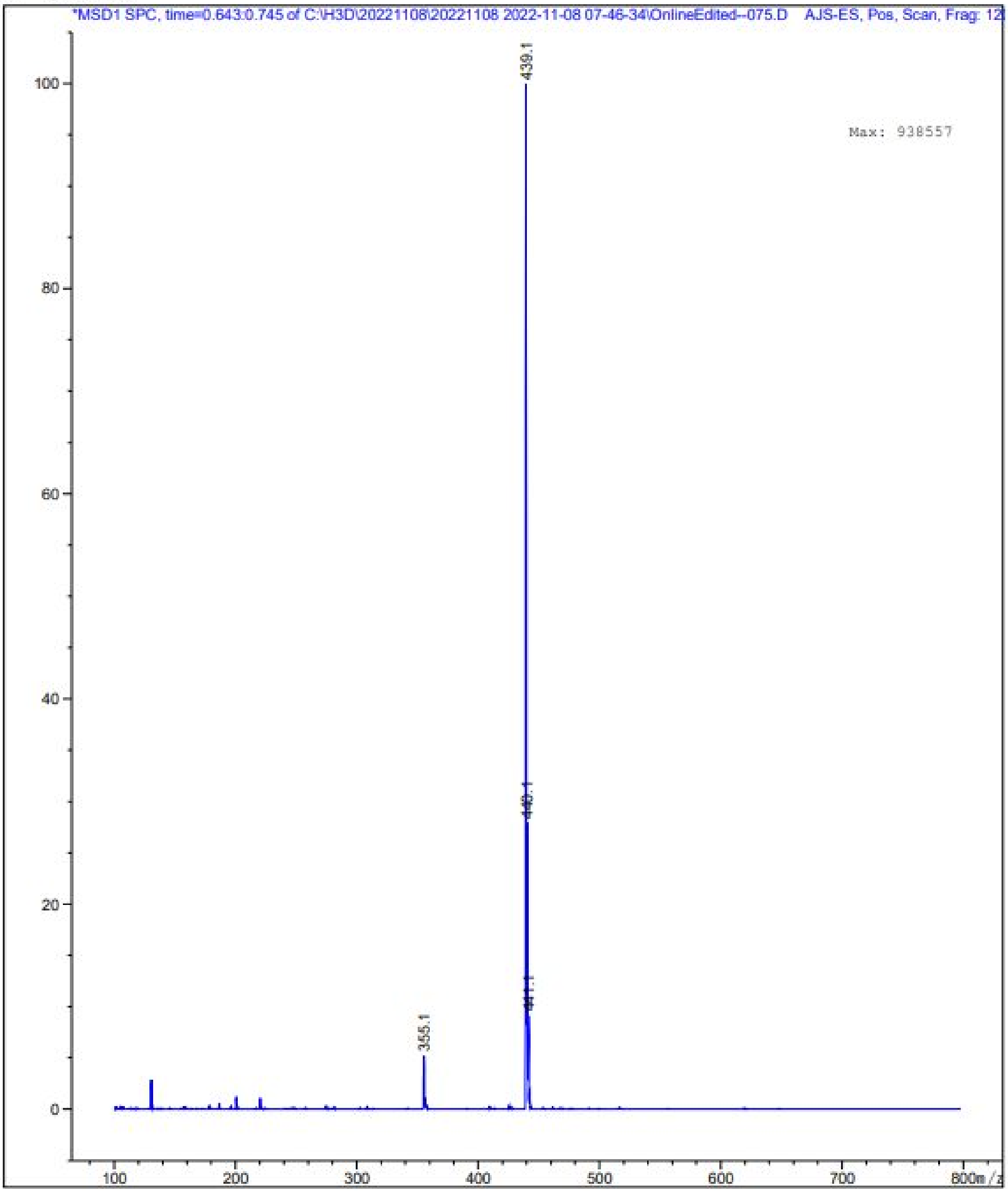

*MSD1 SPC, time=0.643:0.745 of C:\H3D\20221108\20221108 2022-11-08 07-46-34\OnlineEdited--075.D AJS-ES, Pos, Scan, Frag: 12
Max: 938557
439.1
355.1
100
80
60
40
20
0
100
200
300
400
500
600
700
800m/z

- ^{1}H NMR spectrum of Compound **17**

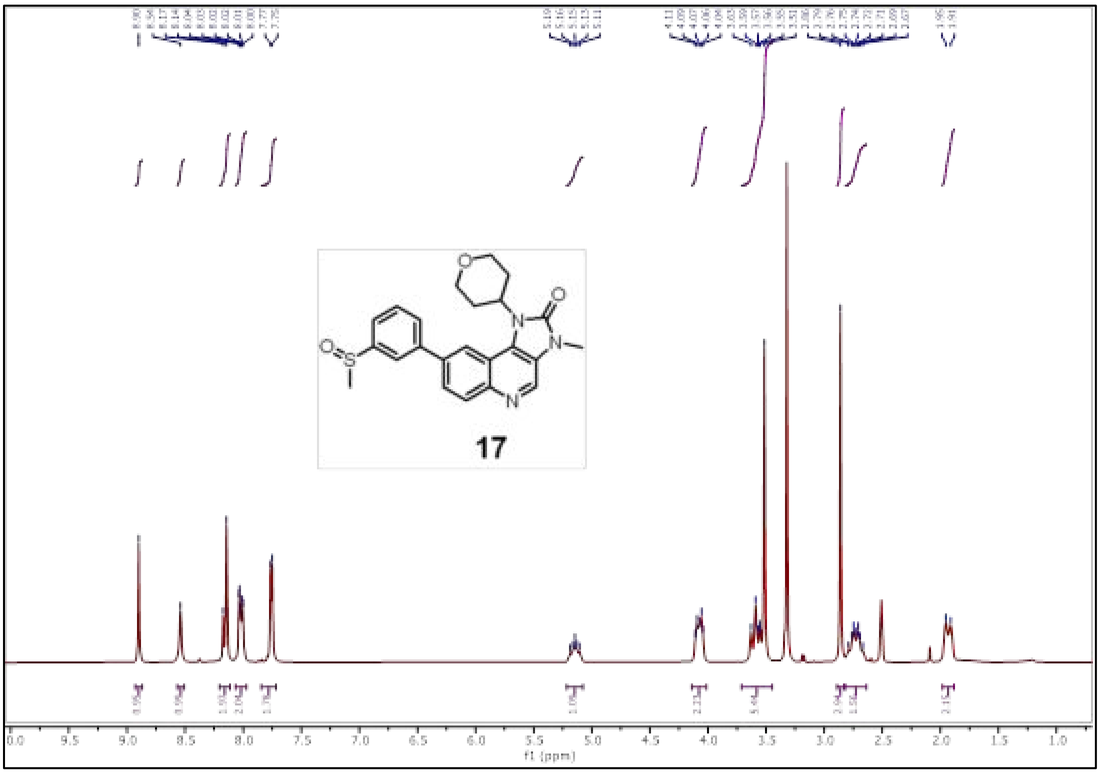

- ^{13}C NMR spectrum of Compound **17**

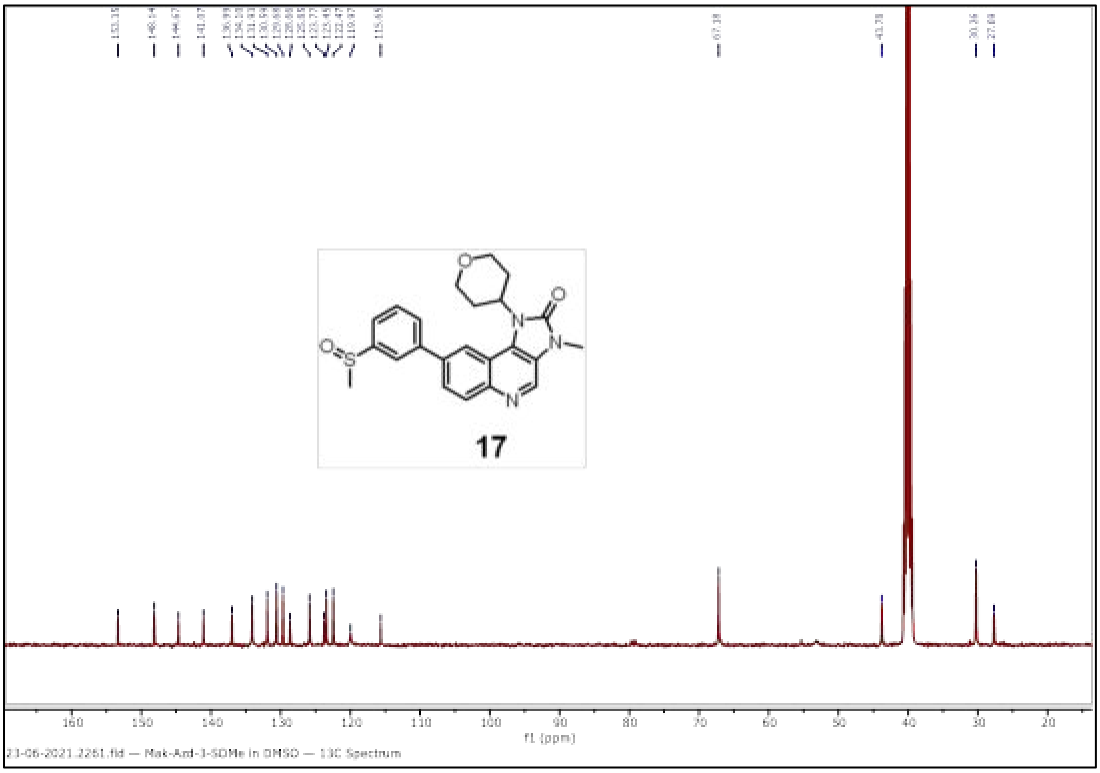

- HPLC trace of Compound **17**

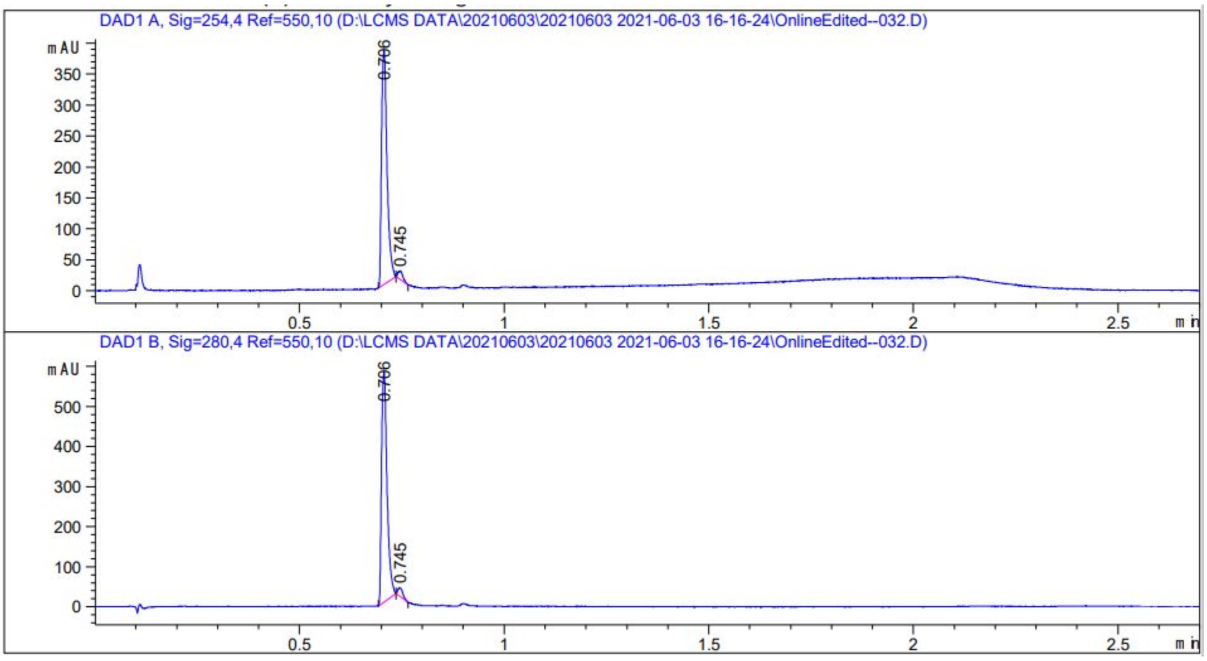

Peak #	RetTime [min]	Type	Width [min]	Area [mAU*s]	Height [mAU]	Area %
1	0.706	BB	0.0150	536.06970	570.88873	96.7834
2	0.745	BB	0.0138	17.81605	21.26246	3.2166
Totals :				553.88575	592.15119	

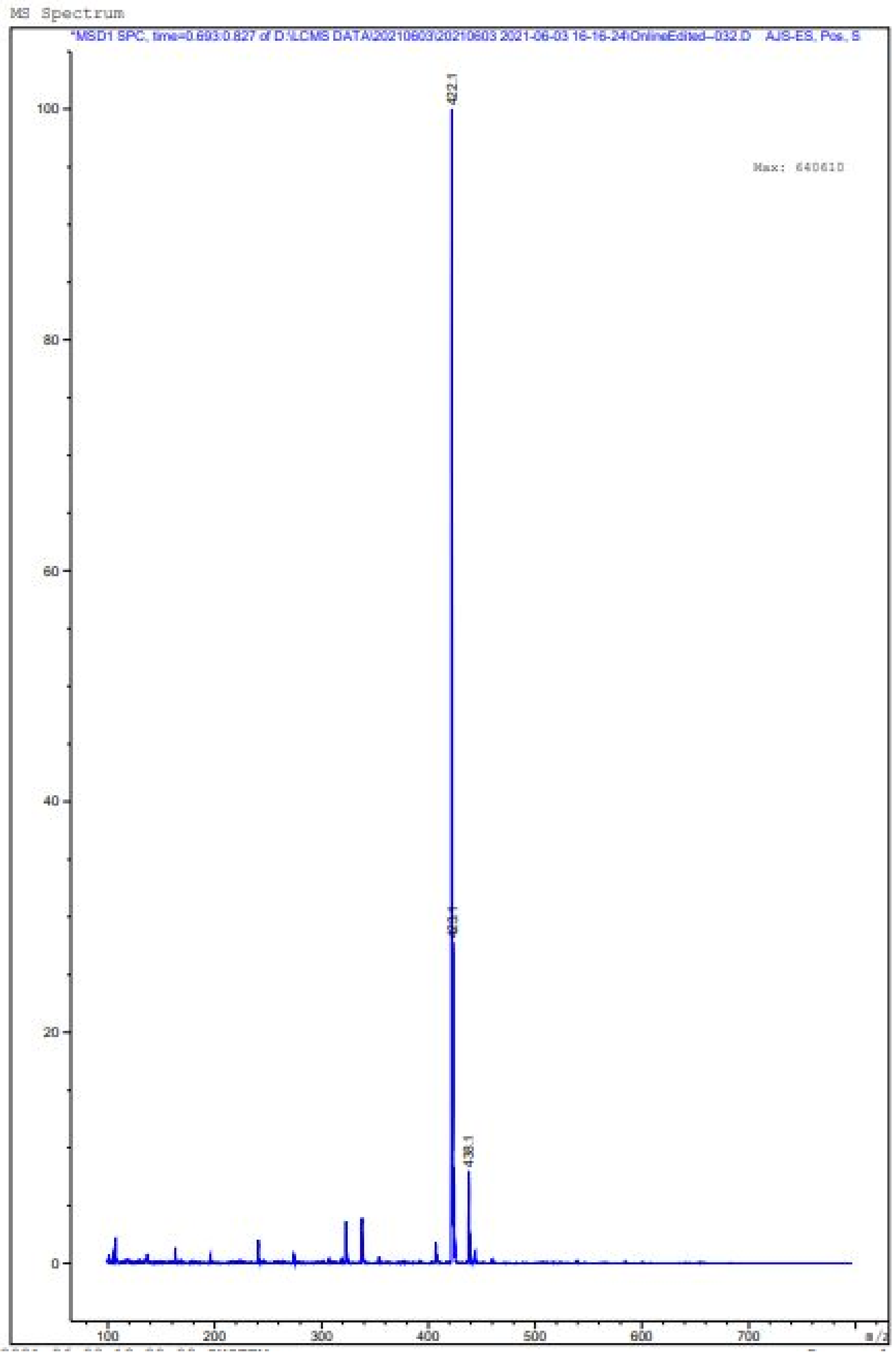
MS Spectrum
*MSD1 SPC, time=0.693:0.827 of D:\LCMS DATA\20210603\20210603 2021-06-03 16-16-24\OnlineEdited--032.D AJS-ES, Pos, S
422.1
438.1
Max: 640610
100
80
60
40
20
0
100
200
300
400
500
600
700

- ^{1}H NMR spectrum of Compound **19**

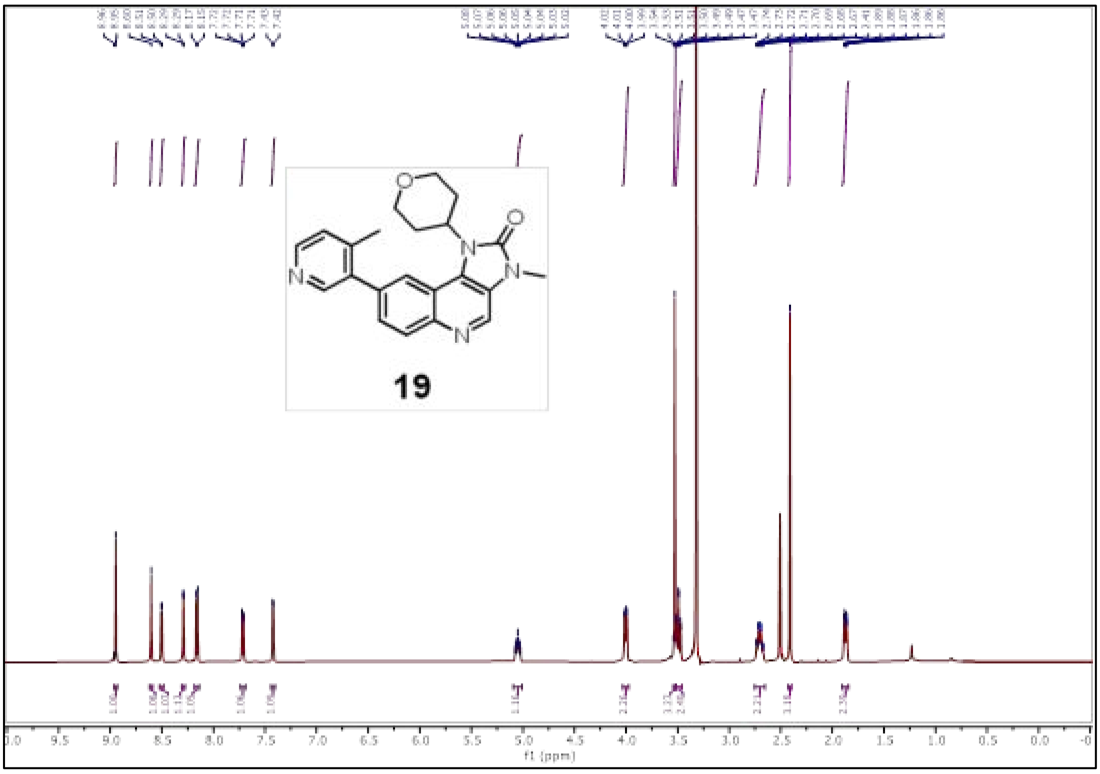

- ^{13}C NMR spectrum of Compound **19**

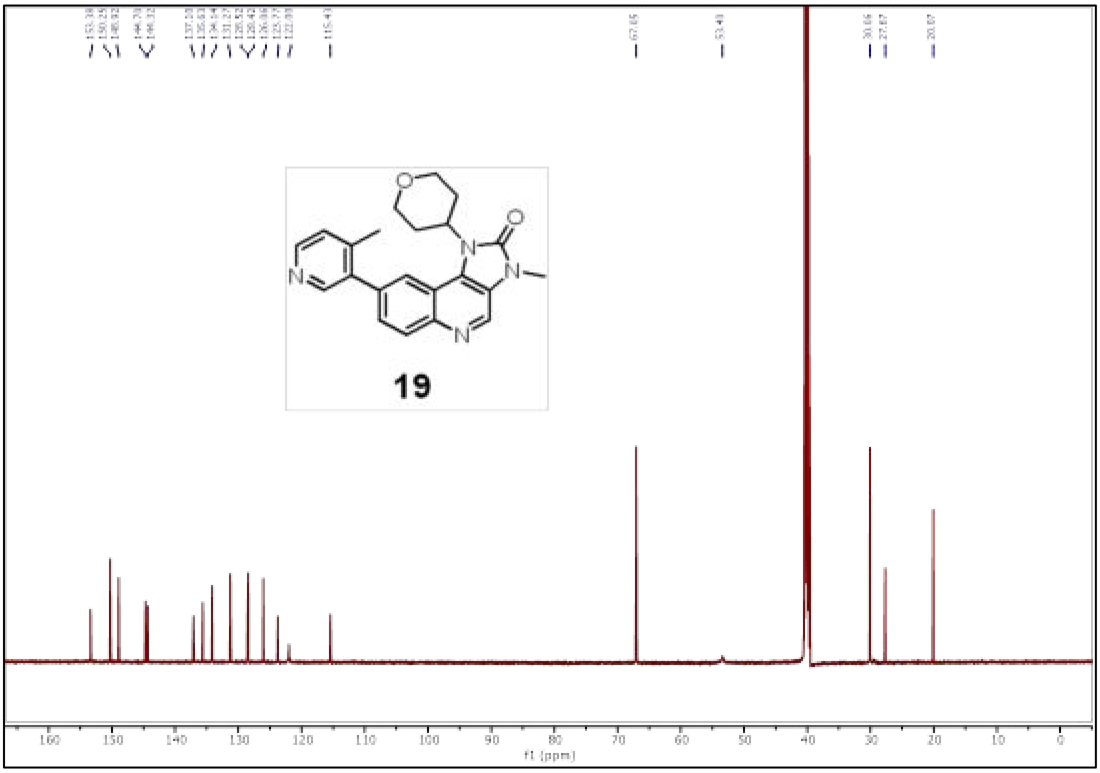

- HPLC trace of Compound **19**

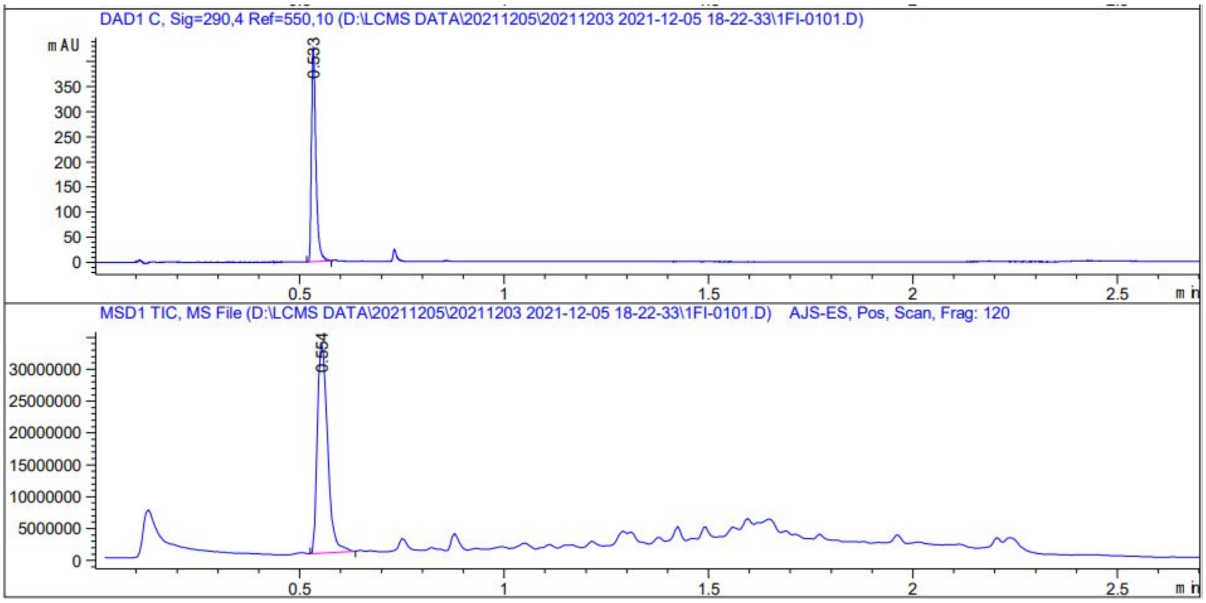

Peak #	RetTime [min]	Type	Width [min]	Area [mAU*s]	Height [mAU]	Area %
1	0.533	BB	0.0117	317.05640	424.38675	100.0000

Totals : 317.05640 424.38675

MS Spectrum

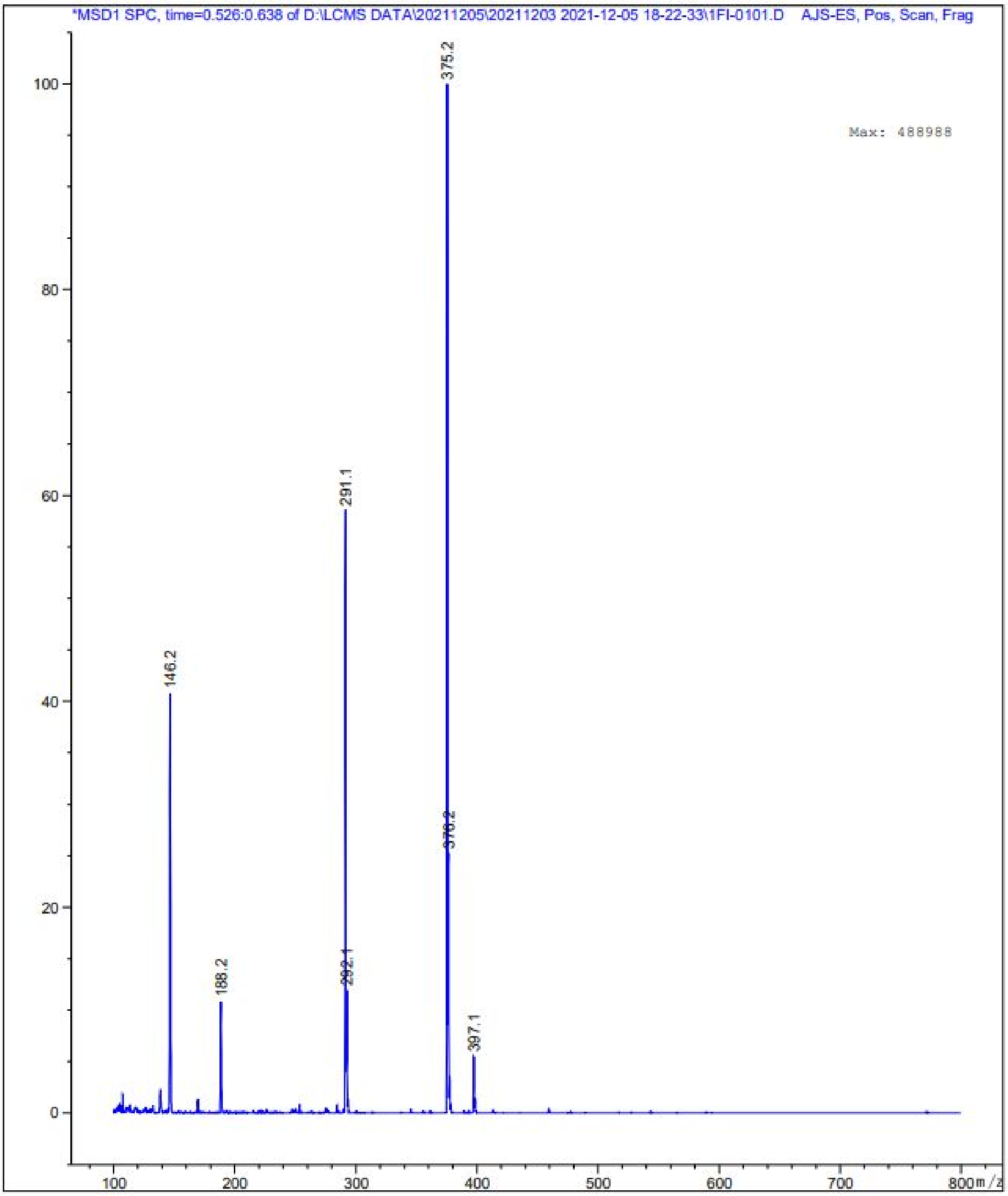

*MSD1 SPC, time=0.526:0.638 of D:\LCMS DATA\20211205\20211203 2021-12-05 18-22-33\1FI-0101.D AJS-ES, Pos, Scan, Frag
Max: 488988
375.2
291.1
146.2
376.2
188.2
292.1
397.1
100
80
60
40
20
0
100
200
300
400
500
600
700
800
m/z

- ^{1}H NMR spectrum of Compound **20**

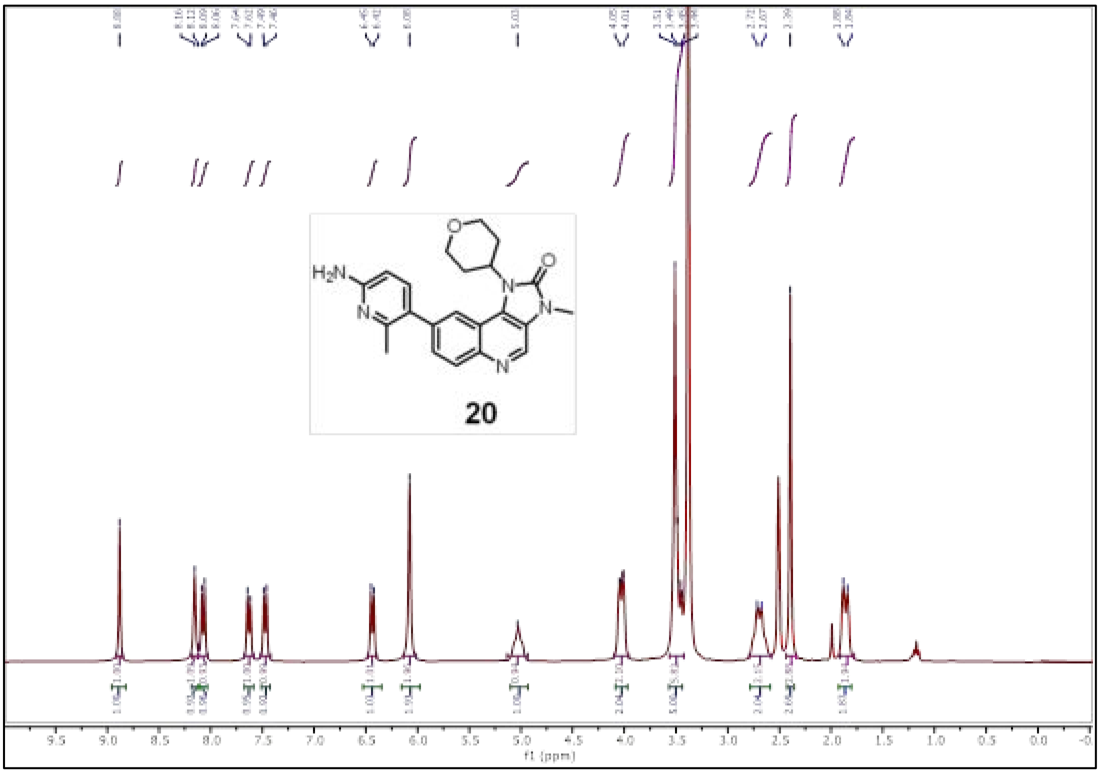

- ^{13}C NMR spectrum of Compound **20**

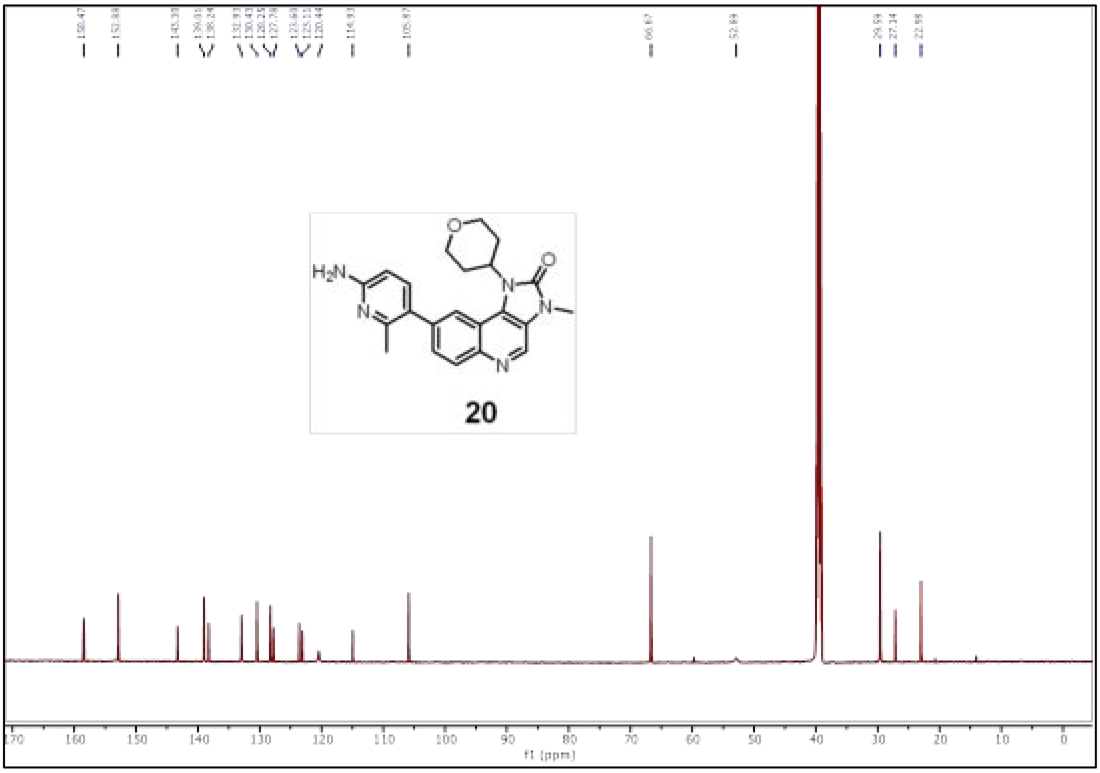

- HPLC trace of Compound **20**

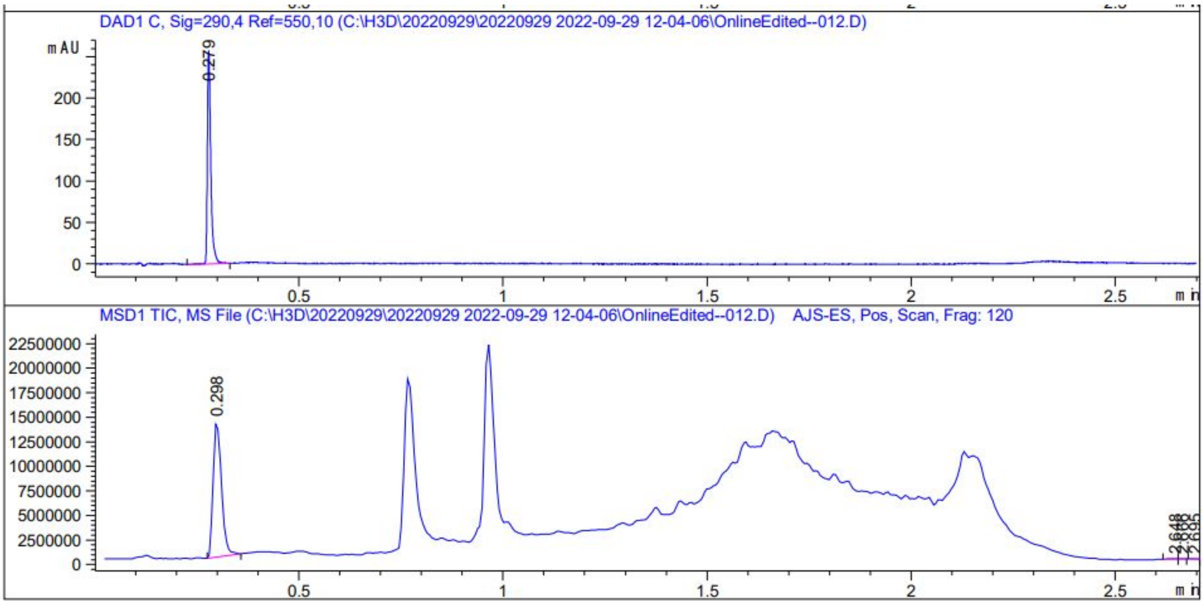


```
Peak RetTime Type  Width     Area      Height     Area
  #   [min]        [min]   [mAU*s]     [mAU]       %
----|-------|----|-------|----------|----------|--------|
   1   0.279 BB   9.04e-3  152.33865  252.62595 100.0000

Totals :                   152.33865  252.62595
```

MS Spectrum

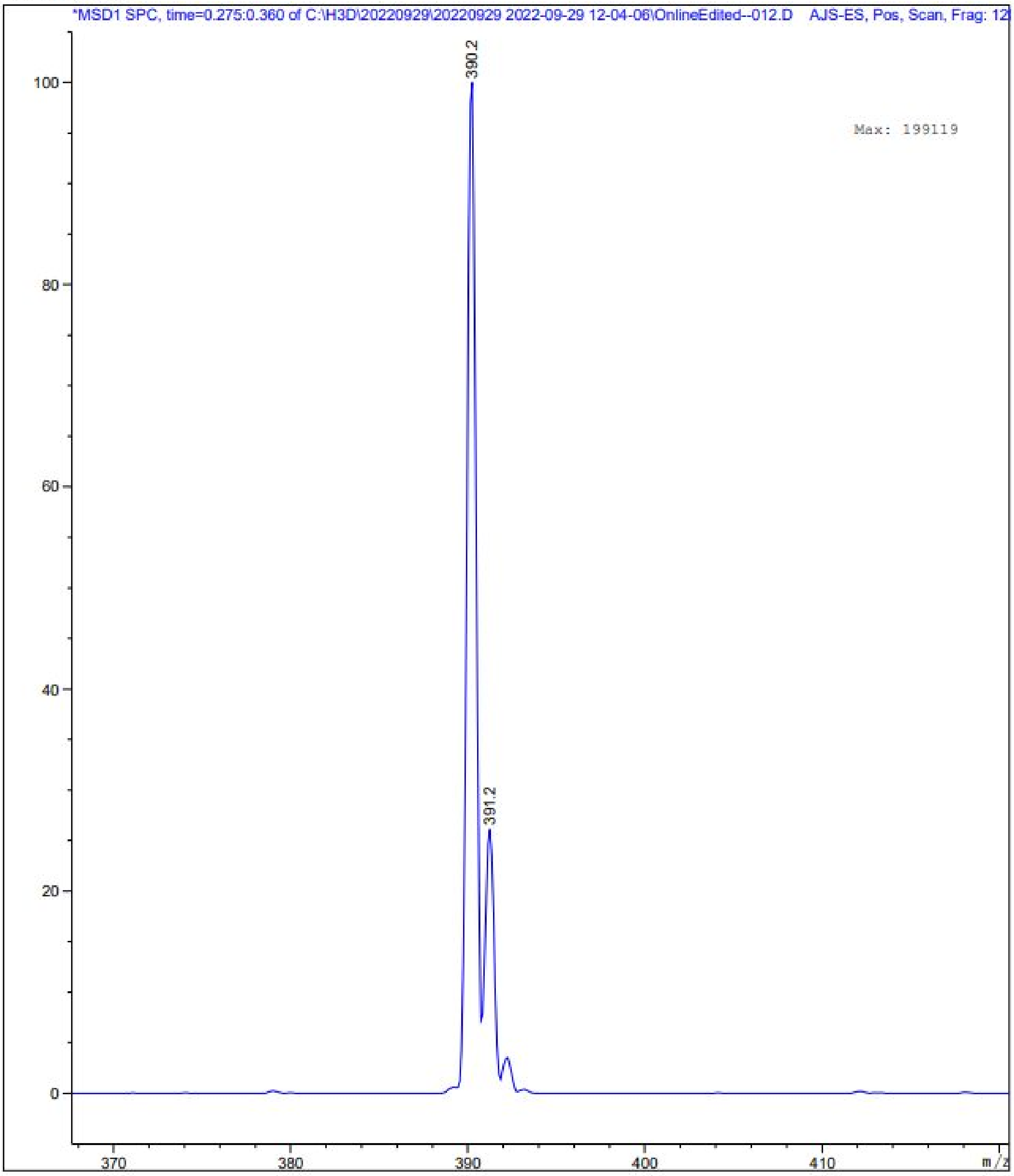
*MSD1 SPC, time=0.275:0.360 of C:\H3D\20220929\20220929 2022-09-29 12-04-06\OnlineEdited--012.D AJS-ES, Pos, Scan, Frag: 12
Max: 199119
390.2
391.2
100
80
60
40
20
0
370
380
390
400
410
m/z

- ^{1}H NMR spectrum of Compound **21**

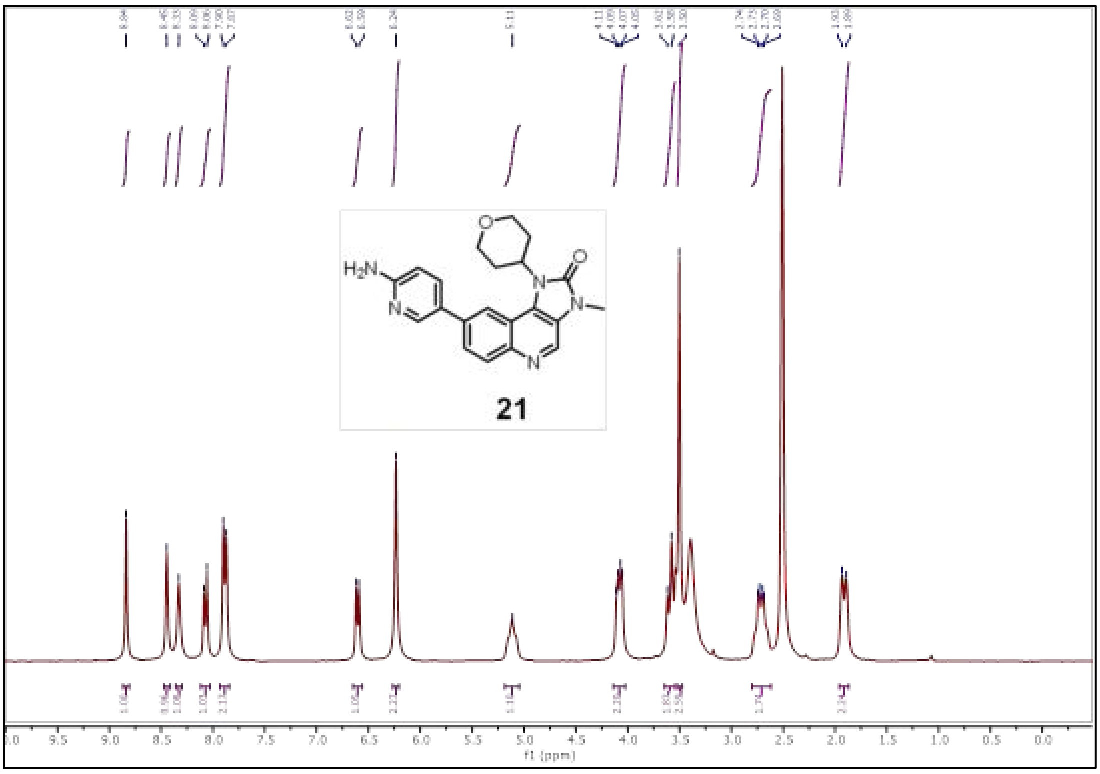

- ^{13}C NMR spectrum of Compound **21**

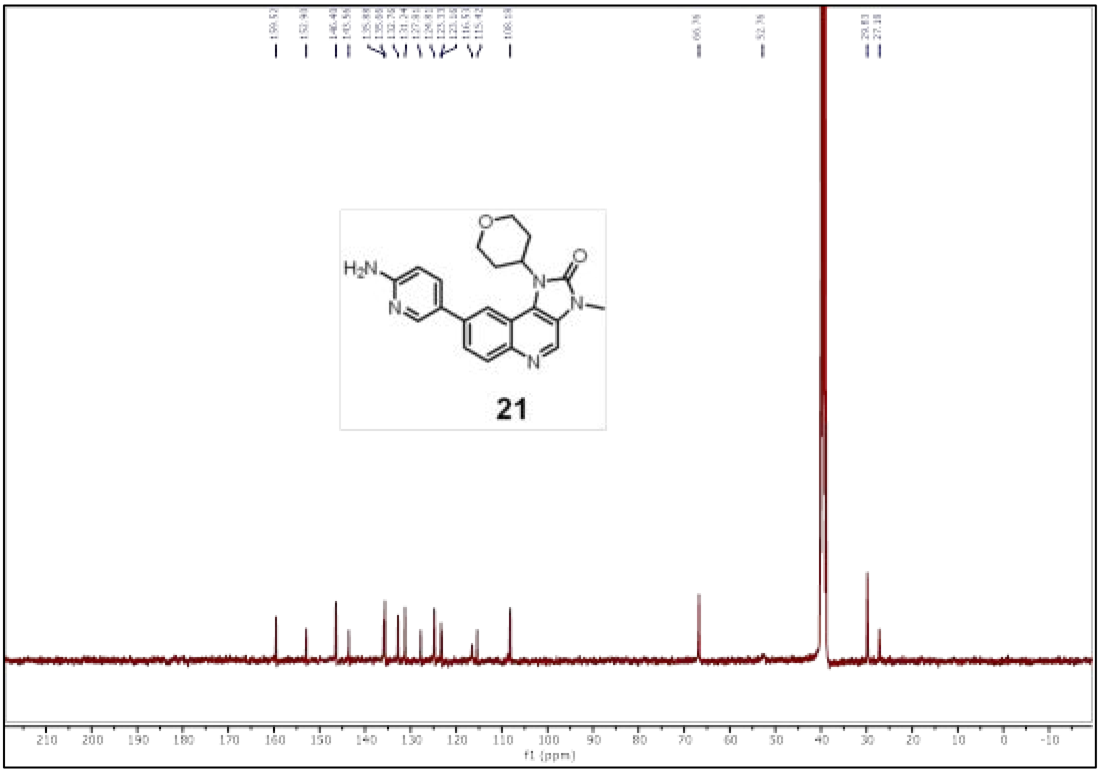

- HPLC trace of Compound **21**

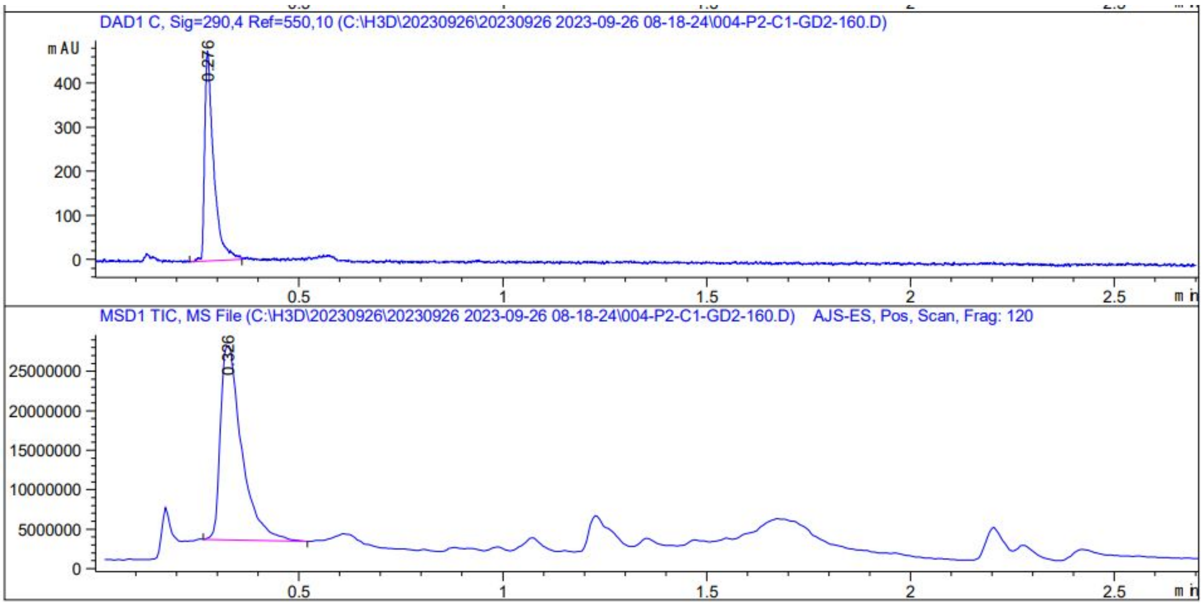

Peak #	RetTime [min]	Type	Width [min]	Area	Height	Area %
1	0.326	BB	0.0534	8.92677e7	2.47056e7	100.0000
Totals :				8.92677e7	2.47056e7	

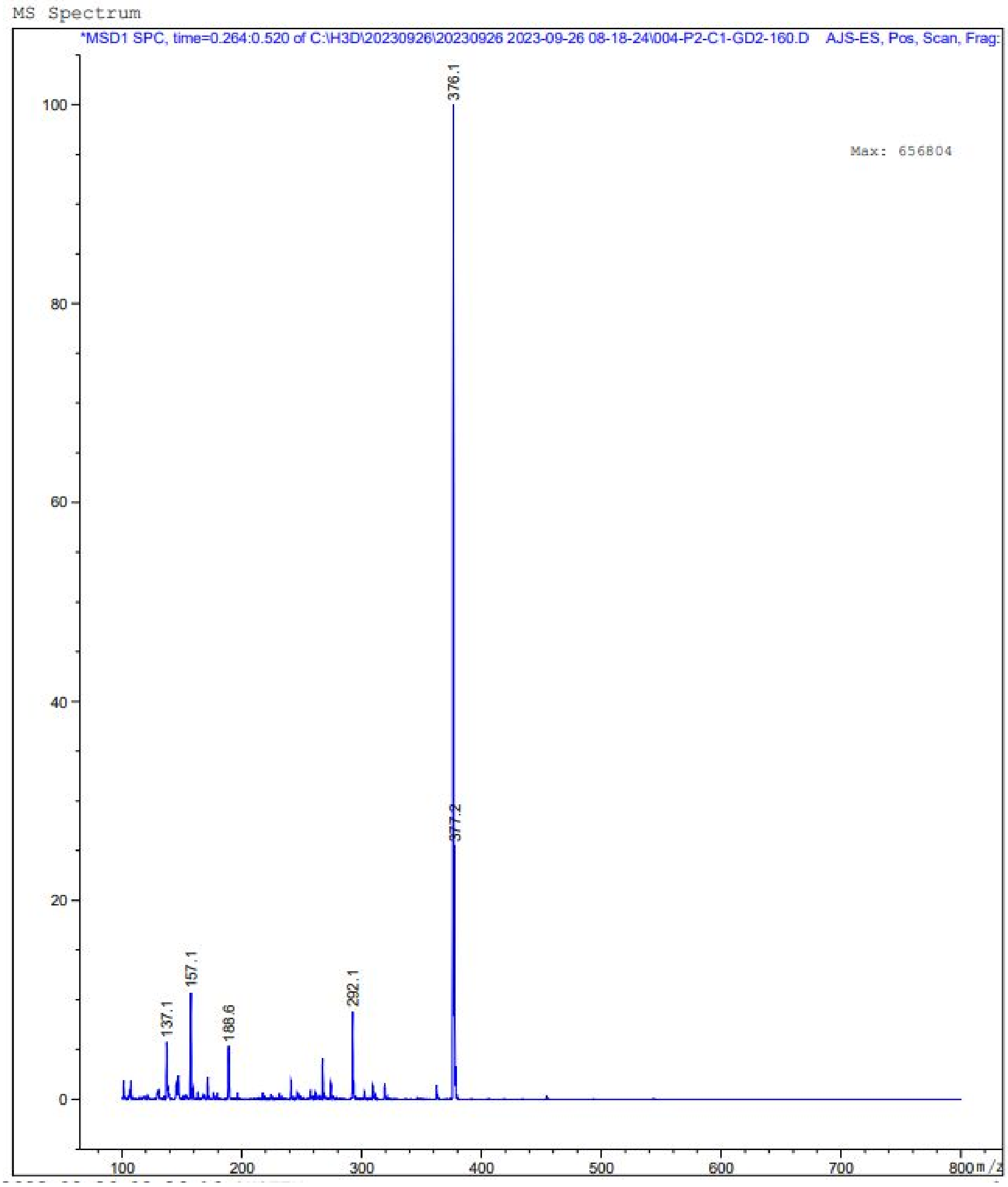
MS Spectrum
*MSD1 SPC, time=0.264:0.520 of C:\H3D\20230926\20230926 2023-09-26 08-18-24\004-P2-C1-GD2-160.D AJS-ES, Pos, Scan, Frag:
Max: 656804
376.1
377.2
137.1
157.1
188.6
292.1
m/z

- ^{1}H NMR spectrum of Compound **22**

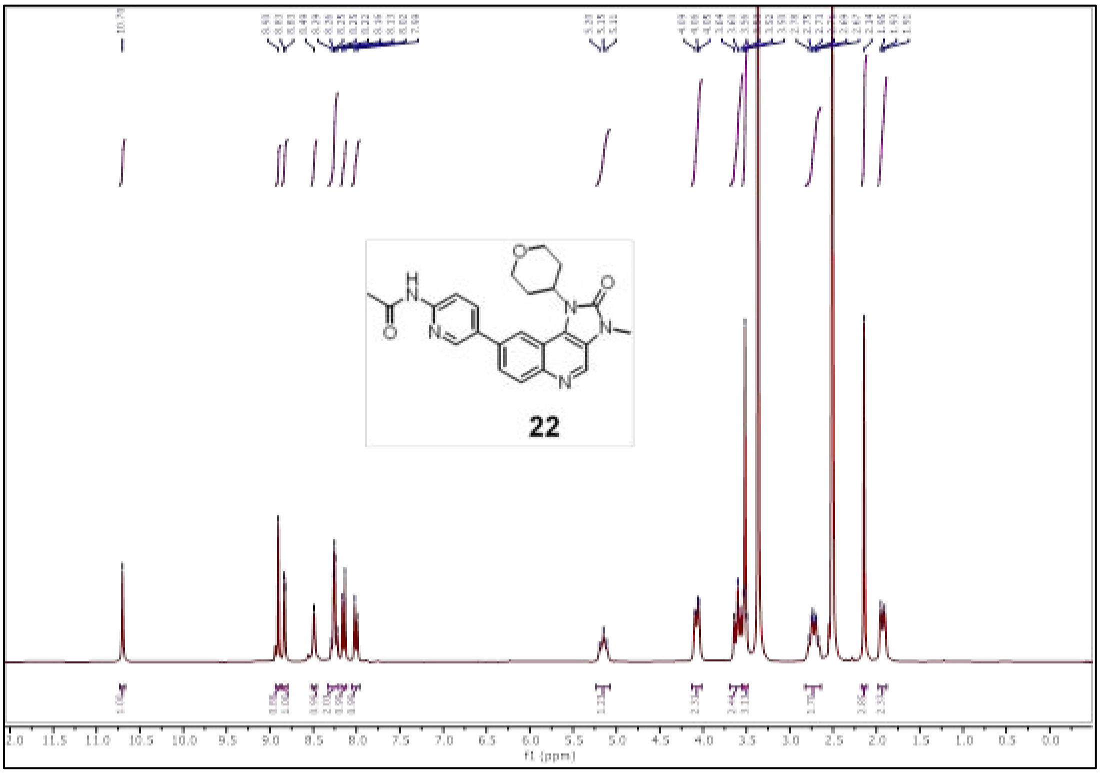

- ^{13}C NMR spectrum of Compound **22**

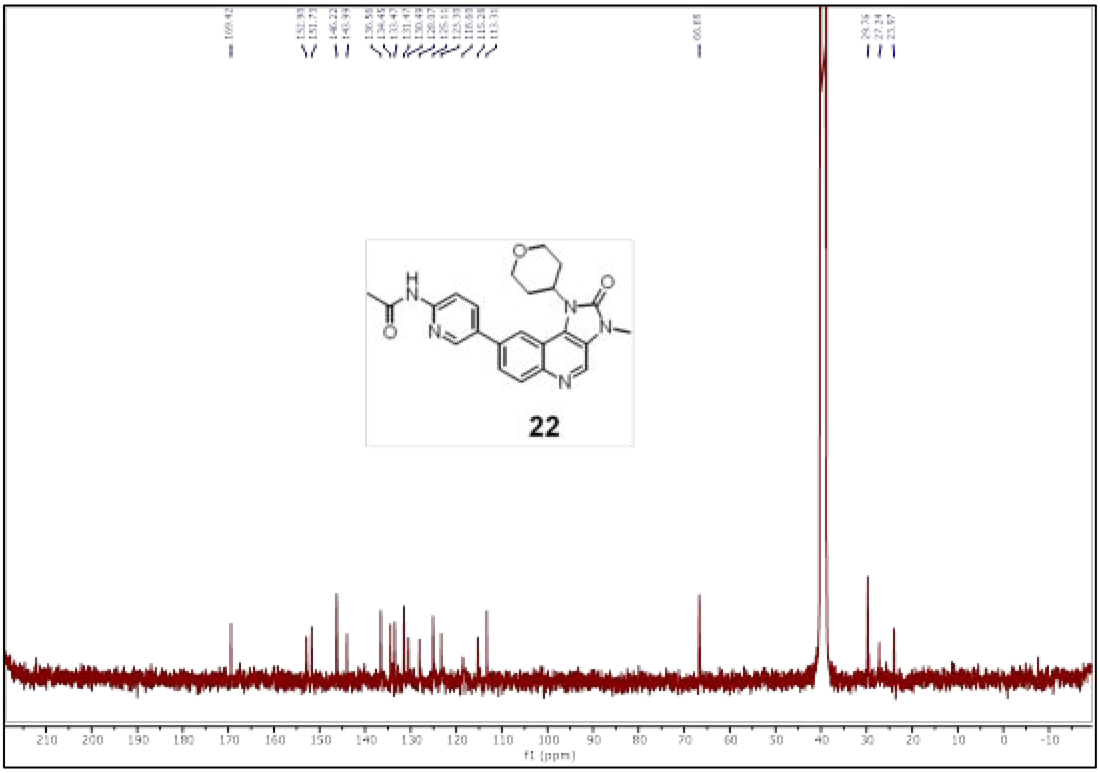

- HPLC trace of Compound **22**

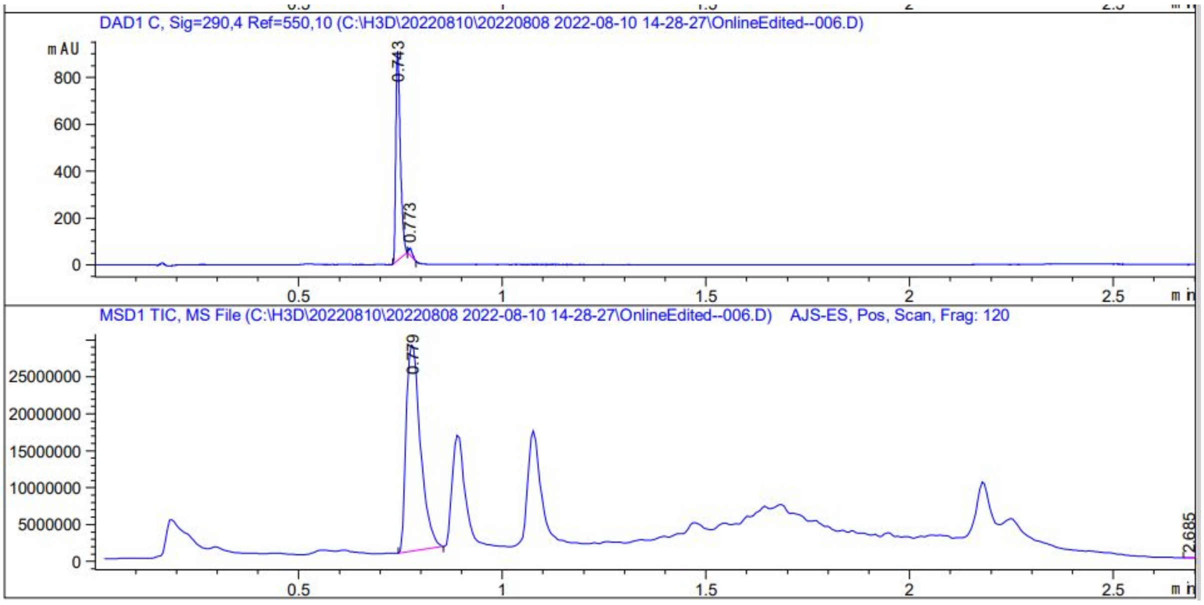

Peak #	RetTime [min]	Type	Width [min]	Area [mAU*s]	Height [mAU]	Area %
1	0.743	BB	0.0121	692.37738	889.34351	97.3896
2	0.773	BB	9.74e-3	18.55862	29.90574	2.6104
Totals :				710.93600	919.24925	

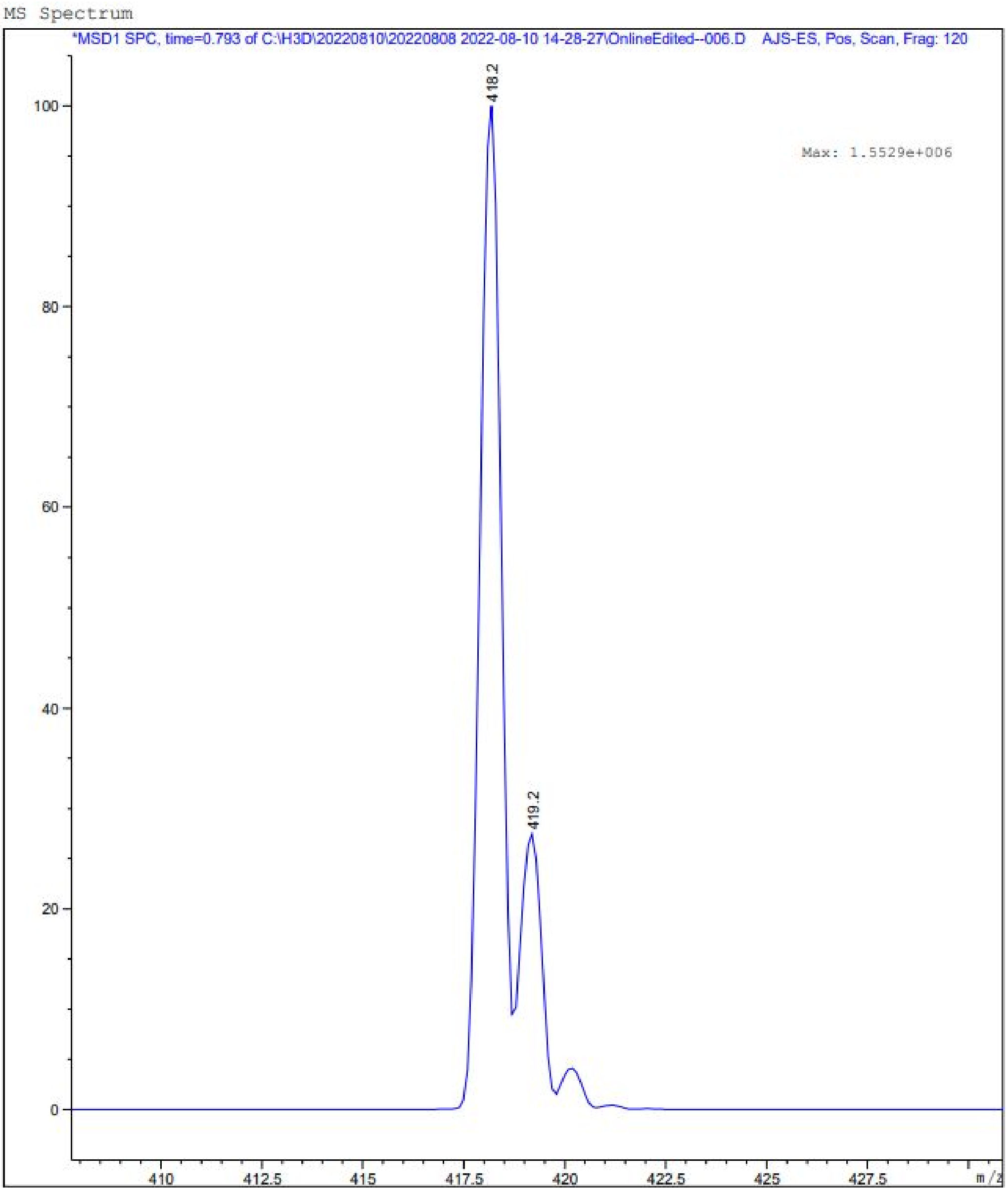
MS Spectrum
*MSD1 SPC, time=0.793 of C:\H3D\20220810\20220808 2022-08-10 14-28-27\OnlineEdited--006.D AJS-ES, Pos, Scan, Frag: 120
418.2
419.2
Max: 1.5529e+006
100
80
60
40
20
0
410
412.5
415
417.5
420
422.5
425
427.5
m/z

- ^{1}H NMR spectrum of Compound **23**

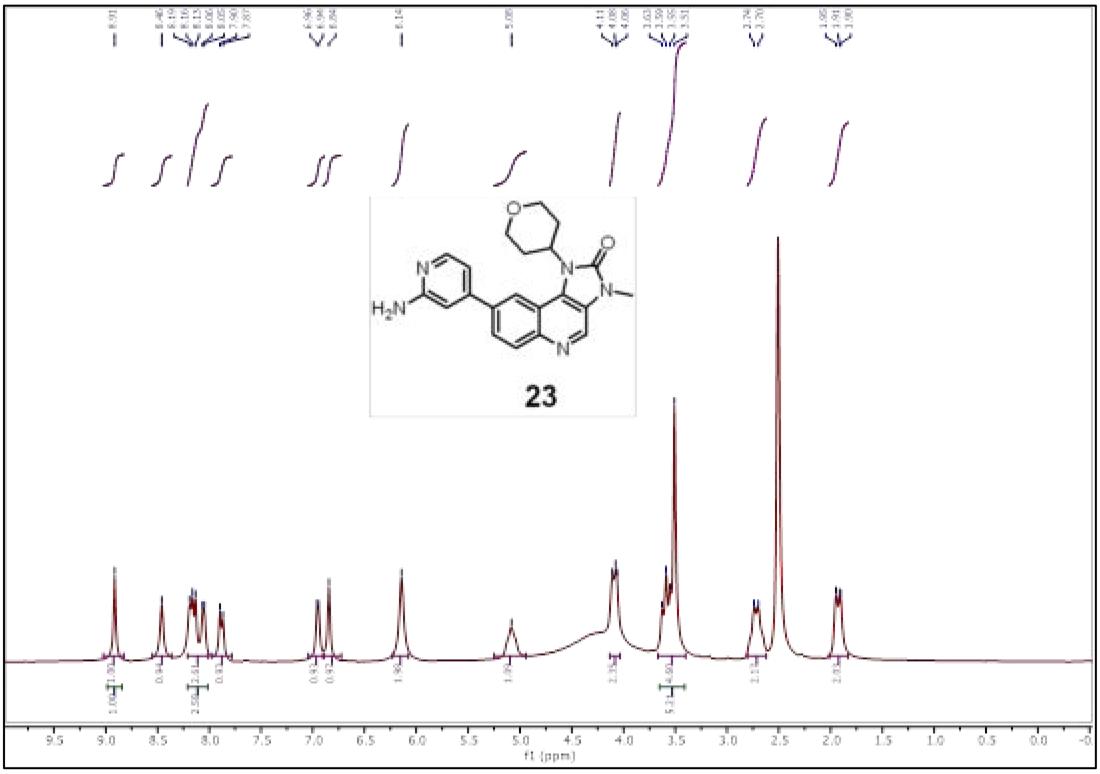

- ^{13}C NMR spectrum of Compound **23**

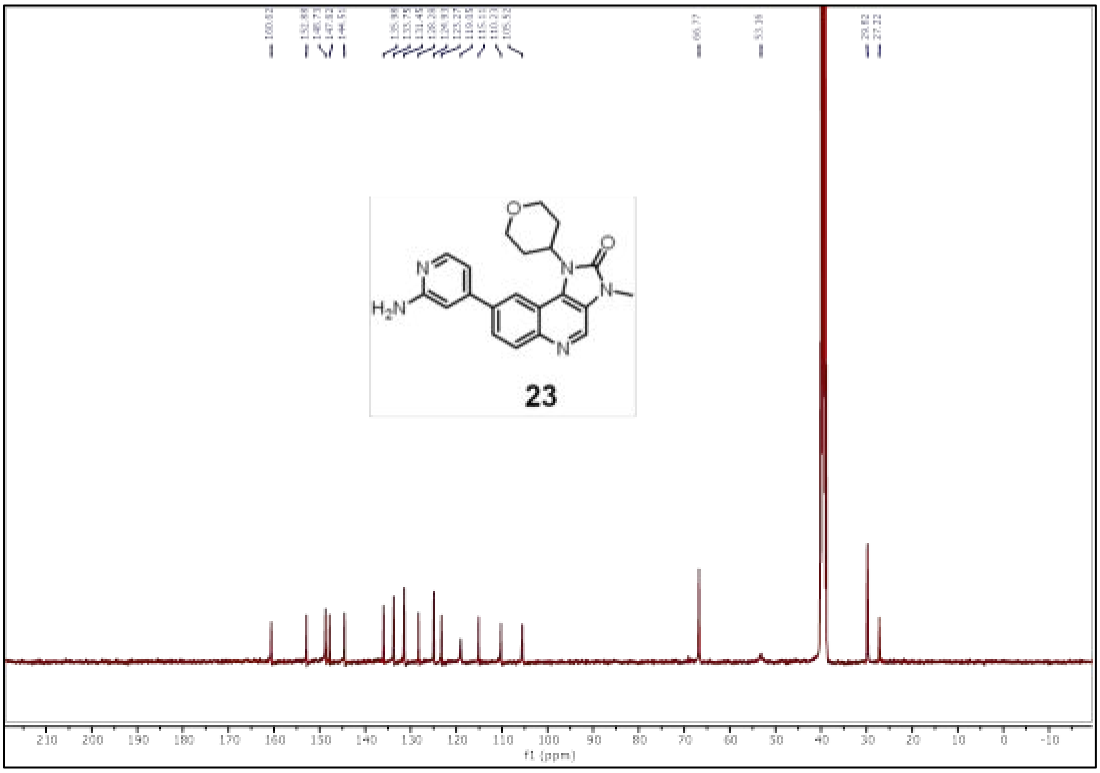

- HPLC trace of Compound **23**

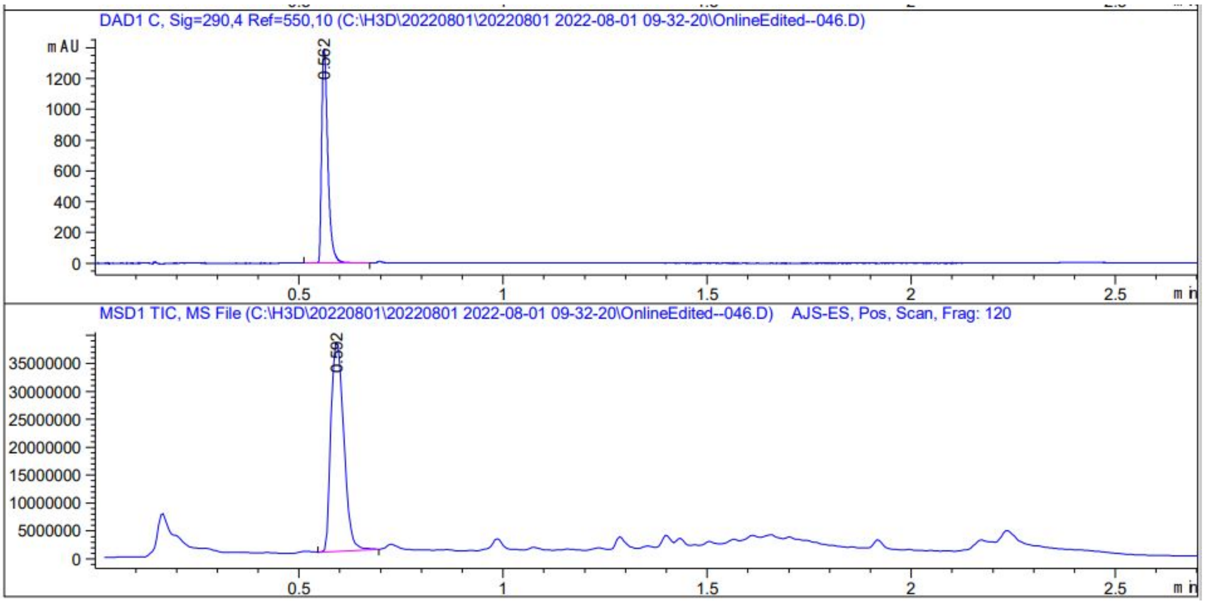

Peak #	RetTime [min]	Type	Width [min]	Area [mAU*s]	Height [mAU]	Area %
1	0.562	BB	0.0163	1450.84021	1381.53711	100.0000

Totals : 1450.84021 1381.53711

MS Spectrum

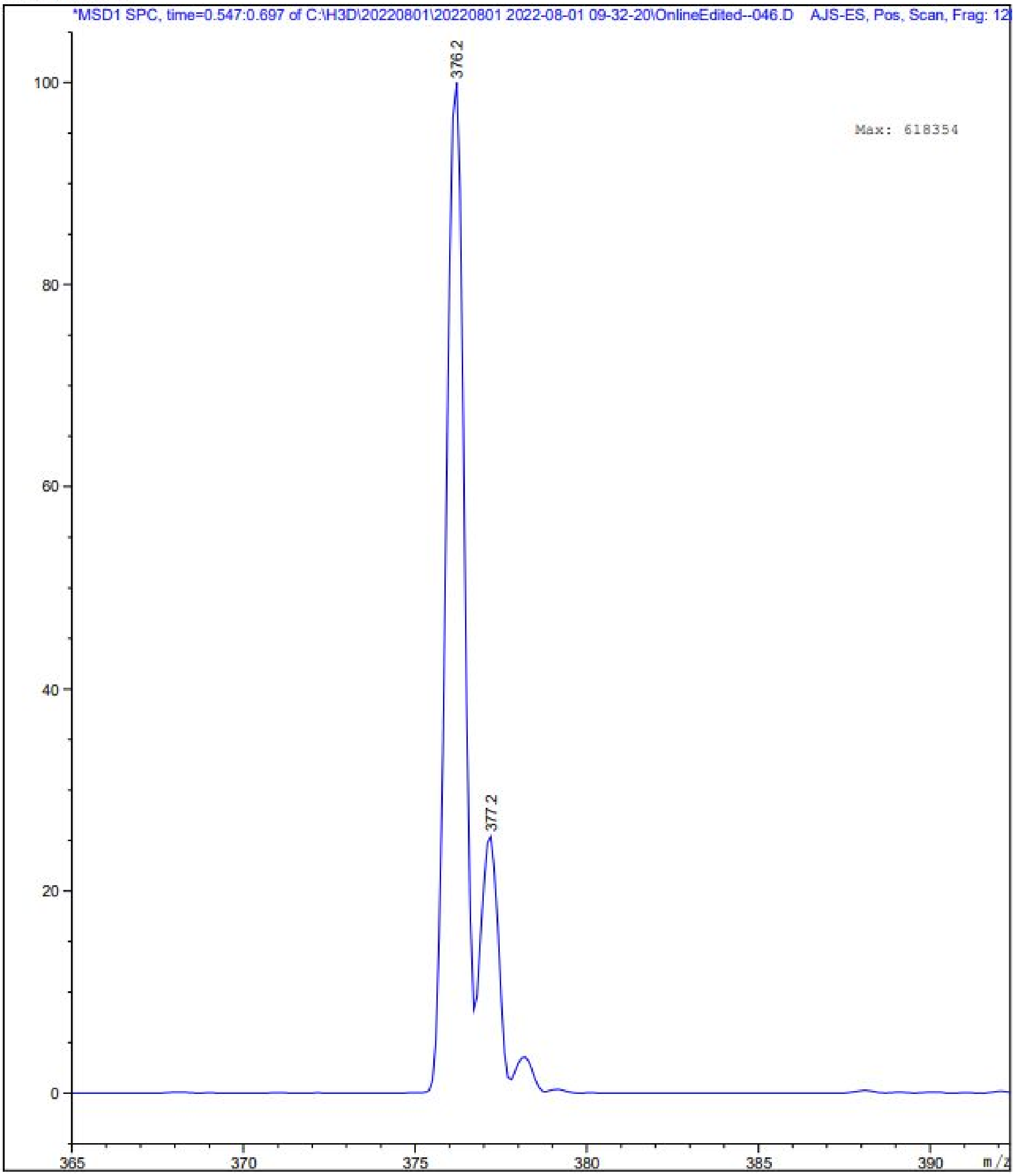
*MSD1 SPC, time=0.547:0.697 of C:\H3D\20220801\20220801 2022-08-01 09-32-20\OnlineEdited--046.D AJS-ES, Pos, Scan, Frag: 12
376.2
377.2
Max: 618354
100
80
60
40
20
0
365
370
375
380
385
390
m/z

- ^{1}H and ^{13}C NMR spectrum of Compound **24**

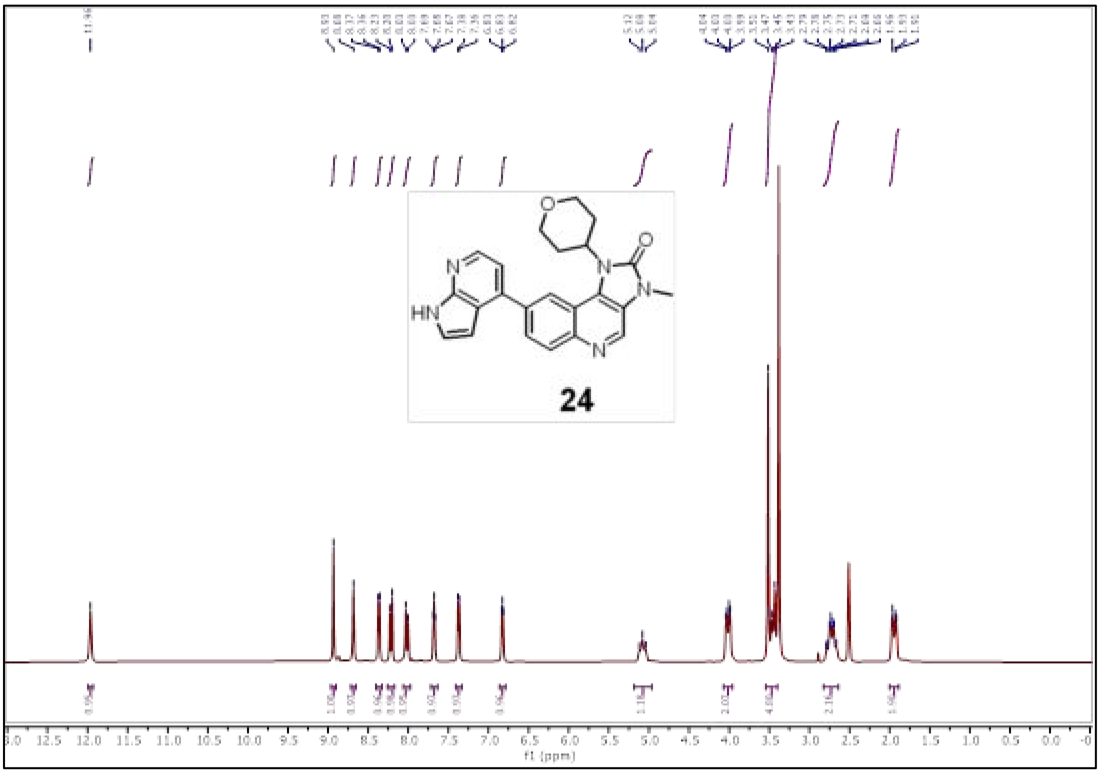

- ^{13}C NMR spectrum of Compound **24**

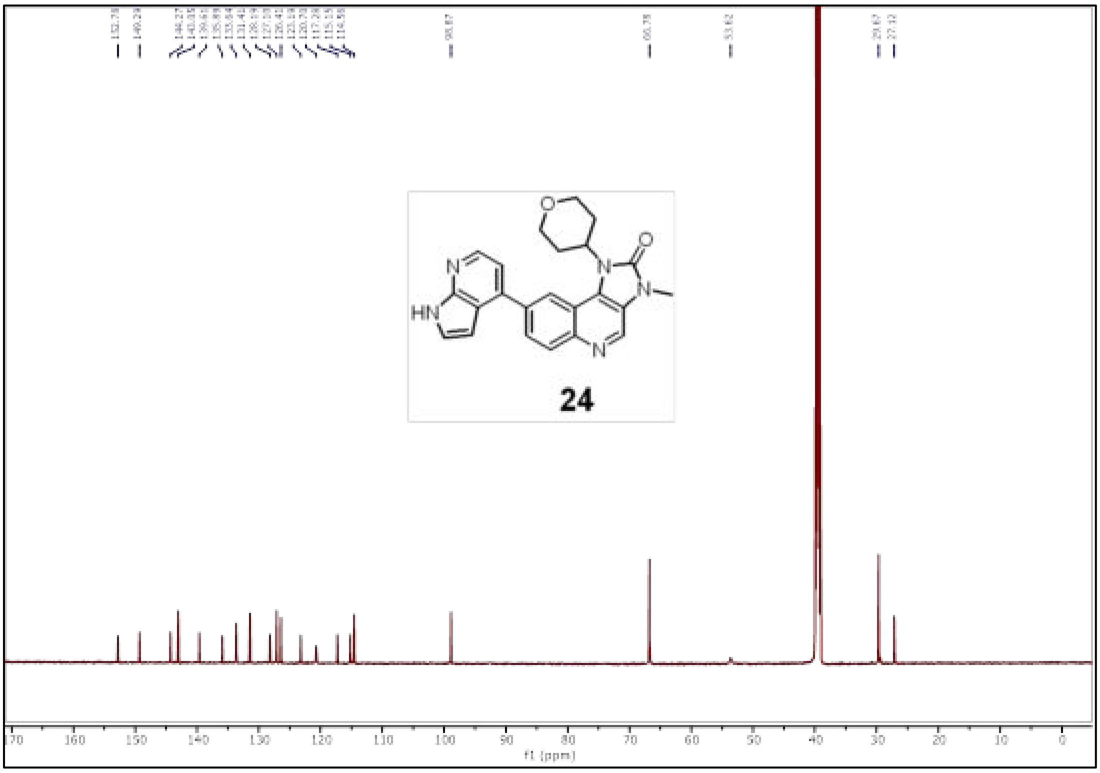

- HPLC trace of Compound **24**

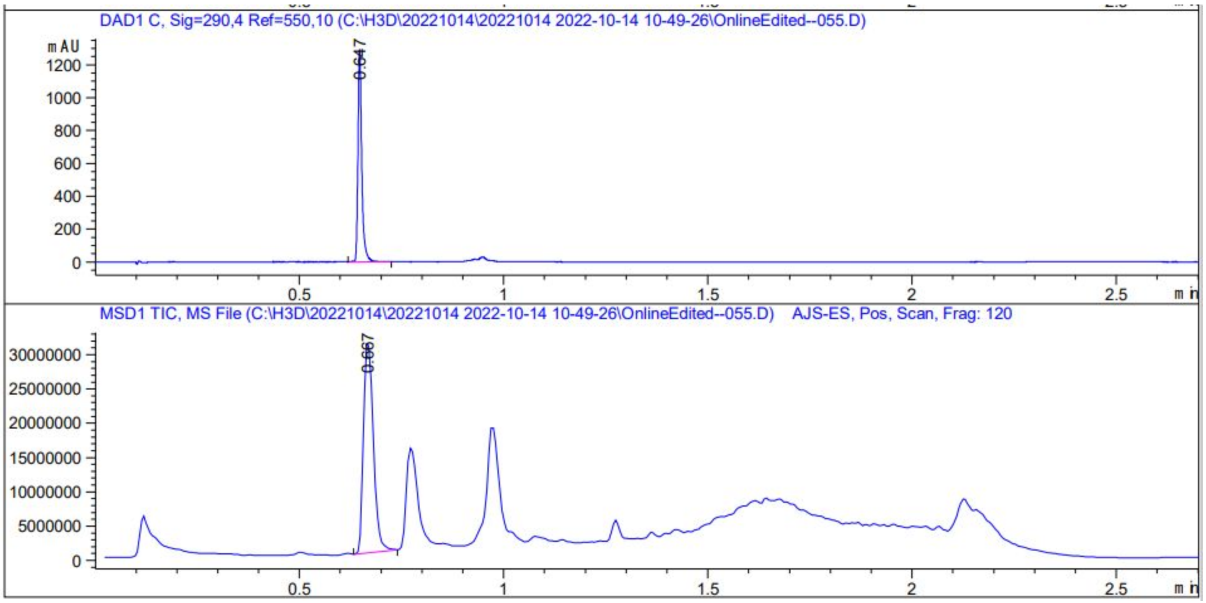

Peak #	RetTime [min]	Type	Width [min]	Area [mAU*s]	Height [mAU]	Area %
1	0.647	BB	0.0101	835.27954	1278.77051	100.0000

Totals : 835.27954 1278.77051

MS Spectrum

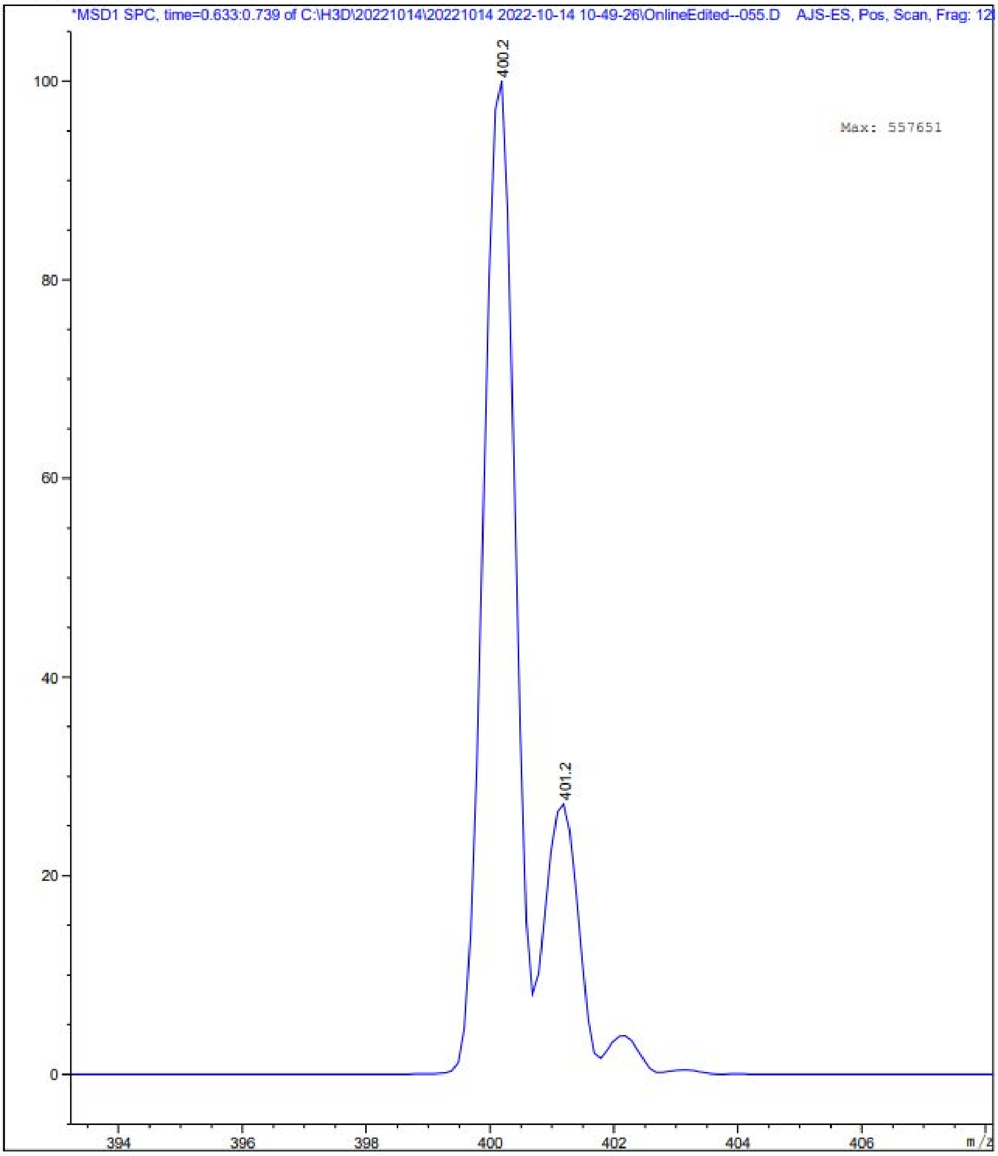

*MSD1 SPC, time=0.633:0.739 of C:\H3D\20221014\20221014 2022-10-14 10-49-26\OnlineEdited--055.D AJS-ES, Pos, Scan, Frag: 12
400.2
401.2
Max: 557651
100
80
60
40
20
0
394
396
398
400
402
404
406
m/z

- ^{1}H NMR spectrum of Compound **25**

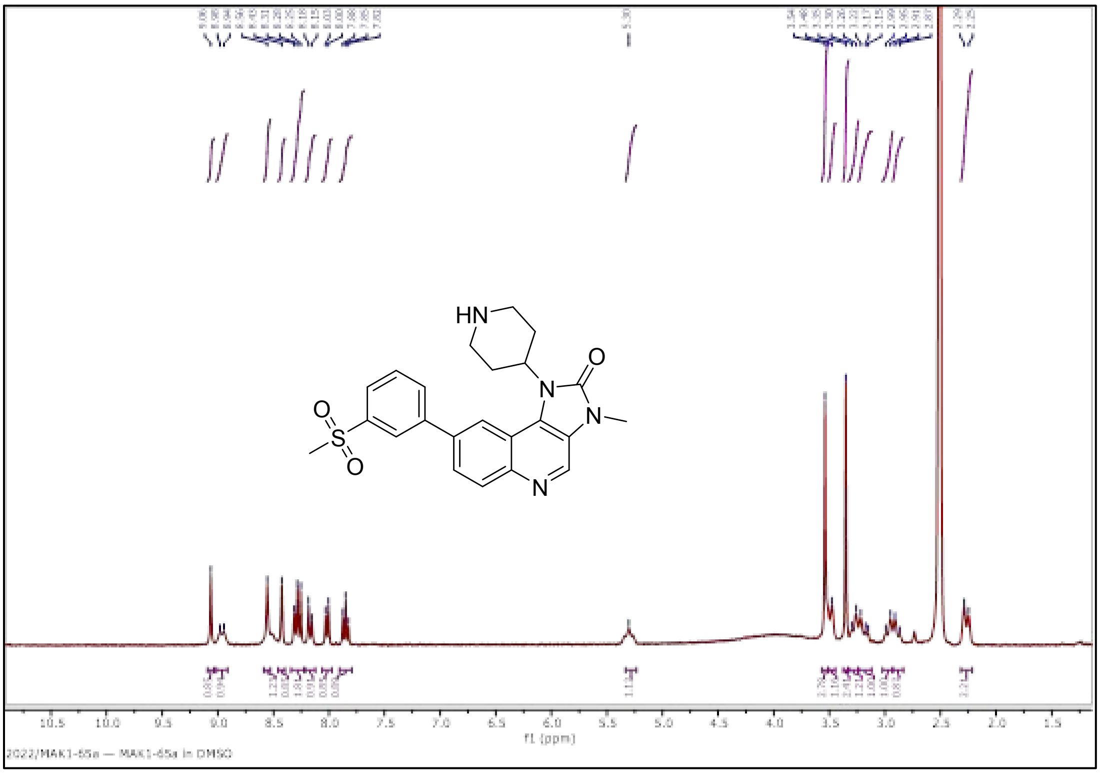

- ^{13}C NMR spectrum of Compound **25**

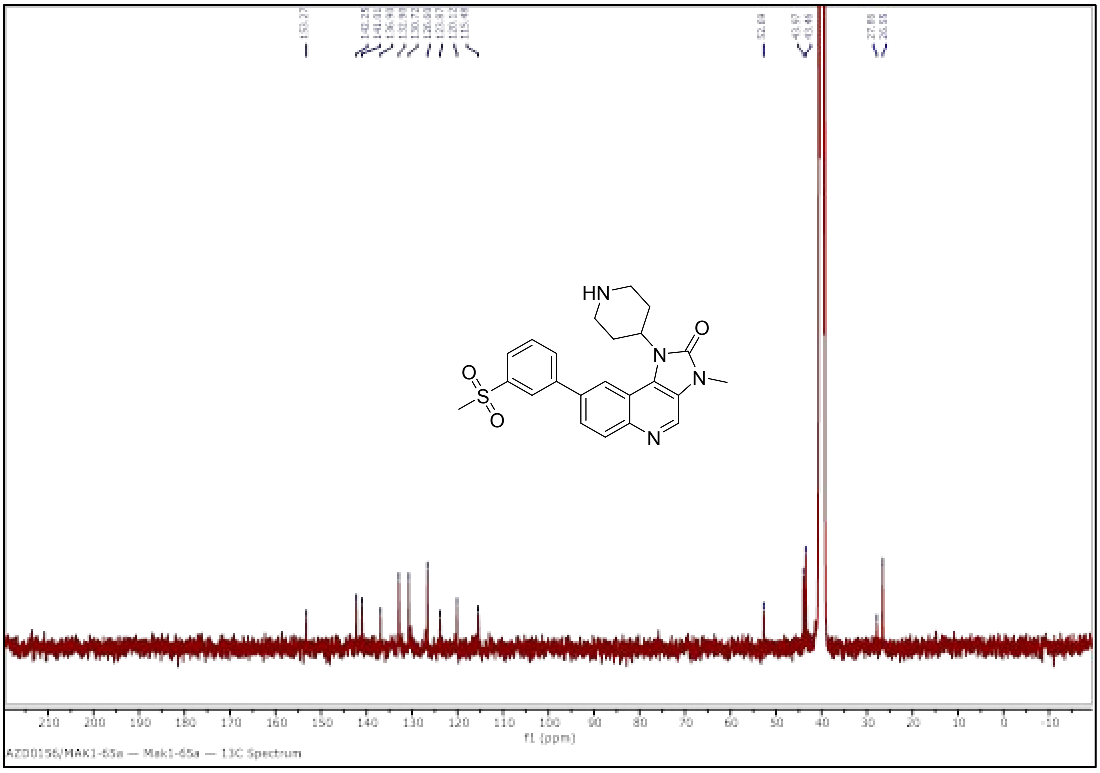

• HPLC trace of Compound **25**

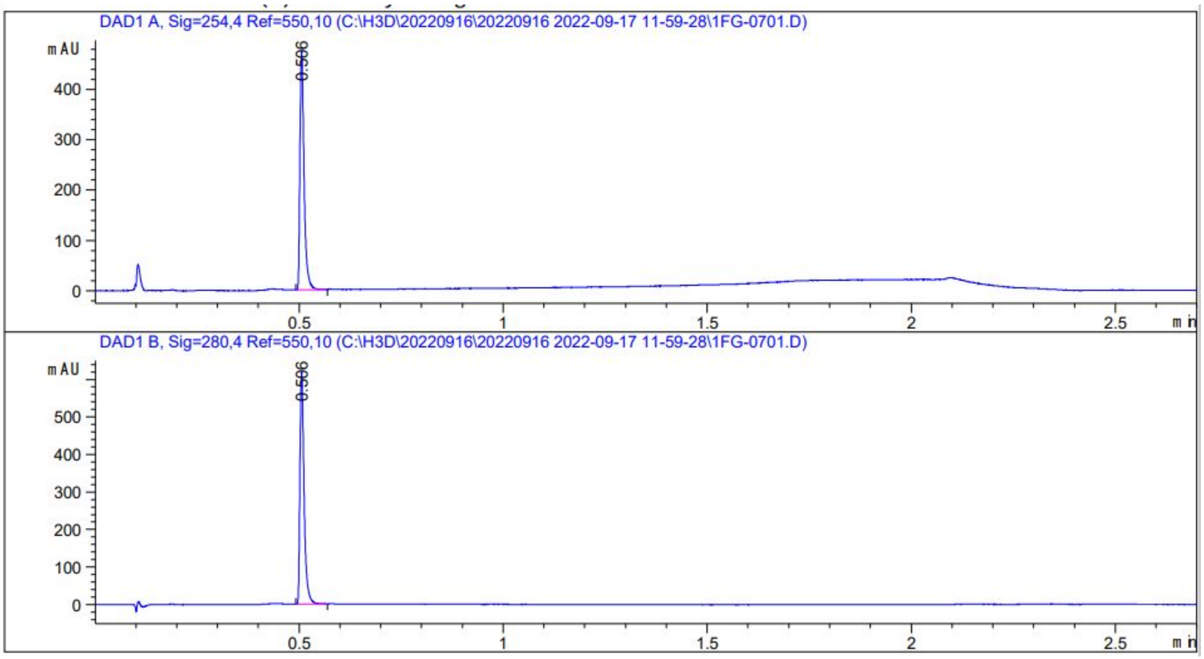

Peak #	RetTime [min]	Type	Width [min]	Area [mAU*s]	Height [mAU]	Area %
1	0.506	BB	0.0107	432.79840	615.83838	100.0000
Totals :				432.79840	615.83838	

MS Spectrum

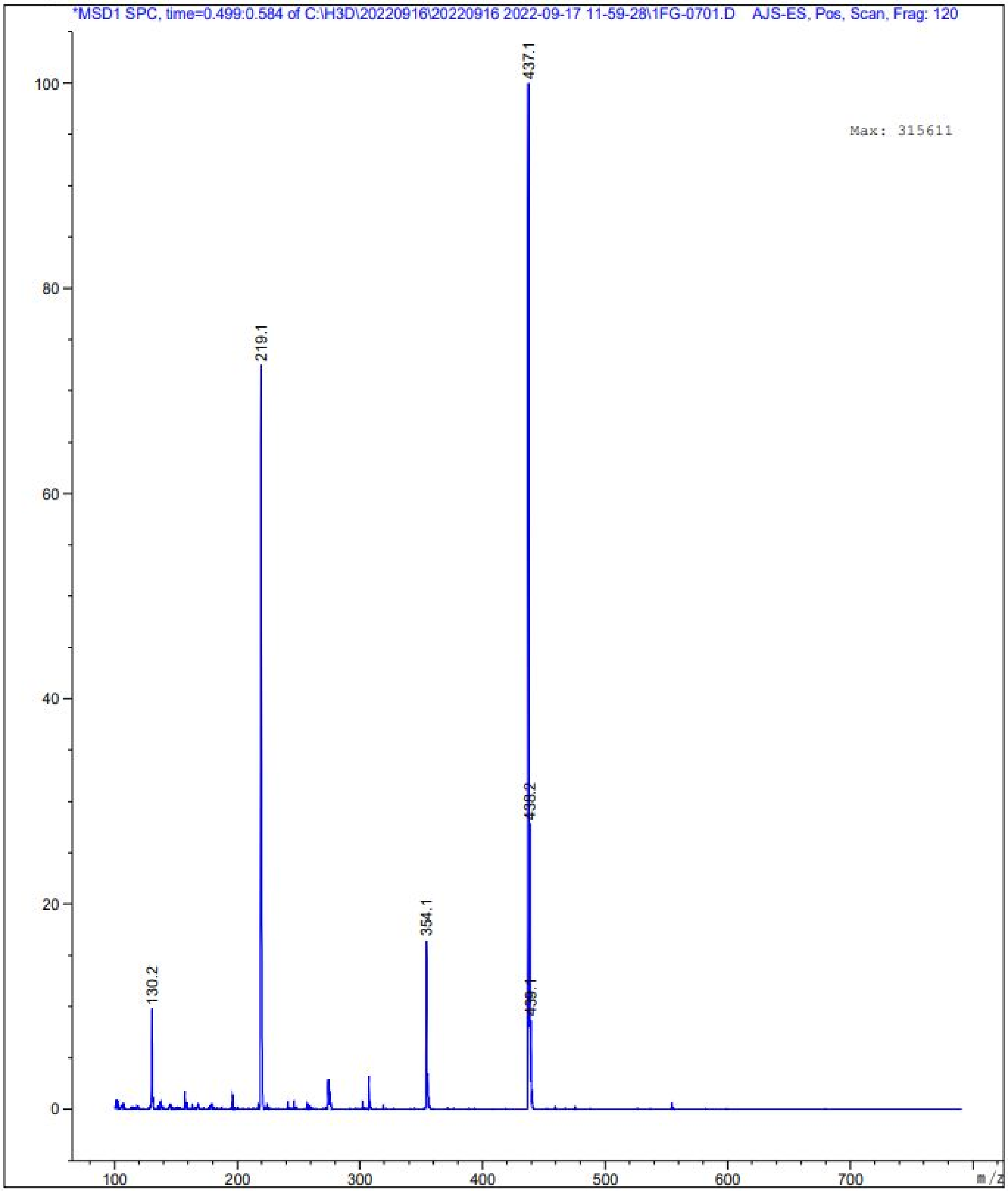
*MSD1 SPC, time=0.499:0.584 of C:\H3D\20220916\20220916 2022-09-17 11-59-28\1FG-0701.D AJS-ES, Pos, Scan, Frag: 120
Max: 315611
437.1
219.1
354.1
130.2
438.2
m/z

- ^{1}H NMR spectrum of Compound **26**

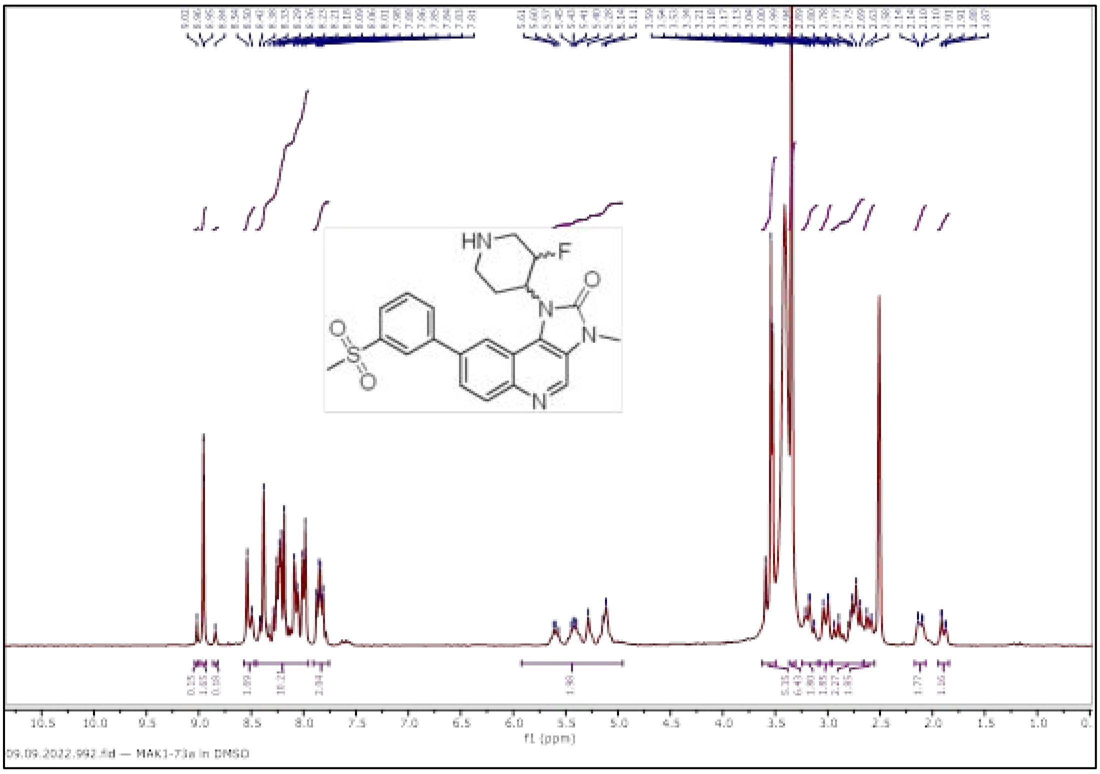

- ^{13}C NMR spectrum of Compound **26**

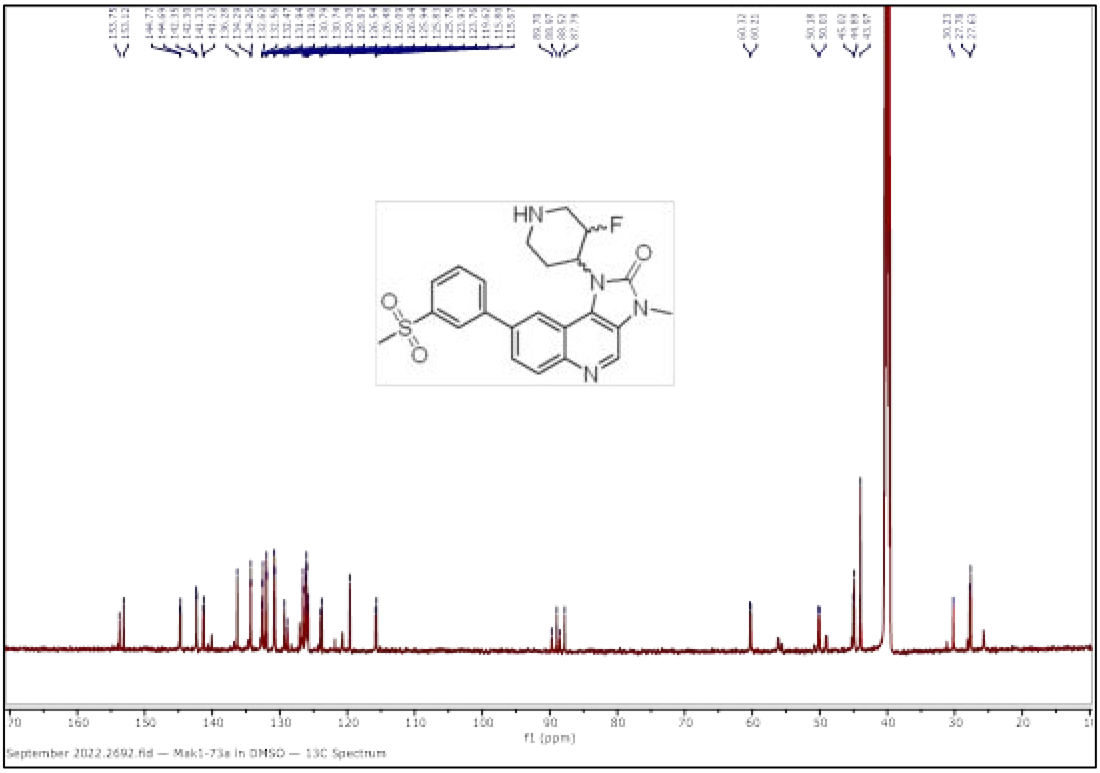

- HPLC trace of Compound **26**

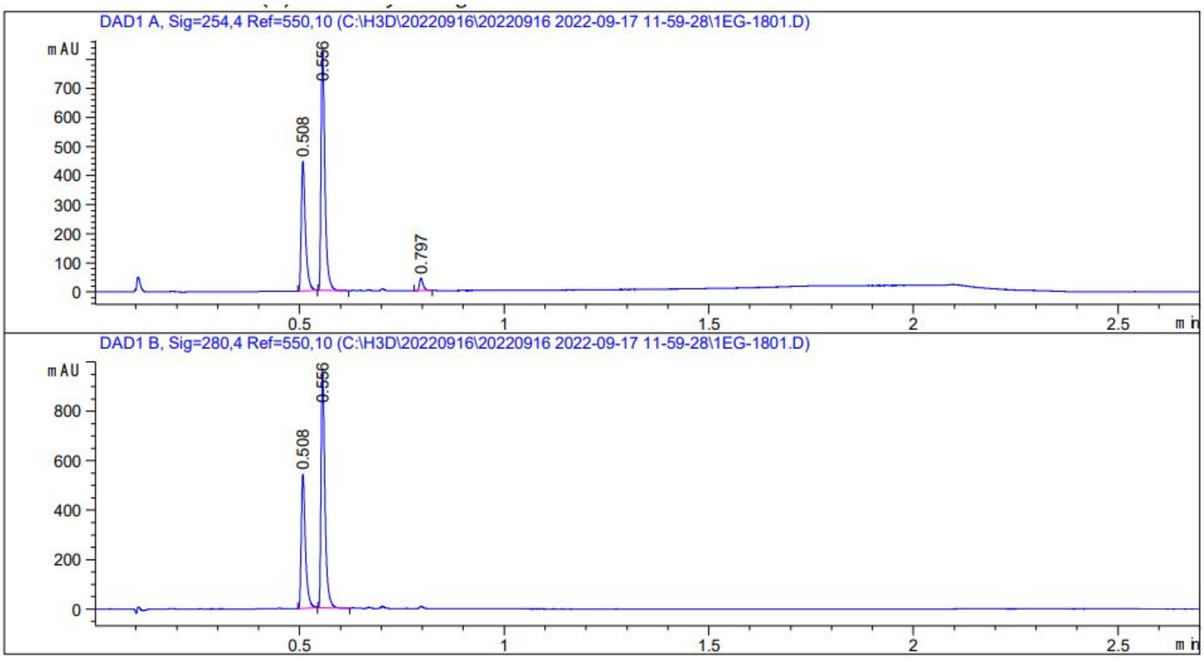

Peak #	RetTime [min]	Type	Width [min]	Area [mAU*s]	Height [mAU]	Area %
1	0.508	BB	0.0107	380.48535	541.08368	38.1461
2	0.556	BB	0.0101	616.95752	943.19684	61.8539
Totals :				997.44287	1484.28052	

MS Spectrum

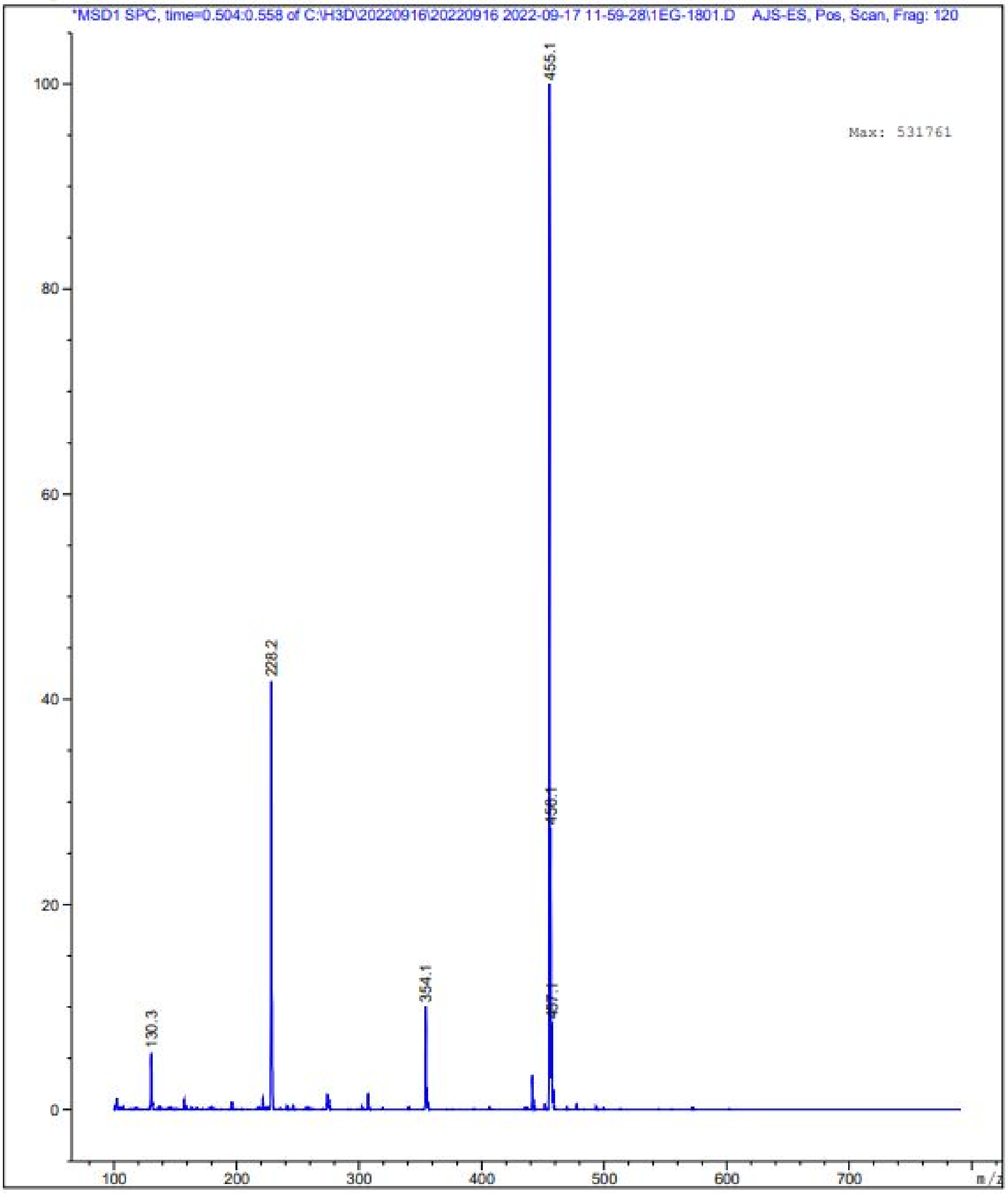
*MSD1 SPC, time=0.504:0.558 of C:\H3D\20220916\20220916 2022-09-17 11-59-28\1EG-1801.D AJS-ES, Pos, Scan, Frag: 120
Max: 531761
455.1
228.2
354.1
130.3
100
80
60
40
20
0
100
200
300
400
500
600
700
m/z

- ^{1}H NMR spectrum of Compound **27**

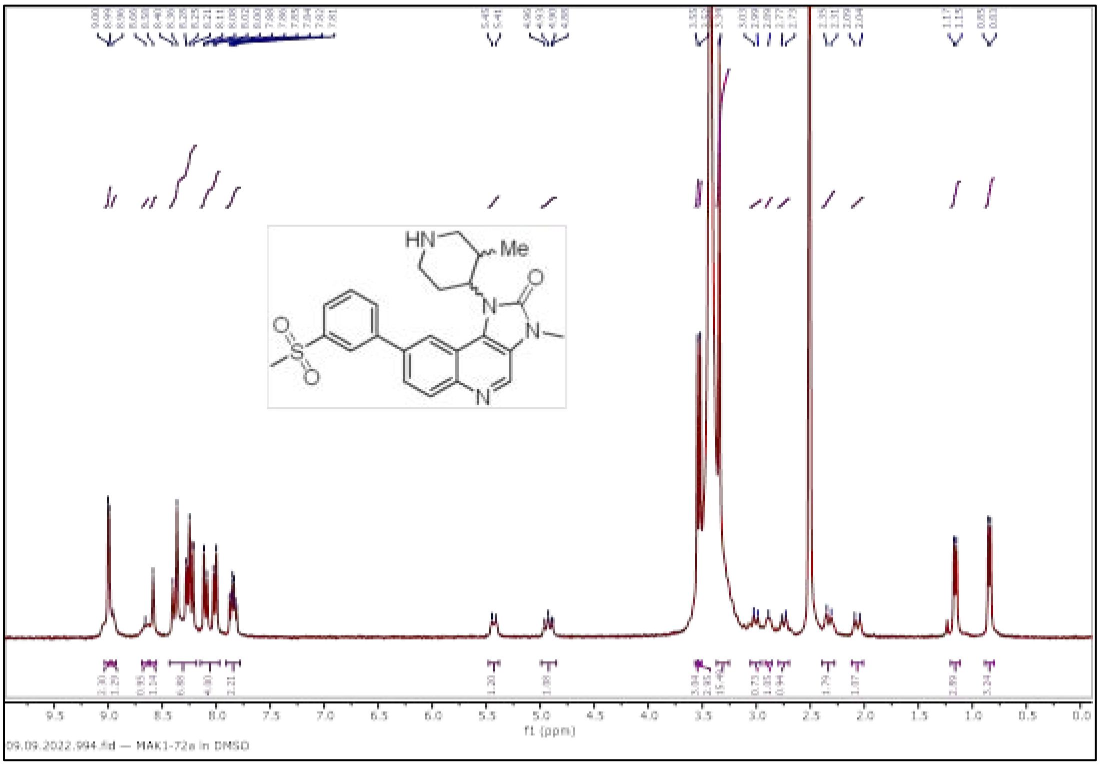

- ^{13}C NMR spectrum of Compound **27**

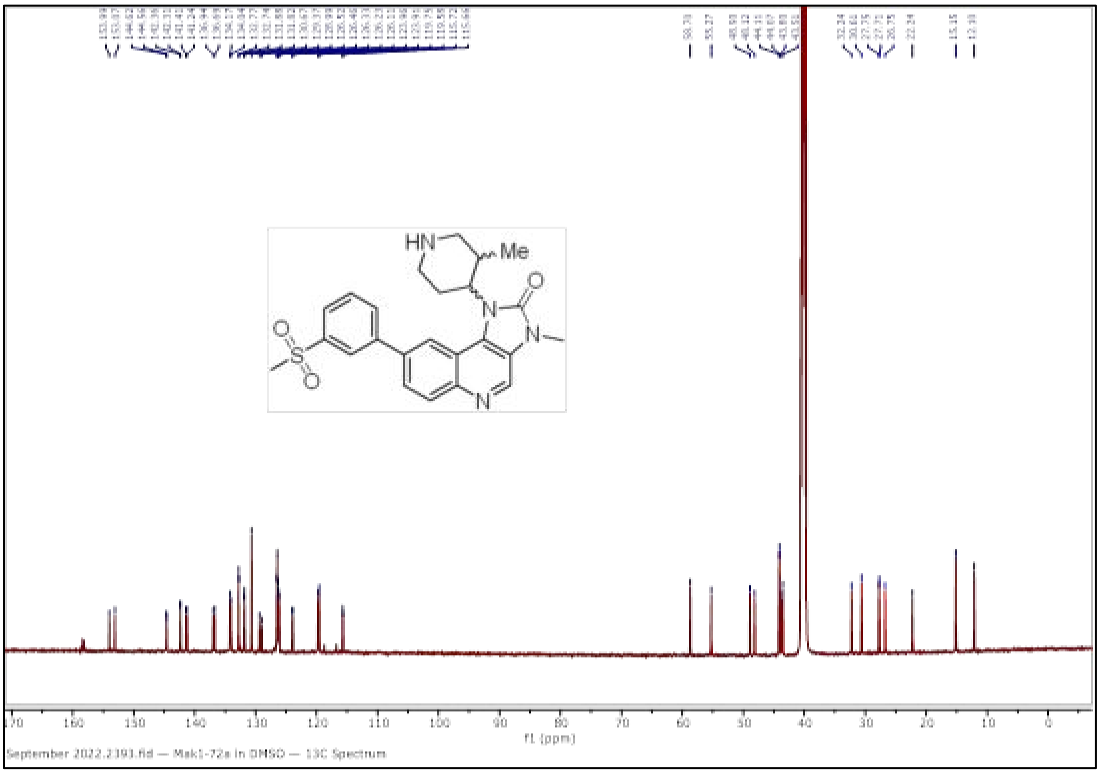

- HPLC trace of Compound **27**

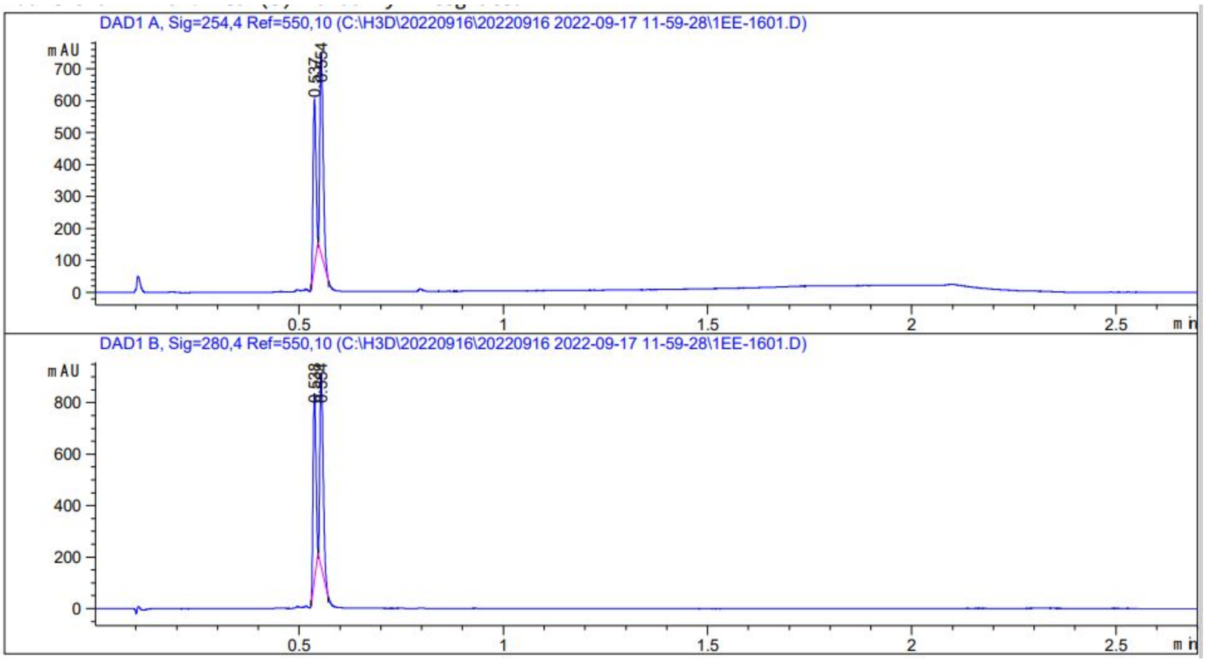

Peak #	RetTime [min]	Type	Width [min]	Area [mAU*s]	Height [mAU]	Area %
1	0.538	BB	8.43e-3	362.95398	714.64099	46.8582
2	0.554	BB	9.00e-3	411.62543	738.72217	53.1418
Totals :				774.57941	1453.36316	

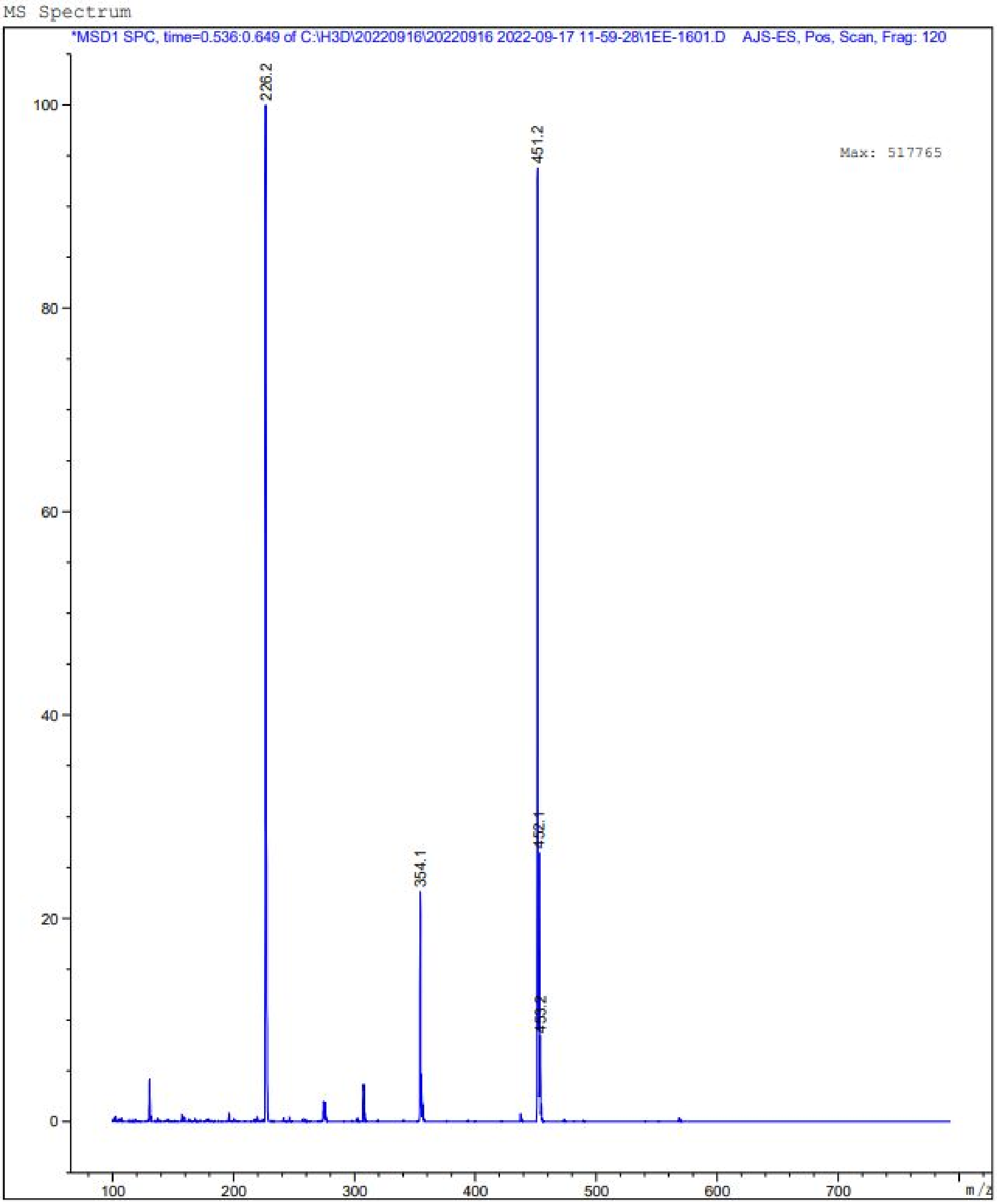
MS Spectrum
*MSD1 SPC, time=0.536:0.649 of C:\H3D\20220916\20220916 2022-09-17 11-59-28\1EE-1601.D AJS-ES, Pos, Scan, Frag: 120
Max: 517765
226.2
451.2
354.1
m/z

- ^{1}H NMR spectrum of Compound **28**

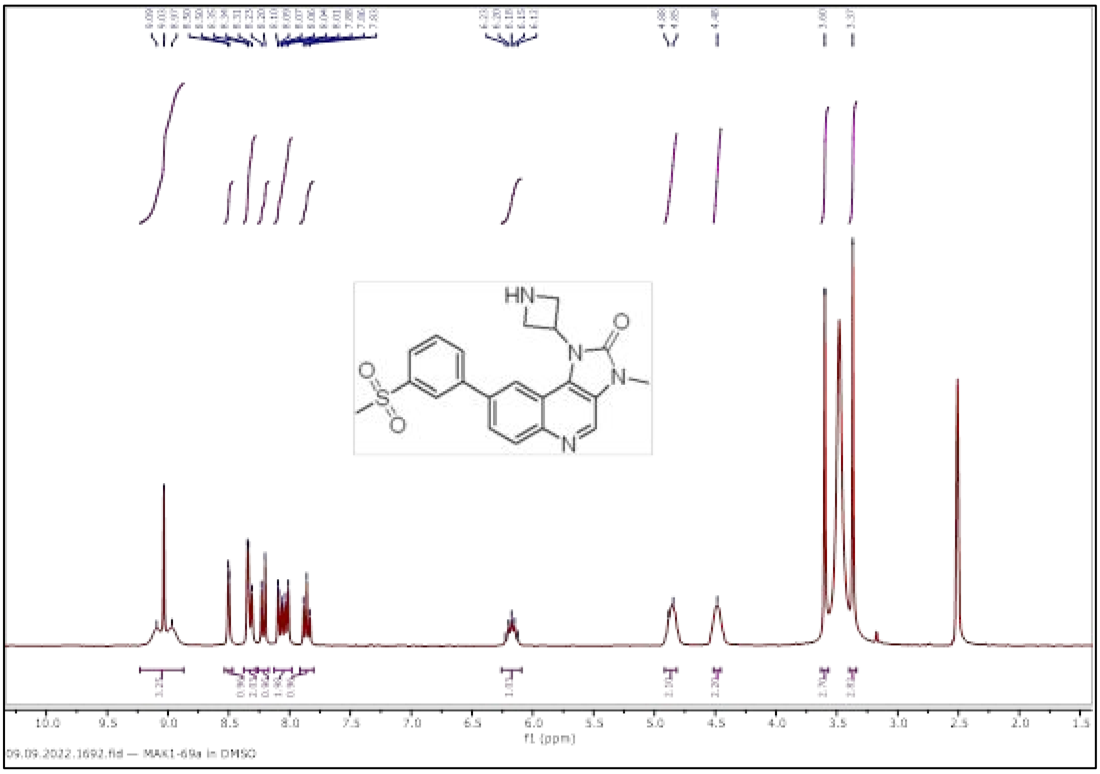

- ^{13}C NMR spectrum of Compound **28**

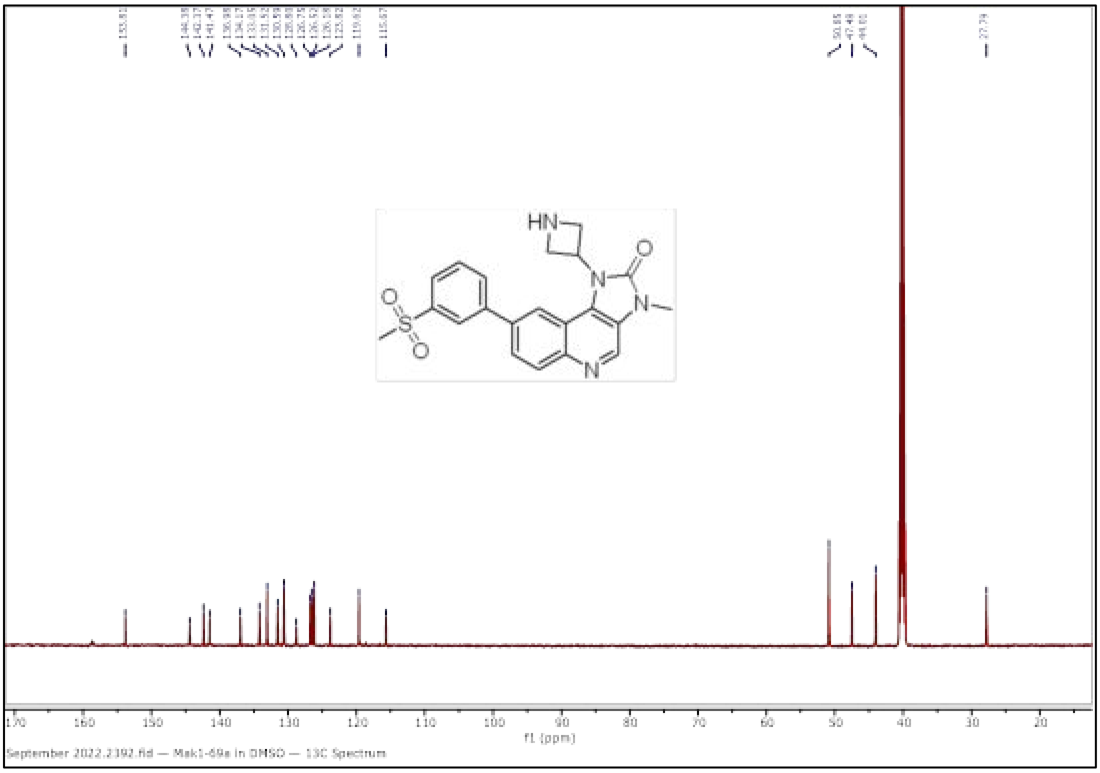

- HPLC trace of Compound **28**

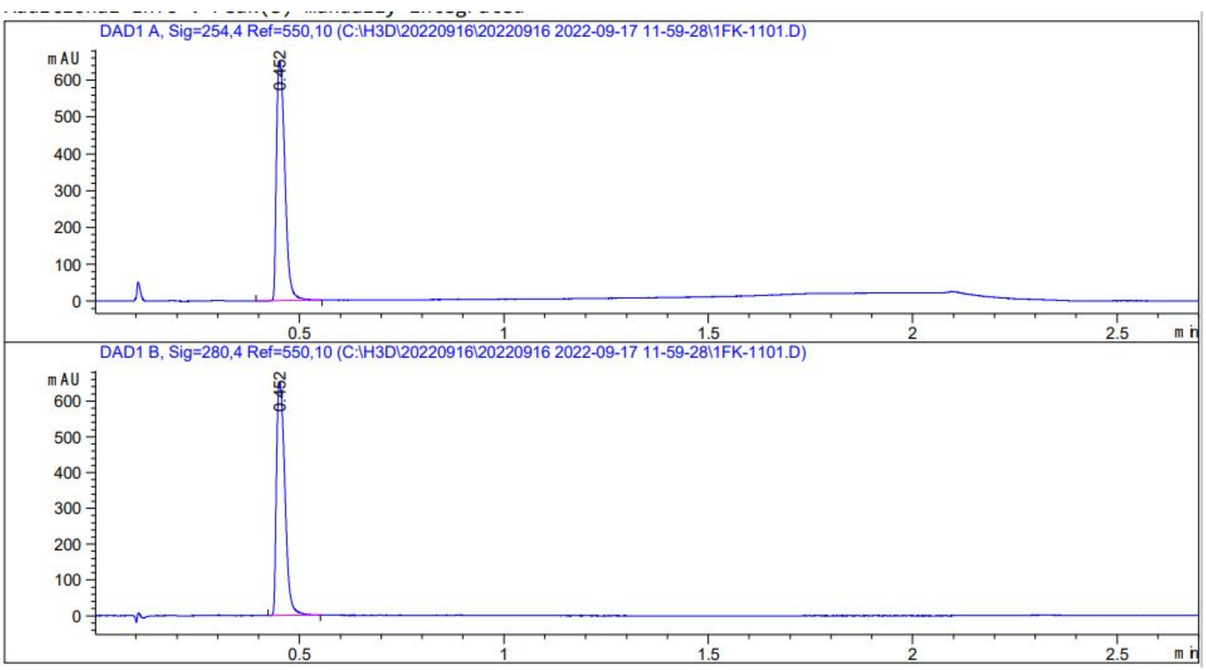

Peak #	RetTime [min]	Type	Width [min]	Area [mAU*s]	Height [mAU]	Area %
1	0.452	BB	0.0232	931.75757	650.25958	100.0000

Totals : 931.75757 650.25958

MS Spectrum

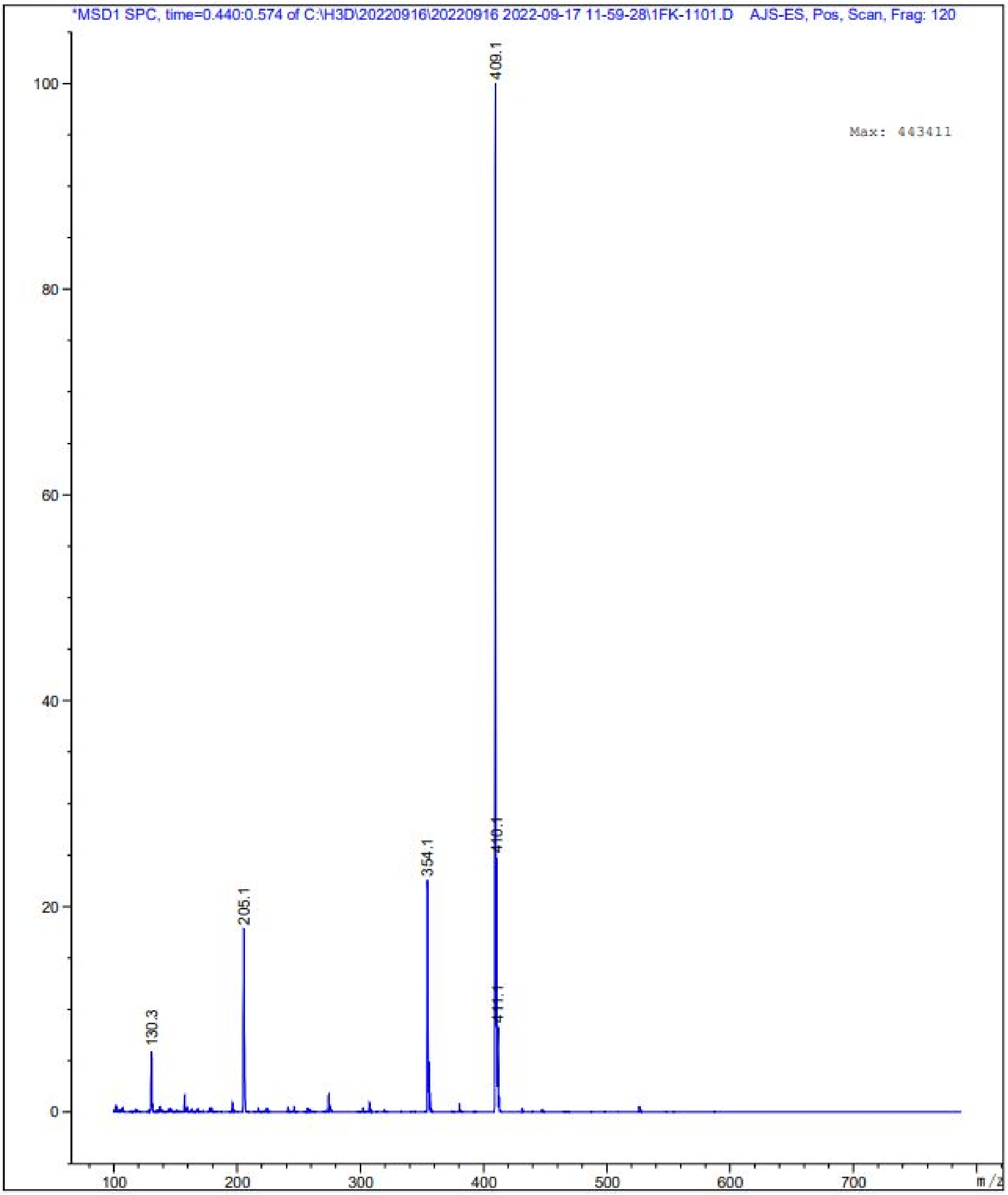

*MSD1 SPC, time=0.440:0.574 of C:\H3D\20220916\20220916 2022-09-17 11-59-28\1FK-1101.D AJS-ES, Pos, Scan, Frag: 120
Max: 443411
409.1
410.1
411.1
354.1
205.1
130.3
100
80
60
40
20
0
100
200
300
400
500
600
700
m/z

- ^{1}H NMR spectrum of Compound **29**

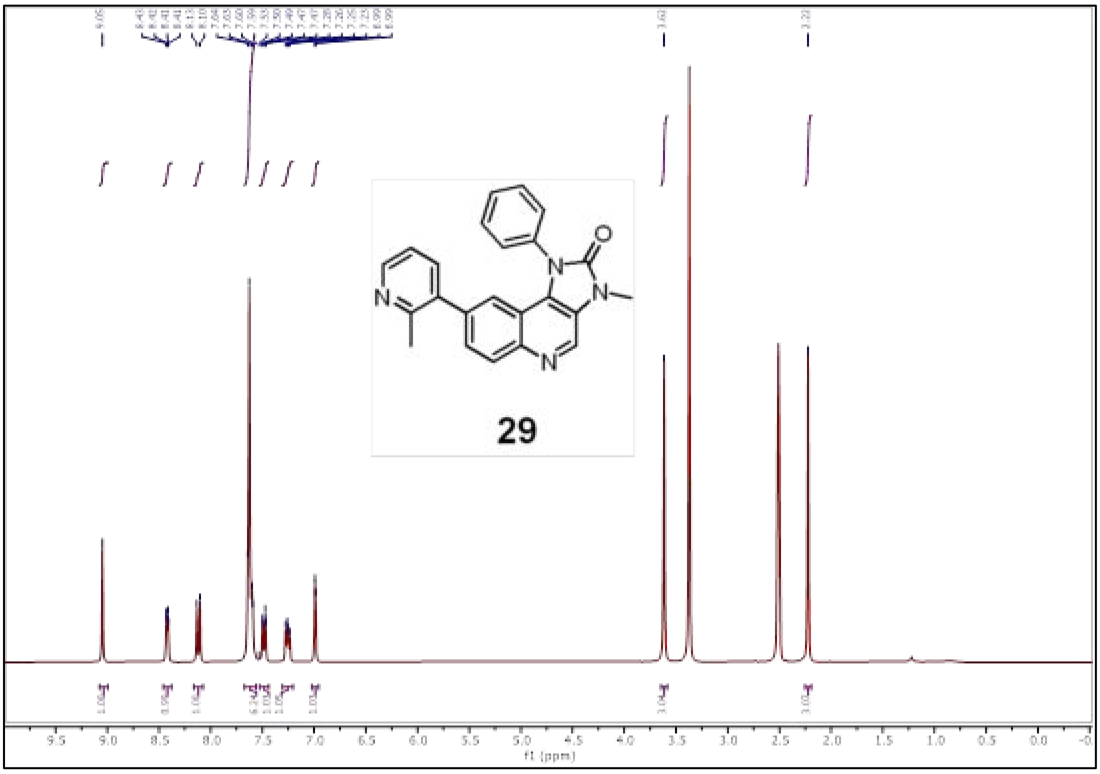

- ^{13}C NMR spectrum of Compound **29**

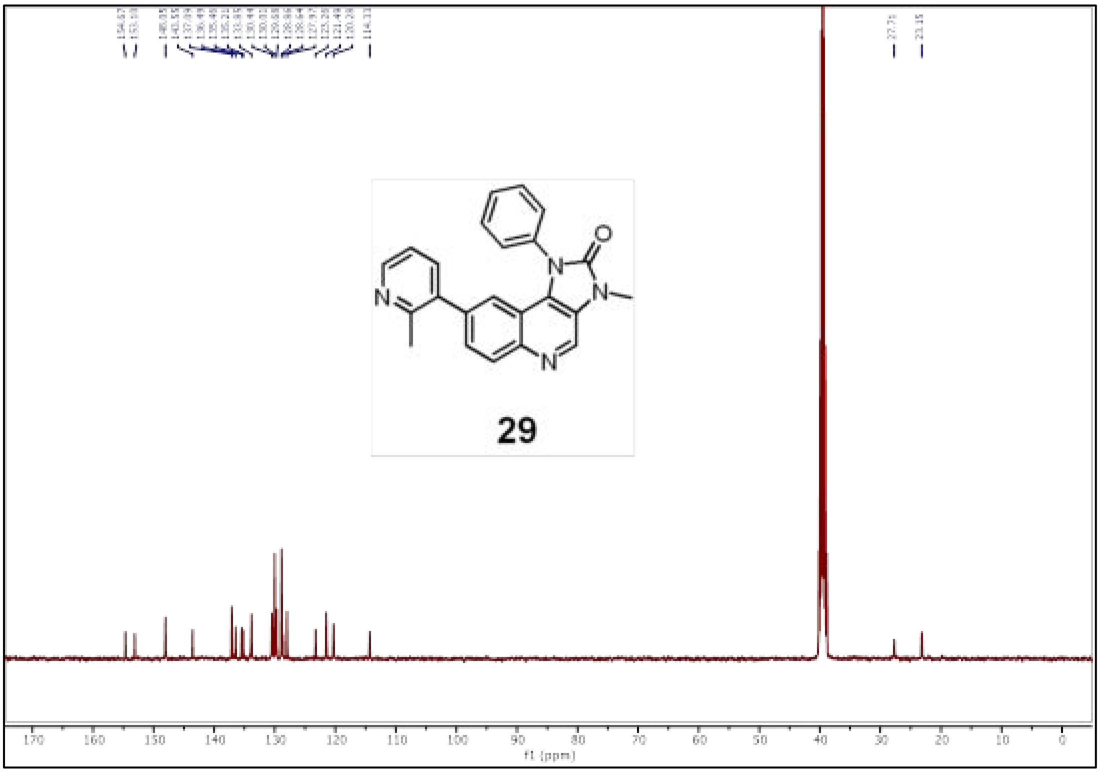

- HPLC trace of Compound **29**

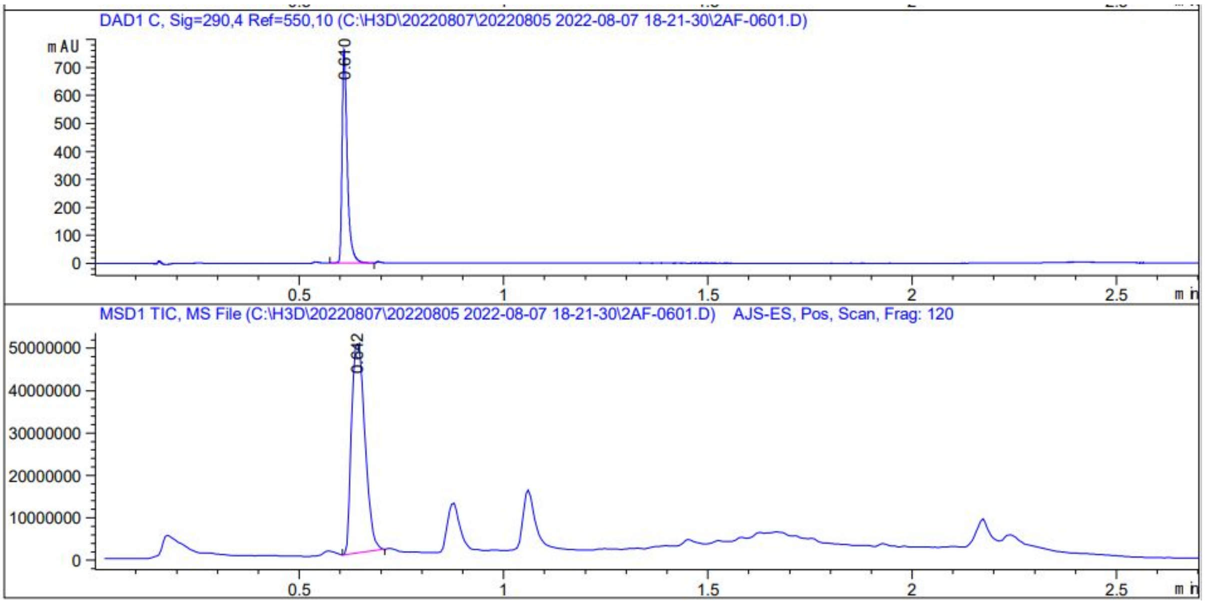

Peak #	RetTime [min]	Type	Width [min]	Area	Height	Area %
1	0.642	BB	0.0362	1.12684e8	4.95652e7	100.0000

Totals : 1.12684e8 4.95652e7

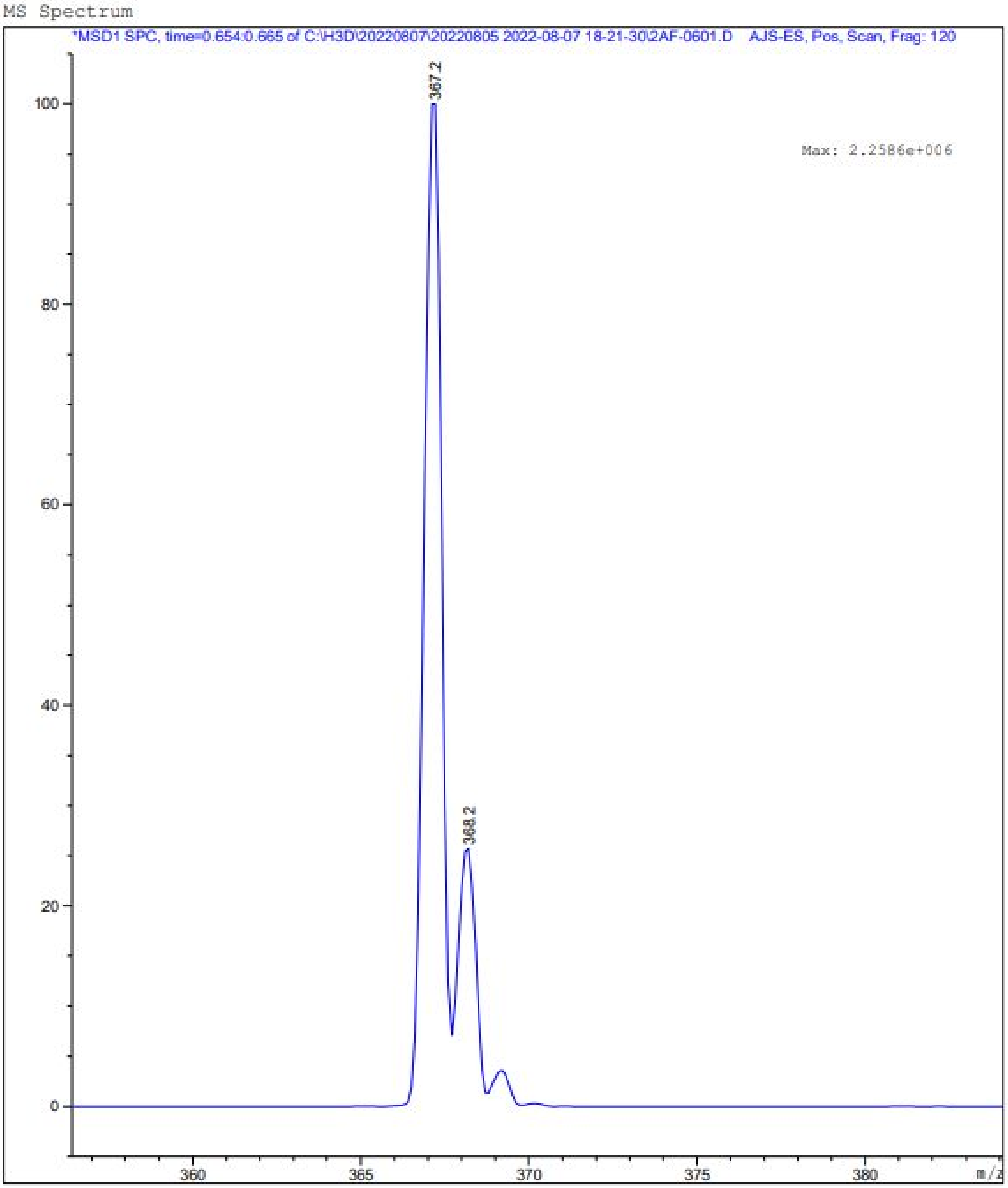

MS Spectrum
*MSD1 SPC, time=0.654:0.665 of C:\H3D\20220807\20220805 2022-08-07 18-21-30\2AF-0601.D AJS-ES, Pos, Scan, Frag: 120
367.2
368.2
Max: 2.2586e+006
m/z

- ^{1}H NMR spectrum of Compound **30**

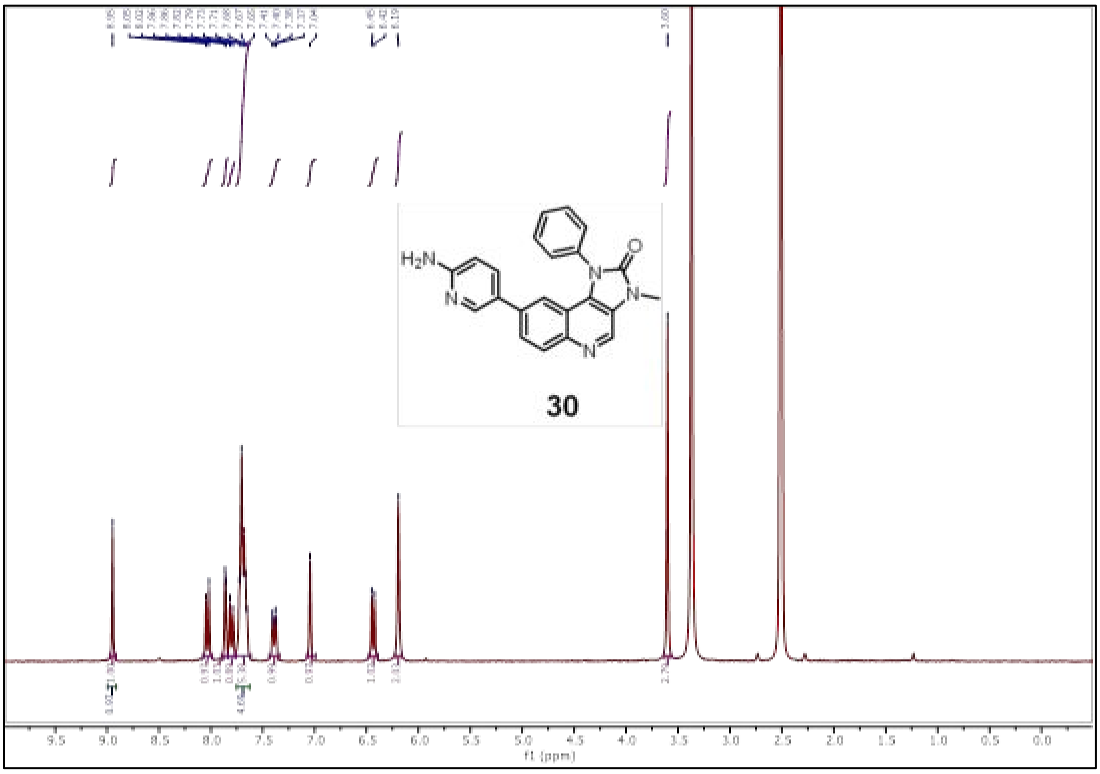

- ^{13}C NMR spectrum of Compound **30**

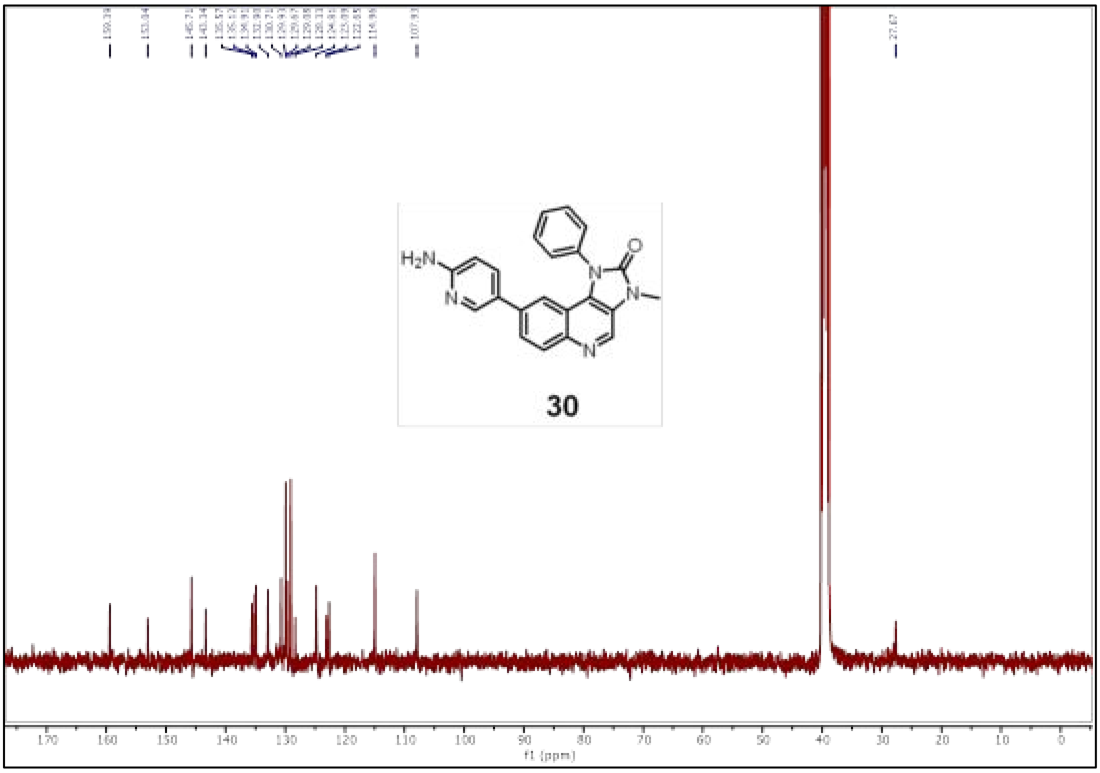

- HPLC trace of Compound **30**

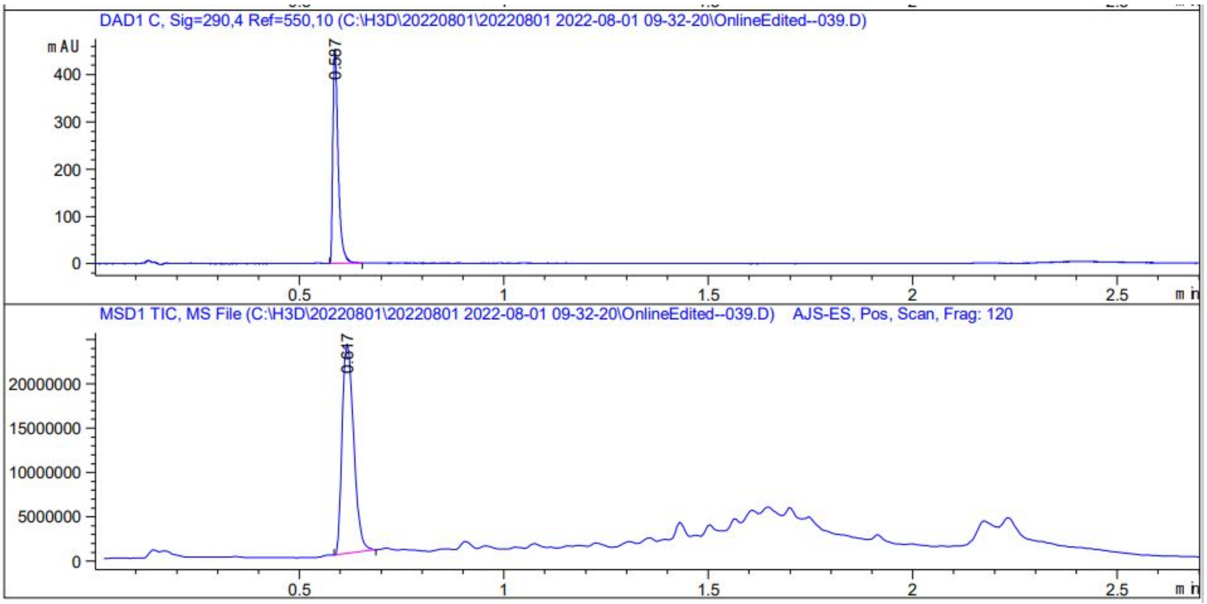

Peak #	RetTime [min]	Type	Width [min]	Area [mAU*s]	Height [mAU]	Area %
1	0.587	BB	0.0135	407.93018	452.59958	100.0000
Totals :				407.93018	452.59958	

MS Spectrum

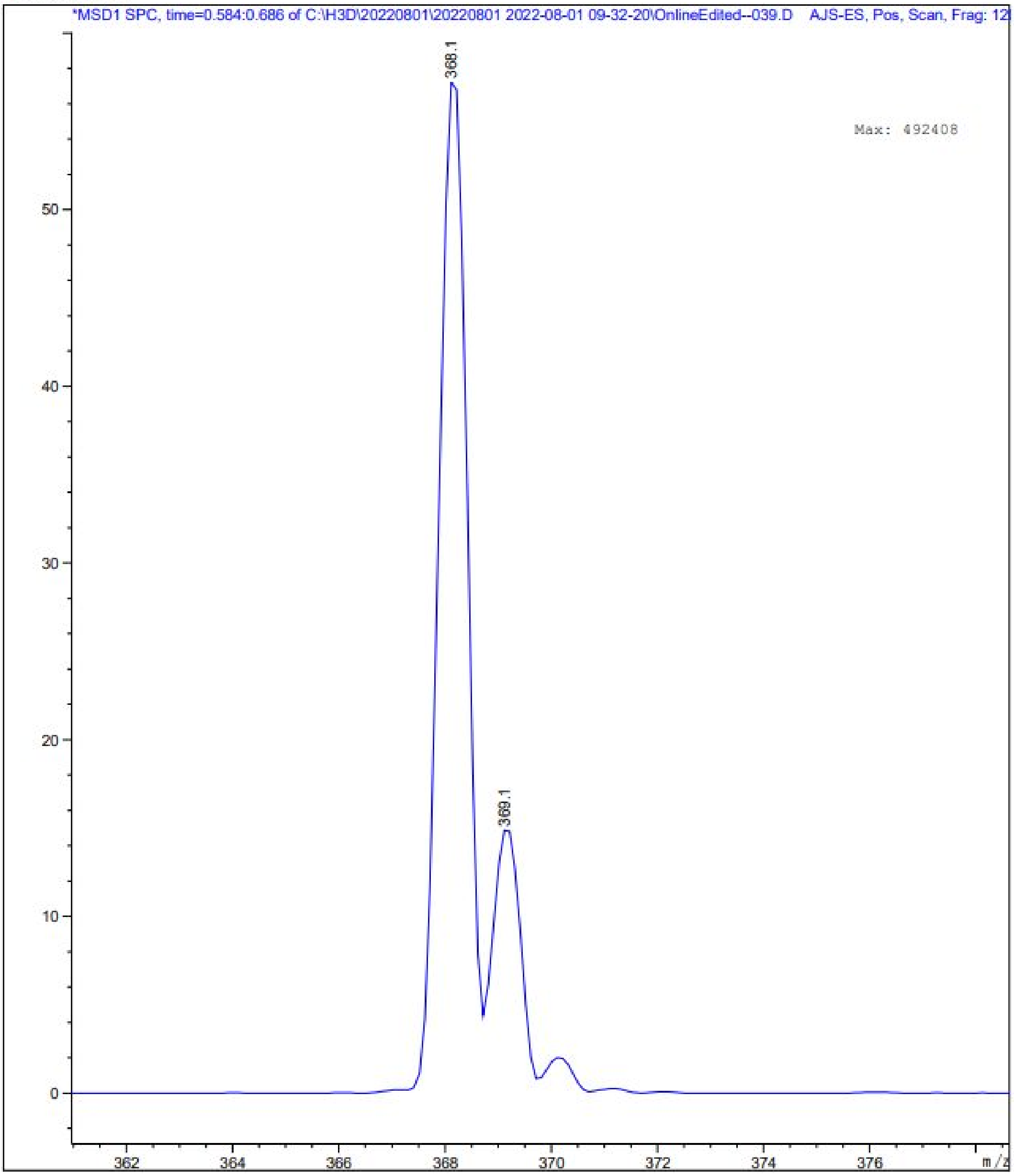
*MSD1 SPC, time=0.584:0.686 of C:\H3D\20220801\20220801 2022-08-01 09-32-20\OnlineEdited--039.D AJS-ES, Pos, Scan, Frag: 12
368.1
369.1
Max: 492408
50
40
30
20
10
0
362
364
366
368
370
372
374
376
m/z

21. References

(1) Leshabane, M.; Dziwornu, G. A.; Coertzen, D.; Reader, J.; Moyo, P.; van der Watt, M.; Chisanga, K.; Nsanzubuhoro, C.; Ferger, R.; Erlank, E.; Venter, N.; Koekemoer, L.; Chibale, K.; Birkholtz, L.-M. Benzimidazole Derivatives Are Potent against Multiple Life Cycle Stages of *Plasmodium Falciparum* Malaria Parasites. *ACS Infect Dis* **2021**, *7* (7), 1945–1955. https://doi.org/10.1021/acsinfecdis.0c00910.

(2) Snyder, C.; Chollet, J.; Santo-Tomas, J.; Scheurer, C.; Wittlin, S. In Vitro and in Vivo Interaction of Synthetic Peroxide RBx11160 (OZ277) with Piperaquine in Plasmodium Models. *Exp Parasitol* **2007**, *115* (3), 296–300. https://doi.org/10.1016/j.exppara.2006.09.016.

(3) Duffey, M.; Blasco, B.; Burrows, J. N.; Wells, T. N. C.; Fidock, D. A.; Leroy, D. Assessing Risks of Plasmodium Falciparum Resistance to Select Next-Generation Antimalarials. *Trends Parasitol* **2021**, *37* (8), 709–721. https://doi.org/10.1016/j.pt.2021.04.006.

(4) Ding, X. C.; Ubben, D.; Wells, T. N. A Framework for Assessing the Risk of Resistance for Anti-Malarials in Development. *Malar J* **2012**, *11* (1), 292. https://doi.org/10.1186/1475-2875-11-292.

(5) Cevenini, L.; Camarda, G.; Michelini, E.; Siciliano, G.; Calabretta, M. M.; Bona, R.; Kumar, T. R. S.; Cara, A.; Branchini, B. R.; Fidock, D. A.; Roda, A.; Alano, P. Multicolor Bioluminescence Boosts Malaria Research: Quantitative Dual-Color Assay and Single-Cell Imaging in *Plasmodium Falciparum* Parasites. *Anal Chem* **2014**, *86* (17), 8814–8821. https://doi.org/10.1021/ac502098w.

(6) Reader, J.; van der Watt, M. E.; Taylor, D.; Le Manach, C.; Mittal, N.; Ottilie, S.; Theron, A.; Moyo, P.; Erlank, E.; Nardini, L.; Venter, N.; Lauterbach, S.; Bezuidenhout, B.; Horatscheck, A.; van Heerden, A.; Spillman, N. J.; Cowell, A. N.; Connacher, J.; Opperman, D.; Orchard, L. M.; Llinás, M.; Istvan, E. S.; Goldberg, D. E.; Boyle, G. A.; Calvo, D.; Mancama, D.; Coetzer, T. L.; Winzeler, E. A.; Duffy, J.; Koekemoer, L. L.; Basarab, G.; Chibale, K.; Birkholtz, L.-M. Multistage and Transmission-Blocking Targeted Antimalarials Discovered from the Open-Source MMV Pandemic Response Box. *Nat Commun* **2021**, *12* (1), 269. https://doi.org/10.1038/s41467-020-20629-8.

(7) Delves, M. J.; Straschil, U.; Ruecker, A.; Miguel-Blanco, C.; Marques, S.; Dufour, A. C.; Baum, J.; Sinden, R. E. Routine in Vitro Culture of P. Falciparum Gametocytes to Evaluate Novel Transmission-Blocking Interventions. *Nat Protoc* **2016**, *11* (9), 1668–1680. https://doi.org/10.1038/nprot.2016.096.

(8) Delves, M. J.; Miguel-Blanco, C.; Matthews, H.; Molina, I.; Ruecker, A.; Yahiya, S.; Straschil, U.; Abraham, M.; León, M. L.; Fischer, O. J.; Rueda-Zubiaurre, A.; Brandt, J. R.; Cortés, Á.; Barnard, A.; Fuchter, M. J.; Calderón, F.; Winzeler, E. A.; Sinden, R. E.; Herreros, E.; Gamo, F. J.; Baum, J. A High Throughput Screen for Next-Generation Leads Targeting Malaria Parasite Transmission. *Nat Commun* **2018**, *9* (1), 3805. https://doi.org/10.1038/s41467-018-05777-2.

(9) Arendse, L. B.; Murithi, J. M.; Qahash, T.; Pasaje, C. F. A.; Godoy, L. C.; Dey, S.; Gibhard, L.; Ghidelli-Disse, S.; Drewes, G.; Bantscheff, M.; Lafuente-Monasterio, M. J.; Fienberg, S.; Wambua, L.; Gachuhi, S.; Coertzen, D.; van der Watt, M.; Reader, J.; Aswat, A. S.; Erlank, E.; Venter, N.; Mittal, N.; Luth, M. R.; Ottilie, S.; Winzeler, E. A.; Koekemoer, L. L.; Birkholtz, L.-M.; Niles, J. C.; Llinás, M.; Fidock, D. A.; Chibale, K. The Anticancer Human MTOR Inhibitor Sapanisertib Potently Inhibits Multiple *Plasmodium* Kinases and Life Cycle Stages. *Sci Transl Med* **2022**, *14* (667). https://doi.org/10.1126/scitranslmed.abo7219.

(10) Hunt, R. H.; Brooke, B. D.; Pillay, C.; Koekemoer, L. L.; Coetzee, M. Laboratory Selection for and Characteristics of Pyrethroid Resistance in the Malaria Vector Anopheles Funestus. *Med Vet Entomol* **2005**, *19* (3), 271–275. https://doi.org/10.1111/j.1365-2915.2005.00574.x.

(11) Coetzee, M.; Hunt, R. H.; Wilkerson, R.; Della Torre, A.; Coulibaly, M. B.; Besansky, N. J. Anopheles Coluzzii and Anopheles Amharicus, New Members of the Anopheles Gambiae Complex. *Zootaxa* **2013**, *3619*, 246–274.

(12) Fanello, C.; Santolamazza, F.; della Torre, A. Simultaneous Identification of Species and Molecular Forms of the Anopheles Gambiae Complex by PCR-RFLP. *Med Vet Entomol* **2002**, *16* (4), 461–464. https://doi.org/10.1046/j.1365-2915.2002.00393.x.

(13) Miura, K.; Swihart, B. J.; Deng, B.; Zhou, L.; Pham, T. P.; Diouf, A.; Burton, T.; Fay, M. P.; Long, C. A. Transmission-Blocking Activity Is Determined by Transmission-Reducing Activity and Number of Control Oocysts in Plasmodium Falciparum Standard Membrane-Feeding Assay. *Vaccine* **2016**, *34* (35), 4145–4151. https://doi.org/10.1016/j.vaccine.2016.06.066.

(14) Brunschwig, C.; Lawrence, N.; Taylor, D.; Abay, E.; Njoroge, M.; Basarab, G. S.; Le Manach, C.; Paquet, T.; Cabrera, D. G.; Nchinda, A. T.; de Kock, C.; Wiesner, L.; Denti, P.; Waterson, D.; Blasco, B.; Leroy, D.; Witty, M. J.; Donini, C.; Duffy, J.; Wittlin, S.; White, K. L.; Charman, S. A.; Jiménez-Díaz, M. B.; Angulo-Barturen, I.; Herreros, E.; Gamo, F. J.; Rochford, R.; Mancama, D.; Coetzer, T. L.; van der Watt, M. E.; Reader, J.; Birkholtz, L.-M.; Marsh, K. C.; Solapure, S. M.; Burke, J. E.; McPhail, J. A.;

Vanaerschot, M.; Fidock, D. A.; Fish, P. V.; Siegl, P.; Smith, D. A.; Wirjanata, G.; Noviyanti, R.; Price, R. N.; Marfurt, J.; Silue, K. D.; Street, L. J.; Chibale, K. UCT943, a Next-Generation Plasmodium Falciparum PI4K Inhibitor Preclinical Candidate for the Treatment of Malaria. *Antimicrob Agents Chemother* **2018**, *62* (9). https://doi.org/10.1128/AAC.00012-18.

(15) Mosmann, T. Rapid Colorimetric Assay for Cellular Growth and Survival: Application to Proliferation and Cytotoxicity Assays. *J Immunol Methods* **1983**, *65* (1–2), 55–63. https://doi.org/10.1016/0022-1759(83)90303-4.

(16) McNamara, C. W.; Lee, M. C. S.; Lim, C. S.; Lim, S. H.; Roland, J.; Nagle, A.; Simon, O.; Yeung, B. K. S.; Chatterjee, A. K.; McCormack, S. L.; Manary, M. J.; Zeeman, A.-M.; Dechering, K. J.; Kumar, T. R. S.; Henrich, P. P.; Gagaring, K.; Ibanez, M.; Kato, N.; Kuhen, K. L.; Fischli, C.; Rottmann, M.; Plouffe, D. M.; Bursulaya, B.; Meister, S.; Rameh, L.; Trappe, J.; Haasen, D.; Timmerman, M.; Sauerwein, R. W.; Suwanarusk, R.; Russell, B.; Renia, L.; Nosten, F.; Tully, D. C.; Kocken, C. H. M.; Glynne, R. J.; Bodenreider, C.; Fidock, D. A.; Diagana, T. T.; Winzeler, E. A. Targeting Plasmodium PI(4)K to Eliminate Malaria. *Nature* **2013**, *504* (7479), 248–253. https://doi.org/10.1038/nature12782.

(17) Cheuka, P. M.; Centani, L.; Arendse, L. B.; Fienberg, S.; Wambua, L.; Renga, S. S.; Dziwornu, G. A.; Kumar, M.; Lawrence, N.; Taylor, D.; Wittlin, S.; Coertzen, D.; Reader, J.; van der Watt, M.; Birkholtz, L.-M.; Chibale, K. New Amidated 3,6-Diphenylated Imidazopyridazines with Potent Antiplasmodium Activity Are Dual Inhibitors of *Plasmodium* Phosphatidylinositol-4-Kinase and CGMP-Dependent Protein Kinase. *ACS Infect Dis* **2021**, *7* (1), 34–46. https://doi.org/10.1021/acsinfecdis.0c00481.

(18) Mathew, J.; Zhou, B.; Haney, R. S.; Kunz, K. A.; Do Amaral, L. S.; Roy Chowdhury, R.; Butler, J. H.; Li, H.; Chakraborty, A. J.; Tabassum, A.; Bremers, E. K.; Merino, E. F.; Coyle, R.; Lee, M. C. S.; Baud, D.; Brand, S.; Totrov, M.; Cassera, M. B.; Carlier, P. R. β-Carboline-3-Carboxamide Antimalarials: Structure–Activity Relationship, ADME-Tox Studies, and Resistance Profiling. *ACS Infect Dis* **2024**, *10* (11), 3951–3962. https://doi.org/10.1021/acsinfecdis.4c00653.

(19) Hill, A. P.; Young, R. J. Getting Physical in Drug Discovery: A Contemporary Perspective on Solubility and Hydrophobicity. *Drug Discov Today* **2010**, *15* (15–16), 648–655. https://doi.org/10.1016/j.drudis.2010.05.016.

(20) Alelyunas, Y. W.; Pelosi-Kilby, L.; Turcotte, P.; Kary, M.-B.; Spreen, R. C. A High Throughput Dried DMSO LogD Lipophilicity Measurement Based on 96-Well Shake-Flask and Atmospheric Pressure Photoionization Mass Spectrometry Detection. *J*

Chromatogr A **2010**, *1217* (12), 1950–1955. https://doi.org/10.1016/j.chroma.2010.01.071.

(21) Obach, R. S. Prediction of Human Clearance of Twenty-Nine Drugs from Hepatic Microsomal Intrinsic Clearance Data: An Examination of in Vitro Half-Life Approach and Nonspecific Binding to Microsomes. *Drug Metab Dispos* **1999**, *27* (11), 1350–1359.

(22) Jiménez-Díaz, M. B.; Mulet, T.; Viera, S.; Gómez, V.; Garuti, H.; Ibáñez, J.; Alvarez-Doval, A.; Shultz, L. D.; Martínez, A.; Gargallo-Viola, D.; Angulo-Barturen, I. Improved Murine Model of Malaria Using *Plasmodium Falciparum* Competent Strains and Non-Myelodepleted NOD- *Scid IL2R* γ *null* Mice Engrafted with Human Erythrocytes. *Antimicrob Agents Chemother* **2009**, *53* (10), 4533–4536. https://doi.org/10.1128/AAC.00519-09.

(23) Angulo-Barturen, I.; Jiménez-Díaz, M. B.; Mulet, T.; Rullas, J.; Herreros, E.; Ferrer, S.; Jiménez, E.; Mendoza, A.; Regadera, J.; Rosenthal, P. J.; Bathurst, I.; Pompliano, D. L.; Gómez de las Heras, F.; Gargallo-Viola, D. A Murine Model of Falciparum-Malaria by In Vivo Selection of Competent Strains in Non-Myelodepleted Mice Engrafted with Human Erythrocytes. *PLoS One* **2008**, *3* (5), e2252. https://doi.org/10.1371/journal.pone.0002252.

(24) Jiménez-Díaz, M. B.; Mulet, T.; Gómez, V.; Viera, S.; Alvarez, A.; Garuti, H.; Vázquez, Y.; Fernández, A.; Ibáñez, J.; Jiménez, M.; Gargallo-Viola, D.; Angulo-Barturen, I. Quantitative Measurement of *Plasmodium* -infected Erythrocytes in Murine Models of Malaria by Flow Cytometry Using Bidimensional Assessment of SYTO-16 Fluorescence. *Cytometry Part A* **2009**, *75A* (3), 225–235. https://doi.org/10.1002/cyto.a.20647.

(25) Delyon, B.; Lavielle, M.; Moulines, E. Convergence of a Stochastic Approximation Version of the EM Algorithm. *The Annals of Statistics* **1999**, *27* (1). https://doi.org/10.1214/aos/1018031103.
